# Unusual Chemodivergence in the Amination of 2-Chloro-1,10-Phenanthroline with Di- and Polyamines: Comparison of the Pd-Catalyzed and Catalyst-Free Pathways

**DOI:** 10.3390/molecules31183344

**Published:** 2026-09-21

**Authors:** Violetta A. Ionova, Victoria E. Gontcharenko, Konstantin S. Viderskii, Alexei D. Averin, Anton S. Abel, Irina P. Beletskaya

**Affiliations:** 1 Lomonosov Moscow State University, Department of Chemistry, 1-3, Leninskie Gory, Moscow 119991, Russia; vidersky2@gmail.com (K.S.V.); alexaveron@yandex.ru (A.D.A.); beletska@org.chem.msu.ru (I.P.B.); 2 A. V. Topchiev Institute of Petrochemical Synthesis, Russian Academy of Sciences, Leninskiy Pr. 29, Moscow 119991, Russia; 3 P.N. Lebedev Physical Institute of the Russian Academy of Sciences, 53, Leninsky Prospect, Moscow 119991, Russia; victo.goncharenko@gmail.com; 4 Frumkin Institute of Physical Chemistry and Electrochemistry, Russian Academy of Sciences, Leninsky Pr. 31–4, Moscow 119071, Russia

**Keywords:** 1,10-phenanthroline, amination, Pd catalysis, nucleophilic aromatic substitution, diamines

## Abstract

The amination of 2-chloro-1,10-phenanthroline with various alkylamines and diamines was investigated. The Pd-catalyzed reaction and catalyst-free protocols were shown to lead to different products. Catalyst-free amination of this substrate using aliphatic amines yielded secondary phenanthrolin-2-yl amines in 40–80% yields. The Pd-catalyzed reaction afforded tertiary di(phenanthrolin-2-yl)amines even in the presence of excess amine. This allows for the synthesis of unsymmetric *N*,*N*-diheteroaryl-substituted diamines in 44–50% yields with good selectivity. Using 2-bromo-1,10-phenanthroline leads to an increase in their yields (62–91%). We have also demonstrated the possibility to obtain *N*,*N*,*N*′,*N*′-tetraheteroarylated derivatives of diamines in yields of up to 55%. Thus obtained compounds can serve as promising ligands for various applications.

## 1. Introduction

1,10-Phenanthroline is one of the most renowned diimine ligands. Its derivatives find a wide range of applications as extracting agents, ligands in catalytic systems, and building blocks for supramolecular assemblies and functional materials [1,2,3,4,5,6,7,8]. The introduction of substituents of different nature and with different properties in the phenanthroline core opens the way for fine tuning of the properties of ligands and their complexes [2,7,9,10,11]. E.g., the tailoring of the additional chelating site to 1,10-phenanthroline allows for the synthesis of polynuclear coordination compounds with attractive catalytic and photophysical properties [3,12,13,14]. Substituents at positions 2 and 9 can be used to enhance steric hindrances at the coordinating site or to increase the denticity of the ligand with additional donor atoms; the latter is used to create selective extractants of *f*-elements [15,16,17,18,19].

The introduction of nitrogen-containing substituents in the phenanthroline core is of special interest. This enables a broad variation of the photophysical properties of the luminescent complexes of such ligands, e.g., with Ir(III), Ru(II), or Eu(III) [20,21,22,23]. Amino substituents with chelating properties open the way to create fluorescent chemosensors for detecting various analytes and to form supramolecular assemblies of different structures [24,25,26,27,28]. Amino-substituted phenanthrolines can also bind to a number of naturally occurring molecules, including DNA, as they possess versatile biological activity [29,30,31]. The synthesis of 2- and 2,9-diamino-substituted 1,10-phenanthrolines can be achieved via S_N_Ar amination, as α-chloro-1,10-phenanthrolines readily undergo the amination reaction [14,32,33,34,35]. However, commonly, this reaction demands harsh conditions in the autoclave and often requires excess amine, which serves also as a solvent [31,36,37,38]. Notwithstanding high yields, the isolation of the product poses serious problems, as target aminophenanthrolines are usually water-soluble [30]. Catalytic methods like Buchwald–Hartwig amination allows for milder conditions and a stoichiometric amount of amine [39,40,41,42,43], but to the best of our knowledge, this approach has not yet been employed for the synthesis of 2-amino-1,10-phenanthrolines.

**Scheme 1:**
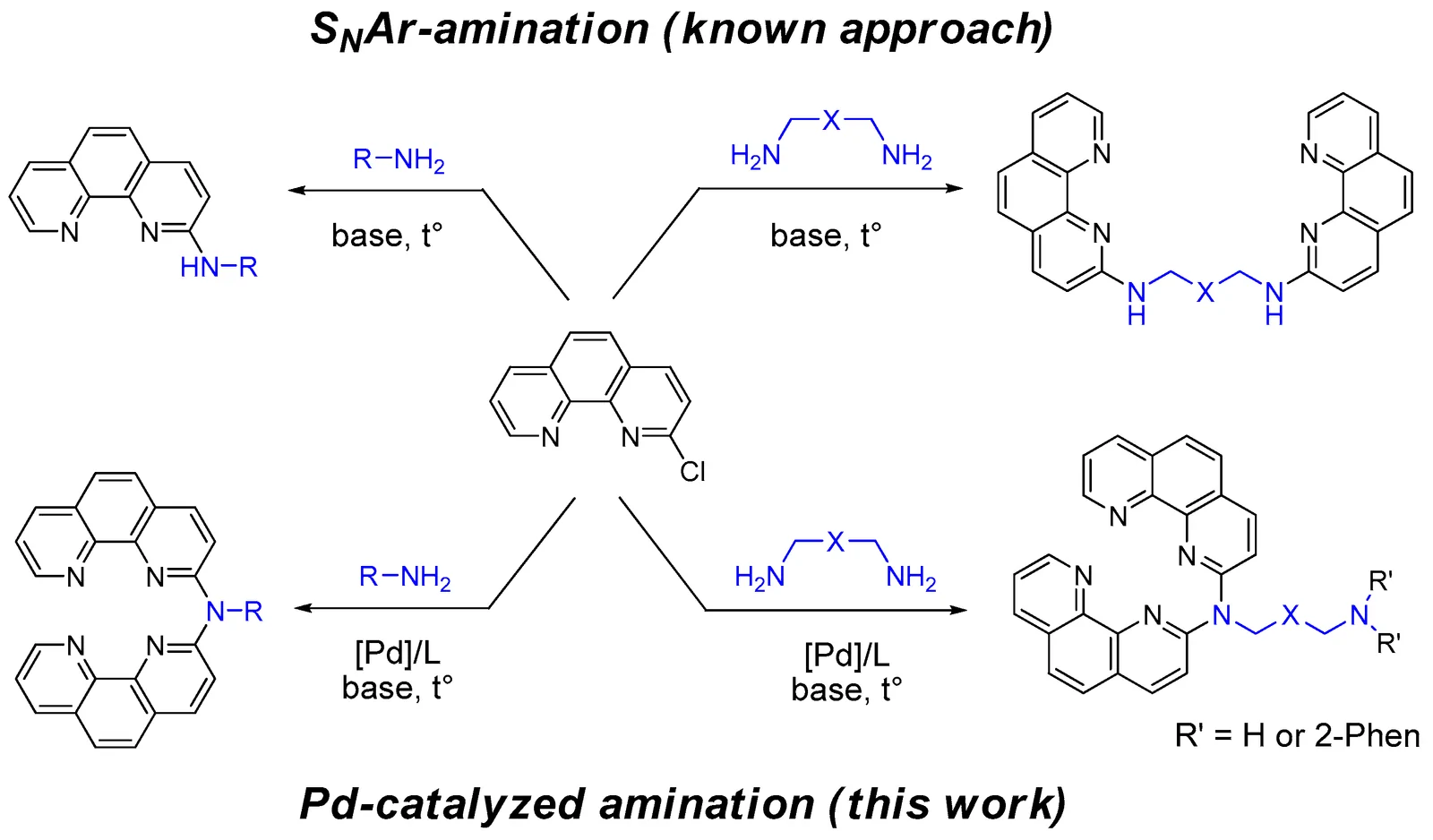
Amination of 2-chloro-1,10-phenanthroline.

In this work, we explore the Pd-catalyzed amination of 2-chloro-1,10-phenanthroline with mono- and diamines for the first time. We have disclosed that the route of the Pd catalysis differs much from that of S_N_Ar reaction, and product yields vary substantially (Scheme 1).

Catalyst-free amination, as expected, leads to phenanthroline-2-yl amines (monoarylation products), while Pd-catalyzed amination with aliphatic amines affords exclusively *N*,*N*-bis(phenanthroline-2-yl)amines, even in the presence of excess amine. This opens perspectives for chemodivergent modifications of di- and polyamines and gives way to new representatives of valuable ligands, i.e., bis(1,10-phenanthroline-2-yl)amines, which are under intense investigation now [44,45,46,47].

## 2. Results and Discussion

### 2.1. Heteroarylation of Benzylamine

**Scheme 2:**
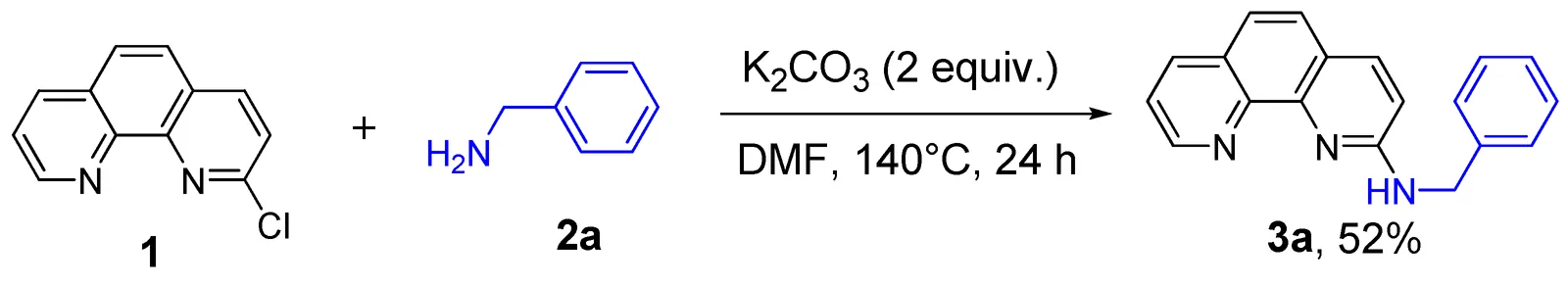
Catalyst-free amination of 2-chloro-1,10-phenanthroline with benzylamine.

The amination of 2-chloro-1,10-phenanthroline (**1**) with benzylamine (**2a**) was studied as a model reaction. Catalyst-free nucleophilic substitution was conducted in DMF (C = 0.5 M) at 140 °C in the presence of K_2_CO_3_ as a base. Full conversion of the starting chloroheteroarene was achieved in 24 h, and the target amination product **3a** was isolated in a 52% yield (Scheme 2).

**Scheme 3:**
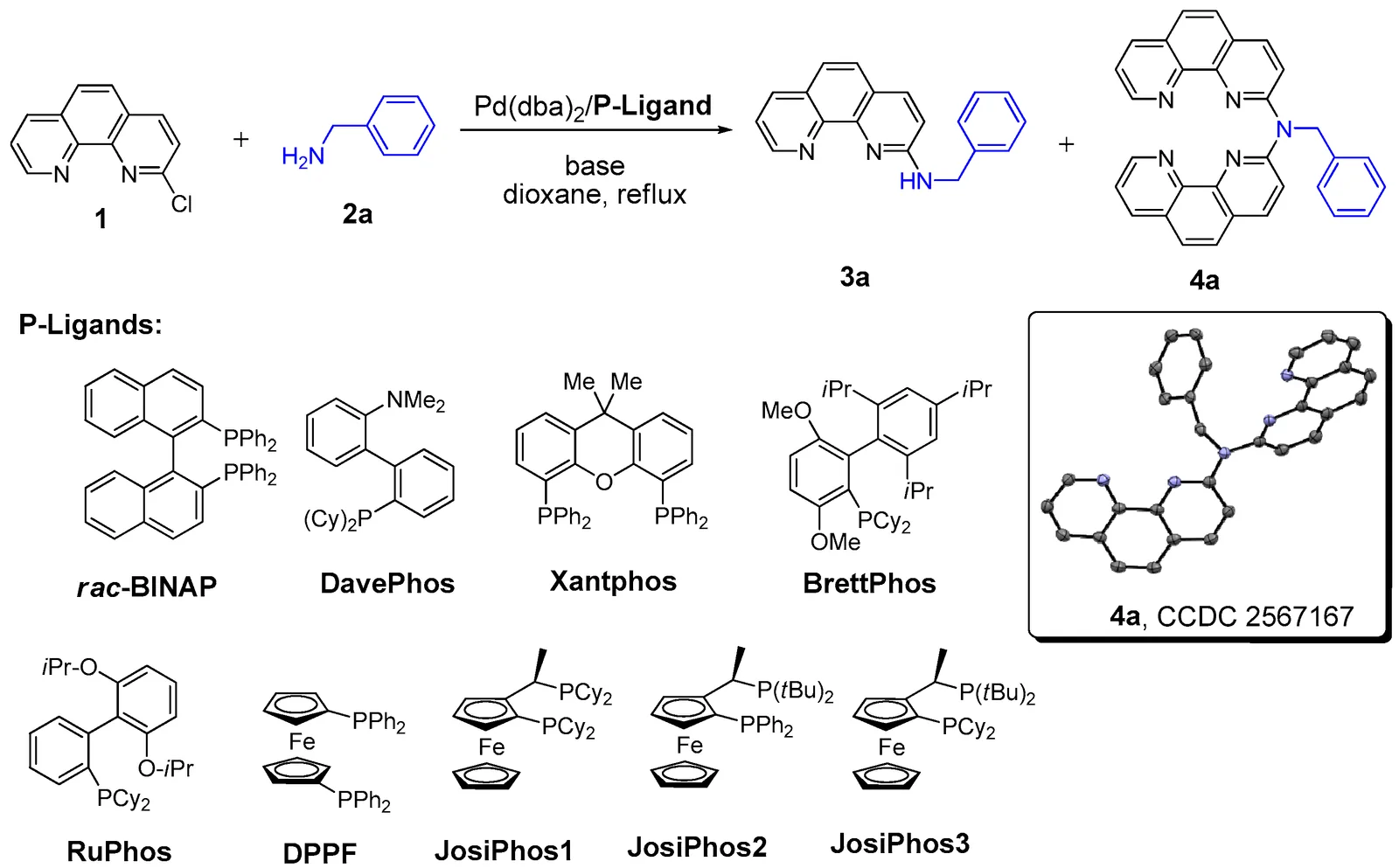
Pd-catalyzed amination of 2-chloro-1,10-phenanthroline with benzylamine. **Inset:** the structure of **4a** obtained by single crystal X-ray diffraction analysis (50% probability ellipsoids).

The Pd-catalyzed amination of 2-chloro-1,10-phenanthroline (**1**) with benzylamine (**2a**) was carried out in boiling dioxane (C = 0.1 M) under argon atmosphere. Various catalytic systems and bases were tested (Scheme 3).

The main results are given in Table 1. One of the most versatile catalytic systems, Pd(dba)_2_/BINAP (4/4.5 mol%) (dba = dibenzylideneacetone, BINAP = *rac*-2,2′-bis(diphenylphosphino)-1,1′-binaphthalene), in the presence of sodium *tert*-butoxide as a base afforded full conversion of the starting chloroheteroarene in 5 h. To our surprise, the sole product was **4a** resulting from the *N*,*N*-diheteroarylation reaction, which was formed nearly in a quantitative yield (Table 1, entry 1). The structure of this compound was verified by X-ray analysis. The application of three equivalents of the amine resulted only in a slight increase in the **3a** yield, the main product still being **4a** (Table 1, entry 2). Only the use of 10 equivalents of **2a** shifted the products ratio in favor of **3a**; however, compound **4a** was also obtained in a 53% yield (Table 1, entry 3). The application of less of the catalyst did not shift the reaction to the monoarylation product **3a** (Table 1, entry 4). The use of weaker bases slowed down the reaction but did not change the ratio of products **3a**/**4a**. NMR monitoring of the reaction showed that monoarylation product **3a** is not accumulated during the reaction, while the amount of diarylation product **4a** steadily increases (Figure S1) [48]. Based on these observations, the rate constant for the formation of **4a** (second stage) can be estimated to be 10 times higher than the rate constant for the formation of product **3a** (first stage) in the presence of the Pd(dba)_2_/BINAP catalytic system.

The nature of the phosphine ligand played a more important role. Thus, running the reaction in the presence of phosphine ligands of the JosiPhos series [49,50] allowed for the preferential formation of product **3a** (Table 1, entries 12–14). However, the chemoselectivity of the reaction decreased due to a side C–O coupling reaction. Running the reaction in the presence of *t*BuONa without a catalyst gave a quantitative yield of the C–O coupling, i.e., 2-*tert*-butoxy-1,10-phenanthroline, demonstrating that the amination proceeds exclusively via catalytic pathway under these conditions. The employment of less nucleophilic base helps to avoid C–O coupling and selectively leads to **3a** (Table 1, entries 15, 16), but the reaction rate diminishes, and one needs 24 h to complete the reaction (Table 1, entries 17, 18). The ratio of the products indicates approximately equal rate constants for the first and second heteroarylations in the presence of the [Pd]/JosiPhos3 catalytic system. The best yield of **3a** (68%) was achieved in the presence of Pd(dba)_2_/JosiPhos3 and an excess of amine (Table 1, entry 19); this result is better than one obtained under catalytic conditions (52%). Nevertheless, this approach only adds to the non-catalytic amination of 2-chloro-1,10-phenanthroline described above.

The preferential formation of **4a** in the course of the Pd-catalyzed amination is of special interest. The formation of side *N*,*N*-diheteroarylated products in the amination of 2-bromopyridine was noted by Buchwald in his pioneer works [51] and also observed by us during the amination of α-halogeno-substituted heterocycles (pyridine, quinoline, pyrimidine) [52,53,54]. However, this process has never been predominant even in the presence of an excess of amine. On the other hand, this reaction gives way to bis(1,10-phenanthroline-2-yl)amine, which is a valuable N4 chelator. This structural moiety is widespread among catalytic systems and complexes [44,45,46,47]. The main known approach to this compound is the non-catalytic heteroarylation of ammonia conducted under harsh conditions in an autoclave [35]. In this connection, we further studied the possibilities of the catalytic and catalyst-free amination of 2-chloro-1,10-phenanthroline to obtain ligands of different structures.

### 2.2. Amination of 2-Chloro-1,10-Phenanthroline with Oxaamines, Diamines and Polyamines

**Figure 1:**
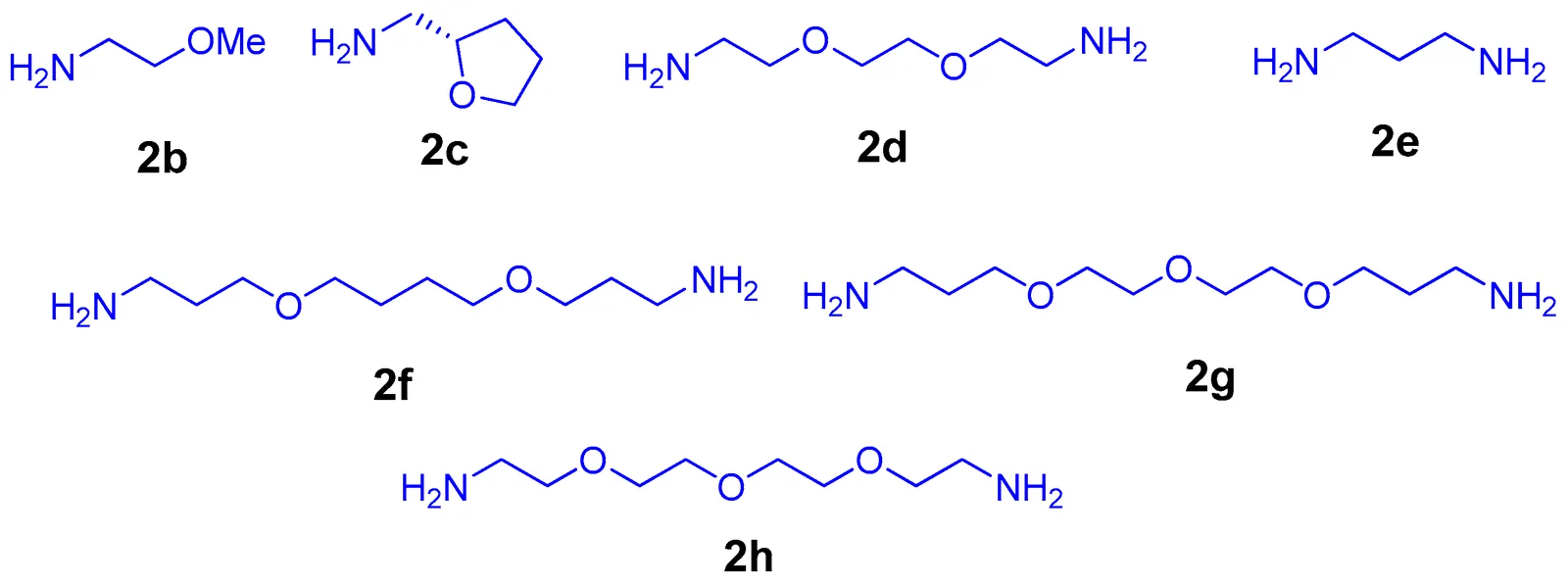
Oxaamines and oxadiamines under study.

In this part of the work, we investigated whether the regularities found with benzylamine are retained in the reactions with other primary amines and diamines (Figure 1).

**Scheme 4:**
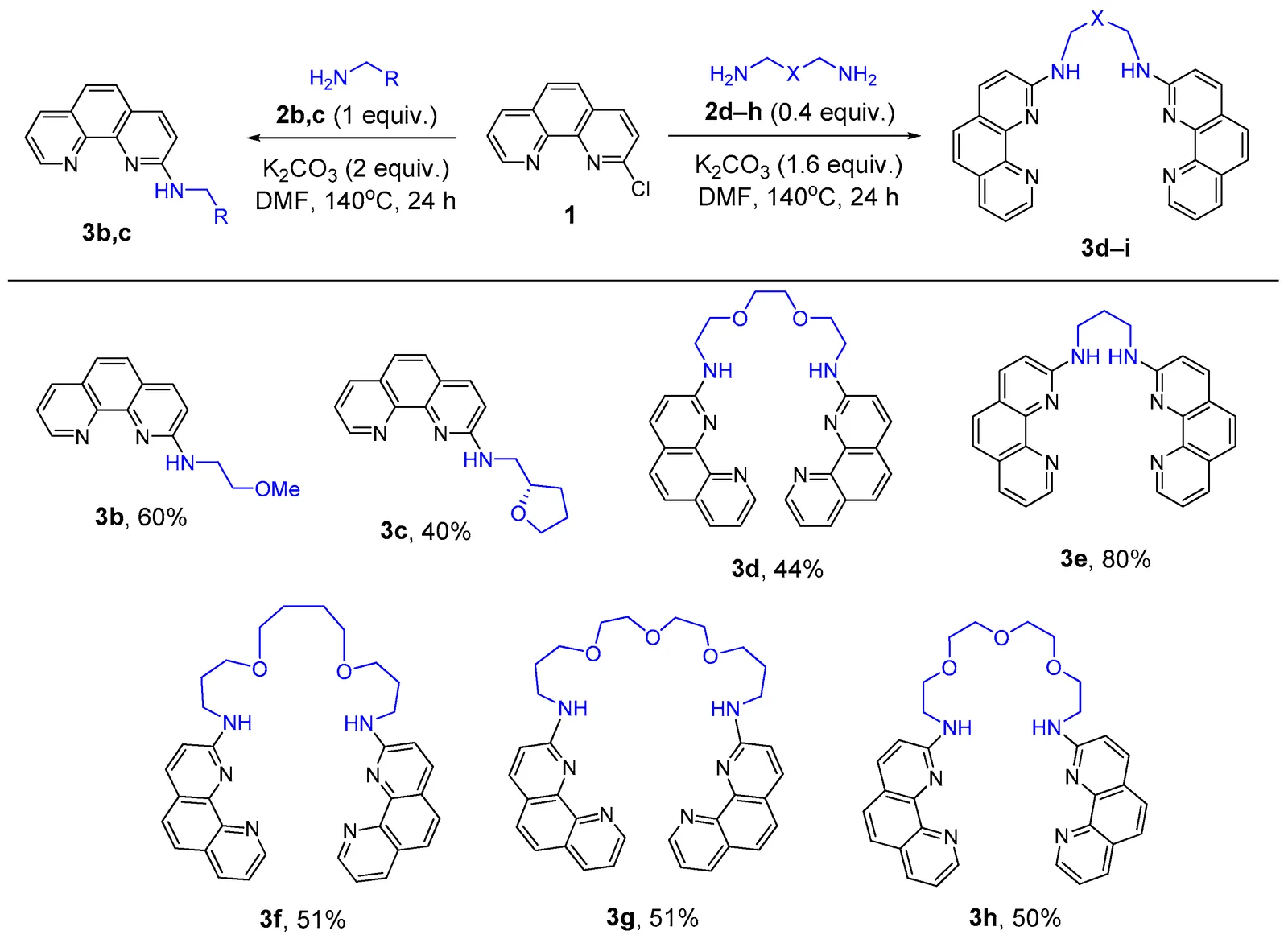
Catalyst-free amination of 2-chloro-1,10-phenanthroline [14].

Catalyst-free amination was conducted in DMF at 140 °C in the presence of K_2_CO_3_ (Scheme 4).

In the case of the diamines **2d**–**h**, 2-chloro-1,10-phenanthroline was taken in a small excess to assure full conversion of the diamines. The products were isolated using column chromatography on silica gel. Like in the case with benzylamine, in the catalyst-free reactions, only monoheteroarylated amines were formed, and compounds **3b**,**c** were obtained in 60% and 40% yields, respectively. As expected, the diamines provided symmetrical *N*,*N*′-disubstituted products **3d**–**h**. This result can be easily explained by the fact that heteroarylation leads to lowering the amino group nucleophilicity and increasing steric hindrances. The retained second amino group becomes more nucleophilic and readily reacts with the second molecule of chloroheteroarene. The selectivity of this reaction is high, and the corresponding compound **3** was isolated in 40–60% yields, while the products with the *N*,*N*-disubstituted amino group were not obtained. However, some side reactions did occur, e.g., the reactions of 2-chloro-1,10-phenanthroline with the products of DMF thermal decomposition at an elevated temperature. Also, the diamino derivatives are polar enough to diminish the preparative yield during chromatography, while the analysis of the reaction mixtures indicates fine selectivity and high yields of the products after the reactions. The less polar compound **3e** was isolated in an 80% yield. Thus, the non-catalytic amination of 2-chloro-1,10-phenanthroline allows for the synthesis of various ligands and podands in good yields, including chiral ones, which are promising for the synthesis of metal complexes and constructing supramolecular systems.

**Scheme 5:**
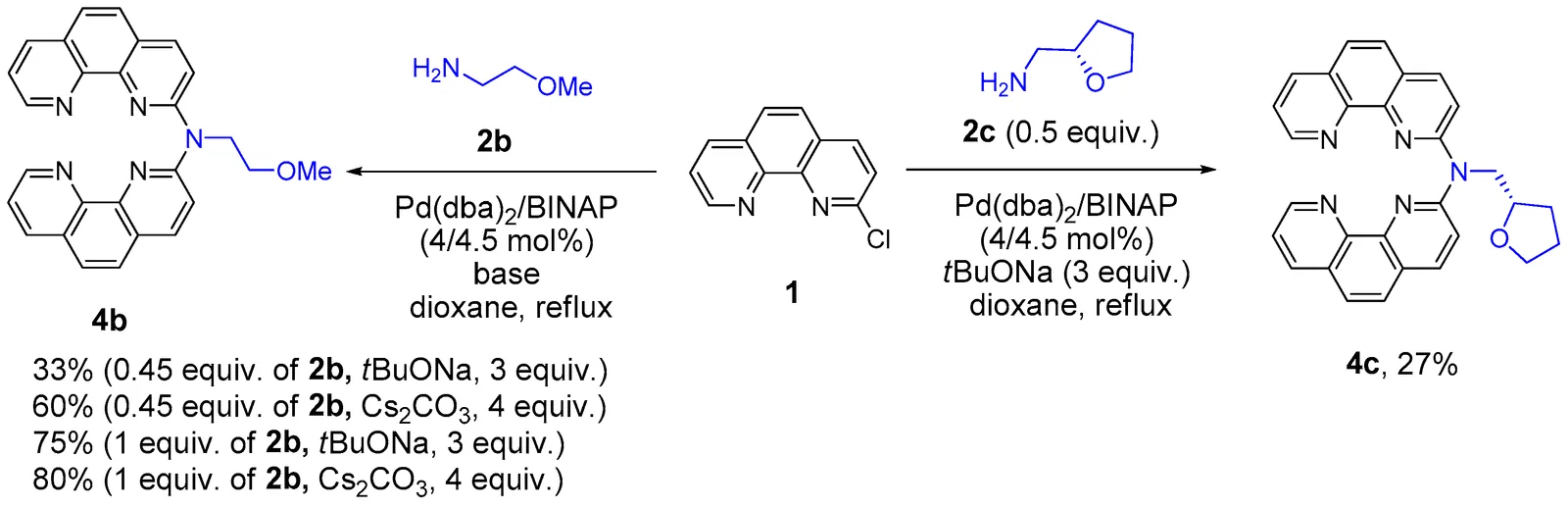
Pd-catalyzed heteroarylation of amines **2b** and **2c** with 2-chloro-1,10-phenanthroline.

The Pd-catalyzed amination of halogeno-substituted heteroarenes is more complicated because both the substrate and the product (such as 1,10-phenanthroline) are good chelators that can bind palladium, which leads to the catalyst deactivation [51]. The chelating nature of the amines can also slow the reaction [55]. This results in a longer reaction time, lower product yield, and side reactions. Thus, the amination of **1** with **2b** in the presence of BINAP ligand proceeds at a slower rate compared to the reaction with **2a**, and takes 20 h (Scheme 5). The only product was **4b**; the monoarylated compound **3b** was not observed in the reaction mixture.

In the presence of sodium *tert*-butylate, the yield of **4b** was only 33% due to the side reactions of **1**. The yield of compound **4c** under analogous conditions was even lower (27%). The application of a softer base Cs_2_CO_3_ increased the yield of **4b** up to 60%. Surprisingly, the change of the reagents ratio (**1**:**2b**) from 2.2:1 to 1:1 raised the yields of **4b** to 75–80% in the presence of both bases. This may be explained by the fact that the formation of monoheteroarylated product **3b** is the rate-limiting step, and this intermediate readily transforms into the final compound **4b**.

**Scheme 6:**
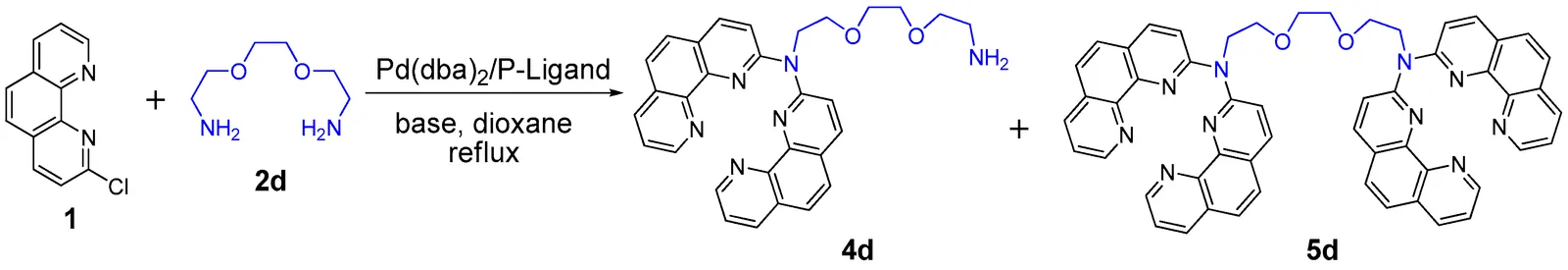
Pd-catalyzed heteroarylation of diamine **2d** with 2-chloro-1,10-phenanthroline.

The reaction with the diamines was studied using **2d** (Scheme 6), and the results are given in Table 2. 

The only products were compounds **4d** and **5d** formed in the course of the *N*,*N*-diheteroarylation process; compound **3d** was not detected. The reactions with the diamines proceeded slower than with monoamines **2a**–**c**. In the presence of the Pd(dba)_2_/BINAP (4/4.5 mol% relative to **2d**) catalytic system and Cs_2_CO_3_, the conversion of **1** (4 equiv.) attained only 30% (Table 2, entry 1). This modest result can be explained by the chelating effect of phenanthroline and dioxadiamine fragments, which is unfavorable for the catalytic performance of Pd. The use of a two-fold amount of the catalyst and a stronger base resulted only in side reactions (Table 2, entry 2). A further increase in the catalyst loading allowed for the synthesis of unsymmetrically substituted **4d** (50%) (Table 2, entry 3). As an alternative to BINAP, another diphosphine ligand, Xantphos ((9,9-dimethyl-9H-xanthene-4,5-diyl)bis(diphenylphosphane)), was tested. This ligand forms more stable complexes with Pd, which is supposed to enhance the stability of the catalytic system in the reaction. Earlier, Xantphos was shown to be efficient in the synthesis of *N*,*N*-diarylamines [56]. In our case, we managed to ensure the full conversion of **1** in the presence of Pd(dba)_2_/Xantphos (8/9 mol%) in 20 h; however, the only reaction result was a complex mixture of different products (Table 2, entry 4). The use of cesium carbonate did not improve the result (Table 2, entry 6), but the application of as much as 16 mol% of the catalyst and a stronger base *t*BuONa allowed for the synthesis of **5d** in 51% yield together with a 35% yield of **4d** (Table 2, entry 5). This fact implies that sodium *tert*-butoxide, though promoting side reactions, can be used for our goal in the presence of enough catalyst to ensure a faster amination reaction.

Further, we aimed at the synthesis of the unsymmetrically substituted compound **4d**. In order to retard the tetraarylation process, cesium carbonate and 8 mol% catalyst were employed (Table 2, entries 7–9). Using one equivalent of 2-chloro-1,10-phenanthroline and running the reaction for 96 h afforded product **4d** in a 49% yield with an 80% conversion of **1** (Table 2, entry 10). A further increase in the yield (90%) was possible when using more active 2-bromo-1,10-phenanthroline, which is less prone to non-catalytic alkoxylation in the presence of *t*BuONa (Table 2, entry 11).

Thus, we found conditions for the synthesis of tetraaryl-substituted diamine **5** and unsymmetric diarylated diamine **4** using Pd catalysis. The preparation of compound **5** demands prolonged time and relatively high catalyst loadings (16 mol%). However, it should be noted that these products cannot be obtained under catalyst-free conditions.

**Scheme 7:**
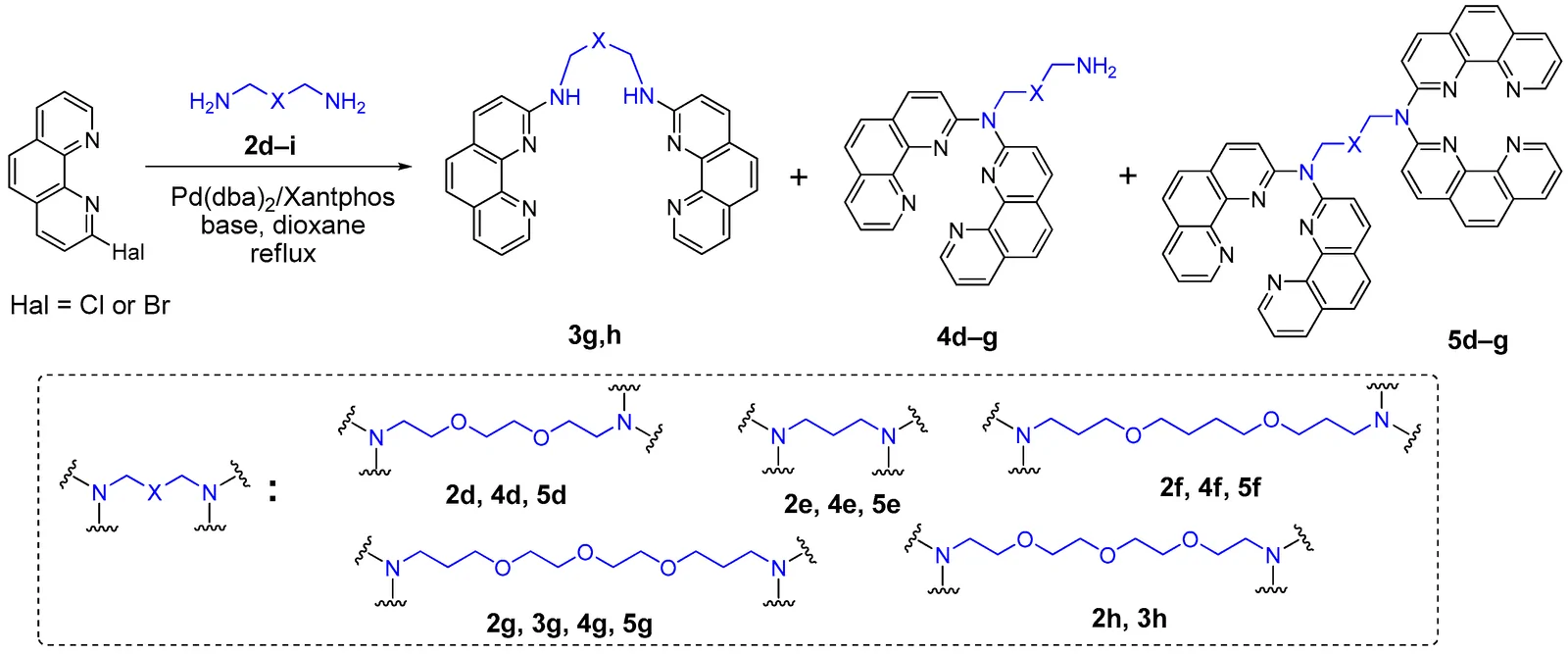
Pd-catalyzed heteroarylation of diamines **2d**–**h** with 2-chloro-1,10-phenanthroline.

Under optimized conditions, we studied the heteroarylation of a series of diamines, **2e**–**h**, differing by the chain length and number of oxygen atoms (Scheme 7).

The results are given in Table 3. 

An excess of **1** in the presence of [Pd]/Xantphos 8/9 mol% (conditions A) provided mixtures of products **4** and **5** (Table 3, entries 1–4). In the case of the diamines **2d**,**e** with shorter chains, compound **5** prevailed (55% and 31% yields, respectively). A longer chain of the diamine leads to an increase in the yield of compound **4** (Table 3, entries 3, 4). Possibly, this is due to the ability of such diamines to better coordinate palladium and hinder the catalytic reaction. Surprisingly, in the reaction with trioxadiamine **2g**, the asymmetrically diarylated product **3g** was also found in the reaction mixture (Table 3, entry 4), which is not typical for this sort of catalytic process. More curious is the fact that the reaction of amine **2h** led only to compound **3h**, while no products of type **4** and **5** were detected. Amine **2h** possesses the strongest chelating ability due to two nitrogen and three oxygen atoms linked by ethylene bridges. Thus, one can conclude that the chelating ability of the substrate and product may seriously influence the formation of the products of the Pd-catalyzed amination of 2-chloro-1,10-phenanthroline.

Introducing one equivalent of chloride **1** (conditions B), we managed to obtain unsymmetrically disubstituted products **4d**–**g** in 44–50% yields (Table 3, entries 6–9). The use of 2-bromo-1,10-phenanthroline helps to increase the selectivity of the reactions, and compounds **4d**–**g** can be obtained in higher yields (62–90%, Table 3, entries 11–14). In the reaction with **2h**, again, the sole symmetrical *N*,*N*′-disubstituted product **3h** was obtained (46%, Table 3, entry 10); this result supports the specific nature of this diamine.

**Scheme 8:**
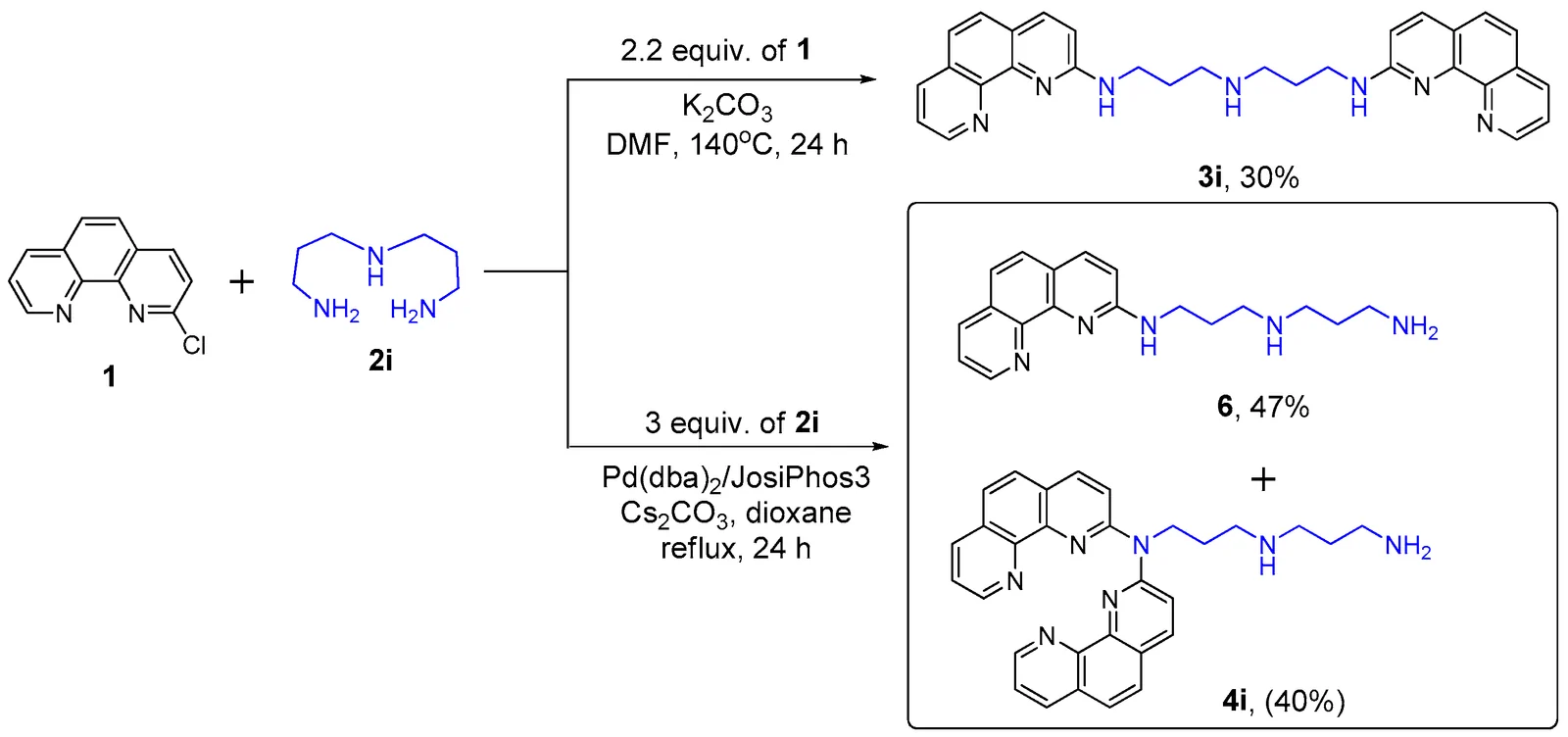
Heteroarylation of triamine **2i** with 2-chloro-1,10-phenanthroline.

Additionally, we studied the modification of triamine **2i** under optimized conditions (Scheme 8). Catalyst-free conditions provided only a mixture of the products as the reaction proceeded non-selectively at the primary and secondary amino groups. However, from this mixture, symmetrically substituted product **3i** was isolated in a 30% yield.

Pd-catalyzed amination is known to proceed selectively at the primary amino groups, whereas secondary amino groups stay mainly intact. This allows for the synthesis of symmetrically arylated polyamines without using protecting groups [57,58]. On the other hand, polyamines are stronger chelators of palladium than polyoxadiamines, and this makes them more challenging substrates [55]. The [Pd]/JosiPhos3 catalytic system able to suppress *N*,*N*-diarylation process helped to obtain monosubstituted triamine **6** in a 47% yield (Scheme 8). Despite excess polyamine, product **4i** was also detected in the reaction mixture, but we were unable to isolate it without admixtures.

Thus, 2-chloro-1,10-phenanthroline reacts according to two pathways in the non-catalytic S_N_Ar amination and Pd-catalyzed amination. In the first case, only compounds with *N*-(1,10-phenanthrolin-2-yl) moieties are formed, and in the second case, only species with *N*,*N*-di(1,10-phenanthrolin-2-yl) fragments are obtained, even in the presence of excess amine. This is most clearly demonstrated by the reactions with the diamines, which form symmetric *N*,*N*′-diheteroarylated products **3** under catalyst-free conditions, while in the presence of the Pd catalyst, they yield either unsymmetric *N*,*N*-diheteroaryl derivatives **4** or symmetric tetrasubstituted compounds **5**. The synthesis of monoheteroaryl derivatives using Pd catalysis is possible only in the case of specific substrates or as a result of fine tuning of the catalytic system.

### 2.3. Insight into Reactivity of 2-Chloroheteroarenes

Further, a series of experiments was conducted to provide a deeper insight into the grounds of the catalytic *N*,*N*-diheteroarylation. The S_N_Ar amination of 2-chloro-1,10-phenanthroline with mono- and diamines proceeds in accordance with the general rules of the aromatic nucleophilic substitution process. On the contrary, the Pd-catalyzed reaction with the same substrate seems to be non-trivial. The formation of *N*,*N*-diheteroaryl derivatives of the primary amino groups was earlier noted as a side process mainly for α-halo-substituted pyridines, quinolines and other six-membered heterocycles [51,52,53,54]. Its suppression demands the use of excess amine and the adjustment of the catalytic system. Often, this side reaction is explained by a higher reactivity of alpha-halogenoheteroarenes, which readily participate in the oxidative addition and increase the reaction rate [51]. However, specific diheteroarylation in the presence of excess amine or in the reactions with diamines leads to the conclusion that the monoheteroarylated amine is more reactive compared to the starting amine. NMR monitoring of the reaction allowed us to estimate the rate constant for the formation of a *N*,*N*-diheteroarylated product to be 10 times higher than the rate constant for the formation of a monoheteroarylated product in the presence of the Pd(dba)_2_/BINAP catalytic system (Figure S1). It is natural to suppose the higher acidity of the monoheteroarylated amino group, which favors the deprotonation–transmetallation step. The intermediate compound easier coordinates to Pd, which also promotes the second heteroarylation reaction.

**Scheme 9:**
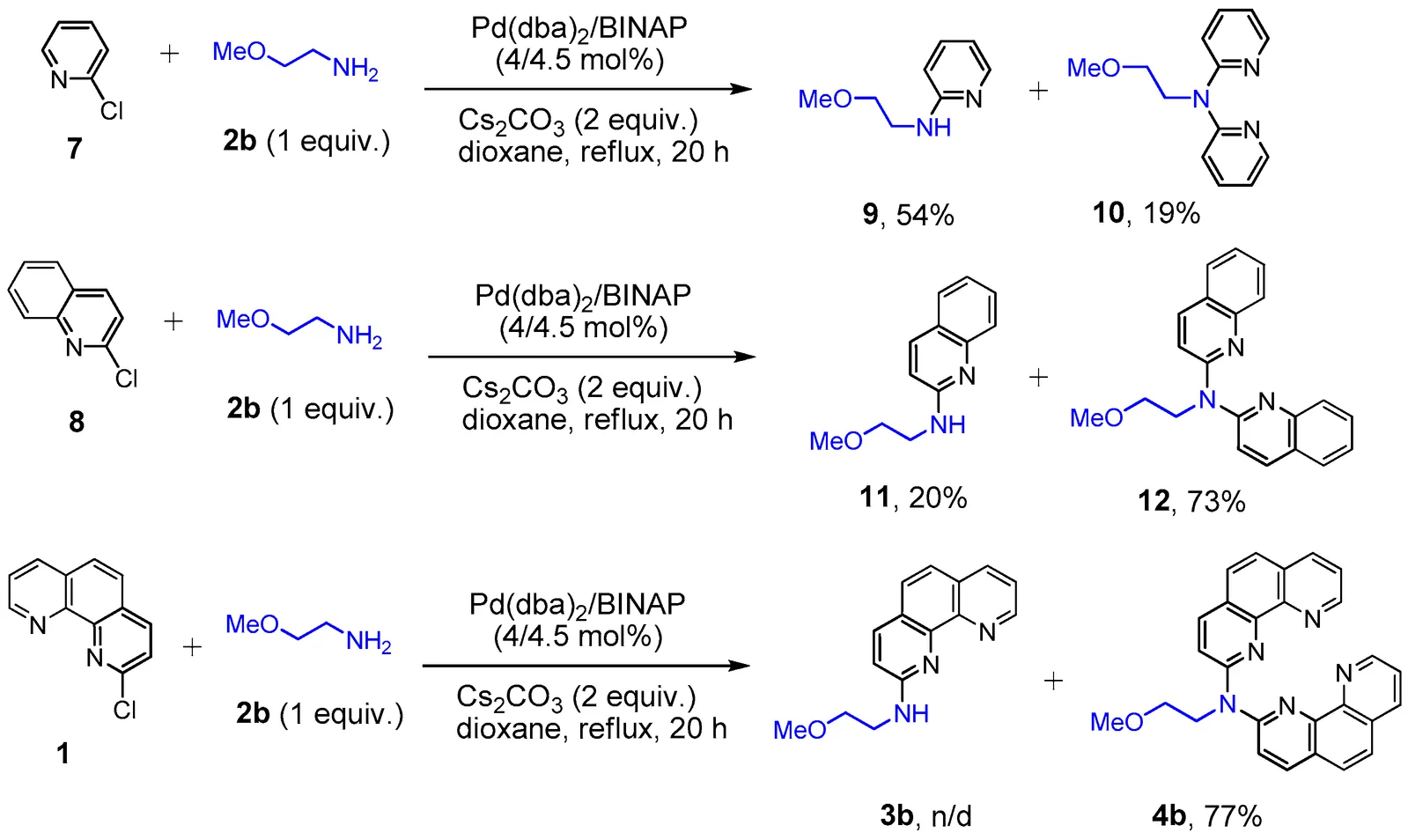
Pd-catalyzed heteroarylation of amine **2b.** The yields were determined by NMR ^1^H analysis of reaction mixtures.

To get more information, we compared the results of the Pd-catalyzed amination of various chloroheteroarenes (2-chloropyridine (**7**), 2-chloroquinoline (**8**), and 2-chloro-1,10-phenantroline (**1**)) using amine **2b** under the same conditions (Scheme 9).

**Scheme 10:**
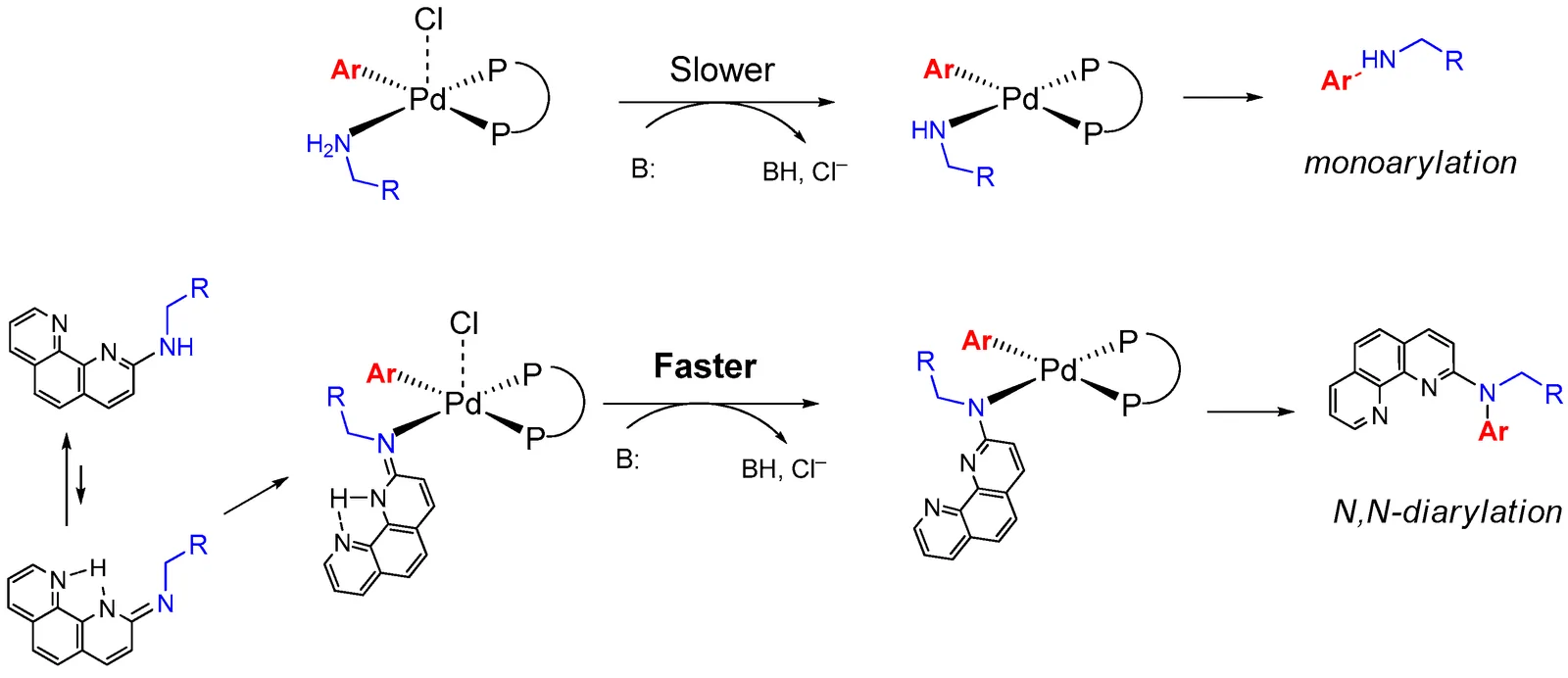
Possible influence of proton mobility on the reaction rate.

In the case of 2-chloropyridine, monoheteroarylated derivative **9** constitutes the main product, with diheteroarylated compound **10** being the by-product. The reaction with 2-chloroquinoline provided diheteroarylated derivative **11** as the main product (73%), while monosubstituted compound **12** was formed in a 20% yield. In the case of 2-chloro-1,10-phenanthroline, the only product was diheteroarylated derivative **4b** (preparative yield 77%), and no monosubstituted compound **3b** was detected. This observation shows a tendency in the series pyridine–quinoline–1,10-phenanthroline to boost the formation of diheteroarylated derivatives. The reason for the fact that quinoline is much more prone to this process compared to pyridine is not yet clear. The coordination properties of its nitrogen atom as well as the acidity of the NH proton in the monoheteroarylated derivative demand a special investigation. In one of our previous works, we have demonstrated that the NH proton in such heterocyclic derivatives is more labile, which leads to a tautomeric equilibrium, which promotes diheteroarylation [54]. In some 2,9-diamino-1,10-phenanthrolines, such tautomerism may occur, but in ours, we did not observe it explicitly [35]. However, taking this into consideration, we may assume that the lability of the proton in 2-amino-1,10-phenanthroline may favor diheteroarylation (Scheme 10).

**Scheme 11:**
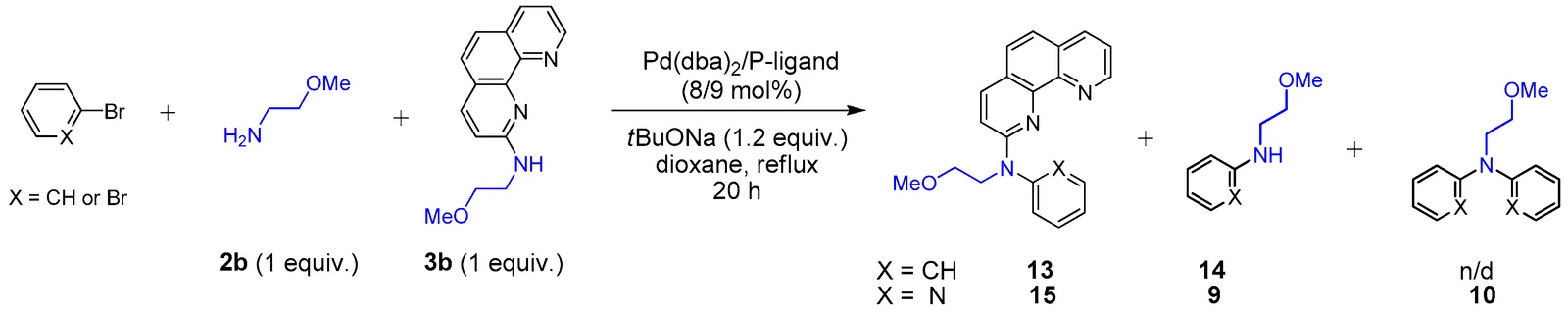
Pd-catalyzed arylation of **2b** in the presence of **3b**.

To verify this hypothesis, we conducted the Pd-catalyzed heteroarylation of amine **2b** in the presence of the equimolar amount of substituted amine **3b** (Scheme 11); the results are given in Table 4.

The reaction of bromobenzene solely with amine **2b** in the presence of [Pd]/BINAP proceeded smoothly, providing nearly a quantitative yield of product **14** (Table 4, entry 1). The additional introduction of one equivalent of compound **3b** fully suppressed the reaction of bromobenzene with **2b**, and only traces of the *N*,*N*-derivative **13** were detected in the mass spectrum (Table 4, entry 2). This is likely due to the coordination of Pd with **3b**, which blocks the catalytic cycle. The reaction of 2-bromopyridine with **2b** in the presence of an equimolar amount of **3b** led to the formation of compounds **15**, **9**, and **10** in a 1:1.3:1 ratio (Table 4, entry 3). The Xantphos ligand promotes *N*,*N*-diarylation, and compound **15** becomes predominant, its yield attaining 58% (Table 4, entry 4). Also, another *N*,*N*-diarylated product **10** was observed (30%), but monoaryl derivative **9** was not found. These facts support an idea that it is the coordination ability of both compounds (heteroarylamine and haloheteroarene) that boosts the diheteroarylation process. Their coordination with palladium somehow favors their participation in the Pd-catalyzed reaction. The influence of the presence of **3b** and **1** on the Pd(dba)_2_–BINAP interaction can be observed by ^31^P NMR spectroscopy and also by the naked eye as a color change and precipitate formation (Figures S2 and S3). It can be hypothesized that after formation, 2-amino-1,10-phenanthroline, via one way or another, more easily returns to the Pd coordination sphere, facilitating its re-participation in the reaction. Apparently, the ability of the 2-haloheterocycle to coordinate to palladium by its nitrogen atom also facilitates its further participation in oxidative addition.

**Scheme 12:**
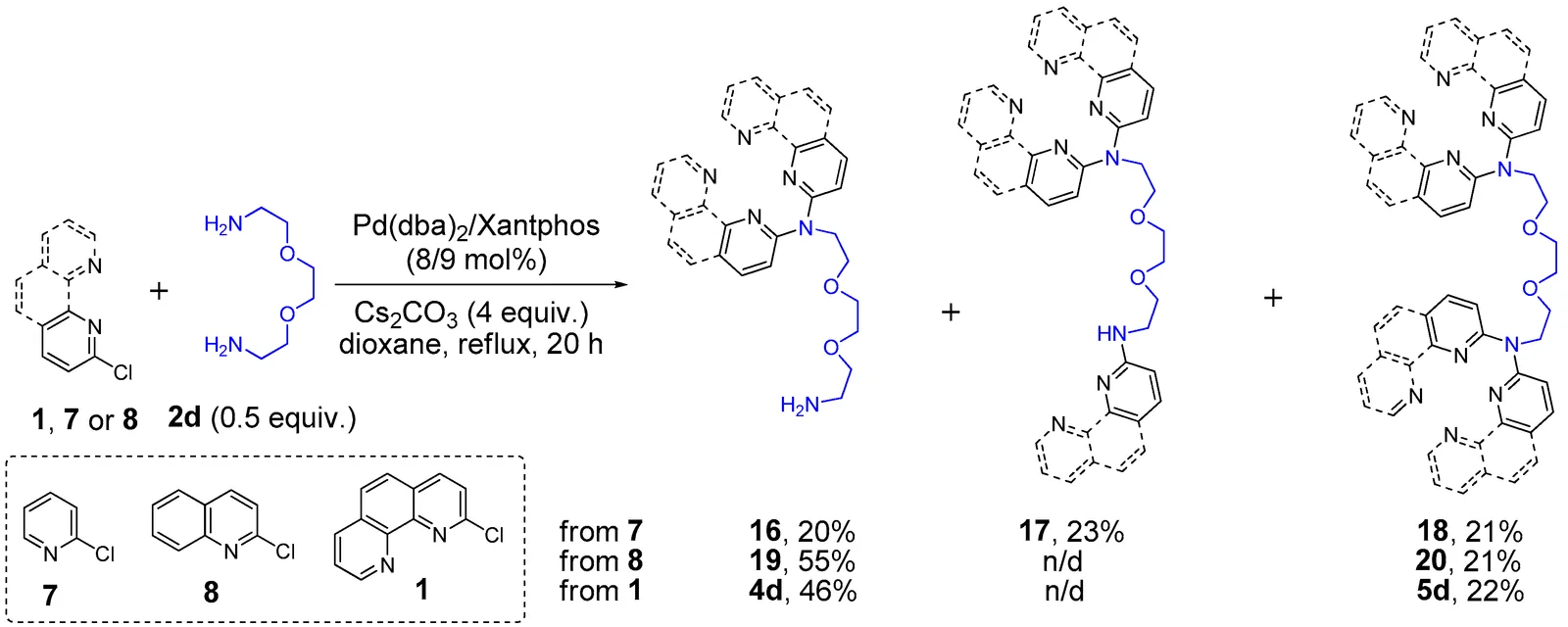
Pd-catalyzed heteroarylation of amine **2b.** Full conversion was observed in the case of **7** and **8**, while the conversion of **1** was 70%. The yields of **4d** and **5d** are based on the conversion of starting compound **1**. The yields and conversions were determined by NMR ^1^H analysis of the reaction mixtures.

Additionally, we studied the heteroarylation of diamine **2d** with the same chloroheteroarenes (two equivalents) under the same conditions ([Pd]/Xantphos, Scheme 12).

No symmetrically *N*,*N*′-disubstituted product was obtained in any reaction. In the case of 2-chloropyridine, we obtained unsymmetrically disubstituted **16**, tetrapyridinyl derivative **18**, and trisubstituted compound **17**. All compounds were isolated in similar yields (20–23%). In the reactions of 2-chloroquinoline and 2-chloro-1,10-phenanthroline, only compounds with *N*,*N*-diheteroarylated moieties were found. Mainly unsymmetrically disubstituted products **19** and **4d** were isolated in 55% and 46% yields, respectively. Secondary products were tetraarylated compounds **20** and **5d** in 21–22% yields. To note, the conversion of 2-chloro-1,10-phenanthroline attained only 70%. The results obtained in these experiments support our supposition that the presence of the chelating moieties in the diamine (two NCCO and one OCCO fragments in **2d**) favor the *N*,*N*-diheteroarylation process.

2-Chloro-1,10-phenanthroline and, moreover, its derivative 2-amino-1,10-phenanthroline are good chelators of palladium, which leads to preferable *N*,*N*-diheteroarylation. This process can be partially suppressed by a carefully selected diphosphine ligand, which forms stable complexes with palladium. E.g., the JosiPhos3 ligand proved to be efficient in the formation of a monophenanthrolinyl derivative. Exclusively, the good chelating properties of the polyoxadiamine (like **2h**) can also prevent the *N*,*N*-diheteroarylation process, possibly to a certain conformation of the intermediate complex, which is unfavorable for *N*,*N*-diheteroarylation.

### 2.4. Synthetic Application of the Ligands

**Scheme 13:**
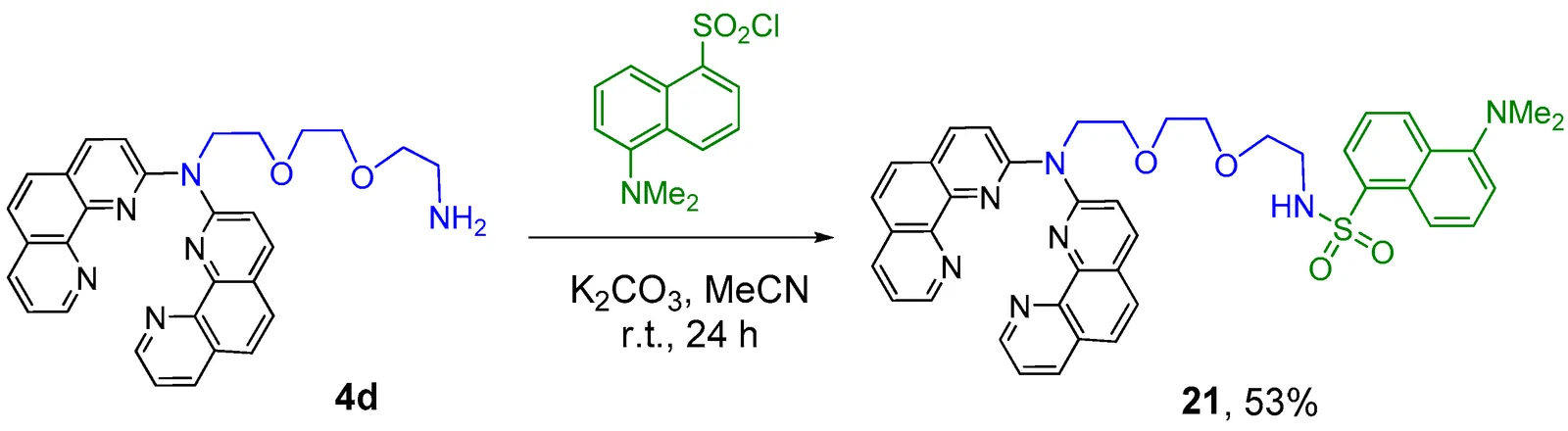
Modification of compound **4d.**

Di(1,10-phenanthrolin-2yl)amines found various applications as transition metal ligands for the design of catalytic systems and components of molecular electronic devices [29,31,44,45,46,47,59,60,61,62]. However, the number of synthetic approaches to such valuable compounds is restricted. Our approach substantially expands the structural diversity of phenanthroline-containing ligands. The presence of the free primary amino groups in compounds of type **4** can be used for further modifications. We demonstrated that **4d** can react with dansyl chloride (Scheme 13), affording luminescent conjugate **21** containing a chelating block and fluorophore. A solution of **21** in acetonitrile absorbs UV–visible light in the range of 300–400 nm and luminesces with green light (λ_max_ = 525 nm, Φ = 0.1).

**Scheme 14:**
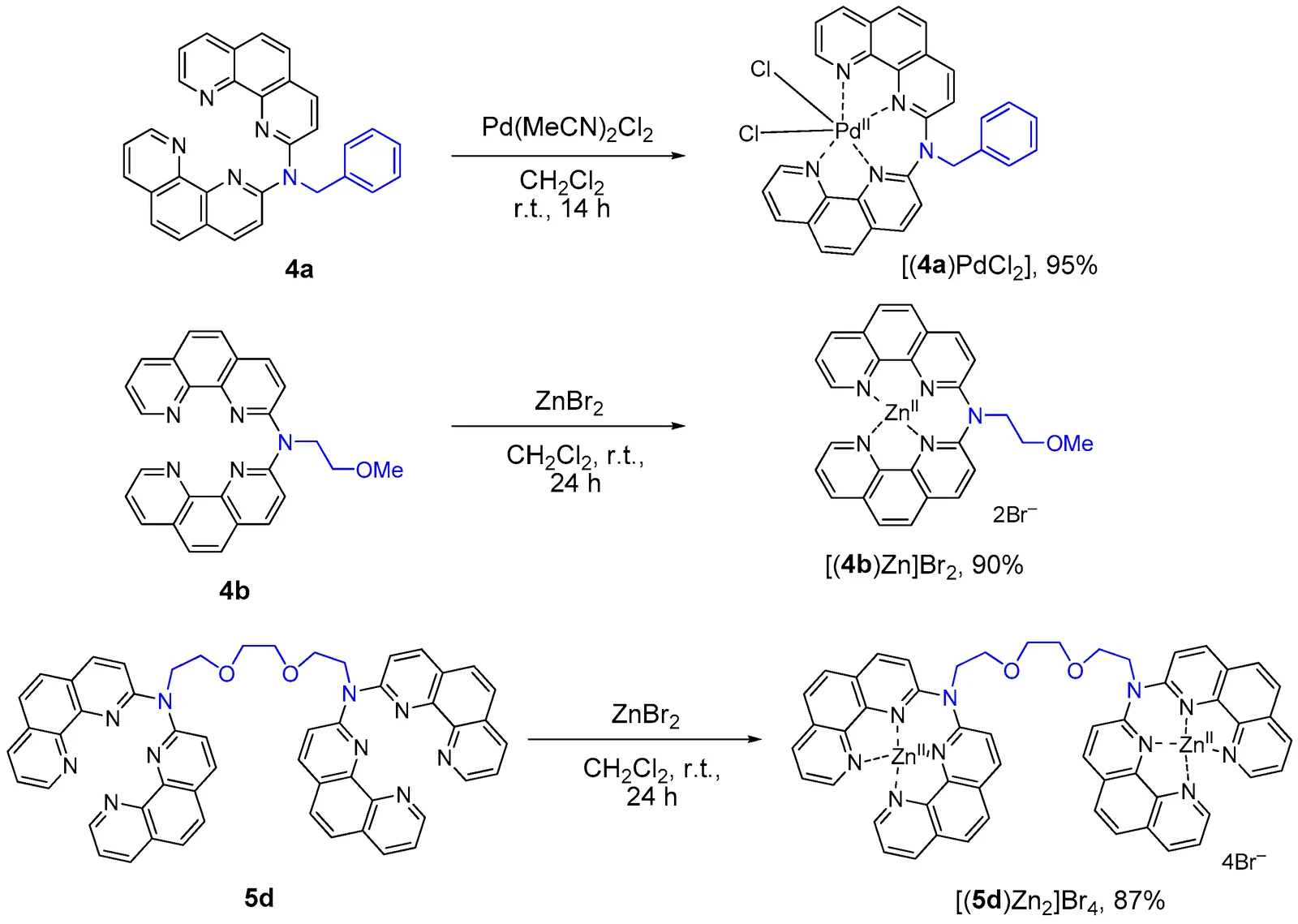
Synthesis of the complexes from ligands **4a**, **4b** and **5d**.

The obtained bis(1,10-phenanthrolin-2-yl)amines can be used for the synthesis of complexes with various transition metals. To demonstrate this possibility, compounds **4a**,**b** were used to prepare Pd and Zn complexes (Scheme 14). They were obtained in dichloromethane in high yields from corresponding metal precursors, and their structures were preliminary verified using NMR spectroscopy and mass spectrometry.

Dinuclear complexes also can be obtained; by the reaction of **5d** with two equivalents of ZnBr_2_, we synthesized [(**5d**)Zn_2_]Br_4_ in a high yield. Further in-depth studies will be conducted, aimed at investigating the coordination chemistry of the obtained ligands and the application of their complexes in supramolecular chemistry and catalysis.

## 3. Materials and Methods

### 3.1. Reagents and Solvents

Unless otherwise noted, all chemicals and starting materials were obtained commercially from ABCR (Karlsruhe, Germany), Alfa-Aesar (Thermo Fisher Scientific Co., Ward Hill, MA, USA) or Sigma-Aldrich (Merck Co., Darmstadt, Germany) and used without further purification. 2-Chloro-1,10-phenanthroline was prepared from 1,10-phenanthroline using the reported three-step method [63]. 2-Bromo-1,10-phenanthroline was obtained by the same method [64]. Pd(dba)_2_ was synthesized according to a known procedure [65] and used without recrystallization. Dioxane was distilled successively over NaOH and sodium under argon; CH_2_Cl_2_ was distilled over CaH_2_; MeOH was used freshly distilled. DMF was distilled over CaH_2_ under reduced pressure and stored over 3Å sieves. Preparative column chromatography was carried out using Silica gel 60 (40–63 µm) from Merck (Darmstadt, Germany). Pd-catalyzed reactions were performed under an argon atmosphere.

### 3.2. Apparatus

The ^1^H and ^13^C spectra were recorded using a Bruker Avance-400 spectrometer (Bruker Daltonics, Bremen, Germany) in chloroform-*d* or DMSO*-d6*. The residual proton signals of the solvent were used as internal standards. Methanol-*d4* was added in some cases to increase the solubility of the compounds. MALDI TOF mass spectra were obtained with Bruker Daltonics Autoflex II mass spectrometer (Bruker Daltonics, Bremen, Germany) in positive ion mode with a dithranol matrix and polyethyleneglycols as the internal standards. ESI mass measurements were obtained with a Thermo Scientific Orbitrap Elite high-field hybrid mass spectrometer (Thermo Fisher Scientific, Waltham, MA, USA). FTIR spectra were registered on Nicolet iS 5 with iD3 ATR accessory (ZnSe) (Thermo Fisher Scientific, Madison, WI, USA). Single-crystal X-ray diffraction analysis of **4a** was performed using Bruker D8 Quest diffractometer (Bruker Daltonics, Bremen, Germany). UV–vis spectra were registered with a Hitachi U-2900 device (Tokyo, Japan) in a quartz cuvette (Hellma, l = 1 cm). Fluorescence spectra were registered with a Horiba Jobin Yvon Fluoromax-2 apparatus (Edison, NJ, USA) in a quartz cuvette (Hellma, l = 1 cm). Luminescence quantum yield of compound **21** was determined in MeCN (HPLC garde) relative to quinine sulfate solution in 0.05 H_2_SO_4_ (Φ = 0.53) according to a standard procedure [66].

### 3.3. S_N_Ar Amination of 2-Chloro-1,10-Phenanthroline

*General procedure.* A glass vial (8 mL) equipped with a magnetic stirrer was charged with 2-chloro-1,10-phenanthroline **1** (0.5–1.25 mmol), corresponding amine **2a**–**i**, anhydrous K_2_CO_3_ (2 equiv.) and DMF (1 mL). The reaction mixture was stirred at 140 °C for 24 h. After cooling to room temperature, the reaction mixture was diluted with CH_2_Cl_2_ (ca. 10 mL) and an inorganic precipitate was filtered off and additionally washed with dichloromethane (5 mL). The combined organic filtrates were concentrated under reduced pressure, and the residue was analyzed by NMR spectroscopy. The products were purified using a chromatography column on silica gel using CH_2_Cl_2_/MeOH and CH_2_Cl_2_/MeOH/NH_3_(aq.) mixtures as eluents (gradual elution using pure CH_2_Cl_2_, CH_2_Cl_2_/MeOH, 100:1 to 5:1 *v*/*v* and CH_2_Cl_2_/MeOH/NH_3_(aq.) 100:20:1 to 10:4:1 *v*/*v*/*v*). The products were obtained as yellow oils. *Note:* The amount of DMF used in the reaction was such that the concentration of the reagent used in the deficiency was 0.5 M. Compounds **3a**–**h** were prepared using this procedure. Products **3b** and **3d**–**h** were described in our recent work [14]. Experimental details, characterization data and NMR spectra of the products are given in the Supplementary Materials.

### 3.4. Pd-Catalyzed Amination of 2-Halogenoheteroarenes

*General procedure.* A two-neck flask equipped with a condenser and a magnetic stirrer was charged with corresponding halogeno-substituted heteroarene (0.2–1.1 mmol), Pd(dba)_2_ (2–16 mol%), and phosphine ligand (2.5–18 mol%) and flushed with dry argon. Then, absolute dioxane was added and the mixture was stirred for 2–3 min. Then, corresponding amine **2** (0.2–2 mmol) and a base were added, and the reaction mixture was refluxed for 5–96 h. After cooling it down to ambient temperature, the reaction mixture was diluted with CH_2_Cl_2_, the solution was filtered and evaporated in vacuo, and the residue was analyzed by NMR spectroscopy. The products were purified using a chromatography column on silica gel using CH_2_Cl_2_/MeOH and CH_2_Cl_2_/MeOH/NH_3_(aq.) mixtures as eluents (gradual elution using CH_2_Cl_2_, CH_2_Cl_2_/MeOH, 100:1 to 5:1 *v*/*v*, CH_2_Cl_2_/MeOH/NH_3_(aq.) 100:20:1 to 100:20:3 *v*/*v*/*v*). The products were obtained as colorless or pale-yellow oils. *Note:* The catalyst molar loading is given relative to the reagent used in the deficiency. The amount of dioxane added to the reaction was such that the concentration of the reagent used in the deficiency was 0.1 M.

Compounds **9** [67], **10** [12], **11** [68], **14** and **16** [69] are known and their NMR spectra are in agreement with those reported in the literature. The yields of products **9**–**12** and **16**–**20** were determined from ^1^H NMR spectra of the reaction mixtures. These compounds were prepared and characterized by direct synthesis to confirm their correct identification. Compounds **9**, **10**, **16**, **17**, and **18** were prepared from 2-bromopyridine according to the general procedure. Compound **11** was prepared from 2-chloroquinoline using the above-described catalyst-free amination procedure in DMF.

The experimental details, characterization data and NMR spectra are given in the Supplementary Materials.

### 3.5. Synthetic Application of the Ligands

The synthetic procedures for obtaining compound **21** and complexes [(**4a**)PdCl_2_], [(**4b**)Zn]Br_2_ and [(**5d**)Zn_2_]Br_4_; characterization data; and NMR spectra of the products and mass spectra of the complexes are given in the Supplementary Materials.

## 4. Conclusions

To conclude, we have established that 2-chloro-1,10-phenanthroline under the conditions of Pd-catalyzed amination using primary aliphatic amines is prone to form the products of *N*,*N*-diheteroarylation. A similar process was noted earlier as a side reaction with α-substituted six-membered azaheterocycles, but with 2-halogeno-substituted 1,10-phenanthrolines, it becomes predominant and occurs even with excess amine. This fact can be explained by a higher reactivity of the intermediate monoarylated amine, which is due to a better coordination ability of both the starting halide and this monoarylated amine with palladium. This observation allowed us to propose two chemodivergent methods of diamine heteroarylation using Pd catalysis and catalyst-free aromatic nucleophilic substitution. The S_N_Ar reaction leads to symmetric *N*,*N*′-diheteroarylated diamines, while the Pd-catalyzed process yields unsymmetric *N*,*N*-diheteroaryl derivatives. The application of 2-bromo-1,10-phenanthroline in this reaction allows for an additional increase in the selectivity of the process and the yields of the products. This reaction can be equally used for the preparation of *N*,*N*,*N*′,*N*′-tetraheteroarylated amines. In addition, we proposed a specific catalytic system to obtain *N*-(1,10-phenanthrolin-2-yl)-substituted amines using Pd catalysis. The obtained di(phenanthrolin-2-yl)amines can be regarded as valuable chelators in the synthesis of complexes with transition metals for diverse purposes.

## Figures and Tables

**Table 1:** Pd-catalyzed amination of 2-chloro-1,10-phenanthroline with benzylamine.

Entry ^1^	2a (Equiv.)	P-Ligand	Base (Equiv.)	Conversion of 1 (%)	Yield (%) ^2^	
					3a	4a
1	1	BINAP	*t*BuONa (1.5)	>95	2	98 ^3^
2	3	BINAP	*t*BuONa (1.5)	>95	11	89
3	10	BINAP	*t*BuONa (1.5)	>95	47	53
4 ^4^	3	BINAP	*t*BuONa (1.5)	>95	8	92
5	3	BINAP	Cs_2_CO_3_ (2)	>95	3	97
6	3	BINAP	K_3_PO_4_ (2)	>95	3	97
7	3	DavePhos	*t*BuONa (1.5)	>95	5	95
8	3	Xantphos	*t*BuONa (1.5)	>95	17	83
9	3	RuPhos	*t*BuONa (1.5)	>95	10	90
10	3	BrettPhos	*t*BuONa (1.5)	>95	8	92
11	3	DPPF	*t*BuONa (1.5)	>95	18	82
12	3	JosiPhos1	*t*BuONa (1.5)	>95	40	51
13	3	JosiPhos2	*t*BuONa (1.5)	>95	27	59
14	3	JosiPhos3	*t*BuONa (1.5)	>95	38	38
15	3	JosiPhos3	Cs_2_CO_3_ (2)	13	13	0
16	3	JosiPhos3	K_3_PO_4_ (2)	19	19	0
17 ^5^	3	JosiPhos3	Cs_2_CO_3_ (2)	>95	63	37
18 ^5^	3	JosiPhos3	K_3_PO_4_ (2)	>95	56	44
19 ^5^	5	JosiPhos3	Cs_2_CO_3_ (2)	>95	68 ^3^	32

^1^ **Reaction conditions**: 2-chloro-1,10-phenanthroline (0.2 mmol), **2a** (0.2–2 mmol), Pd(dba)_2_ (0.008 mmol), P-ligand (0.009 mmol), base, dioxane (2 mL), reflux under argon atmosphere, 5 h. ^2^ The yield and conversion were determined by NMR ^1^H analysis of reaction mixtures. ^3^ Preparative yield. ^4^ Pd(dba)_2_ (0.004 mmol) and BINAP (0.0045 mmol) were used. ^5^ The reaction time was 24 h.

**Table 2:** Pd-catalyzed heteroarylation of diamine **2d**.

Entry ^1^	1 (Equiv.)	P-Ligand	[Pd]/P-Ligand (mol%)	Base (Equiv.)	Time (h)	Conversion of 1 (%)	Yield (%) ^2^	
							4d	5d
1	4.4	BINAP	4/4.5	Cs_2_CO_3_ (8)	20	30	4	18
2	4.4	BINAP	8/9	*t*BuONa (6)	20	66	n/d	n/d
3	4.4	BINAP	16/18	*t*BuONa (6)	20	75	50	n/d
4	4.4	Xantphos	8/9	*t*BuONa (6)	20	>95	– ^3^	– ^3^
5	4.4	Xantphos	16/18	*t*BuONa (8)	20	>95	35	51
6	4.4	Xantphos	8/9	Cs_2_CO_3_ (8)	20	>95	14	5
7	3	Xantphos	8/9	Cs_2_CO_3_ (6)	20	>95	41	8
8	2	Xantphos	8/9	Cs_2_CO_3_ (2)	20	70	32	15
9	1	Xantphos	8/9	Cs_2_CO_3_ (2)	20	75	34	<5
10	1	Xantphos	8/9	Cs_2_CO_3_ (2)	96	80	49	<5
11 ^4^	0.85 ^4^	Xantphos	8/9	*t*BuONa (1.5)	96	>95	90	traces

^1^ **Reaction conditions A**: 2-chloro-1,10-phenanthroline (0.25–1 mmol), amine (0.25 mmol), Pd(dba)_2_, P-ligand, base, dioxane (2.5 mL), reflux under argon atmosphere. ^2^ Preparative yields. ^3^ Complex mixture of products. ^4^ 2-Bromo-1,10-phenanthroline was used instead of **1**.

**Table 3:** Pd-catalyzed amination of 2-chloro-1,10-phenanthroline with diamines.

Entry	Amine	Conditions	Product (Yield, %) ^1^		
			3	4	5
1	**2d**	**A ^2^**	n/d	**4d** (35)	**5d** (55)
2	**2e**	**A ^2^**	n/d	**4e** (17)	**5e** (31)
3	**2f**	**A ^2^**	n/d	**4f** (41)	**5f** (22)
4	**2g**	**A ^2^**	**3g** (16)	**4g** (30)	**5g** (10)
5	**2h**	**A ^2^**	**3h** (34)	n/d	n/d
6	**2d**	**B ^3^**	n/d	**4d** (49)	**5d** (traces)
7	**2e**	**B ^3^**	n/d	**4e** (48)	**5e** (12)
8	**2f**	**B ^3^**	n/d	**4f** (44)	**5f** (traces)
9	**2g**	**B ^3^**	n/d	**4g** (50)	**5g** (17)
10	**2h**	**B ^3^**	**3h** (46)	n/d	n/d
11	**2d**	**C ^4^**	n/d	**4d** (90)	**5d** (traces)
12	**2e**	**C ^4^**	n/d	**4e** (91)	**5e** (traces)
13	**2f**	**C ^4^**	n/d	**4f** (87)	**5f** (traces)
14	**2g**	**C ^4^**	n/d	**4g** (62)	**5g** (traces)

^1^ Preparative yields. ^2^ **Reaction conditions A**: 2-chloro-1,10-phenanthroline (1.1 mmol), amine (0.25 mmol), Pd(dba)_2_ (0.04 mmol), Xantphos (0.045 mmol), *t*BuONa (1.5 mmol), dioxane (2.5 mL), reflux under argon atmosphere, 20 h. ^3^ **Reaction conditions B**: 2-chloro-1,10-phenanthroline (0.25 mmol), amine (0.25 mmol), Pd(dba)_2_ (0.02 mmol), Xantphos (0.023 mmol), Cs_2_CO_3_ (1 mmol), dioxane (2.5 mL), reflux under argon atmosphere, 96 h. ^4^ **Reaction conditions C**: 2-bromo-1,10-phenanthroline (0.25 mmol), amine (0.3 mmol), Pd(dba)_2_ (0.02 mmol), Xantphos (0.023 mmol), Cs_2_CO_3_ (1 mmol), dioxane (2.5 mL), reflux under argon atmosphere, 96 h.

**Table 4:** Pd-catalyzed arylation of **2b** in the presence of **3b**.

Entry ^1^	Substrate	P-Ligand	Product (Yield, %) ^2^
1 ^3^	bromobenzene	BINAP	**14** (>95%)
2	bromobenzene	BINAP	**13** (traces), no conversion ^4^
3	2-bromopyridine	BINAP	**15** (24), **9** (30), **10** (25)
4	2-bromopyridine	Xantphos	**15** (58), **10** (30)

^1^ **Reaction conditions**: bromoarene (0.25 mmol), **2b** (0.25 mmol), **3b** (0.25 mmol), Pd(dba)_2_ (0.02 mmol), P-ligand (0.02 mmol), *t*BuONa (0.3 mmol), dioxane (2.5 mL), reflux under argon atmosphere, 20 h. ^2^ The yields were determined by NMR. ^3^ **3b** was not added. ^4^ Detected by MS (MALDI TOF).

## Data Availability

The data presented in this work are available in this article and the Supplementary Materials.

## Supplementary Materials

The following supporting information can be downloaded at https://www.mdpi.com/article/10.3390/molecules31183344/s1: Synthetic procedures and characterization data for the compounds **3a**–**i**, **4a**–**g,i**, **5d**–**g**, **6**, **9**–**12**, and **16**–**21**, and complexes [(**4a**)PdCl_2_], [(**4b**)Zn]Br_2_ and [(**5d**)Zn_2_]Br_4_; Figure S1: NMR monitoring of the reaction of **1** and **2a**; Figures S2 and S3: ^31^P NMR spectra of BINAP and the Pd(dba)_2_/BINAP, Pd(dba)_2_/BINAP/**1** and Pd(dba)_2_/BINAP/**3b** stoichiometric mixtures in dioxane; Figure S4: Absorption and emission spectra of **21** in MeCN; Table S1: Crystal data and refinement parameters for **4a** [70,71,72]; Figures S5–S71: ^1^H and ^13^C NMR spectra of the new compounds; Figures S72–S74: mass spectra of the complexes; the structure of **4a** obtained by single crystal X-ray diffraction analysis as CIF-file.
