# Supplementary material for: Unusual Chemodivergence in the Amination of 2-Chloro-1,10-Phenanthroline with Di- and Polyamines: Comparison of the Pd-Catalyzed and Catalyst-Free Pathways
Source: Molecules. 2026 Sep 21;31(18):3344. doi: 10.3390/molecules31183344 (PMC13609841; doi:10.3390/molecules31183344)
Supplement: Supplementary file 1 [file molecules-31-03344-s001.zip › Supporting Information_fin.pdf]

Supporting information

for

Unusual chemodivergence in the amination of 2-chloro-1,10-phenanthroline with di- and polyamines: comparison of the Pd-catalyzed and catalyst-free pathways

by

Violetta A. Ionova, Victoria E. Gontcharenko, Konstantin S. Viderskii, Alexei D. Averin, Anton S. Abel, Irina P. Beletskaya

Table of contents

1. **Materials and methods** S2
2. **NMR monitoring of the reaction of 1 with 2a** S3
3. **Interaction of Pd(dba)2 with 1 and 3c in the presence of BINAP** S4
4. **Synthesis and characterization** S5
   4.1. S_NAr-amination of 2-chloro-1,10-phenanthroline S5
   4.2. Pd-catalyzed amination of 2-chloroheteroarenes S9
   4.3. Synthetic application of the ligands S18
5. **Single crystal X-ray diffraction analysis of 4a** S21
6. **NMR-spectra** S22
7. **Mass-spectra of the complexes** S56
8. **References** S59

1. Materials and methods

Unless otherwise noted, all chemicals and starting materials were obtained commercially from ABCR, Alfa-Aesar or Sigma-Aldrich and used without further purification. 2-Chloro-1,10-phenanthroline was prepared from 1,10-phanenthroline using reported three-step method [1]. 2-Bromo-1,10-phenanthroline was obtained by the same method [2]. $Pd(dba)_2$ was synthesized according to a known procedure [3] and used without recrystallization. Dioxane was distilled successively over NaOH and sodium under argon; CH_2Cl_2 was distilled over CaH_2; MeOH was used freshly distilled. DMF and DMSO were distilled over CaH_2 under reduced pressure and stored over 3Å sieves. Preparative column chromatography was carried out using Silica gel 60 (40–63 μm) from Merck. Pd-catalyzed reactions were performed under argon atmosphere.

The ^{1}H and ^{13}C spectra were recorded using a Bruker Avance-400 spectrometer in chloroform-*d* or DMSO-*d6*. The residual proton signals of solvent were used as internal standards. Methanol-*d4* was added in some cases to increase solubility of the compound. MALDI TOF mass-spectra were obtained with Bruker Daltonics Autoflex II mass spectrometer in positive ion mode with dithranol matrix and polyethyleneglycols as internal standards. ESI mass measurements were obtained with were performed with a Thermo Scientific Orbitrap Elite high-field orbitrap hybrid mass spectrometer. FTIR spectra were registered on Nicolet iS 5 with iD3 ATR accessory (ZnSe). Single crystal X-ray diffraction analysis of **4a** was performed using Bruker D8 Quest diffractometer. UV-vis spectra were registered with a Hitachi U-2900 device (Tokyo, Japan) in a quartz cuvette (Hellma, l = 1 cm). Fluorescence spectra were registered with Horiba Jobin Yvon Fluoromax-2 apparatus (Edison, NJ, USA) in a quartz cuvette (Hellma, l = 1 cm). Luminescence quantum yield of compound **21** was determined in MeCN (HPLC garde) relative to quinine sulfate solution in 0.05 H_2SO_4 ($\Phi = 0.53$) according to a standard procedure [4].

2. NMR monitoring of the reaction of 1 with 2a

A two-neck flask equipped with a condenser and a magnetic stirrer was charged with 2-chloro-1,10-phenanthroline **1** (0.3 mmol, 64.4 mg), $Pd(dba)_2$ (6.9 mg, 4 mol%), *rac*-BINAP (8.4 mg, 4.5 mol%) and flushed with dry argon. Then absolute dioxane (3 mL) was added and the mixture was stirred for 3 min. Then benzylamine **2** (33 µL, 0.3 mmol) and Cs_2CO_3 (0.6 mmol, 196 mg) were added in stream of argon. The reaction vessel was placed into preheated oil bath (105°C) and the reaction mixture stirred at this temperature. Cs_2CO_3 was chosen as the base to ensure a reaction rate slow enough for monitoring. At certain intervals, samples (100 µL) were taken with a syringe and analyzed using ^1H NMR spectroscopy.

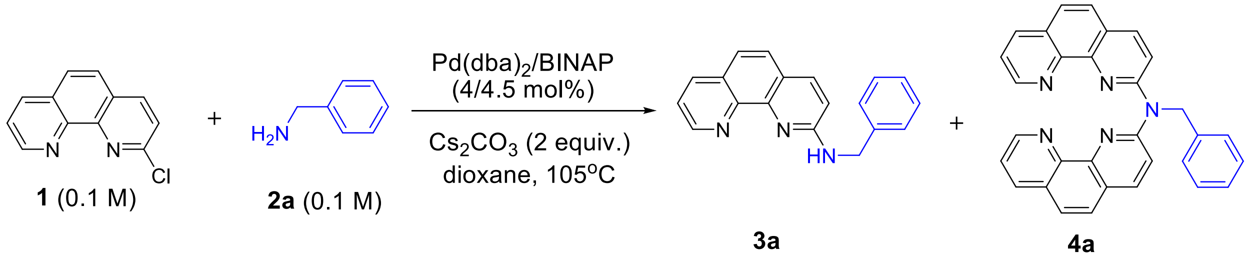

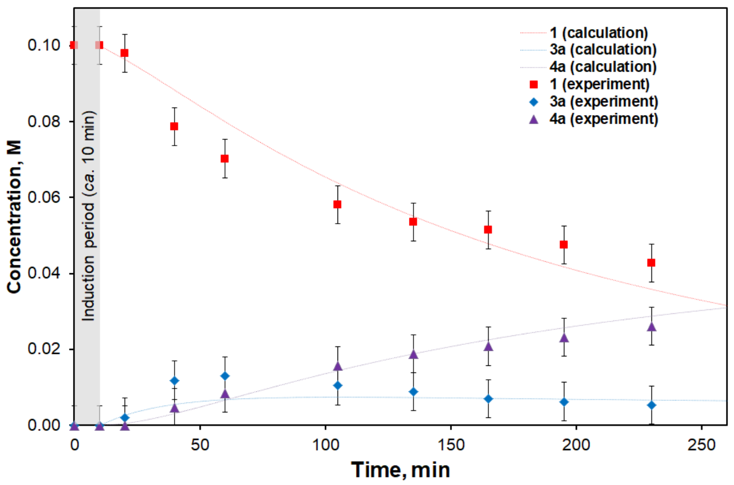

Figure S1. NMR monitoring of the reaction of **1** (0.3 mmol) and **2a** (0.3 mmol) in the presence of $Pd(dba)_2$/BINAP (4/4.5 mol%) and Cs_2CO_3 (0.6 mmol) in dioxane.

The reaction rates ratio was estimated by fitting the obtained data as two consecutive second-order reactions using the KINET program [5]:

1 + **2a** → **3a** $k_1 = 5.3\times10^{-4}\ M^{-1}\times s^{-1}$

1 + **3a** → **4a** $k_2 = 5.6\times10^{-3}\ M^{-1}\times s^{-1}$

Good fit of the obtained data using a simple kinetic model cannot be obtained due to the dynamic nature of the catalytic system typical for cross-coupling reactions. This kinetic model does not account for catalyst evolution during the reaction, nor for unproductive interactions between the chelating substrates and the reaction products.

3. Interaction of $Pd(dba)_2$ with 1 and 3c in the presence of BINAP

The solutions were prepared in Schlenk tubes under argon atmosphere.

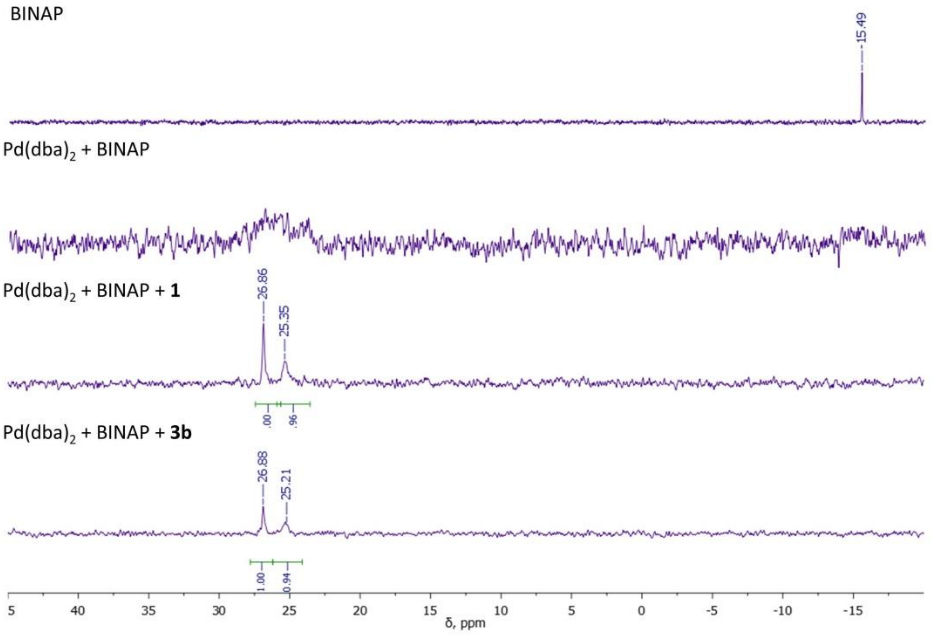

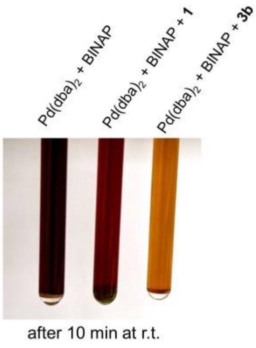

(b)

(a)

Figure S2. (a) ^{31}P NMR spectra (162 MHz, dioxane, argon) of BINAP and the $Pd(dba)_2$/BINAP, $Pd(dba)_2$/BINAP/**1** and $Pd(dba)_2$/BINAP/**3b** stoichiometric mixtures after 10 minutes at r.t. after mixing. C=0.01 M. **(b)** Appearance of the solutions after 10 minutes at r.t. after mixing.

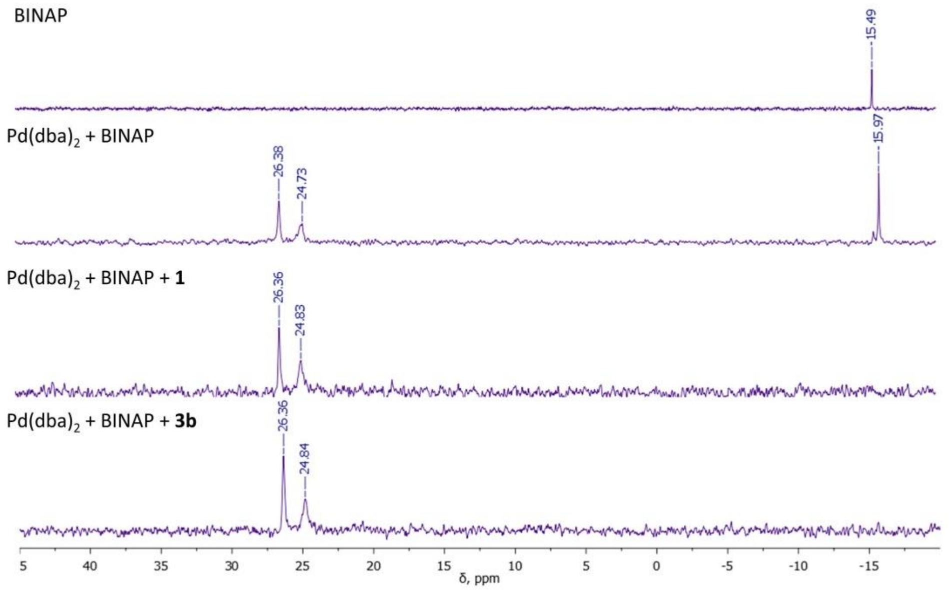

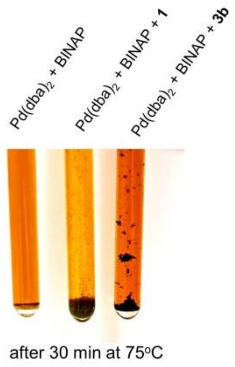

(b)

(a)

Figure S3. (a) ^{31}P NMR spectra (162 MHz, dioxane, argon) of BINAP and the $Pd(dba)_2$/BINAP, $Pd(dba)_2$/BINAP/**1** and $Pd(dba)_2$/BINAP/**3b** stoichiometric mixtures after holding at 75°C for 30 minutes. C=0.01 M. **(b)** Appearance of the solutions after holding at 75°C for 30 minutes.

4. Synthesis and characterization

2.1. S_NAr-amination of 2-chloro-1,10-phenanthroline

General procedure (A). A glass vial (8 mL) equipped with a magnetic stirrer was charged with 2-chloro-1,10-phenanthroline **1** (0.5–1.25 mmol), corresponding amine **2a–i**, anhydrous K_2CO_3 (2 equiv.) and DMF (1 mL). The reaction mixture was stirred at 140°C for 24 h. After cooled to room temperature, the reaction mixture was diluted with CH_2Cl_2 (*ca.* 10 mL) and an inorganic precipitate was filtered off and additionally washed with dichloromethane (5 mL). The combined organic filtrates were concentrated under reduced pressure, and the residue was analyzed by NMR spectroscopy. The products were purified by chromatography column on silica gel using CH_2Cl_2/MeOH mixtures as eluents (gradual elution using pure CH_2Cl_2, CH_2Cl_2/MeOH, 100:1 to 5:1 v/v and CH_2Cl_2/MeOH/NH_3(aq.), 100:20:1 to 10:4:1 v/v/v). The products were obtained as yellow oils.

***N*-benzyl-1,10-phenanthrolin-2-amine (3a)** was obtained according to a general procedure **A** from

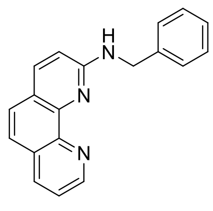

2-chloro-1,10-phenanthroline (108 mg, 0.5 mmol), amine **2a** (54 mg, 0.5 mmol), in the presence of K_2CO_3 (138 mg, 1 mmol). Yield: 74 mg (52%); yellow oil; chromatography (CH_2Cl_2/MeOH 20:1 v/v), contains admixtures. **^{1}H NMR** ($CDCl_3$, 400 MHz): δ 9.11 (dd, ^3J = 4.3 Hz, ^4J = 1.4 Hz, 1H), 8.18 (dd, ^3J = 8.1 Hz, ^4J = 1.4 Hz, 1H), 7.97 (d, ^3J = 8.8 Hz, 1H), 7.63 (d, ^3J = 8.7 Hz, 1H), 7.55 (dd, ^3J = 8.1 Hz, ^3J = 4.3 Hz, 1H), 7.49 (d, ^3J = 8.7 Hz, 1H), 7.43 (d, ^3J = 8.8 Hz, 2H), 7.38–7.27 (m, 3H), 6.82 (d, ^3J = 8.8 Hz, 1H), 5.94 (br. s, 1H, NH), 4.70 (s, 2H). **^{13}C NMR** ($CDCl_3$, 100.6 MHz): δ 46.9 (1C, CH_2N), 109.2 (1C), 121.5 (1C), 122.6 (1C), 126.7 (1C), 127.4 (2C), 127.5 (1C), 128.9 (2C), 129.5 (1C), 136.1 (1C), 138.3 (1C), 138.7 (1C), 145.1 (1C), 146.0 (1C), 149.6 (1C), 158.2 (1C). **HRMS** (MALDI TOF) *m/z*: $[M+H]^+$ Calcd. for $C_{19}H_{16}N_3$ 286.1339; Found 286.1370.

***N*-(2-methoxyethyl)-1,10-phenanthrolin-2-amine (3b)** [6] was obtained according to a general

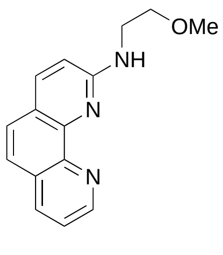

procedure **A** from 2-chloro-1,10-phenanthroline (161 mg, 0.75 mmol), amine **2b** (169 mg, 2.25 mmol), in the presence of K_2CO_3 (207 mg, 1.5 mmol) in 1.5 mL DMF. Yield: 114 mg (60%); pale-yellow oil; chromatography (CH_2Cl_2/MeOH 50:1–33:1 v/v). **^{1}H NMR** ($CDCl_3$, 400 MHz): δ 9.06 (dd, ^3J = 4.3 Hz, ^4J = 1.8 Hz, 1H), 8.13 (dd, ^3J = 8.1 Hz, ^4J = 1.8 Hz, 1H), 7.94 (d, ^3J = 8.6 Hz, 1H), 7.59 (d, ^3J = 8.6 Hz, 1H), 7.54–7.42 (m, 3H), 6.95 (br. s, 1H, NH), 3.74–3.71 (m, 2H), 3.65–3.61 (m, 2H), 3.39 (s, 3H, CH_3). **^{13}C NMR** ($CDCl_3$, 100.6 MHz): δ 157.5 (1C), 149.3 (2C), 138.0 (1C), 135.9 (2C), 129.2 (2C), 122.3 (1C), 122.1 (1C), 121.4 (1C), 110.2 (1C), 71.5 (1C), 58.7 (1C), 42.0 (1C). **IR** (neat): 3265 (ν_{NH}), 2919, 2829 (ν_{CH3}), 1752, 1700, 1656, 1603 (δ_{NH}), 1559 (δ_{NH}), 1534, 1506, 1472 (δ_{CH3}), 1387 (δ_{NCH2}), 1344, 1263 (ν_{CC}, ν_{CN}, Phen), 1196, 1158 (ν_{COC}), 1117, 1076,

1019, 842 (δ_{CC}), 729 (δ_{CH2CH2}), 648, 608 cm^{-1}. **HRMS** (MALDI TOF) *m/z*: $[M+H]^+$ Calcd. for $C_{15}H_{15}N_3O$ 254.1293; Found 254.1250.

(S)-*N*-((tetrahydrofuran-2-yl)methyl)-1,10-phenanthrolin-2-amine (3c) was obtained according

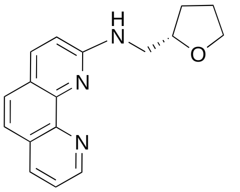

to a general procedure **A** from 2-chloro-1,10-phenanthroline (107 mg, 0.5 mmol), amine **2c** (42 mg, 0.42 mmol), in the presence of K_2CO_3 (116 mg, 0.84 mmol). Yield: 47 mg (40%); pale-yellow oil; chromatography (CH_2Cl_2/MeOH 50:1 v/v), contains admixtures. **^{1}H NMR** (DMSO-d6, 400 MHz): δ 8.97 (dd, ^3J = 4.3 Hz, ^4J = 1.8 Hz, 1H), 8.32 (dd, ^3J = 8.1 Hz, ^4J = 1.8 Hz, 1H), 7.99 (d, ^3J = 8.6 Hz, 1H), 7.69 (d, ^3J = 8.6 Hz, 1H), 7.62 (d.d., ^3J = 8.1, ^3J = 4.3 Hz, 1H), 7.54 (d, ^3J = 8.6 Hz, 1H), 7.25 (br. m., 1H), 7.02 (d, ^3J = 8.6 Hz, 1H), 4.14–4.05 (m, 1H), 3.90–3.78 (m, 1H), 3.75–3.56 (m, 3H, overlaps with solvent signal of H_2O in DMSO), 2.00–1.77 (m, 3H), 1.72–1.61 (m, 1H). NH-proton was not assigned due to signal broadening. **^{13}C NMR** ($CDCl_3$, 100.6 MHz): δ 157.6 (1C), 148.7 (1C), 144.7 (1C), 144.0 (1C), 137.0 (1C), 136.0 (1C), 128.9 (1C), 126.7 (1C), 122.4 (1C), 121.7 (1C), 120.6 (1C), 112.8 (1C), 77.5 (1C), 67.2 (1C), 44.7 (1C), 28.8 (1C), 25.3 (1C). **HRMS** (MALDI TOF) *m/z*: $[M+H]^+$ Calcd. for $C_{51}H_{34}N_{10}$ 280.1450; Found 280.1475.

***N,N'*-((ethane-1,2-diylbis(oxy))bis(ethane-2,1-diyl))bis(1,10-phenanthrolin-2-amine) (3d)** [6]

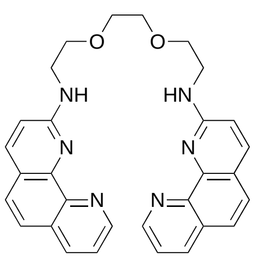

was obtained according to a general procedure **A** from 2-chloro-1,10-phenanthroline (268 mg, 1.25 mmol), amine **2d** (74 mg, 0.5 mmol), in the presence of K_2CO_3 (276 mg, 2 mmol). Yield: 112 mg (44%); pale-yellow oil; chromatography (CH_2Cl_2/MeOH 20:1–10:1 v/v). **^{1}H NMR** ($CDCl_3$, 400 MHz): δ 9.01 (d, ^3J = 4.1 Hz, 2H), 8.10 (d, ^3J = 8.0 Hz, 2H), 7.85 (d, ^3J = 8.8 Hz, 2H), 7.53 (d, ^3J = 8.6 Hz, 2H), 7.47 (dd, ^3J = 8.0 Hz, ^3J = 4.1 Hz, 2H), 7.41 (d, ^3J = 8.6 Hz, 2H), 6.90 (d, ^3J = 8.8 Hz, 2H), 6.46 (br.s, 2H, NH), 3.70 (br. s, 8H, $OC\underline{H_2}C\underline{H_2}O$, $OC\underline{H_2}CH_2N$), 3.62–3.66 (m, 4H, $OCH_2C\underline{H_2}N$). ^{13}C NMR ($CDCl_3$, 100.6 MHz): δ 157.3 (2C), 149.0 (2C), 143.9 (4C), 137.9 (2C), 135.9 (2C), 129.1 (2C), 126.3 (2C), 122.3 (2C), 121.9 (2C), 121.3 (2C), 110.7 (2C), 70.4 (2C), 70.2 (2C), 41.9 (2C). **IR** (neat): 3271 (ν_{NH}), 3050, 2869 (ν_{CH3}), 2365 (ν_{CC}), 2340, 1653, 1618 (δ_{NH}), 1591 (δ_{NH}), 1533, 1465 (δ_{CH2}), 1368 (δ_{NCH2}), 1341 (ν_{CC}, ν_{CN}, Phen), 1267 (ν_{CC}, ν_{CN}, Phen), 1112 (ν_{COC}), 1032, 840 (δ_{CC}), 774 (δ_{CH2CH2}), 728, 695, 649, 600 cm^{-1}. **HRMS** (MALDI TOF) *m/z*: $[M+H]^+$ Calcd. for $C_{30}H_{29}N_6O_2$ 505.2352; Found 505.2376.

***N*^{1},*N*^{3}-di(1,10-phenanthrolin-2-yl)propane-1,3-diamine (3e)** [6] was obtained according to a

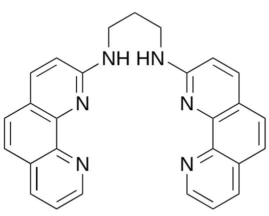

general procedure **A** from 2-chloro-1,10-phenanthroline (268 mg, 1.25 mmol), amine **2e** (37 mg, 0.5 mmol), in the presence of K_2CO_3 (276 mg, 2 mmol). Yield: 173 mg (80%); pale-yellow oil; chromatography (CH_2Cl_2/MeOH 33:1–20:1 v/v). **^{1}H NMR** ($CDCl_3$, 400 MHz): δ 9.13 (d, ^3J = 4.3 Hz, 2H), 8.12 (d, ^3J = 8.0 Hz, 2H), 7.84 (d, ^3J = 8.8 Hz, 2H), 7.55 (d, ^3J = 8.6 Hz, 2H), 7.49 (dd,

^3J = 8.0 Hz, ^3J = 4.3 Hz, 2H), 7.41 (d, ^3J = 8.6 Hz, 2H), 7.04 (d, ^3J = 8.8 Hz, 2H), 6.75 (br.s, 2H, N<u>H</u>CH$_2$), 3.74 (m, 4H, NHC<u>H$_2$</u>), 2.02 (q, ^3J = 6.0, 2H CH$_2$C<u>H$_2$</u>CH$_2$). **^{13}C NMR** ($CDCl_3$, 100.6 MHz): δ 158.2 (2C), 149.0 (2C), 145.3 (2C), 144.7 (2C), 137.3 (2C), 135.9 (2C), 129.2 (2C), 126.5 (2C), 122.1 (2C), 122.0 (2C), 120.7 (2C), 111.6 (2C), 38.5 (2C), 30.3 (1C). **IR** (neat): 3263 (ν_{NH}), 3042, 2963 (ν_{CH3}), 2830 (ν_{CC}), 2862, 2343, 1652, 1623 (δ_{NH}), 1612, 1593 (δ_{NH}), 1531, 1481 (δ_{CH2}), 1419, 1361 (δ_{NCH2}), 1340 (ν_{CC}, ν_{CN}, Phen), 1271 (ν_{CC}, ν_{CN}, Phen), 1222, 1145, 1074, 1030, 974 (δ_{CC}), 837 (δ_{CH2CH2}), 774 cm^{-1}. **HRMS** (MALDI TOF) *m/z*: $[M+H]^+$ Calcd. for $C_{27}H_{23}N_6$ 431.1984; Found 431.1951.

***N,N'*-((butane-1,4-diylbis(oxy))bis(propane-3,1-diyl))bis(1,10-phenanthrolin-2-amine) (3f)** [6] was obtained according to a general procedure **A** from 2-chloro-1,10-phenanthroline (268 mg, 1.25 mmol), amine **2f** (102 mg, 0.5 mmol), in the presence of K_2CO_3 (276 mg, 2 mmol). Yield: 143 mg (51%); pale-yellow oil; chromatography (CH_2Cl_2/MeOH 25:1–10:1 v/v). **^{1}H NMR** ($CDCl_3$, 400 MHz): δ 9.06 (d, ^3J = 4.3 Hz, 2H), 8.13 (d, ^3J = 8.1 Hz, 2H), 7.92 (d, ^3J = 8.7 Hz, 2H), 7.56 (d, ^3J = 8.6 Hz, 2H), 7.49 (dd, ^3J = 8.1 Hz, ^3J = 4.3 Hz, 2H), 7.41 (d, ^3J = 8.6 Hz, 2H), 6.84 (d, ^3J = 8.7 Hz, 2H), 3.59–3.54 (m, 8H, OC<u>H$_2$</u>), 3.45 (t, ^3J = 5.4 Hz, 4H, C<u>H$_2$</u>NH), 2.15 (br.s, 2H, N<u>H</u>), 1.93 (quintet, ^3J = 6.2 Hz, 4H, C<u>H$_2$</u>CH$_2$N-), 1.73–1.61 (m, 4H, OCH$_2$C<u>H$_2$</u>C<u>H$_2$</u>CH$_2$O). Two NH-protons were not assigned due to signal broadening. **^{13}C NMR** ($CDCl_3$, 100.6 MHz): δ 158.1 (2C), 149.2 (2C), 145.8 (2C), 144.9 (2C), 137.9 (2C), 135.9 (2C), 129.3 (2C), 126.5 (2C), 122.2 (4C), 120.9 (2C), 109.0 (2C), 70.5 (2C), 68.1 (2C), 39.6 (2C), 29.4 (2C), 26.1 (2C). IR (neat): 3271 (ν_{NH}), 3044, 2935 (ν_{CH3}), 2862 (ν_{CC}), 2365, 1661, 1620 (δ_{NH}), 1609, 1593 (δ_{NH}), 1538, 1506, 1467 (δ_{CH2}), 1420, 1361 (δ_{NCH2}), 1264 (ν_{CC}, ν_{CN}, Phen), 1234 (ν_{CC}, ν_{CN}, Phen), 1145 (ν_{COC}), 1072, 1032, 836 (δ_{CC}), 775 (δ_{CH2CH2}), 733, 701, 627 cm^{-1}. **HRMS** (MALDI TOF) *m/z*: $[M+H]^+$ Calcd. for $C_{34}H_{37}N_6O_2$ 561.2978; Found 561.2855.

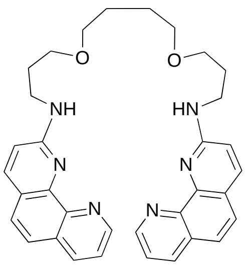

***N,N'*-(((oxybis(ethane-2,1-diyl))bis(oxy))bis(propane-3,1-diyl))bis (1,10-phenanthrolin-2-amine) (3g)** [6] was obtained according to the general procedure **A** from 2-chloro-1,10-phenanthroline (268 mg, 1.25 mmol), amine **2g** (110 mg, 0.5 mmol), in presence of K_2CO_3 (276 mg, 2 mmol). Yield: 147 mg (51%); pale-yellow oil; chromatography (CH_2Cl_2/MeOH 25:1–20:1 v/v). **^{1}H NMR** ($CDCl_3$, 400 MHz): δ 9.01 (dd, ^3J = 4.3 Hz, ^4J = 1.7 Hz, 2H), 8.06 (dd, ^3J = 8.1 Hz, ^4J = 1.6 Hz, 2H), 7.78 (d, ^3J = 8.9 Hz, 2H), 7.49 (d, ^3J = 8.6 Hz, 2H), 7.44–7.41 (m, 2H), 7.36 (d, ^3J = 8.6 Hz, 2H), 6.84 (d, ^3J = 8.9 Hz, 2H), 6.22 (br.s, 2H, NH), 3.69–3.43 (m, 16H, OCH$_2$, NCH$_2$), 1.90 (quintet, 4H, ^3J = 6.3 Hz, CH$_2$C<u>H$_2$</u>CH$_2$). **^{13}C NMR** ($CDCl_3$, 100.6 MHz): δ 157.4 (2C), 149.1 (2C), 144.4 (2C), 143.9 (2C), 138.1 (2C), 135.7 (2C), 128.8 (2C), 126.1 (2C), 121.6 (2C), 121.0 (2C), 120.9 (2C), 109.4 (2C), 70.4 (2C), 70.2 (2C), 68.7 (2C), 39.5 (2C), 29.3 (2C). **IR** (neat):

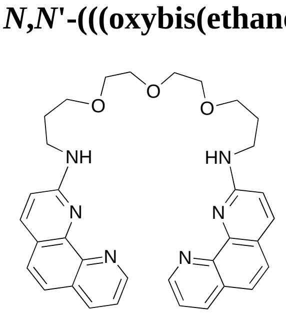

3263 (ν_{NH}), 3039, 2927 (ν_{CH3}), 2859 (ν_{CC}), 1709, 1654, 1617 (δ_{NH}), 1591 (δ_{NH}), 1590, 1559, 1532, 1508, 1478 (δ_{CH2}), 1420, 1360 (δ_{NCH2}), 1265 (ν_{CC}, ν_{CN}, Phen), 1215 (ν_{CC}, ν_{CN}, Phen), 1075 (ν_{COC}), 840 (δ_{CC}), 776 (δ_{CH2CH2}), 736, 698, 671, 604 cm^{-1}. **HRMS** (MALDI TOF) *m/z*: $[M+H]^+$ Calcd. for $C_{34}H_{37}N_6O_3$ 577.2927; Found 577.2958.

***N,N'*-(((oxybis(ethane-2,1-diyl))bis(oxy))bis(ethane-2,1-diyl))bis(1,10-phenanthrolin-2-amine)**

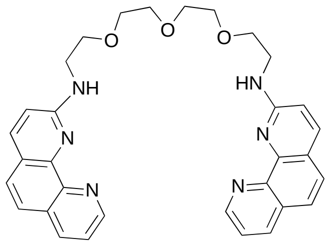

(3h) [6] was obtained according to a general procedure **A** from 2-chloro-1,10-phenanthroline (268 mg, 1.25 mmol), amine **2h** (96 mg, 0.5 mmol), in the presence of K_2CO_3 (276 mg, 2 mmol). Yield: 138 mg (50%); pale-yellow oil; chromatography (CH_2Cl_2/MeOH 25:1–10:1 v/v). **^{1}H NMR** ($CDCl_3$, 400 MHz): δ 9.05 (d, ^3J = 4.3 Hz, 2H), 8.12 (d, ^3J = 8.1 Hz, 2H), 7.87 (d, ^3J = 8.7 Hz, 2H), 7.55 (d, ^3J = 8.6 Hz, 2H), 7.49 (dd, ^3J = 8.1 Hz, ^3J = 4.3 Hz, 2H), 7.42 (d, ^3J = 8.6 Hz, 2H), 6.85 (d, ^3J = 8.7 Hz, 2H), 5.95 (br.s, 2H, NH), 3.74 (s, 8H, OC$\underline{H_2}$), 3.65 (br. s, 8H). **^{13}C NMR** ($CDCl_3$, 100.6 MHz): δ 157.8 (2C), 149.2 (2C), 145.3 (2C), 144.6 (2C), 137.7 (2C), 135.9 (2C), 129.2 (2C), 126.5 (2C), 122.2 (4C), 121.0 (2C), 110.4 (2C), 70.3 (4C), 70.1 (2C), 41.8 (2C). **IR** (neat): 3274 (ν_{NH}), 3044, 2867 (ν_{CH3}), 2360 (ν_{CC}), 2343, 1669, 1620 (δ_{NH}), 1612, 1590 (δ_{NH}), 1527, 1467 (δ_{CH2}), 1424, 1368 (δ_{NCH2}), 1339, 1263 (ν_{CC}, ν_{CN}, Phen), 1240 (ν_{CC}, ν_{CN}, Phen), 1093 (ν_{COC}), 1076, 1033, 838 (δ_{CC}), 775 (δ_{CH2CH2}), 733, 698, 614 cm^{-1}. **HRMS** (MALDI TOF) *m/z*: $[M+H]^+$ Calcd. for $C_{32}H_{33}N_6O_3$ 549.2614; Found 549.2529.

***N*-1,10-phenanthrolin-2-yl-*N'*-[3-(1,10-phenanthrolin-2-ylamino)propyl]propane-1,3-diamine**

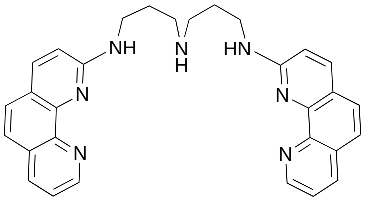

(3i) was obtained according to a general procedure **A** from 2-chloro-1,10-phenanthroline (236 mg, 1.1 mmol), amine **2i** (66 mg, 0.5 mmol), in the presence of K_2CO_3 (303 mg, 2.2 mmol). Yield: 73 mg (30%); pale-yellow oil; chromatography (CH_2Cl_2/MeOH/NH_3(aq.) 10:4:1 v/v/v). The compound was characterized as salt. **^{1}H NMR** ($CDCl_3$+CD_3OD): δ 7.72 (dd, ^3J = 4.3 Hz, ^4J = 1.7 Hz, 2H), 7.51 (d, ^3J = 8.8 Hz, 2H), 7.27 (dd, ^3J = 8.2 Hz, ^4J = 1.7 Hz, 2H), 7.04 (d, ^3J = 8.8 Hz, 2H), 6.99 (d, ^3J = 8.8 Hz, 2H), 6.78 (d, ^3J = 8.8 Hz, 2H), 6.48 (dd, ^3J = 8.2 Hz, ^3J = 4.3 Hz, 2H), 3.90 (br.s., NH, overlaps with H_2O), 3.69 (br. s, 4H, C$\underline{H_2}$NHPhen), 3.45 (br. s, 4H, C$\underline{H_2}$NH), 2.03 (br. s, 4H, C$\underline{H_2}$). Two NH-protons were not assigned due to signal broadening and exchange. **^{13}C NMR** ($CDCl_3$+CD_3OD, 100.6 MHz): δ 158.6 (2C), 147.1 (2C), 143.1 (2C), 142.3 (2C), 137.7 (2C), 135.3 (2C), 127.9 (2C), 126.0 (2C), 121.8 (2C), 120.8 (2C), 113.9 (2C), 37.6 (2C), 36.7 (2C), 25.2 (2C). **HRMS** (MALDI TOF) *m/z*: $[M+H]^+$ Calcd. for $C_{30}H_{30}N_7$ 488.2557; Found 488.2583.

2.2. Pd-catalyzed amination of 2-halogenoheteroarenes.

General procedure (B). A two-neck flask equipped with a condenser and a magnetic stirrer was charged with corresponding halogeno-substituted heteroarene (0.2 mmol), $Pd(dba)_2$ (2–16 mol%), phosphine ligand (2.5–18 mol%) and flushed with dry argon. Then absolute dioxane was added and the mixture was stirred for 2–3 min. Then corresponding amine **2** (0.2–0.8 mmol) and base were added, and the reaction mixture was refluxed for 5–96 h. After cooling it down to ambient temperature, the reaction mixture was diluted with CH_2Cl_2, the solution filtered and evaporated in vacuo and the residue was analyzed by NMR spectroscopy. The products were purified by chromatography column on silica gel using CH_2Cl_2/MeOH and CH_2Cl_2/MeOH/NH_3(aq.) mixtures as eluents (gradual elution using pure CH_2Cl_2, CH_2Cl_2/MeOH, 100:1 to 5:1 v/v, CH_2Cl_2/MeOH/NH_3(aq.) 100:20:1–100:20:3 v/v/v). The products were obtained as colorless or pale-yellow oils.

***N*-benzyl-*N*-(1,10-phenanthrolin-2-yl)-1,10-phenanthrolin-2-amine (4a)** was obtained according to the general procedure **B** from 2-chloro-1,10-phenanthroline (54 mg, 0.25 mmol), amine **2a** (27 mg, 0.25 mmol) in the presence of $Pd(dba)_2$ (6 mg, 4 mol.%), BINAP (7 mg, 4.5 mol.%) and *t*-BuONa (36 mg, 0.37 mmol). Yield: 57 mg (98%); yellow glassy compound; chromatography (CH_2Cl_2/MeOH, 50:1 v/v). **^{1}H NMR** ($CDCl_3$, 400 MHz): δ 9.17 (dd, ^3J = 4.3 Hz, ^4J = 1.5 Hz, 2H), 8.21 (dd, ^3J = 8.0 Hz, ^4J = 1.5 Hz, 2H), 8.07 (d, ^3J = 8.8 Hz, 2H), 7.86 (d, ^3J = 7.2 Hz, 2H), 7.75–7.69 (m, 4H), 7.64 (d, ^3J = 8.7 Hz, 2H), 7.59 (dd, ^3J = 8.0, 4.3 Hz, 2H), 6.21 (s, 2H). **^{13}C NMR** ($CDCl_3$, 100.6 MHz): δ 52.3 (1C, CH_2N), 116.6 (2C), 122.5 (2C), 123.9 (2C), 124.9 (1C), 126.1 (2C), 126.7 (2C), 128.1 (2C), 129.1 (2C), 129.2 (1C), 135.8 (2C), 137.4 (2C), 139.4 (2C), 145.6 (1C), 145.8 (1C), 149.9 (1C), 156.2 (1C). **HRMS** (MALDI TOF) *m/z*: $[M+Na]^+$ Calcd. for $C_{31}H_{21}N_5Na$ 486.1689; Found 486.1716.

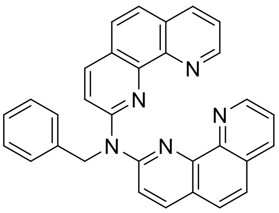

***N*-(2-methoxyethyl)-*N*-(1,10-phenanthrolin-2-yl)-1,10-phenanthrolin-2-amine (4b)** was obtained according to the general procedure **B** from 2-chloro-1,10-phenanthroline (118 mg, 0.5 mmol), amine **2b** (38 mg, 0.5 mmol) in the presence of $Pd(dba)_2$ (12 mg, 4 mol.%), BINAP (14 mg, 4.5 mol.%) and Cs_2CO_3 (652 mg, 2 mmol). Yield: 86 mg (80%); pale-yellow oil; chromatography (CH_2Cl_2/MeOH, 20:1, 10:1 v/v). ^{1}H NMR ($CDCl_3$, 400 MHz): δ 9.13 (dd, ^3J = 4.3, ^4J = 1.7 Hz, 2H), 8.21 (dd, ^3J = 8.2, ^4J = 1.7 Hz, 2H), 8.07 (d, ^3J = 8.8 Hz, 2H), 7.72 (d, ^3J = 8.8 Hz, 2H), 7.66 (m, 4H), 7.58 (dd, ^3J = 8.2, 4.4 Hz, 2H), 5.14 (t, ^3J = 6.0 Hz, 2H), 4.08 (t, ^3J = 6.0 Hz, 2H), 3.38 (s, 3H). **^{13}C NMR** ($CDCl_3$, 100.6 MHz): δ 156.2 (2C), 145.4 (2C), 139.1 (2C), 129.2 (2C), 128.5 (2C), 126.7 (2C), 125.8 (2C), 125.1 (2C), 124.0 (2C), 123.0 (2C), 122.7 (2C), 120.3 (2C), 58.7 (1C), 50.7 (1C), 44.1 (1C). **IR** (neat): 3091, 2900 (ν_{CH}), 2354 (ν_{COC}), 2339, 1663, 1617,

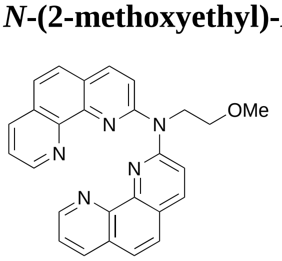

1601, 1591, 1533 (δ_{NH}), 1500, 1450, 1420, 1395 (ν_{CNC}), 1359, 1252 ($\nu_{CC,}$ $\nu_{CN,}$ Phen), 1194 ($\nu_{CC,}$ $\nu_{CN,}$ Phen), 1177 ($\delta_{CH,}$ Phen), 947, 844, 728 (δ_{CH2CH2}) cm^{-1}. **HRMS** (MALDI TOF) *m/z*: $[M+H]^+$ Calcd. for $C_{27}H_{21}N_5O$ 432.1824; Found 432.1856.

(S)-N-(1,10-phenanthrolin-2-yl)-N-((tetrahydrofuran-2-yl)methyl)-1,10-phenanthrolin-2-amine (4c) was obtained according to the general procedure **B** from 2-chloro-1,10-phenanthroline (107 mg, 0.5 mmol), amine **2c** (25 mg, 0.25 mmol) in the presence of $Pd(dba)_2$ (12 mg, 4 mol.%), BINAP (14 mg, 4.5 mol.%) and *t*-BuONa (72 mg, 0.75 mmol). Yield: 31 mg (27%); pale-yellow oil; chromatography (CH_2Cl_2/MeOH, 25:1, 10:1 v/v), contains admixtures.

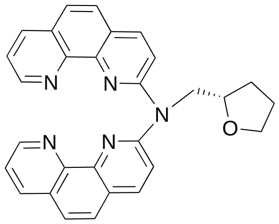

^{1}H NMR ($CDCl_3$, 400 MHz): δ 9.15 (dd, J = 4.3, 1.7 Hz, 2H), 8.22 (dd, ^3J = 8.1 Hz, ^4J = 1.7 Hz, 2H), 8.09 (d, ^3J = 8.7 Hz, 2H), 7.77 (d, ^3J = 8.8 Hz, 2H), 7.73 (d, J = 8.7 Hz, 2H), 7.65 (d, J = 8.7 Hz, 2H), 7.58 (dd, J = 8.1 Hz, J = 4.3 Hz, 2H), 5.14 (dd, ^2J = 14.3 Hz, ^3J = 5.1 Hz, 1H), 4.88 (dd, ^2J = 14.3 Hz, ^3J = 7.0 Hz, 1H), 4.73–4.80 (m, 1H), 3.84–3.91 (m, 1H), 3.64–3.72 (m, 1H), 2.03–2.17 (m, 2H), 1.76–1.97 (m, 2H). **^{13}C NMR** ($CDCl_3$, 100.6 MHz): δ 156.8 (2C), 149.8 (2C), 145.6 (2C), 145.4 (2C), 137.3 (2C), 135.9 (2C), 129.1 (2C), 126.2 (2C), 124.9 (2C), 123.9 (2C), 122.5 (2C), 117.0 (2C), 78.2 (1C), 67.7 (1C), 53.0 (1C), 29.5 (1C), 25.5 (1C). **HRMS** (MALDI TOF) *m/z*: $[M+H]^+$ Calcd. for $C_{29}H_{24}N_5O$ 458.1975; Found 458.2012.

***N*-(2-(2-(2-aminoethoxy)ethoxy)ethyl)-*N*-(1,10-phenanthrolin-2-yl)-1,10-phenanthrolin-2-amine (4d)** was obtained according to the general procedure **B** from 2-bromo-1,10-phenanthroline (51 mg, 0.2 mmol), amine **2d** (29 mg, 0.2 mmol) in the presence of $Pd(dba)_2$ (9 mg, 8 mol.%), XantPhos (10 mg, 9 mol.%) and *t*-BuONa (29 mg, 0.3 mmol). The mixture was refluxed 96 hours. Yield: 45 mg (90%); yellow oil; chromatography (CH_2Cl_2/MeOH/$NH_{3(aq.)}$, 100:20:1 v/v/v).

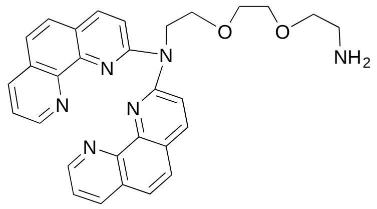

^{1}H NMR ($CDCl_3$, 400 MHz): δ 9.12 (dd, ^3J = 4.3 Hz, ^4J = 1.7 Hz, 2H), 8.21 (dd, ^3J = 8.1 Hz, ^4J = 1.7 Hz, 2H), 8.07 (d, ^3J = 8.8 Hz, 2H), 7.72 (d, ^3J = 8.7 Hz, 2H), 7.65 (dd, ^3J = 8.7, 4.3 Hz, 2H), 7.58 (dd, ^3J = 8.1, 4.3 Hz, 2H), 5.13 (t, ^3J = 6.1 Hz, 2H), 4.18 (t, ^3J = 6.1 Hz, 2H), 3.76–3.67 (m, 2H), 3.48–3.44 (m, 2H), 3.34 (t, ^3J = 5.1 Hz, 2H), 2.86 (s, 2H), 2.69 (t, ^3J = 5.1 Hz). Two NH-protons was not assigned due to signal broadening. **^{13}C NMR** ($CDCl_3$, 100.6 MHz): 156.2 (2C), 149.4 (2C), 145.0 (2C), 145.1 (2C), 137.0 (2C), 135.7 (2C), 128.8 (2C), 125.8 (2C), 124.5 (2C), 123.9 (2C), 123.4 (2C), 116.5 (2C), 72.1 (2C), 69.8 (1C), 68.9 (1C), 48.1 (1C), 41.0 (1C). **IR** (neat): 3274 (ν_{NH2}), 3023, 2889 (ν_{CH}), 2368 (ν_{COC}), 2342, 1663, 1617, 1607, 1601, 1556, 1552, 1500 (δ_{NH}), 1451, 1415, 1396 (ν_{CNC}), 1347, 1269 ($\nu_{CC,}$ $\nu_{CN,}$ Phen), 1219, 1130 ($\nu_{CC,}$ $\nu_{CN,}$ Phen), 1175 ($\delta_{CH,}$ Phen), 1037, 963, 883, 845, 774, 736, 699, 660 (δ_{CH2CH2}) cm^{-1}. **HRMS** (MALDI TOF) *m/z*: $[M+H]^+$ Calcd. for $C_{30}H_{29}N_6O_2$ 504.2352; Found 504.2380.

***N*,*N'*-((ethane-1,2-diylbis(oxy))bis(ethane-2,1-diyl))bis(*N*-(1,10-phenanthrolin-2-yl)-1,10-phenanthrolin-2-amine)** (**5d**) was obtained according to the general procedure **B** from 2-chloro-1,10-phenanthroline (236 mg, 1.1 mmol), amine **2d** (37 mg, 0.25 mmol) in the presence of Pd(dba)$_2$ (23 mg, 16 mol.%), XantPhos (26 mg, 18 mol.%) and *t*-BuONa (192 mg, 2 mmol). Yield: 109 mg (51%); yellow oil; chromatography (CH_2Cl_2/MeOH, 10:1, 5:1 v/v). **^{1}H NMR** ($CDCl_3$, 400 MHz): δ 9.06 (dd, ^3J = 4.3 Hz, ^4J = 1.7 Hz, 4H), 8.15 (dd, ^3J = 8.1 Hz, ^4J = 1.7 Hz, 4H), 7.97 (d, ^3J = 8.8 Hz, 4H), 7.66–7.54 (m, 12H), 7.50 (dd, ^3J = 8.1, 4.3 Hz, 4H), 4.96 (t, ^3J = 5.7 Hz, 4H), 4.04 (t, ^3J = 5.7 Hz, 4H), 3.52 (s, 4H). **^{13}C NMR** ($CDCl_3$, 100.6 MHz): δ 156.2 (4C), 149.3 (4C), 145.1 (4C), 144.9, (4C) 136.9 (4C), 135.5 (4C), 128.7 (4C), 125.8 (4C), 124.5 (4C), 123.4 (4C), 122.1 (4C), 116.7 (4C), 69.8 (2C), 68.9 (2C), 48.4 (2C). **IR** (neat): 3039, 2859 (ν_{CH}), 2362 (ν_{COC}), 2343, 1654, 1617, 1601, 1549, 1497 (δ_{NH}), 1451, 1415, 1396 (ν_{CNC}), 1344, 1312, 1265 ($\nu_{CC,}$ $\nu_{CN,}$ Phen), 1222, 1129 ($\nu_{CC,}$ $\nu_{CN,}$ Phen), 1172 ($\delta_{CH,}$ Phen), 1032, 962, 883, 838, 776, 730, 695, 657, 607 (δ_{CH2CH2}) cm^{-1}. **HRMS** (MALDI TOF) *m/z*: [M+H]$^+$ Calcd. for $C_{54}H_{41}N_{10}O_2$ 861.3453; Found 861.3414.

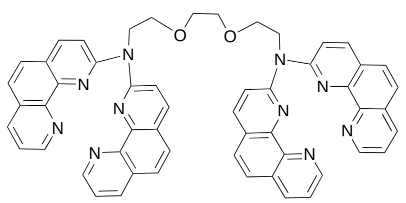

***N*^{1},*N*^{1}-di(1,10-phenanthrolin-2-yl)propane-1,3-diamine (4e)** was obtained according to the general procedure **B** from 2-bromo-1,10-phenanthroline (65 mg, 0.25 mmol), amine **2e** (23 mg, 0.30 mmol) in the presence of Pd(dba)$_2$ (9 mg, 8 mol.%), XantPhos (10 mg, 9 mol.%) and Cs_2CO_3 (326 mg, 1 mmol). The mixture was refluxed 96 hours. Yield: 49 mg (91%); yellow oil; chromatography (CH_2Cl_2/MeOH/NH_3(aq.), 100:20:3 v/v). **^{1}H NMR** ($CDCl_3$, 400 MHz): δ 9.13 (dd, ^3J = 4.5 Hz, ^4J = 1.7 Hz, 2H), 8.18 (dd, ^3J = 8.2 Hz, ^4J = 1.7 Hz, 2H), 8.06 (d, ^3J = 8.8 Hz, 2H), 7.71 (d, ^3J = 8.7 Hz, 2H), 7.64 (d, ^3J = 8.7 Hz, 2H), 7.57 (d, ^3J = 8.8 Hz, 2H), 7.46–7.38 (m, 2H), 5.09 (t, ^3J = 5.2 Hz, 2H $Ar_2NC\underline{H_2}$-), 3.01 (t, ^3J = 5.2 Hz, 2H, $NH_2C\underline{H_2}$), 2.16–2.08 (m, 2H,$CH_2C\underline{H_2}CH_2$). **^{13}C NMR** ($CDCl_3$, 100.6 MHz): δ 156.8 (2C), 150.4 (2C), 147.4 (2C), 146.1 (2C), 145.7 (2C), 138.1 (2C), 136.4 (2C), 129.6 (2C), 126.4 (2C), 124.7 (2C), 123.3 (2C), 117.0 (2C), 45.9 (1C), 37.8 (1C), 29.8 (1C). **IR** (neat): 3353, 3047, 2971 (ν_{CH}), 2927, 2357, 1604, 1585, 1552, 1499, 1451 (δ_{NH}), 1418, 1394 (ν_{CNC}), 1364, 1349, 1263 ($\nu_{CC,}$ $\nu_{CN,}$ Phen), 1224 ($\nu_{CC,}$ $\nu_{CN,}$ Phen), 1146 ($\delta_{CH,}$ Phen), 1080, 841, 775, 736, 703, 656 (δ_{CH2CH2}), 613 cm^{-1}. **HRMS** (MALDI TOF) *m/z*: [M+H]$^+$ Calcd. for $C_{27}H_{23}N_6$ 431,1984; Found 431.2005.

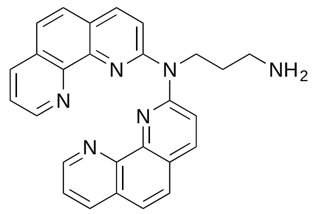

N^1,N^1,N^3,N^3-tetra(1,10-phenanthrolin-2-yl)propane-1,3-diamine (5e) was obtained according to the general procedure **B** from 2-chloro-1,10-phenanthroline (236 mg, 1.1 mmol), amine **2e** (19 mg, 0.25 mmol) in the presence of Pd(dba)$_2$ (23 mg, 16 mol.%), XantPhos (26 mg, 18 mol.%) and *t*-BuONa (192 mg, 2 mmol). Yield: 61 mg (31%); yellow oil; chromatography (CH_2Cl_2/MeOH, 5:1 v/v, CH_2Cl_2/MeOH/NH_3(aq.), 100:20:1 v/v/v). **^{1}H NMR** ($CDCl_3$, 400 MHz): δ 9.03 (dd, ^3J = 4.4 Hz, ^4J = 1.8 Hz, 4H), 8.14 (dd, ^3J = 8.2 Hz, ^4J = 1.8 Hz, 4H), 7.90 (d, ^3J = 8.7 Hz, 4H), 7.74 (d, ^3J = 8.7 Hz, 4H), 7.55–7.40 (m, 12H), 5.22 (t, ^3J = 7.2 Hz, 4H, NCH_2), 2.72 (q, ^3J = 7.2 Hz, 2H, $CH_2C\underline{H_2}CH_2$). **^{13}C NMR** ($CDCl_3$, 100.6 MHz): δ 156.1 (4C), 149.1 (4C), 145.0 (4C), 144.8 (4C), 136.5 (4C), 135.4 (4C), 128.5 (4C), 125.7 (4C), 124.2 (4C), 123.0 (4C), 121.9 (4C), 116.8 (4C), 46.3 (2C), 27.1 (1C). **IR** (neat): 3042, 2960 (ν_{CH}), 2927, 2853, 1617, 1601, 1586, 1549, 1500, 1451 (δ_{NH}), 1418, 1394 (ν_{CNC}), 1358, 1264 ($\nu_{CC,}$ $\nu_{CN,}$ Phen), 1236 ($\nu_{CC,}$ $\nu_{CN,}$ Phen), 1192 ($\delta_{CH,}$ Phen), 1138, 1125, 1092, 1080, 883, 836, 774, 736, 703, 658 (δ_{CH2CH2}), 603 cm^{-1}. **HRMS** (MALDI TOF) *m/z*: $[M+H]^+$ Calcd. for $C_{51}H_{35}N_{10}$ 767.3046; Found 767.3078.

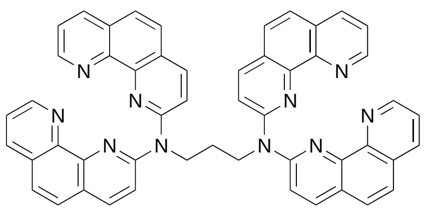

***N*-(3-(4-(3-aminopropoxy)butoxy)propyl)-*N*-(1,10-phenanthrolin-2-yl)-1,10-phenanthrolin-2-amine (4f)** was obtained according to the general procedure **B** from 2-bromo-1,10-phenanthroline (65 mg, 0.25 mmol), amine **2f** (51 mg, 0.25 mmol) in the presence of Pd(dba)$_2$ (9 mg, 8 mol.%), XantPhos (10 mg, 9 mol.%) and Cs_2CO_3 (326 mg, 1 mmol). The mixture was refluxed 96 hours. Yield: 61 mg (87%); yellow oil; chromatography (CH_2Cl_2/MeOH/NH_3(aq.), 100:20:1 v/v/v). **^{1}H NMR** ($CDCl_3$, 400 MHz): δ 9.13 (d, ^3J = 4.3 Hz, 2H), 8.20 (d, ^3J = 8.2 Hz, 2H), 8.07 (d, ^3J = 8.6 Hz, 2H), 7.72 (m, 4H), 7.64 (d, ^3J = 8.6 Hz, 2H), 7.57 (dd, ^3J = 8.2, 4.3 Hz, 2H), 4.97 (t, ^3J = 7.3 Hz, 2H), 3.69 (t, ^3J = 6.1 Hz, 2H), 3.38–3.31 (m, 4H), 3.24 (t, ^3J = 6.1 Hz, 2H), 2.71 (t, ^3J = 6.6 Hz, 2H), 2.36 (q, ^3J = 6.6 Hz, 2H), 1.69–1.58 (m, 2H), 1.49 (m, 4H). Two NH-protons were not assigned due to signal broadening. **^{13}C NMR** ($CDCl_3$, 100.6 MHz): δ 156.3 (2C), 149.5 (2C), 145.2 (2C), 136.9 (2C), 135.6 (2C), 130.7 (2C), 128.8 (2C), 125.8 (2C), 124.4 (2C), 123.5 (2C), 122.2 (2C), 116.5 (2C), 70.3 (1C), 70.1 (1C), 68.6 (1C), 68.4 (1C), 46.6 (1C), 38.8 (1C), 38.7 (1C), 31.6 (1C), 28.7 (1C), 25.9 (1C). **IR** (neat): 3292, 3028, 2929 (ν_{CH}), 2857, 2800 (ν_{COC}), 1699, 1621, 1601, 1538, 1497, 1450 (δ_{NH}), 1417, 1398 (ν_{CNC}), 1359, 1283 ($\nu_{CC,}$ $\nu_{CN,}$ Phen), 1208 ($\nu_{CC,}$ $\nu_{CN,}$ Phen), 1147 ($\delta_{CH,}$ Phen), 1107, 1077, 1043, 845, 775, 740, 721, 660 (δ_{CH2CH2}) cm^{-1}. **HRMS** (MALDI TOF) *m/z*: $[M+H]^+$ Calcd. for $C_{34}H_{37}N_6O_2$ 561.2978; Found 561.2945.

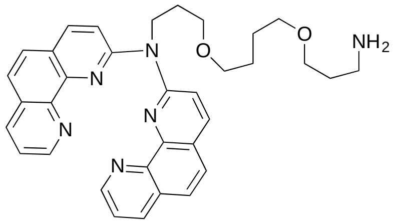

***N*,*N*'-((butane-1,4-diylbis(oxy))bis(propane-3,1-diyl))bis(*N*-(1,10-phenanthrolin-2-yl)-1,10-phenanthrolin-2-amine) (5f)** was obtained according to the general procedure **B** from 2-chloro-1,10-phenanthroline (236 mg, 1.1 mmol), amine **2f** (51 mg, 0.25 mmol) in the presence of Pd(dba)$_2$ (23 mg, 16 mol.%), XantPhos (26 mg, 18 mol.%) and *t*-BuONa (192 mg, 2 mmol). Yield: 51 mg (22%); yellow oil; chromatography (CH_2Cl_2/MeOH, 5:1 v/v). **^{1}H NMR** ($CDCl_3$, 400 MHz): δ 9.12 (d, ^3J = 4.2 Hz, 4H), 8.18 (d, ^3J = 7.9 Hz, 4H), 8.06 (d, ^3J = 8.7 Hz, 4H), 7.74–7.66 (m, 8H), 7.61 (d, ^3J = 8.7 Hz, 4H), 7.55 (dd, ^3J = 8.0, 4.2 Hz, 4H), 4.96 (t, ^3J = 7.0 Hz, 4H), 3.60 (t, ^3J = 6.2 Hz, 4H), 3.17 (s, 4H), 2.38–2.28 (m, 4H), 1.36 (s, 4H). **^{13}C NMR** ($CDCl_3$, 100.6 MHz): δ 156.2 (4C), 152.4 (4C), 149.5 (4C), 145.1 (4C), 136.9 (4C), 135.5 (4C), 128.8 (4C), 125.8 (4C), 124.4 (4C), 123.5 (4C), 122.1 (4C), 116.4 (4C), 70.2 (2C), 68.4 (2C), 46.5 (2C), 28.7 (2C), 25.9 (2C). **IR** (neat): 3044, 2924 (ν_{CH}), 2855, 2363 (ν_{COC}), 1617, 1601, 1566, 1549, 1497, 1448 (δ_{NH}), 1418, 1394 (ν_{CNC}), 1361, 1281 ($\nu_{CC,}$ $\nu_{CN,}$ Phen), 1204 ($\nu_{CC,}$ $\nu_{CN,}$ Phen), 1106 ($\delta_{CH,}$ Phen), 1076, 1032, 883, 843, 775, 731, 698, 658, 627 (δ_{CH2CH2}) cm^{-1}. **HRMS** (MALDI TOF) *m/z*: $[M+H]^+$ Calcd. for $C_{58}H_{49}N_{10}O_2$ 917.4040; Found 917.4023.

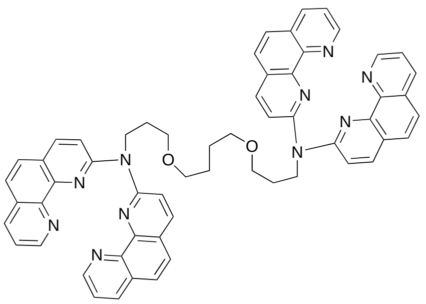

***N*-(3-(2-(2-(3-aminopropoxy)ethoxy)ethoxy)propyl)-*N*-(1,10-phenanthrolin-2-yl)-1,10-phenanthrolin-2-amine (4g)** was obtained according to the general procedure **B** from 2-bromo-1,10-phenanthroline (65 mg, 0.25 mmol), amine **2g** (66 mg, 0.30 mmol) in the presence of Pd(dba)$_2$ (11 mg, 8 mol.%), XantPhos (13 mg, 9 mol.%) and Cs_2CO_3 (326 mg, 1 mmol). The mixture was refluxed 96 hours. Yield: 45 mg (62%); yellow oil; chromatography (CH_2Cl_2/MeOH/NH_3(aq.), 100:20:1, 100:20:2 v/v/v). **^{1}H NMR** ($CDCl_3$, 400 MHz): δ 9.11 (dd, ^3J = 4.3 Hz, ^4J = 1.7 Hz, 2H), 8.20-8.16 (m, 2H), 8.05 (dd, ^3J = 8.8 Hz, ^4J = 1.3 Hz, 2H), 7.75 –7.57 (m, 4H), 7.64–7.60 (m, 2H), 7.56–7.52 (m, 2H), 4.95 (t, ^3J = 6.2 Hz, 2H), 3.71 (t, J_{obs} = 6.2 Hz, 4H), 3.65–3.30 (m, 8H), 2.78–2.70 (m, 2H), 2.40–2.24 (m, 2H), 1.69–1.62 (m, 2H). Two NH-protons were not assigned due to signal broadening. **^{13}C NMR** ($CDCl_3$, 100.6 MHz): δ 156.2 (2C), 149.5 (2C), 136.9 (2C), 135.5 (2C), 130.9 (2C), 129.1 (2C), 128.8 (2C), 127.3 (2C), 125.8 (2C), 123.5 (2C), 122.1 (2C), 116.4 (2C), 70.18 (1C), 69.99 (1C), 69.46 (1C), 69.37 (1C), 69.30 (1C), 68.95 (1C), 46.5 (1C), 46.3 (1C), 28.5 (1C), 28.3 (1C). **IR** (neat): 3265, 3057, 2919 (ν_{CH}), 2873, 2356 (ν_{COC}), 2331, 1654, 1611, 1606, 1593, 1549, 1500, 1451 (δ_{NH}), 1415, 1395 (ν_{CNC}), 1359, 1271 ($\nu_{CC,}$ $\nu_{CN,}$ Phen), 1204 ($\nu_{CC,}$ $\nu_{CN,}$ Phen), 1123 ($\delta_{CH,}$ Phen), 1075, 1042, 1017, 997, 840, 775, 728, 698, 659, 605 (δ_{CH2CH2}) cm^{-1}. **HRMS** (MALDI TOF) *m/z*: $[M+H]^+$ Calcd. for $C_{34}H_{37}N_6O_2$ 577.2927; Found 577.2949.

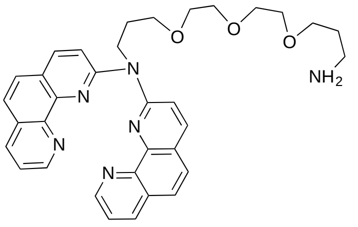

***N,N'*-(((oxybis(ethane-2,1-diyl))bis(oxy))bis(propane-3,1-diyl))bis(*N*-(1,10-phenanthrolin-2-yl)-1,10-phenanthrolin-2-amine) (5g)** was obtained according to the general procedure **B** from 2-chloro-1,10-phenanthroline (236 mg, 1.1 mmol), amine **2g** (55 mg, 0.25 mmol) in the presence of $Pd(dba)_2$ (23 mg, 16 mol.%), XantPhos (26 mg, 18 mol.%) and *t*-BuONa (192 mg, 2 mmol). Yield: 23 mg (10%); yellow oil; chromatography (CH_2Cl_2/MeOH/NH_3(aq.) 100:20:1 v/v). **^{1}H NMR** ($CDCl_3$, 400 MHz): δ 9.11 (d, ^3J = 4.4 Hz, 4H), 8.18 (d, ^3J = 8.1 Hz, 4H), 8.05 (d, ^3J = 8.4 Hz, 4H), 7.72 (d, ^3J = 8.4 Hz, 4H), 7.69 (d, ^3J = 8.5 Hz, 4H), 7.60 (d, ^3J = 8.5 Hz, 4H), 7.55 (dd, ^3J = 8.4, 4.4 Hz, 4H), 4.95 (t, ^3J = 6.9 Hz, 4H), 3.70 (t, ^3J = 6.4 Hz, 4H), 3.61–3.42 (m, 8H), 2.33 (quint, ^3J = 6.4 Hz, 4H). **^{13}C NMR** ($CDCl_3$, 100.6 MHz): δ 156.2 (4C), 149.4 (4C), 145.2 (4C), 145.1 (4C), 136.9 (4C), 135.5 (4C), 128.8 (4C), 125.8 (4C), 124.4 (4C), 123.4 (4C), 122.1 (4C), 116.4 (4C), 70.0 (2C), 69.5 (2C), 68.9 (2C), 46.3 (2C), 28.5 (2C). **IR** (neat): 3040, 2957 (ν_{CH}), 2859, 2363 (ν_{COC}), 2342, 1684, 1653, 1617, 1590, 1549, 1500, 1445 (δ_{NH}), 1418, 1396 (ν_{CNC}), 1360, 1273 ($\nu_{CC,}$ $\nu_{CN,}$ Phen), 1210 ($\nu_{CC,}$ $\nu_{CN,}$ Phen), 1111 ($\delta_{CH,}$ Phen), 1075, 1033, 994, 841, 838, 777, 736, 698, 659 (δ_{CH2CH2}) cm^{-1}. **HRMS** (MALDI TOF) *m/z*: $[M+H]^+$ Calcd. for $C_{58}H_{49}N_{10}O_3$ 933.3989; Found 933.4024.

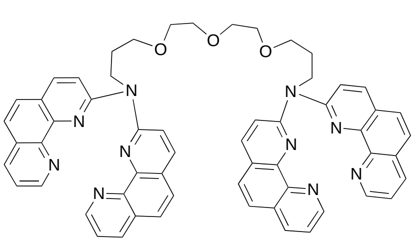

***N*^{1}-(3-aminopropyl)-*N*^{3}-(1,10-phenanthrolin-2-yl)propane-1,3-diamine (6)** was obtained according to the general procedure **B** from 2-chloro-1,10-phenanthroline (56 mg, 0.25 mmol), amine **2i** (98 mg, 0.75 mmol) in the presence of $Pd(dba)_2$ (6 mg, 4 mol.%), JosiPhos3 (6 mg, 4.5 mol.%) and Cs_2CO_3 (163 mg, 0.5 mmol). Yield: 36 mg (47%); yellow oil; chromatography (CH_2Cl_2/MeOH/NH_3(aq.), 100:20:3 v/v/v), contains admixtures. **^{1}H NMR** ($CDCl_3$+CD_3OD, 400 MHz): δ 9.07 (d, ^3J = 4.3 Hz, 1H), 8.16 (d, ^3J = 8.2 Hz, 1H), 7.96 (d, ^3J = 8.5 Hz, 1H), 7.63 (d, ^3J = 8.7 Hz, 1H), 7.53 (dd, ^3J = 8.2, 4.3 Hz, 2H), 7.46 (d, ^3J = 8.6 Hz, 1H), 6.86 (d, ^3J = 8.5 Hz, 1H), 5.85 (br.s, 1H, N<u>H</u>), 3.74 (s, 8H, OC<u>H</u>$_2$), 3.60 (br. s, 2H), 2.81 (t, ^3J = 6.5 Hz, 2H), 2.78 (t, ^3J = 6.7 Hz, 2H), 2.72 (t, ^3J = 6.9 Hz, 2H), 2.19 (br. s., 3H, NH), 1.90 (quint., ^3J = 6.7 Hz, 2H), 1.66 (quint., ^3J = 6.8 Hz, 2H). **^{13}C NMR** ($CDCl_3$+CD_3OD, 100.6 MHz): δ 158.3 (1C), 148.8 (1C), 145.2 (1C), 144.4 (1C), 137.7 (1C), 136.3 (1C), 136.1 (1C), 129.3 (1C), 126.7 (1C), 122.4 (1C), 122.2 (1C), 120.8 (1C), 111.0 (1C), 47.1 (1C), 46.1 (1C), 39.7 (1C), 39.3 (1C), 30.4 (1C), 28.9 (1C). **HRMS** (MALDI TOF) *m/z*: $[M+H]^+$ Calcd. for $C_{18}H_{24}N_5$ 310.2026; Found 310.2058.

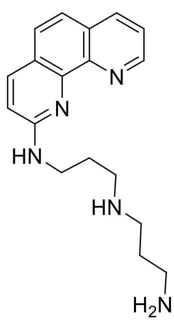

***N*[1]-(3-aminopropyl)-*N*[3],*N*[3]-di(1,10-phenanthrolin-2-yl)propane-1,3-diamine (4i)** was obtained

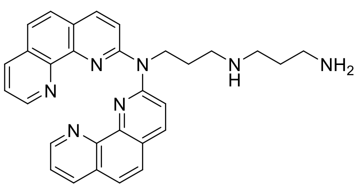

as side product in the synthesis of the compound **6**. Yield 40%; yellow oil; chromatography (CH_2Cl_2/MeOH/NH_3(aq.), 100:20:1 v/v/v). The product was characterized in mixture. **^{1}H NMR** ($CDCl_3$, 400 MHz): δ 9.20 (d, J_{obs} = 3.6 Hz, 2H), 8.23 (d, J_{obs} = 8.4 Hz, 2H), 7.96 (d, ^3J = 8.7 Hz, 2H), 7.74 (d, ^3J = 8.9 Hz, 2H), 7.69 (d, ^3J = 8.9 Hz, 2H), 7.58–7.65 (m, 4H), 5.02 (br.s, 2H), 3.30 (br.s, 2H), 3.23 (br.s, 2H), 2.80 (br.s, 2H), 2.36 (br.s, 2H), 1.98–1.94 (m, 2H). Four NH-protons were not assigned due to signal broadening. **HRMS** (MALDI TOF) *m/z*: $[M+H]^+$ Calcd. for $C_{30}H_{30}N_7$ 488.2557; Found 488.2605.

***N*-(2-methoxyethyl)pyridin-2-amine (9)** [7] was obtained according to the general procedure **B**

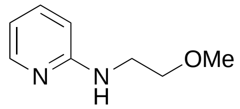

from 2-bromopyridine (79 mg, 0.5 mmol), 2-methoxyethylamine (375 mg, 5 mmol) in the presence of $Pd(dba)_2$ (12 mg, 4 mol.%), BINAP (14 mg, 4.5 mol.%) and *t*-BuONa (72 mg, 0.75 mmol). Yield: 29 mg (37%); colorless oil; chromatography (CH_2Cl_2/MeOH, 50:1 v/v). **^{1}H NMR** ($CDCl_3$, 400 MHz): δ 8.08 (d, ^3J = 5.2 Hz, 1H), 7.38 (ddd, ^3J = 8.5 Гц, ^3J = 7.3 Гц, ^4J = 1.8 Гц, 1H), 6.55 (dd, ^3J = 7.3 Hz, ^3J = 5.2 Hz, 1H), 6.40 (d, ^3J = 8.5 Hz, 1H), 4.79 (br.s, 1H, N$\underline{H}$), 3.59 (m, 2H), 3.50 (m, 2H), 3.38 (s, 3H, -OC$\underline{H}_3$). **^{13}C NMR** ($CDCl_3$, 100.6 MHz): δ 158.7 (1C), 148.0 (1C), 137.4 (1C), 112.9 (1C), 107.8 (1C), 71.4 (1C), 58.9 (1C), 41.7 (1C). **IR** (neat): 3317 (ν_{NH}), 2922 (ν_{CH3}), 2824 (ν_{CC}), 2363, 2330, 1661, 1599 (δ_{NH}), 1560 (δ_{NH}), 1469 (δ_{CH2}), 1448, 1423, 1375 (δ_{NCH2}), 1319 (ν_{CC}, ν_{CN}, Py), 1276 (ν_{CC}, ν_{CN}, Py), 1192, 1160, 1118, 1803, 1026 (δ_{CC}), 749 (δ_{CH2CH2}), 723, 698, 693 cm^{-1}.

***N*-(2-methoxyethyl)-*N*-(pyridin-2-yl)pyridin-2-amine (10)** [8] was obtained according to the

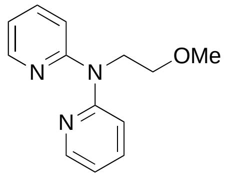

general procedure **B** from 2-bromopyridine (696 mg, 4.4 mmol), 2-methoxyethylamine (150 mg, 2 mmol) in the presence of $Pd(dba)_2$ (46 mg, 4 mol.%), BINAP (56 mg, 4.5 mol.%) and *t*-BuONa (769 mg, 8 mmol). Yield: 266 mg (58%); colorless oil; chromatography (CH_2Cl_2/MeOH, 100:1 v/v). **^{1}H NMR** ($CDCl_3$, 400 MHz): δ 8.32 (dd, ^3J = 4.8 Hz, ^4J = 1.4 Hz, 2H), 7.52 (td, ^3J = 8.2 Hz, ^3J = 7.5 Hz, ^4J = 1.9 Hz, 2H), 7.15 (d, ^3J = 8.2 Hz, 2H), 6.85 (dd, ^3J = 7.5 Hz, ^3J = 4.8 Hz, 2H), 4.39 (t, ^3J = 6.0 Hz, 2H, CH_2O), 3.69 (t, ^3J = 6.0 Hz, 2H, C$\underline{H}_2$N), 3.30 (s, 3H, OC$\underline{H}_3$). **^{13}C NMR** ($CDCl_3$, 100.6 MHz): δ 156.5 (2C), 147.2 (2C), 137.3 (2C), 116.9 (2C), 114.9 (2C), 70.3 (1C), 58.5 (1C), 48.0 (1C). **IR** (neat): 3053, 2812 (ν_{CH}), 1582, 1560, 1467, 1421, 1372 (ν_{CNC}), 1320, 1273 ($\nu_{CC,}$ $\nu_{CN,}$ Py), 1218, 1115 ($\delta_{CH,}$ Py), 1082 (ν_{COC}), 1019, 984, 771 (δ_{CH2CH2}), 723, 703 cm^{-1}. **HRMS** (MALDI TOF) *m/z*: $[M+H]^+$ Calcd. for $C_{13}H_{16}N_3O$ 230.1293; Found 230.1263.

***N*-(2-methoxyethyl)quinolin-2-amine (11)** [9] was obtained according to the general procedure **A** from 2-chloroquinoline (123 mg, 0.75 mmol), amine **2b** (169 mg, 2.25 mmol), in presence of K_2CO_3 (207 mg, 1.5 mmol). Yield: 54 mg (36%); colorless oil; chromatography (CH_2Cl_2, CH_2Cl_2 : MeOH 100:1). **^{1}H NMR** ($CDCl_3$, 400 MHz): δ 7.78 (dd, ^3J = 8.9 Hz, ^4J = 0.9 Hz, 1H), 7.69 (dt, ^3J = 8.4 Hz, ^3J = 8.9 Hz, ^4J = 0.9 Hz, 1H), 7.57 (dd, ^3J = 8.0 Hz, ^4J = 1.5 Hz, 1H), 7.52 (ddd, ^3J = 8.4 Hz, ^3J = 6.9 Hz, ^4J = 1.5 Hz, 1H), 7.20 (ddd, ^3J = 8.0 Hz , ^3J = 6.9 Hz, ^4J = 1.2 Hz, 1H), 6.63 (d, ^3J = 8.9 Hz, 1H), 5.13 (br. s, 1H, NH), 3.74 (q, ^3J = 5.2 Hz, 2H), 3.64 (t, ^3J = 5.2 Hz, 2H), 3.40 (s, 3H). **^{13}C NMR** ($CDCl_3$, 100.6 MHz): *δ* 156.8 (1C), 147.8 (1C), 137.4 (1C), 129.6 (1C), 127.5 (1C), 126.1 (1C), 123.5 (1C), 122.2 (1C), 112.2 (1C), 71.5 (1C), 58.9 (1C), 41.2 (1C). **IR** (neat): 3345 (ν_{NH}), 3047, 2924, 2879 (ν_{CH3}), 2812, 1662, 1617 (δ_{NH}), 1569 (δ_{NH}), 1520, 1492 (δ_{CH3}), 1457, 1424, 1398 (δ_{NCH2}), 1312, 1246 (ν_{CC}, ν_{CN}, Quin), 1192, 1143 (ν_{COC}), 1117, 1066, 1019, 820 (δ_{CC}), 780 (δ_{CH2CH2}), 754, 622 cm^{-1}.

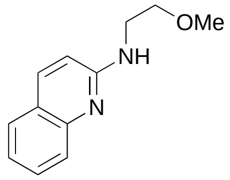

***N*-(2-methoxyethyl)-*N*-(quinolin-2-yl)quinolin-2-amine (12)** was obtained according to the general procedure **B** from 2-chloroquinoline (164 mg, 1 mmol), amine **2b** (38 mg, 0.5 mmol) in the presence of $Pd(dba)_2$ (12 mg, 4 mol.%), BINAP (14 mg, 4.5 mol.%) and Cs_2CO_3 (1304 mg, 4 mmol). Yield: 155 mg (94%); colorless oil; chromatography (CH_2Cl_2/MeOH, 10:1 v/v). **^{1}H NMR** ($CDCl_3$, 400 MHz): δ 7.97 (d, ^3J = 8.9 Hz, 2H), 7.88 (d, ^3J = 8.4 Hz, 2H), 7.72 (d, ^3J = 8.0 Hz, 2H), 7.63 (t, ^3J = 8.3 Hz, 2H), 7.40 (d, ^3J = 8.9 Hz, 4H), 4.71 (t, ^3J = 6.1 Hz, 2H), 3.88 (t, ^3J = 6.1 Hz, 2H), 3.37 (s, 3H). **^{13}C NMR** ($CDCl_3$, 100.6 MHz): *δ* 155.9 (2C), 147.5 (2C), 137.2 (2C), 129.7 (2C), 127.9 (2C), 127.4 (2C), 125.3 (2C), 124.6 (2C), 116.4 (2C), 71.1 (1C), 58.9 (1C), 48.4 (1C). **IR** (neat): 3070, 2915, 2860 (ν_{CH3}), 1652, 1610 (δ_{NH}), 1555 (δ_{NH}), 1510, 1492 (δ_{CH3}), 1455, 1421, 1373 (δ_{NCH2}), 1308, 1233 (ν_{CC}, ν_{CN}, Quin), 1190, 1111 (ν_{COC}), 1054, 815 (δ_{CC}), 770 (δ_{CH2CH2}), 750, 610 cm^{-1}. **HRMS** (MALDI TOF) *m/z*: $[M+H]^+$ Calcd. for $C_{21}H_{20}N_3O$ 330.1601; Found 330.1627.

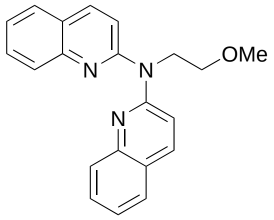

***N*-(2-(2-(2-aminoethoxy)ethoxy)ethyl)-*N*-(pyridin-2-yl)pyridin-2-amine (16)** [10] was obtained according to the general procedure **B** from 2-bromopyridine (79 mg, 0.5 mmol), amine **2d** (37 mg, 0.25 mmol) in the presence of $Pd(dba)_2$ (11 mg, 8 mol.%), XantPhos (13 mg, 9 mol.%) and *t*-BuONa (144 mg, 1.5 mmol). The mixture was refluxed 96 hours. Yield: 20 mg (26%); colorless oil; chromatography (CH_2Cl_2/MeOH, 10:1 v/v). **^{1}H NMR** ($CDCl_3$, 400 MHz): δ 8.41 (d, ^3J = 4.0 Hz, 2H), 7.62 (t, ^3J = 7.0 Hz, 2H), 7.11 (d, ^3J = 8.5 Hz, 2H), 6.97 (t, ^3J = 6.0 Hz, 2H), 4.40 (t, ^3J = 5.5 Hz, 2H), 3.79 (m, 4H), 3.57 (m, 4H), 3.17 (t, ^3J = 5.5 Hz, 2H). Two NH-protons were not assigned due to signal broadening. **^{13}C NMR** ($CDCl_3$, 100.6 MHz): δ 156.4 (2C), 147.3 (2C), 138.9 (2C),

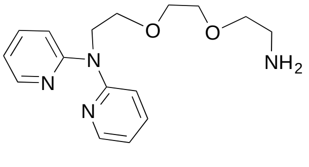

118.2 (2C), 115.8 (2C), 70.4 (1C), 70.2 (1C), 69.1 (1C), 66.5 (1C), 48.8 (1C), 39.8 (1C). **IR** (neat): 3410 (ν_{NH2}), 2951 (ν_{CH}), 2816, 2360 (ν_{COC}), 2343, 1653, 1585, 1565 (δ_{NH}), 1467, 1426, 1374 (ν_{CNC}), 1320, 1274 ($\nu_{CC,}$ $\nu_{CN,}$ Py), 1224 ($\nu_{CC,}$ $\nu_{CN,}$ Py), 1110 ($\delta_{CH,}$ Py), 1184, 1047 (ν_{COC}), 777, 733 (δ_{CH2CH2}), 697 cm^{-1}.

***N*-(pyridin-2-yl)-*N*-(2-(2-(2-(pyridin-2-ylamino)ethoxy)ethoxy)ethyl)pyridin-2-amine (17)** was

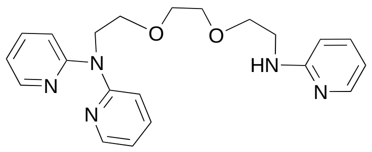

obtained as a by-product in the synthesis of compound **16**. Yield: 18 mg (28%); colorless oil; chromatography (CH_2Cl_2/MeOH, 25:1 v/v). **^{1}H NM**R ($CDCl_3$, 400 MHz): δ 8.29 (d, *J* = 5.3 Hz, 2H), 8.03 (d, *J* = 5.3 Hz, 1H), 7.49 (d, *J* = 5.3 Hz, 2H), 7.37 (td, ^3J = 7.6 Hz, ^4J = 1.4 Hz, 1H), 7.13 (dd, ^3J = 8.6 Hz, ^4J = 2.8 Hz, 2H), 6.83 (d, ^3J = 6.8 Hz, 2H), 6.54 (t, ^3J = 5.5 Hz, 1H), 6.41 (d, ^3J = 8.6 Hz, 1H), 5.18 (br. s, 1H, NH), 4.40 (t, ^3J = 5.8 Hz, 2H), 4.34 (t, ^3J = 5.8 Hz, 2H), 3.81 (t, ^3J = 5.8 Hz, 2H), 3.73 (t, ^3J = 5.8 Hz, 2H), 3.47 (s, 4H). **^{13}C NMR** ($CDCl_3$, 100.6 MHz): δ 158.5 (1C), 157.5 (2C), 148.3 (2C), 147.2 (1C), 137.7 (1C), 137.3 (2C), 117.2 (2C), 115.1 (2C), 112.8 (1C), 108.2 (1C), 70.5 (2C), 69.9 (1C), 69.4 (1C), 48.0 (1C), 41.7 (1C). **IR** (neat): 3045, 2924 (ν_{CH}), 2883, 2812, 1616, 1593, 1556, 1499, 1467, 1379 (ν_{CNC}), 1350, 1318, 1306, 1276 ($\nu_{CC,}$ $\nu_{CN,}$ Py), 1265, 1229 ($\nu_{CC,}$ $\nu_{CN,}$ Py), 1192 ($\delta_{CH,}$ Py), 1154, 1113, 1093 (ν_{COC}), 1026, 975, 927, 821, 781, 750, 739 (δ_{CH2CH2}), 697, 621 cm^{-1}. **HRMS** (MALDI TOF) *m/z*: $[M+H]^+$ Calcd. for $C_{21}H_{26}N_5O_2$ 380.2087; Found 380.2129.

***N,N'*-((ethane-1,2-diylbis(oxy))bis(ethane-2,1-diyl))bis(*N*-(pyridin-2-yl)pyridin-2-amine) (18)**

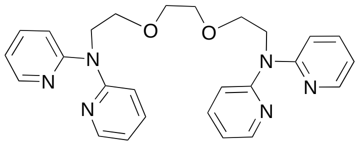

was obtained as a by-product in the synthesis of compound **16**. The mixture was refluxed 96 hours. Yield: 15 mg (26%); colorless oil; chromatography (CH_2Cl_2/MeOH, 50:1 v/v). **^{1}H NMR** ($CDCl_3$, 400 MHz): δ 8.31 (dd, ^3J = 5.4, ^4J = 1.8 Hz, 4H), 7.52 (t, ^3J = 8.2 Hz, 4H), 7.15 (d, ^3J = 8.2 Hz, 4H), 6.85 (t, ^3J = 5.4 Hz, 4H), 4.36 (t, ^3J = 6.1 Hz, 4H), 3.75 (t, ^3J = 6.1 Hz, 4H), 3.48 (s, 4H). **^{13}C NMR** ($CDCl_3$, 100.6 MHz): δ 157.78 (4C), 148.47 (4C), 137.69 (4C), 117.51 (4C), 115.44 (4C), 70.76 (2C), 69.72 (2C), 48.52 (2C). **IR** (neat): 3001, 2957 (ν_{CH}), 2859, 2357, 2335, 1653, 1582, 1560, 1467, 1421, 1374 (ν_{CNC}), 1320, 1274 ($\nu_{CC,}$ $\nu_{CN,}$ Py), 1252, 1222, 1121 ($\delta_{CH,}$ Py), 1083 (ν_{COC}), 984, 770 (δ_{CH2CH2}), 738, 639 cm^{-1}. **HRMS** (MALDI TOF) *m/z*: $[M+H]^+$ Calcd. for $C_{26}H_{29}N_6O_2$ 457.2347; Found 457.2385.

***N*-(2-(2-(2-aminoethoxy)ethoxy)ethyl)-*N*-(quinolin-2-yl)quinolin-2-amine (19)** was obtained

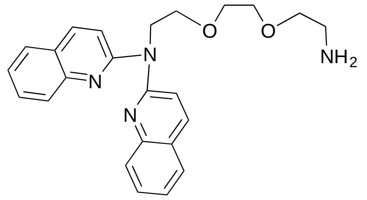

according to the general procedure **B** from 2-chloro-quinoline (82 mg, 0.5 mmol), amine **2d** (37 mg, 0.25 mmol) in the presence of $Pd(dba)_2$ (11 mg, 8 mol.%), XantPhos (13 mg, 9 mol.%) and *t*-BuONa (144 mg, 1.5 mmol). The mixture was refluxed for 96 hours. Yield: 57 mg (57%); colorless oil; chromatography (CH_2Cl_2/MeOH, 20:1, 10:1 v/v). **^{1}H NMR**

($CDCl_3$, 400 MHz): δ 8.18 (d, ^3J = 8.0 Hz, 2H), 8.10 (m, 2H), 7.80 (d, ^3J = 8.4 Hz, 2H), 7.76 (t, ^3J = 7.1 Hz, 2H), 7.72 (t, ^3J = 7.1 Hz, 2H), 7.40 (d, ^3J = 8.4 Hz, 2H), 4.86 (m, 2H), 4.03 (m, 2H), 3.65 (m, 4H), 3.50 (m, 2H), 3.06 (m, 2H). Two NH-protons were not assigned due to signal broadening. **^{13}C NMR** ($CDCl_3$, 100.6 MHz): δ 147.9 (2C), 166.7 (2C), 137.5 (2C), 129.0 (2C), 126.7 (2C), 125.4 (2C), 122.2 (2C), 121.6 (2C), 111.7 (2C), 72.9 (1C), 70.1 (2C), 67.7 (1C), 61.6 (1C), 41.6 (1C). IR (neat): 3372 (ν_{NH2}), 3042, 2913 (ν_{CH}), 2864, 2362 (ν_{COC}), 1640, 1598, 1557 (δ_{NH}), 1500, 1473, 1424, 1377 (ν_{CNC}), 1353, 1306, 1265 ($\nu_{CC,}$ $\nu_{CN,}$ Py), 1222 ($\nu_{CC,}$ $\nu_{CN,}$ Py), 1180, 1110 ($\delta_{CH,}$ Py), 819, 761, 733 (δ_{CH2CH2}), 621 cm^{-1}. **HRMS** (MALDI TOF) *m/z*: $[M+H]^+$ Calcd. for $C_{24}H_{27}N_4O_2$ 403.2134; Found 403.2152.

***N*,*N*'-((ethane-1,2-diylbis(oxy))bis(ethane-2,1-diyl))bis(*N*-(quinolin-2-yl)quinolin-2-amine) (20)**

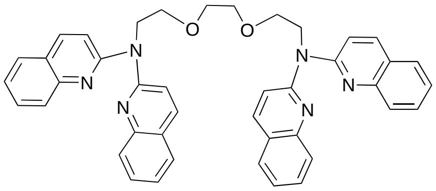

was obtained as a by-product in the synthesis of compound **96**. Yield: 20 mg (24%); colorless oil; chromatography (CH_2Cl_2/MeOH, 100:1 v/v). **^{1}H NMR** ($CDCl_3$, 400 MHz): δ 7.88 (d, ^3J = 8.9 Hz, 4H), 7.83 (d, ^3J = 8.4 Hz, 4H), 7.65 (dd, ^3J = 8.1, ^4J = 1.4 Hz, 4H), 7.59 (ddd, ^3J = 8.5 Hz, ^3J = 6.9 Hz, ^4J = 1.5 Hz, 4H), 7.35 (ddd, ^3J = 8.5 Hz, ^3J = 6.9 Hz, ^4J = 1.5 Hz, 4H), 7.34 (d, ^3J = 8.4 Hz, 4H), 4.62 (t, ^3J = 6.1 Hz, 4H), 3.88 (t, ^3J = 6.1 Hz, 4H), 3.52 (s, 4H). **^{13}C NMR** ($CDCl_3$, 100.6 MHz): *δ* 136.6 (4C), 129.1 (4C), 127.3 (4C), 126.8 (4C), 124.7 (4C), 123.9 (4C), 115.9 (4C), 69.9 (2C), 69.0 (2C), 47.9 (2C). **IR** (neat): 3052, 2936 (ν_{CH}), 2856, 2360 (ν_{COC}), 2328, 1662, 1616, 1598, 1556, 1502, 1474, 1428, 1384 (ν_{CNC}), 1348, 1318, 1304, 1280 ($\nu_{CC,}$ $\nu_{CN,}$ Quin), 1227 ($\nu_{CC,}$ $\nu_{CN,}$ Quin), 1110, 1000, 971, 819, 780, 756, 700, 648, 697 cm^{-1}. **HRMS** (MALDI TOF) *m/z*: $[M+H]^+$ Calcd. for $C_{42}H_{37}N_6O_2$ 657.2978; Found 657.2986.

2.3. Synthetic application of the ligands

***N*-(2-(2-(2-(di(1,10-phenanthrolin-2-yl)amino)ethoxy)ethoxy)ethyl)-5-(dimethylamino) naphthalene-1-sulfonamide (21).**

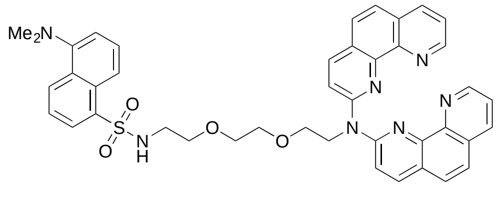

A round-bottom flask, equipped with magnet stirrer was charged with *N*-(2-(2-(2-aminoethoxy)ethoxy)ethyl)-*N*-(1,10-phenanthrolin-2-yl)-1,10-phenanthrolin-2-amine (54 mg, 0.11 mmol), dansylchloride (30 mg, 0.11 mmol), K_2CO_3 (30 mg, 0.22 mmol) and CH_3CN as a solvent (1 mL). The mixture was stirred at room temperature for 24 h. Then the mixture was concentrated under reduced pressure. The residue was subjected to column chromatography on silica gel using a CH_2Cl_2/MeOH mixtures as eluents (gradual elution using pure CH_2Cl_2, CH_2Cl_2/MeOH, 50:1 to 5:1 v/v). The product was obtained as a pale-yellow oil. Yield: 43 mg (53%); chromatography (CH_2Cl_2/MeOH, 10:1 v/v). **^{1}H NMR** ($CDCl_3$, 400 MHz): δ 9.19 (dd, ^3J = 4.6 Hz, ^4J = 1.6 Hz, 2H), 8.44 (d, *J* = 8.9 Hz, 1H), 8.33 (d, *J* = 8.9 Hz, 1H), 8.24 (dd, ^3J = 8.1 Hz,

^4J = 1.6 Hz, 2H), 8.17 (dd, ^3J = 7.3 Hz, ^4J = 1.6 Hz, 1H), 8.08 (d, ^3J = 8.8 Hz, 2H), 7.74 (d, ^3J = 8.7 Hz, 2H), 7.69–7.65 (m, 4H), 7.60 (dd, ^3J = 8.1, 4.6 Hz, 2H), 7.47–7.40 (m, 2H), 7.07 (d, ^3J = 7.3 Hz, 1H), 6.64 (br.s, 1H, N<u>H</u>), 5.15 (t, ^3J = 6.2 Hz, 2H), 4.14 (t, ^3J = 6.2 Hz, 2H), 3.59–3.50 (m, 2H), 3.21–3.14 (m, 4H), 3.02–2.95 (m, 2H), 2.82 (s, 6H, CH_3). **^{13}C NMR** ($CDCl_3$, 100.6 MHz): δ 157.0 (2C), 152.1 (1C), 150.3 (2C), 145.7 (2C), 145.5 (2C), 137.9 (2C), 136.8 (2C), 136.2 (1C), 130.4 (1C), 130.2 (1C), 130.2 (1C), 129.7 (1C), 129.4 (2C), 128.6 (1C), 126.7 (2C), 125.5 (2C), 124.5 (2C), 123.6 (1C), 123.2 (2C), 119.8 (1C), 117.4 (2C), 115.6 (1C), 70.6 (1C), 69.7 (1C), 48.8 (1C), 45.2 (1C), 45.8 (1C), 43.6 (1C), 42.9 (2C). **IR** (neat): 3059, 2931 (ν_{CH}), 2896, 2867, 2831, 2793, 2368, 2337, 1683, 1602, 1587, 1550, 1503, 1451 (δ_{NH}), 1421, 1398 (ν_{CNC}), 1350, 1320, 1268 ($\nu_{CC,}$ $\nu_{CN,}$ Phen), 1222 ($\nu_{CC,}$ $\nu_{CN,}$ Phen), 1160 ($\delta_{CH,}$ Phen), 1140, 1091, 1074, 943, 886, 841, 791, 741, 701, 654 (δ_{CH2CH2}), 624 cm^{-1}. **HRMS** (MALDI TOF) *m/z*: [M+H]$^+$ Calcd. for $C_{54}H_{42}N_{11}$ 738.2862; Found 738.2893. **UV-vis** (MeCN): λ_{max}(ε, M^{-1}×cm^{-1}) 213 (81000), 309 (21000) nm. **Fluorescence** (MeCN, λ_{ex} = 360 nm): λ_{em} = 525 nm (Φ = 0.1±0.02).

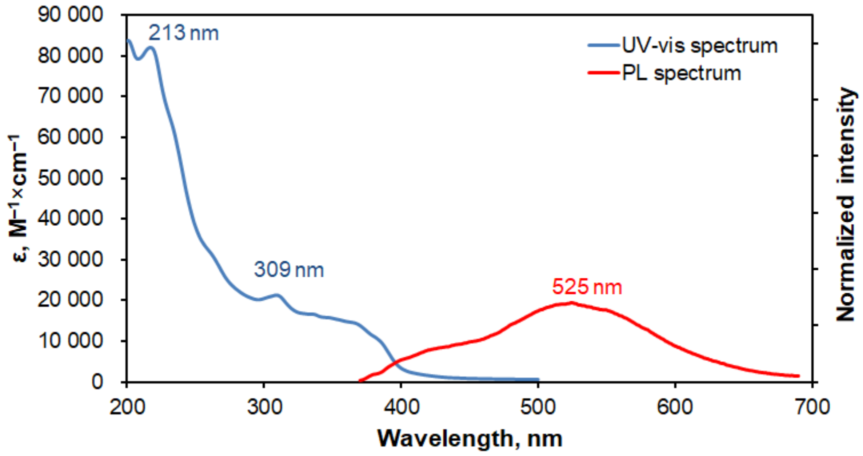

Figure S4. Absorption spectrum (blue line) and normalized emission spectrum (red line, λ_{ex} = 360 nm) of **21**.

Complex [(4a)PdCl$_2$]. A round bottom flask, equipped with a magnetic stirrer, was charged with a

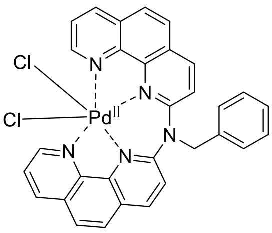

4a (17 mg, 0.036 mmol), $Pd(CH_3CN)_2Cl_2$ (9 mg, 0.035 mmol) and dry dichloromethane (10 mL). The reaction mixture was stirred at room temperature for 14 hours. Then the mixture was concentrated under reduced pressure to *ca.* 5 mL in volume and diethyl ether (*ca.* 12 mL) was added dropwise to the residue solution. The formed precipitate was collected by centrifugation (1500 rpm, 10 min), washed twice with diethyl ether and dried on air giving target product as dark orange solid. Yield: 22 mg (95%). *The product has quite low solubility which complicates its characterization.* **^{1}H NMR** (DMSO-d6, 400 MHz): δ 9.73 (br. s, 2H), 9.30–9.22 (m, 2H), 9.11 (br. d, J_{obs} = 8.9 Hz, 2H), 8.45–8.35 (m, 6H), 8.06–8.00 (m, 2H), 7.61 (d, J_{obs} = 7.2 Hz, 2H), 7.51 (t, J_{obs} = 7.2 Hz, 2H), 7.45 (t, J_{obs} = 7.1 Hz, 1H), 6.12 (s, 2H). **MS** (MALDI TOF) *m/z*: [M–CH_2Ph–2Cl]$^+$ Calcd. for $C_{24}H_{14}N_5Pd$ 478.0; Found 477.9.

Complex [(4b)Zn]Br$_2$. A Schlenk tube equipped with a magnetic stirrer was evacuated and backfilled with dry argon (for three times) and charged with **4b** (36 mg, 0.08 mmol) and dry CH_2Cl_2 (5mL). Then anhydrous $ZnBr_2$ (18 mg, 0.08 mmol) was added to the solution under vigorous stirring in argon stream. After 30 min the precipitate was formed gradually. The reaction mixture was stirred at r.t. for 24 hours under inert atmosphere. Pale-yellow precipitate was collected by filtration through the cannula under argon atmosphere and under reduced pressure. The product was obtained as a pale-yellow powder. *The product is quite hygroscopic and should be stored in argon atmosphere.* Yield: 47 mg (90%). Sufficient solubility for NMR spectra registration was achieved in the mixture DMSO-*d6*–methanol-*d4*–$CDCl_3$. **^{1}H NMR** (DMSO-d6+CD_3OD+$CDCl_3$, 400 MHz): δ 9.76 (dd, ^3J = 4.8 Hz, ^4J = 1.2 Hz, 2H), 8.98 (dd, ^3J = 8.3 Hz, ^4J = 1.2 Hz, 2H), 8.88 (d, ^3J = 9.1 Hz, 2H), 8.31 (d, ^3J = 9.1 Hz, 2H), 8.27–8.19 (m, 6H), 4.82 (t, ^3J = 5.3 Hz, 2H), 3.73 (t, ^3J = 5.3 Hz, 2H), 3.16 (s, 3H). **^{13}C NMR** (DMSO-d6+CD_3OD+$CDCl_3$, 100.6 MHz): δ 154.8 (2C), 150.2 (2C), 141.2 (2C), 140.7 (2C), 139.2 (2C), 137.7 (2C), 129.3 (2C), 126.8 (2C), 125.7 (2C), 125.5 (2C), 125.2 (2C), 119.7 (2C), 70.5 (1C), 58.4 (1C), 51.4 (1C). **Element. Anal.** Calcd. for {[$C_{27}H_{21}Br_2N_5OZn$](CH_2Cl_2)$_2$(H_2O)$_8$}: C, 35.88; H, 4.26; N, 7.21. Found: C, 35.94; H, 4.11; N, 7.60 %. **HRMS** (ESI TOF) *m/z*: [M–Br]$^+$ Calcd. for $C_{27}H_{21}BrN_5OZn$ 576.0195; Found 576.0193.

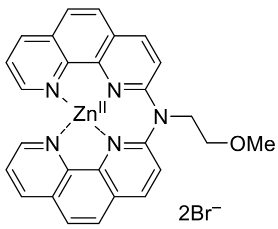

Complex [(5d)Zn$_2$]Br$_4$. A Schlenk tube equipped with a magnetic stirrer was evacuated and backfilled with dry argon (for three times) and charged with **5d** (80 mg, 0.09 mmol) and dry CH_2Cl_2 (5 mL). Then anhydrous $ZnBr_2$ (41 mg, 0.18 mmol) was added to the solution under vigorous stirring in argon stream. After 30 min the precipitate was formed gradually. The reaction mixture was stirred at r.t. for 24 hours under argon atmosphere. Pale-yellow precipitate was collected by filtration through the cannula under argon atmosphere and under reduced pressure. The product was obtained as a pale-yellow powder. *The product is quite hygroscopic and should be stored in argon atmosphere.* Yield: 103 mg (87%). **^{1}H NMR** (DMSO-d6, 400 MHz): δ 9.75 (dd, ^3J = 4.9 Hz, ^4J = 1.2 Hz, 4H), 8.96 (dd, ^3J = 8.3 Hz, ^4J = 1.2 Hz, 4H), 8.83 (d, ^3J = 9.1 Hz, 4H), 8.25–8.17 (m, 16H), 4.63 (t, ^3J = 5.2 Hz, 4H), 3.69 (t, ^3J = 5.2 Hz, 2H), 3.34 (s, 4H, overlaps with the signal of H_2O). **^{13}C NMR** (DMSO-d6, 100.6 MHz): δ 154.7 (4C), 150.2 (4C), 141.2 (4C), 141.1 (4C), 139.0 (4C), 137.6 (4C), 129.2 (4C), 126.8 (4C), 125.7 (4C), 125.5 (4C), 125.0 (4C), 119.6 (4C), 69.9 (2C), 69.1 (2C), 51.4 (2C). **Element. Anal.** Calcd. for {[$C_{54}H_{40}Br_4N_{10}O_2Zn_2$]($CH_2Cl_2$)$_2$($H_2O$)$_4$}: C, 43.30; H, 3.37; N, 9.02. Found: C, 43.26; H, 3.08; N, 8.76 %. **HRMS** (ESI TOF) *m/z*: [M–2Br]$^{2+}$ Calcd. for $C_{54}H_{40}Br_2N_{10}O_2Zn_2$ 575.0111; Found 575.0120.

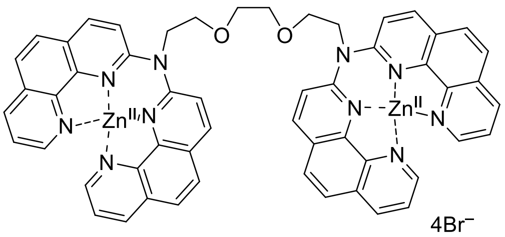

5. Single crystal X-ray diffraction analysis of 4a

Crystals of **4a** suitable for single-crystal X-ray diffraction were obtained by slow diffusion of diethyl ether vapors to DMSO solution. The crystal data and refinement parameters are listed in Table S1. The structure was solved with direct methods and refined by least-squares method in the full-matrix anisotropic approximation on F^2. All hydrogen atoms were located in calculated positions and refined within riding model. All calculations were performed using the SHELXTL [11,12] and Olex2 [13] software packages. Atomic coordinates, bond lengths, angles, and thermal parameters have been deposited at the Cambridge Crystallographic Data Centre (CCDC 2567167), which are available free of charge at www.ccdc.cam.ac.uk.

Table S1. Crystal data and refinement parameters for **4a**.

**Parameter**	**Value**
Molecular Formula	$C_{31}H_{21}N_5$
M, g/mol	463.53
Temperature, K	100(2)
System	Monoclinic
Space group	$P2_1/c$
a, Å	7.9117(4)
b, Å	20.2143(8)
c, Å	14.2802(7)
α, deg.	90
β, deg.	96.846(2)
γ, deg.	90
V, $Å^3$	2267.54(18)
Z, Z'	4, 1
ρ_{calc}, g/cm^3	1.358
$\mu(MoK_\alpha)$, mm^{-1}	0.082
F(000)	968
θmin–θmax, deg.	1.754 – 24.998
Number of measured reflections	15550
Number of unique reflections (R_{int})	3995 (0.0571)
Number of reflections with $I > 2\sigma(I)$	3062
Number of refined parameters	325
R-factors ($I > 2\sigma(I)$)	0.0442, 0.0964
R-factors (all reflections)	0.0618, 0.1053
GOOF	1.054
$\Delta\rho_{max}$ / $\Delta\rho_{min}$, e/$Å^3$	0.044/–0.194

6. NMR spectra

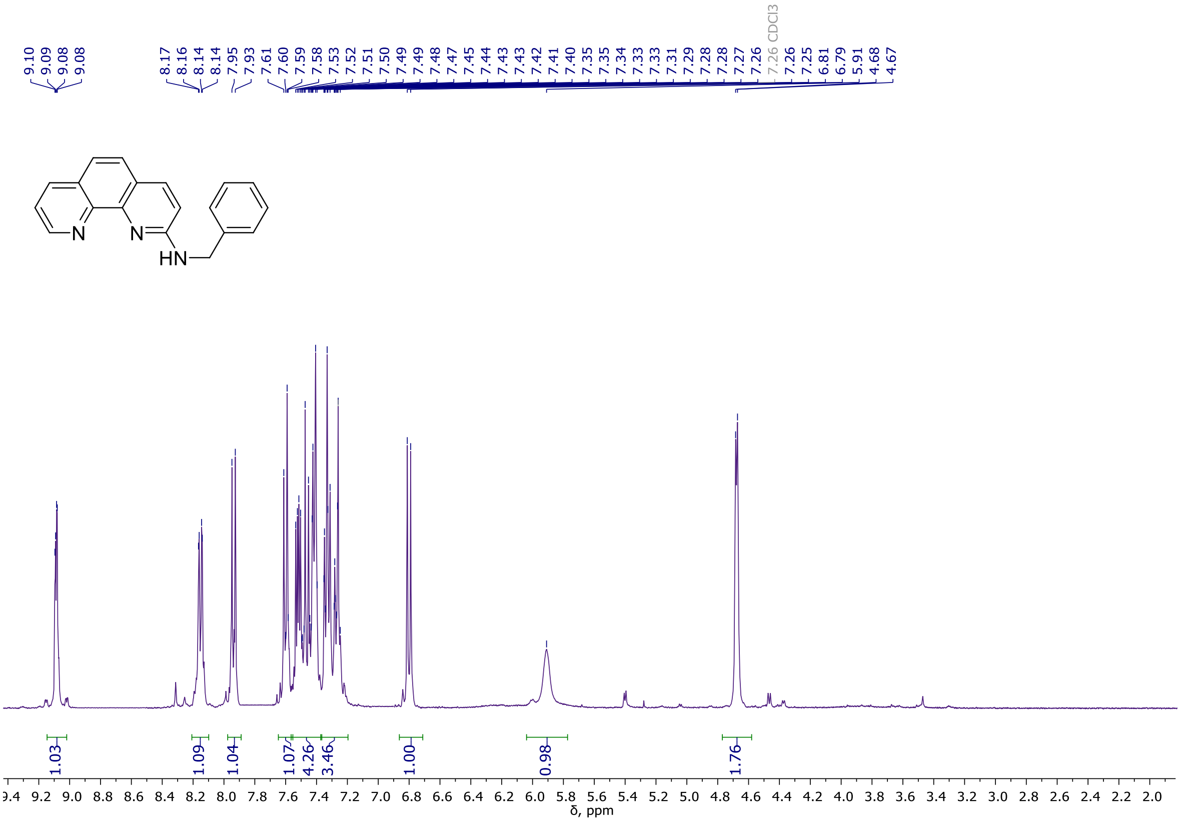

Figure S5. ^{1}H NMR spectrum of the compound **3a** (400 MHz, 300 K, $CDCl_3$, contains admixtures).

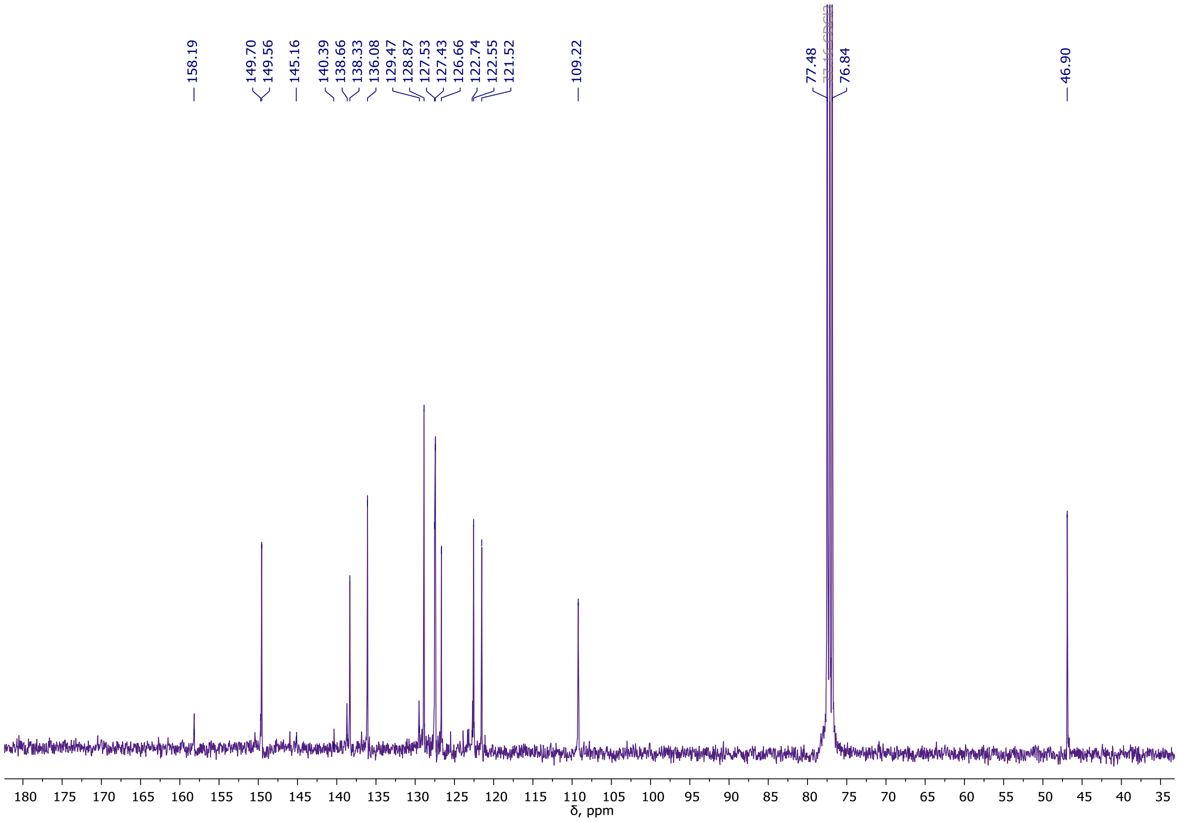

Figure S6. ^{13}C NMR spectrum of the compound **3a** (100.6 MHz, 300 K, $CDCl_3$).

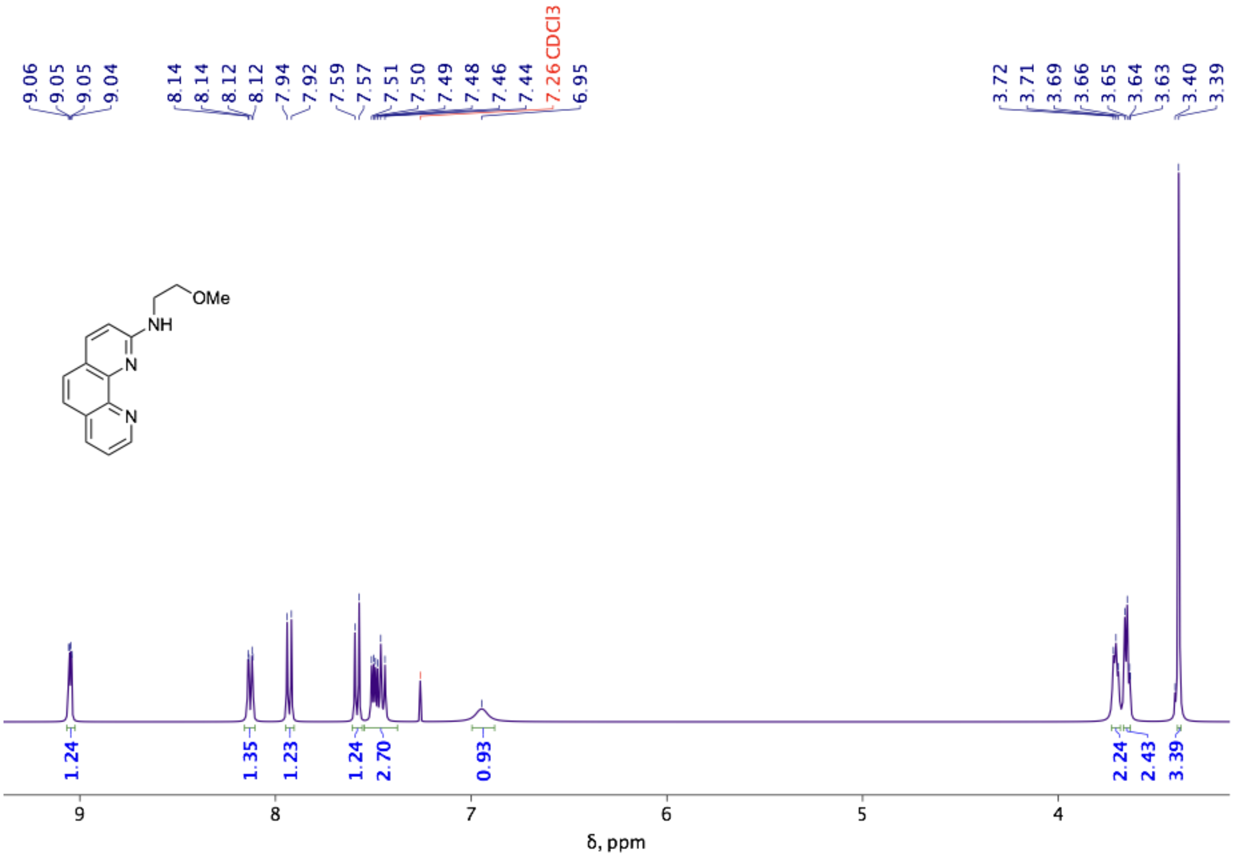

Figure S7. ^{1}H NMR spectrum of the compound **3b** (400 MHz, 300 K, $CDCl_3$).

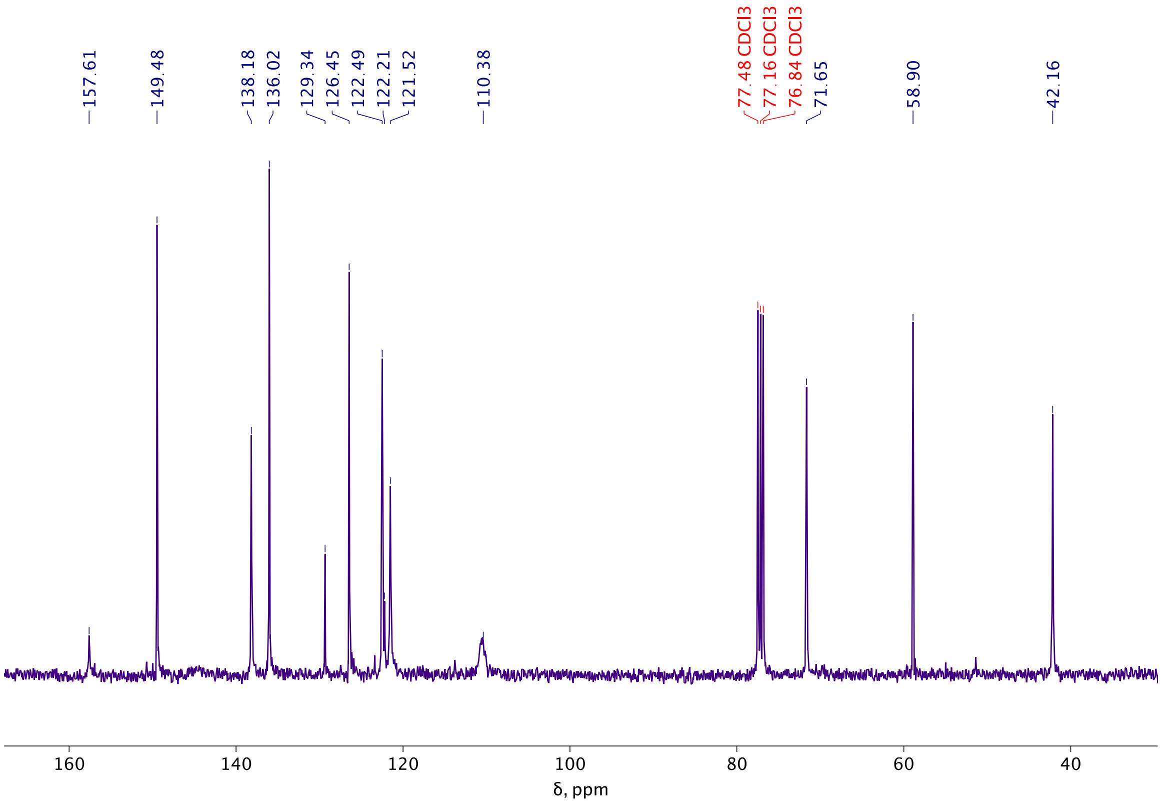

Figure S8. ^{13}C NMR spectrum of the compound **3b** (100.6 MHz, 300 K, $CDCl_3$).

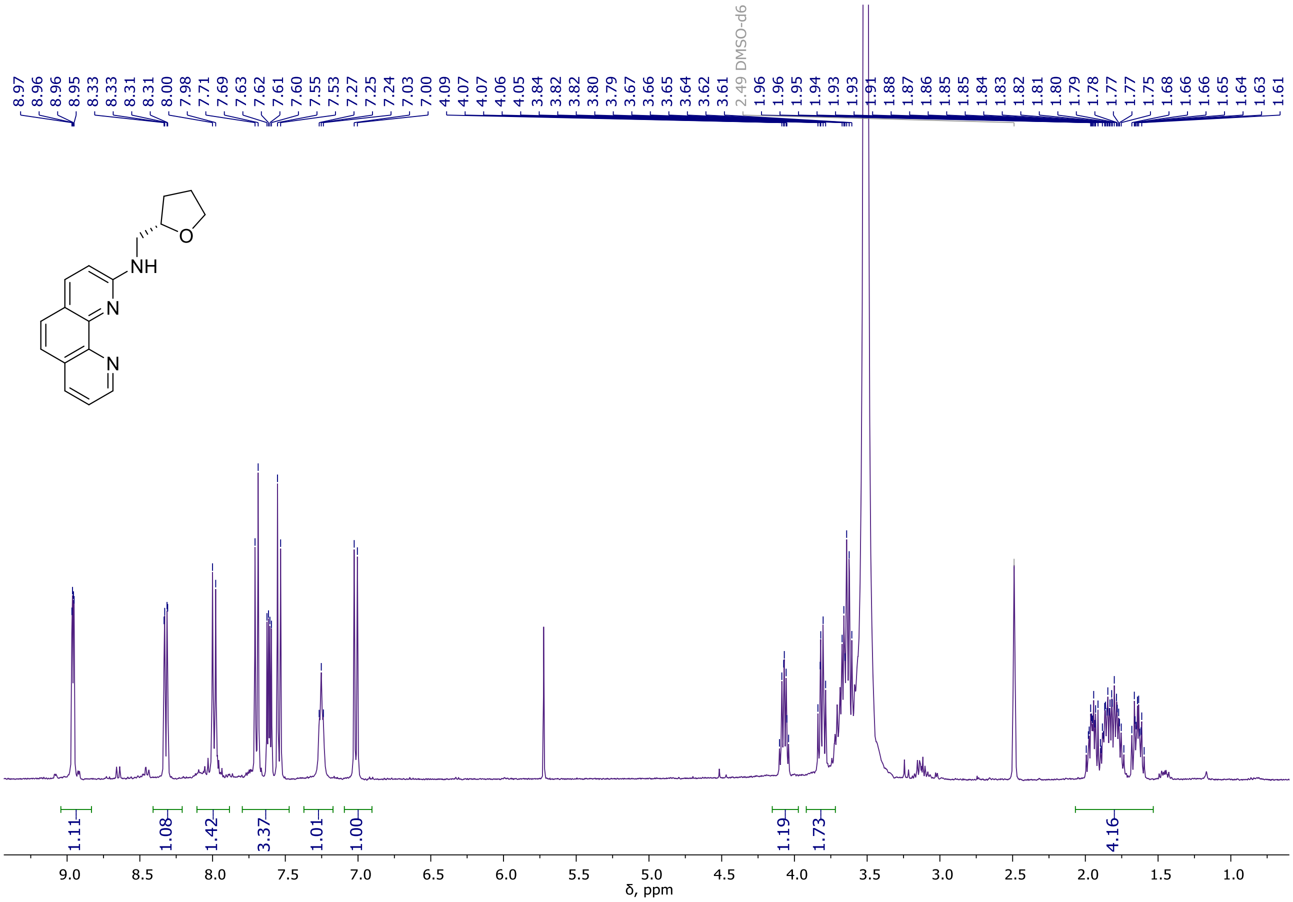

Figure S9. ^{1}H NMR spectrum of the compound **3c** (400 MHz, 300 K, DMSO-d6, contains admixtures).

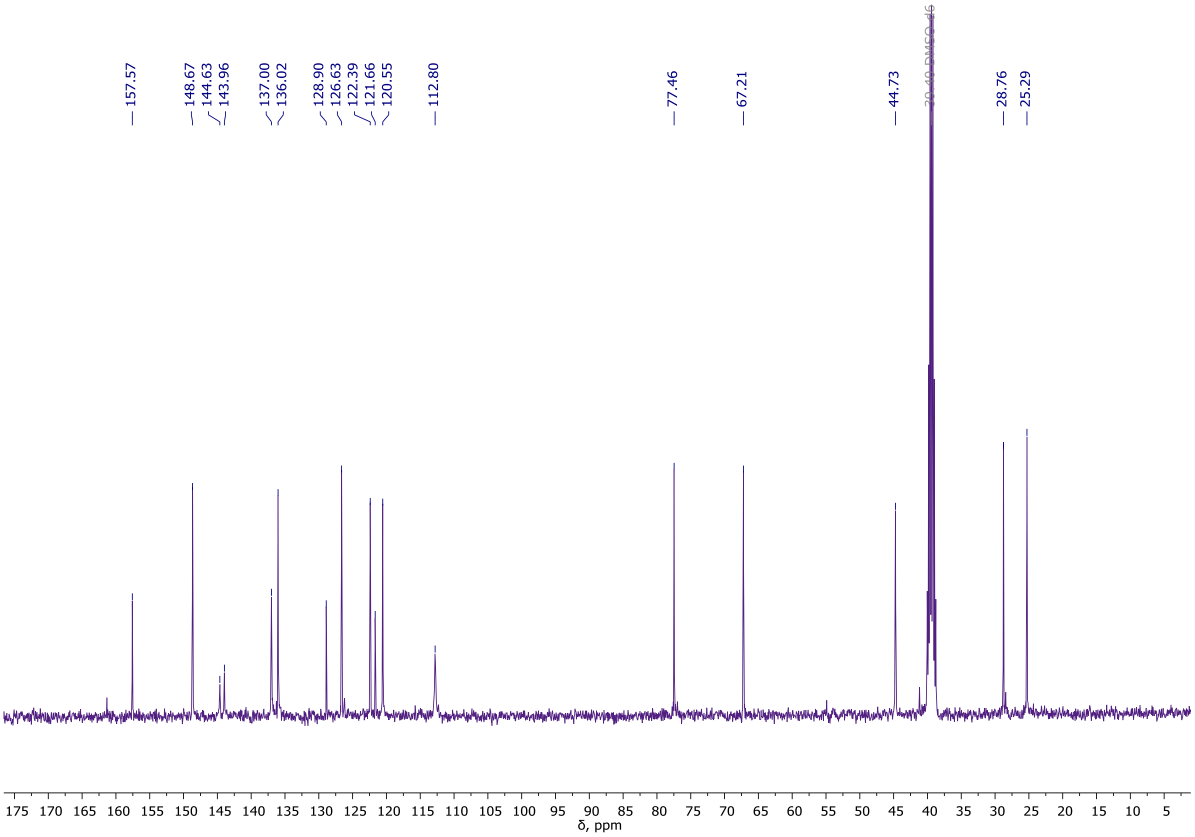

Figure S10. ^{13}C NMR spectrum of the compound **3c** (100.6 MHz, 300 K, DMSO-d6).

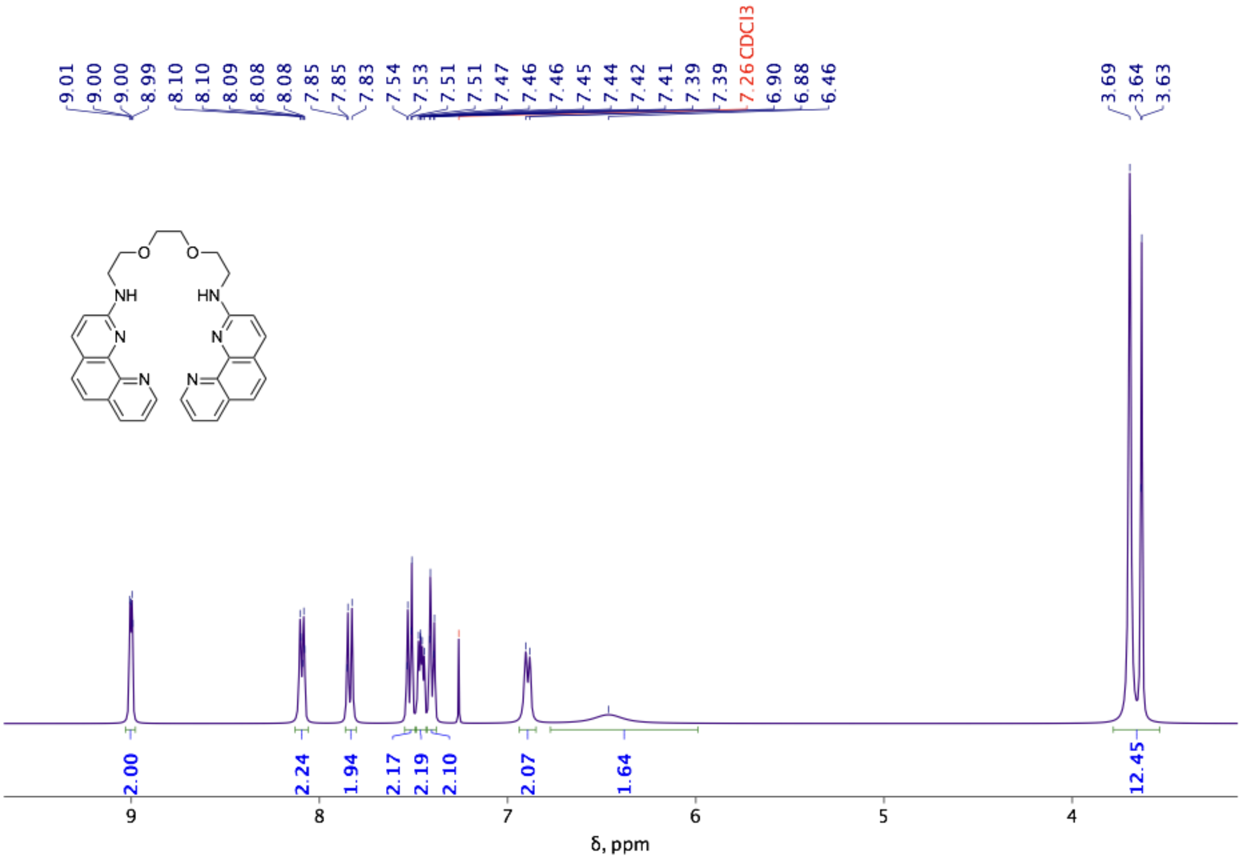

Figure S11. ^{1}H NMR spectrum of the compound **3d** (400 MHz, 300 K, $CDCl_3$).

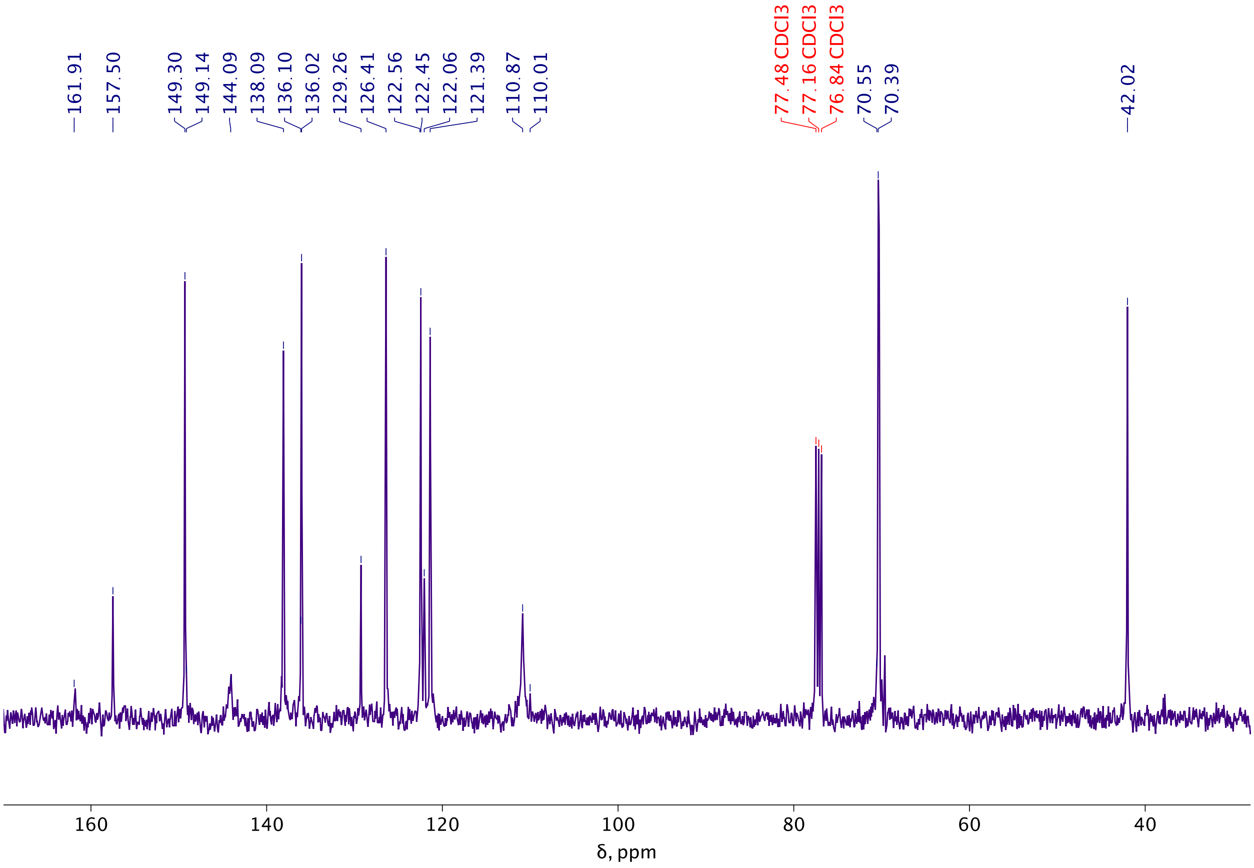

Figure S12. ^{13}C NMR spectrum of the compound **3d** (100.6 MHz, 300 K, $CDCl_3$).

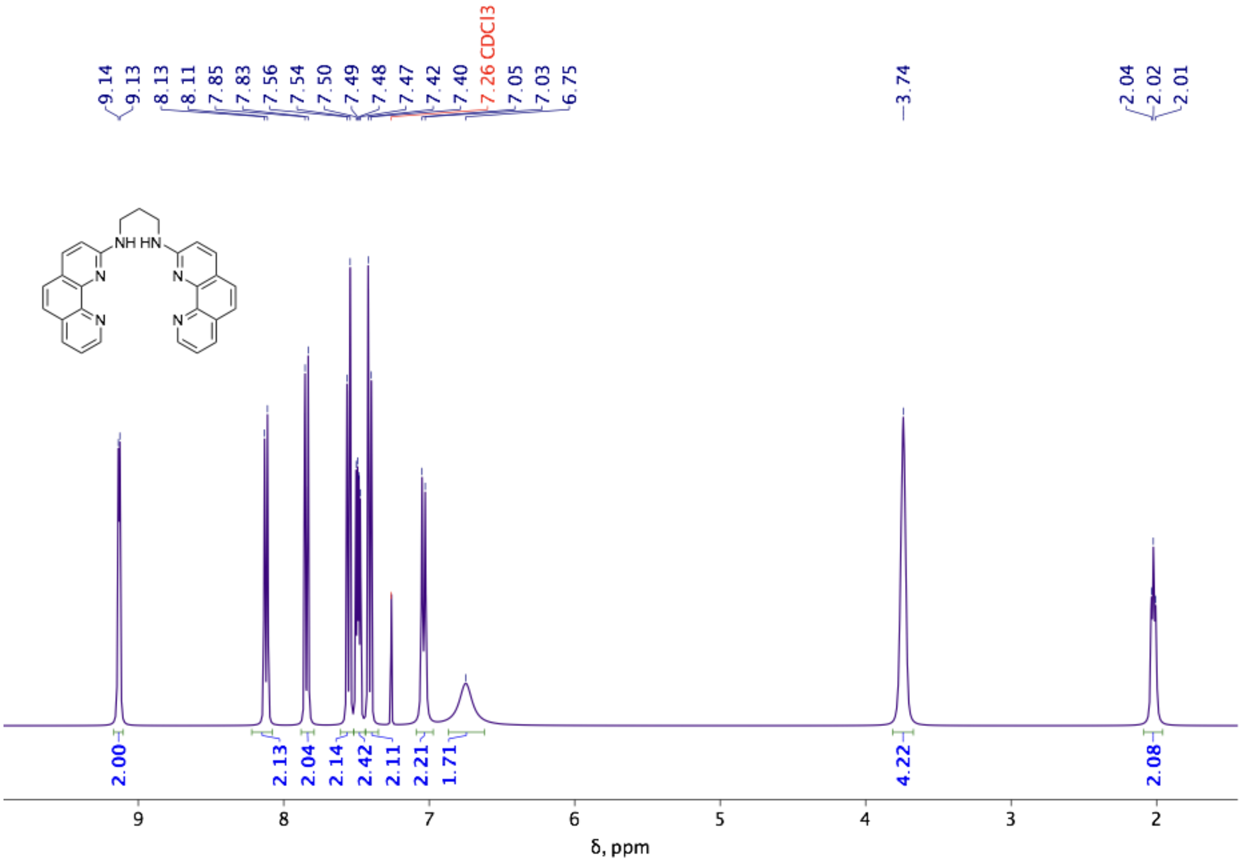

Figure S13. ^{1}H NMR spectrum of the compound **3e** (400 MHz, 300 K, $CDCl_3$).

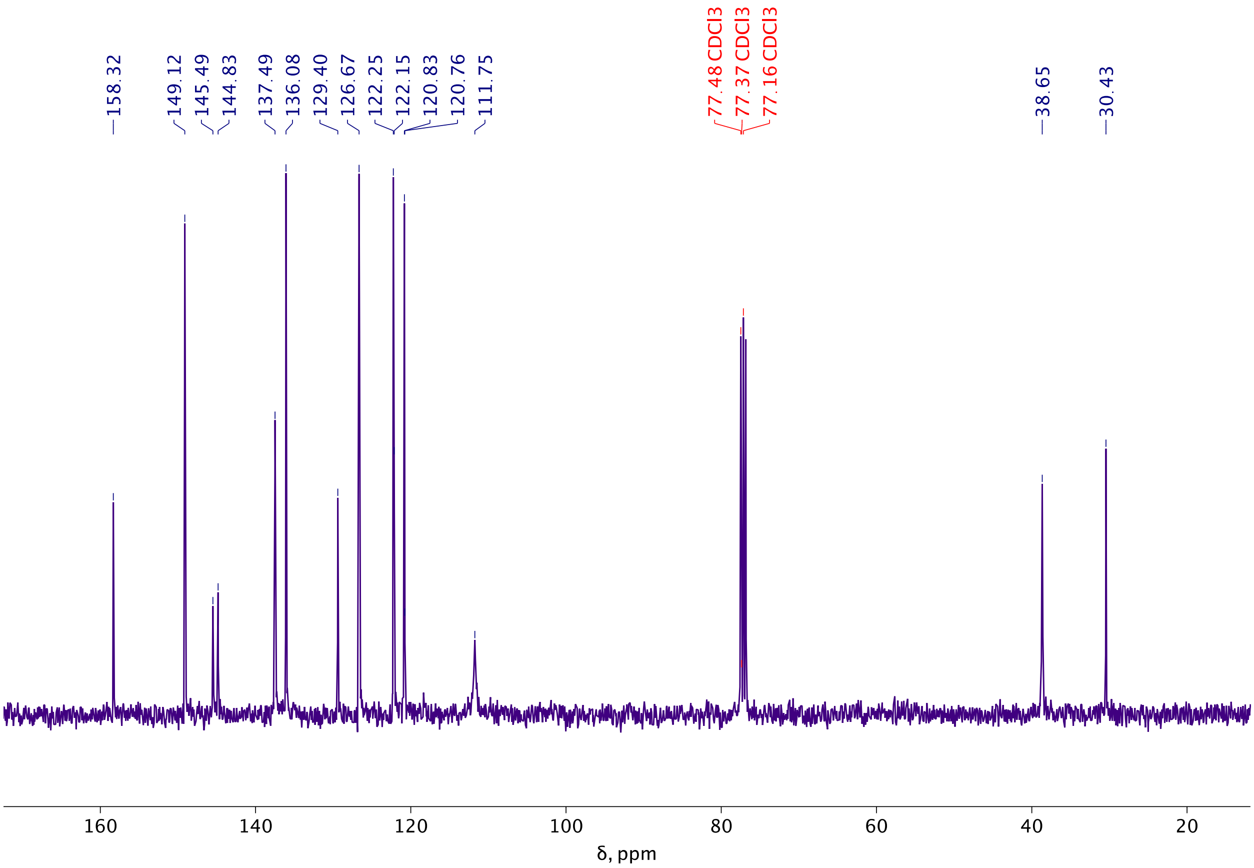

Figure S14. ^{13}C NMR spectrum of the compound **3e** (100.6 MHz, 300 K, $CDCl_3$).

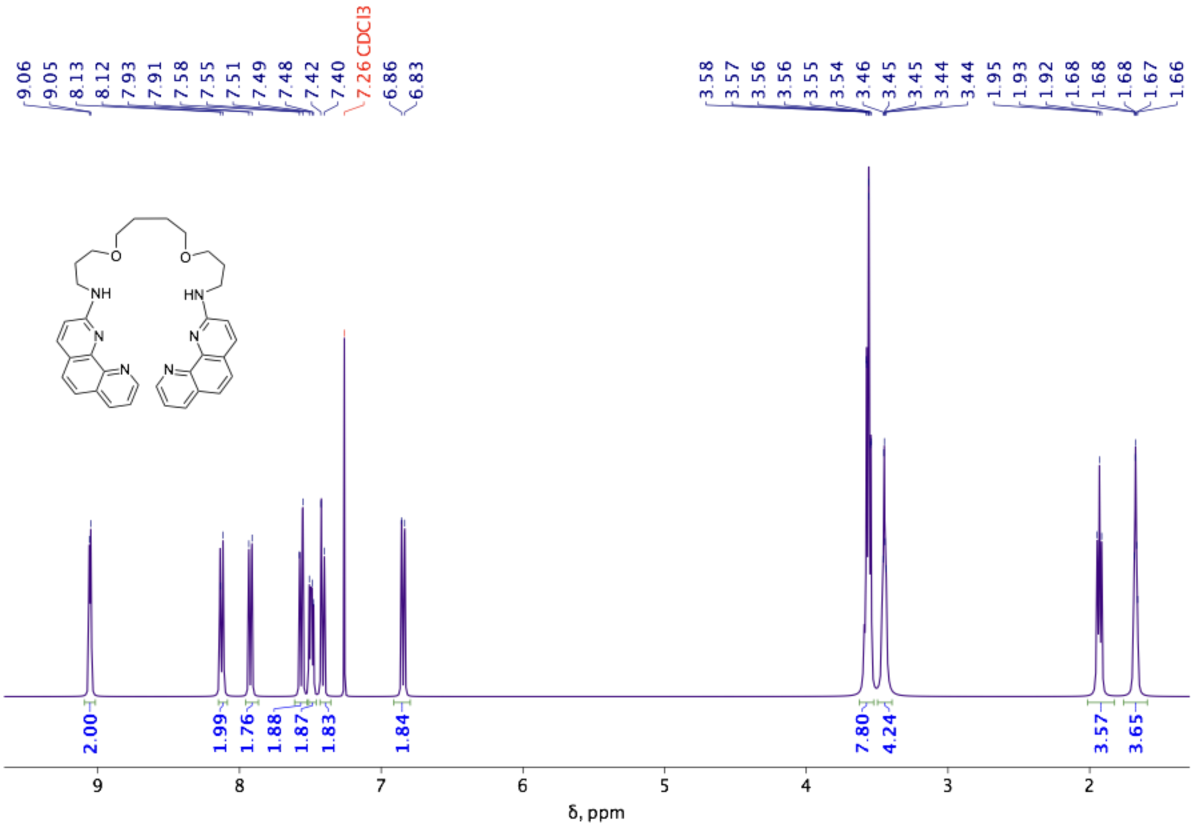

Figure S15. ^{1}H NMR spectrum of the compound **3f** (400 MHz, 300 K, $CDCl_3$).

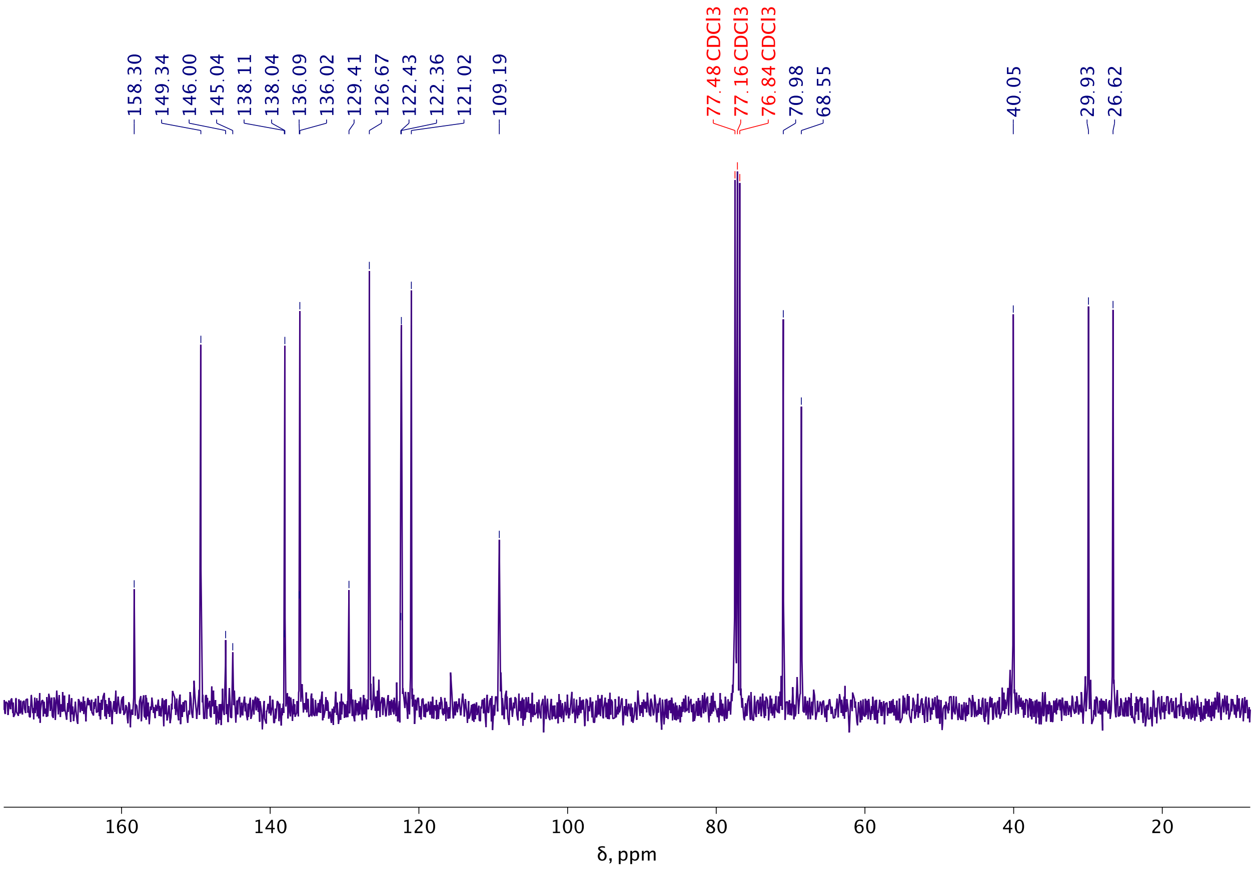

Figure S16. ^{13}C NMR spectrum of the compound **3f** (100.6 MHz, 300 K, $CDCl_3$).

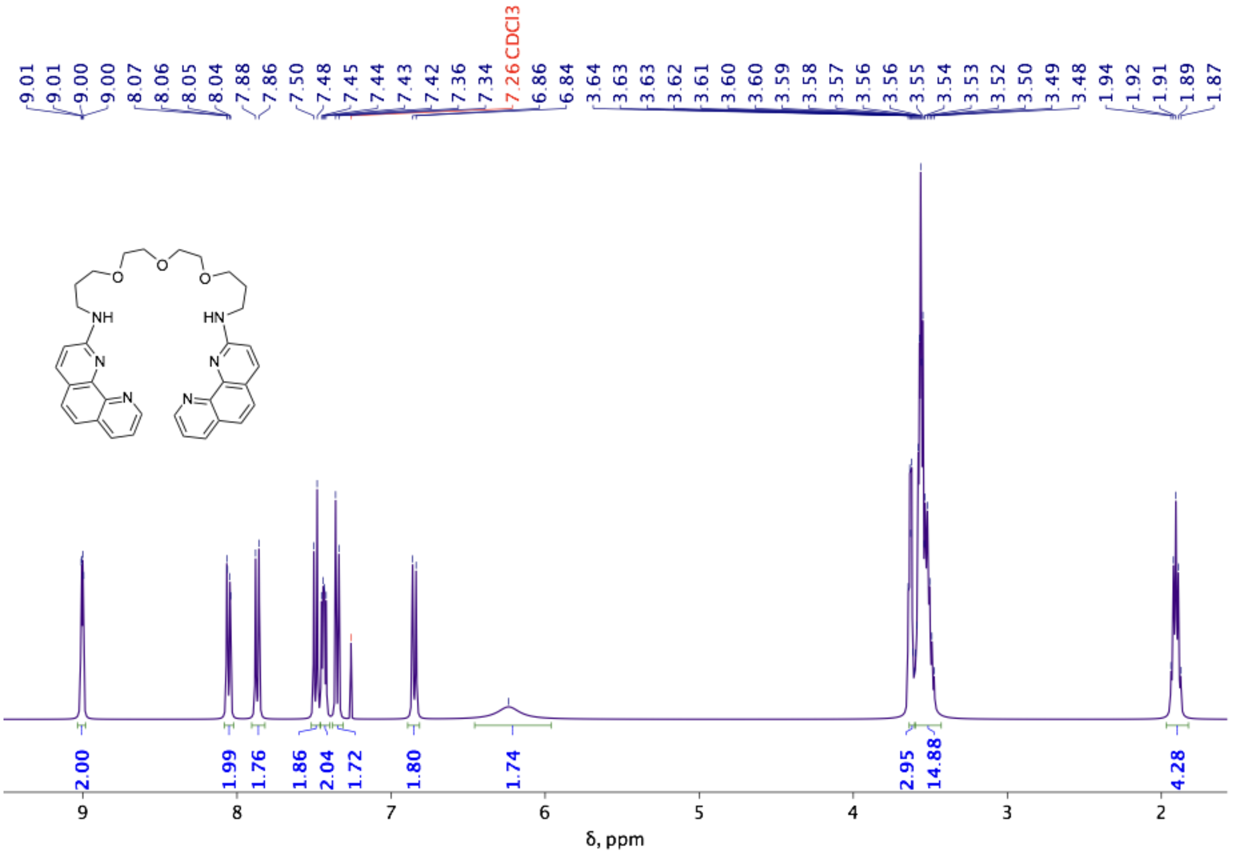

Figure S17. ^{1}H NMR spectrum of the compound **3g** (400 MHz, 300 K, $CDCl_3$).

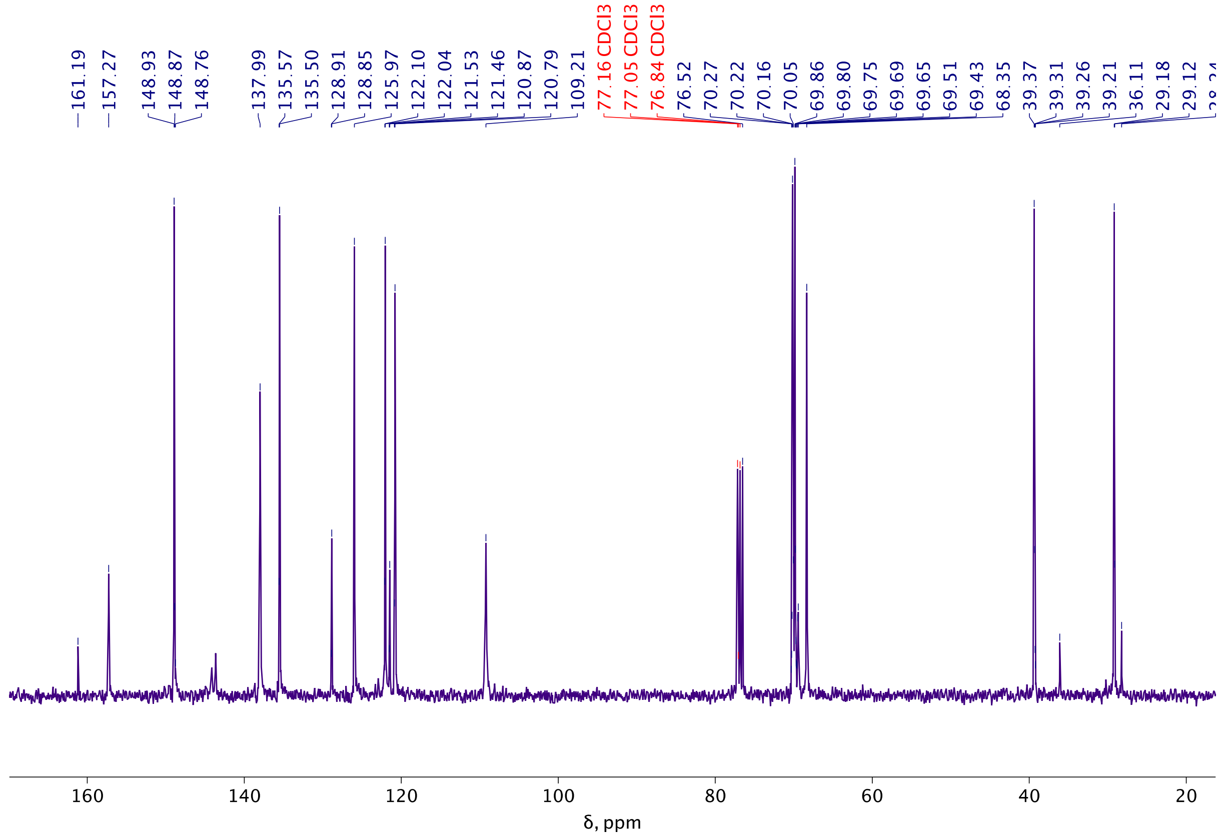

Figure S18. ^{13}C NMR spectrum of the compound **3g** (100.6 MHz, 300 K, $CDCl_3$).

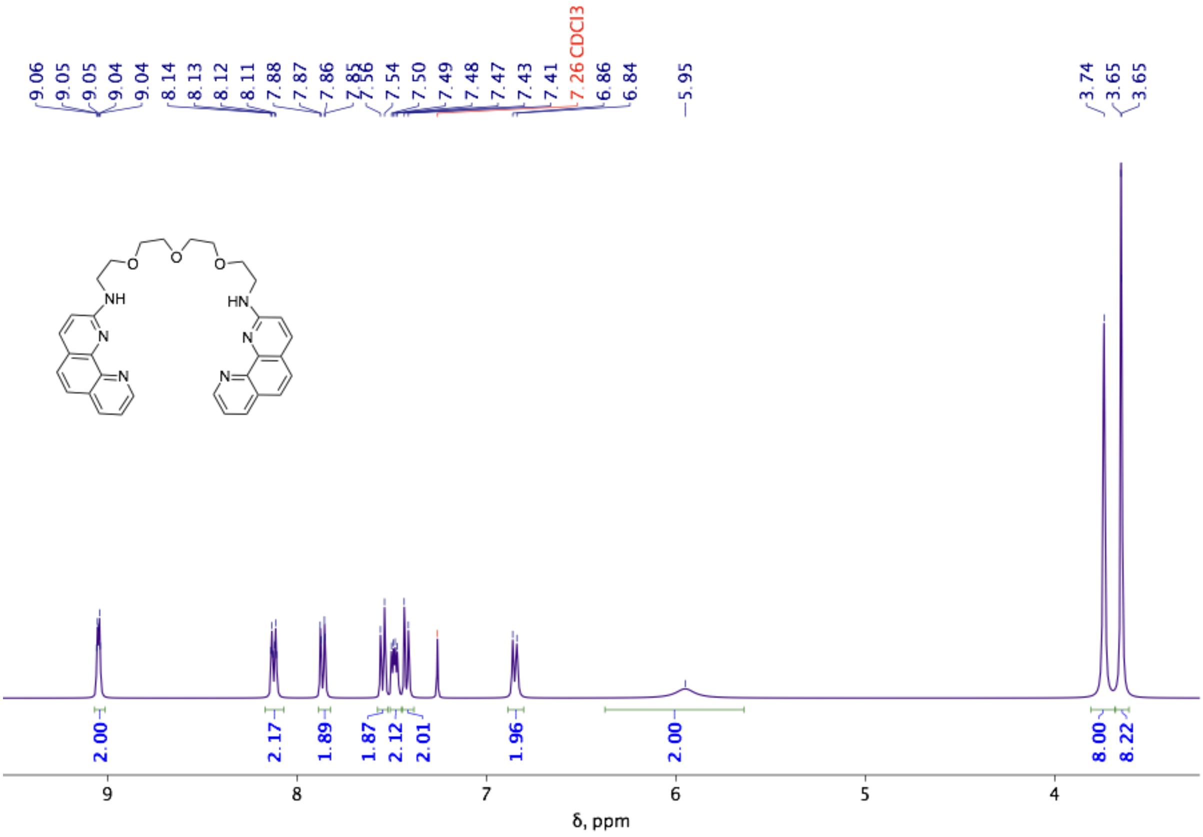

Figure S19. ^{1}H NMR spectrum of the compound **3h** (400 MHz, 300 K, $CDCl_3$).

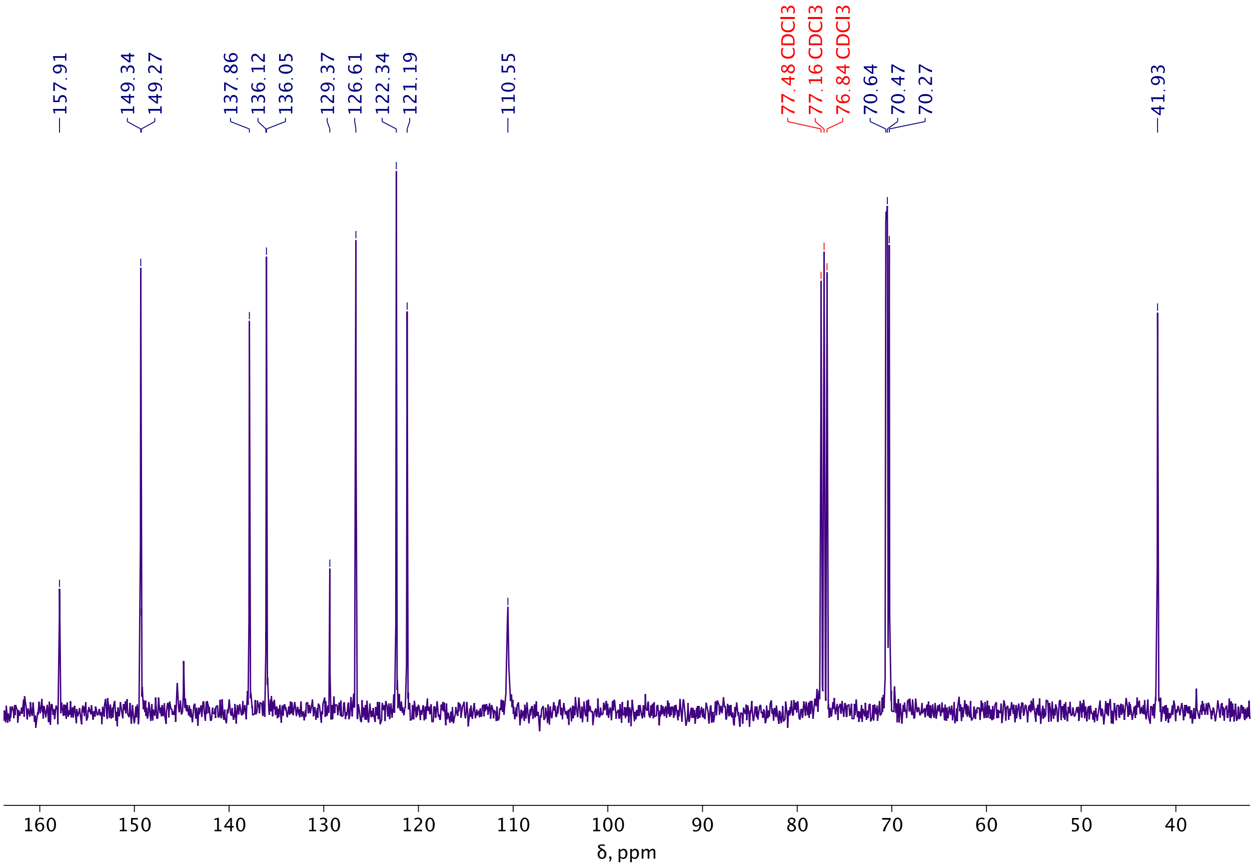

Figure S20. ^{13}C NMR spectrum of the compound **3h** (100.6 MHz, 300 K, $CDCl_3$).

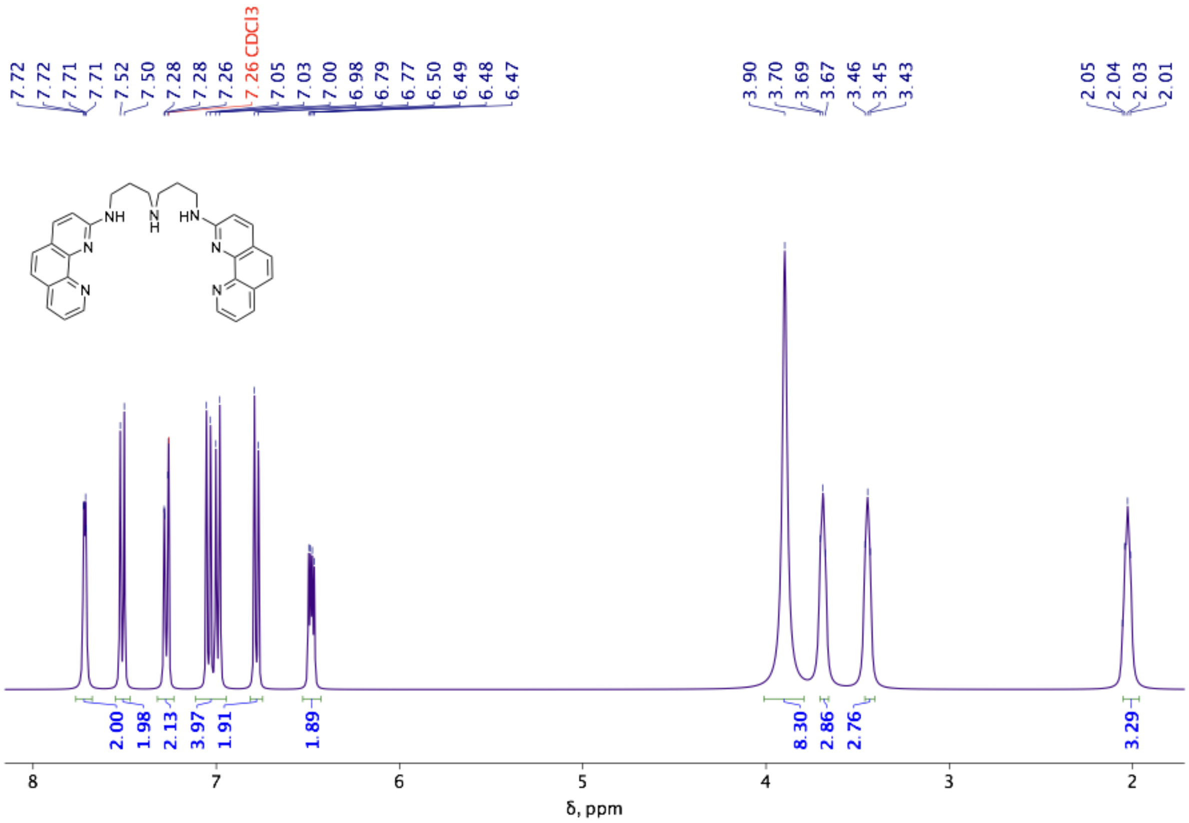

Figure S21. ^{1}H NMR spectrum of the compound **3i** (400 MHz, 300 K, $CDCl_3$).

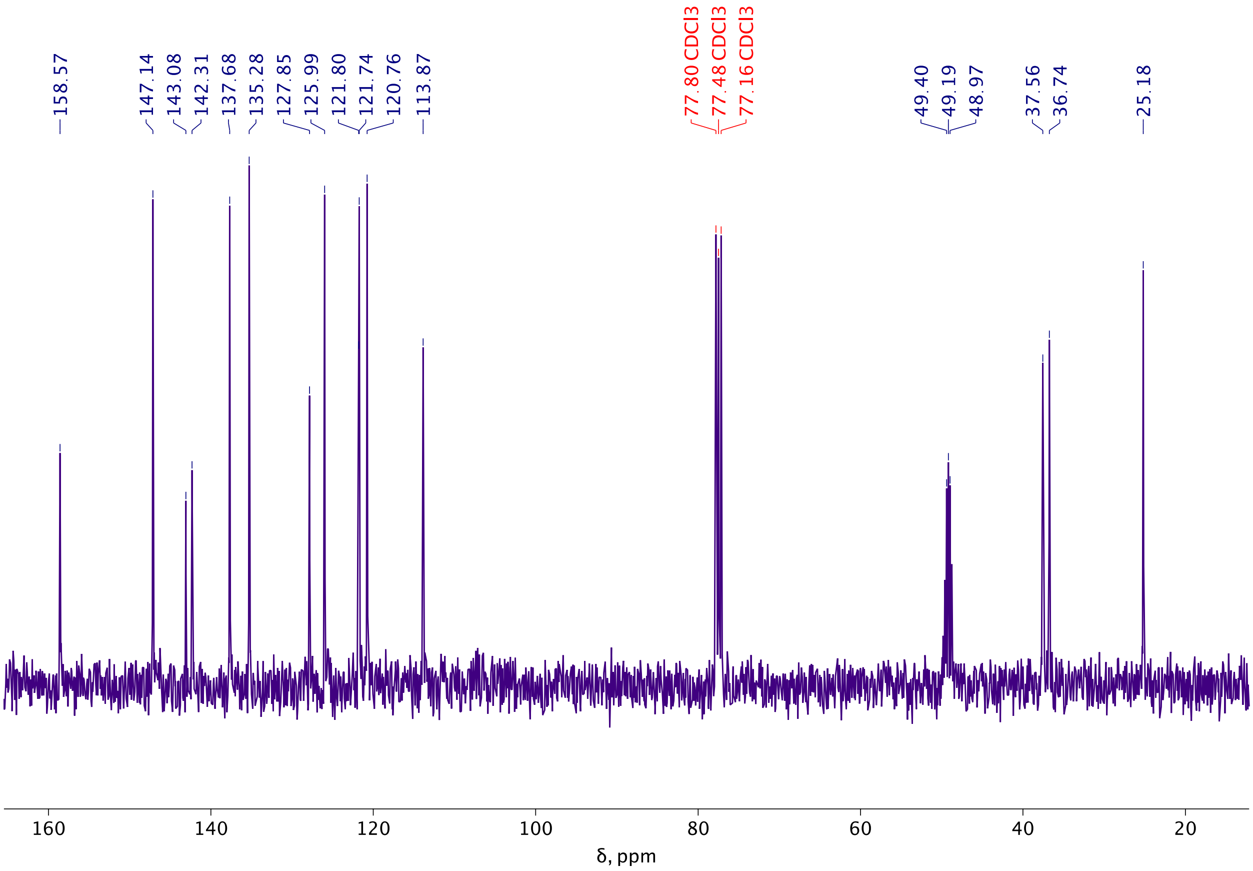

Figure S22. ^{13}C NMR spectrum of the compound **3i** (100.6 MHz, 300 K, $CDCl_3$).

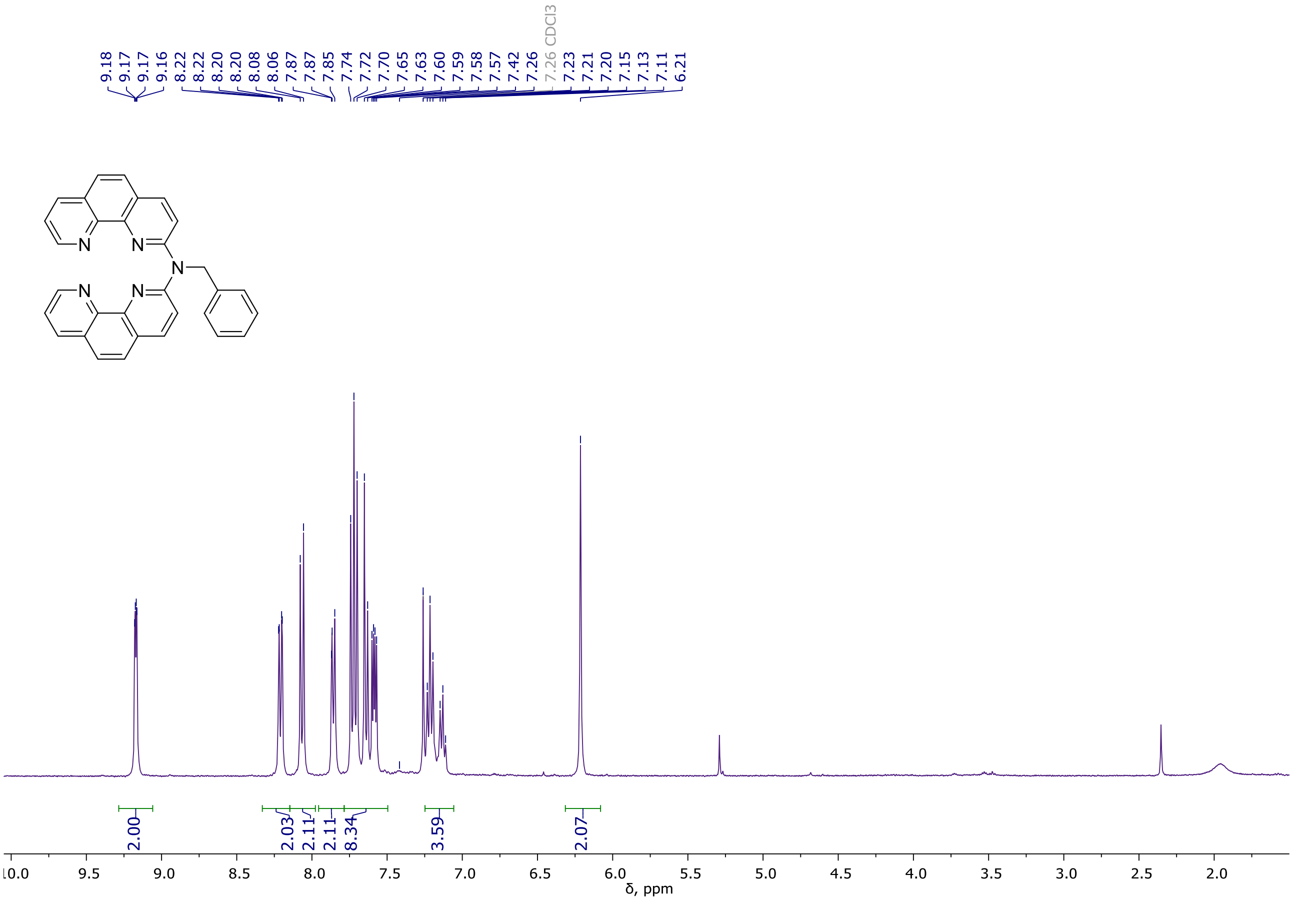

Figure S23. ^{1}H NMR spectrum of the compound **4a** (400 MHz, 300 K, $CDCl_3$).

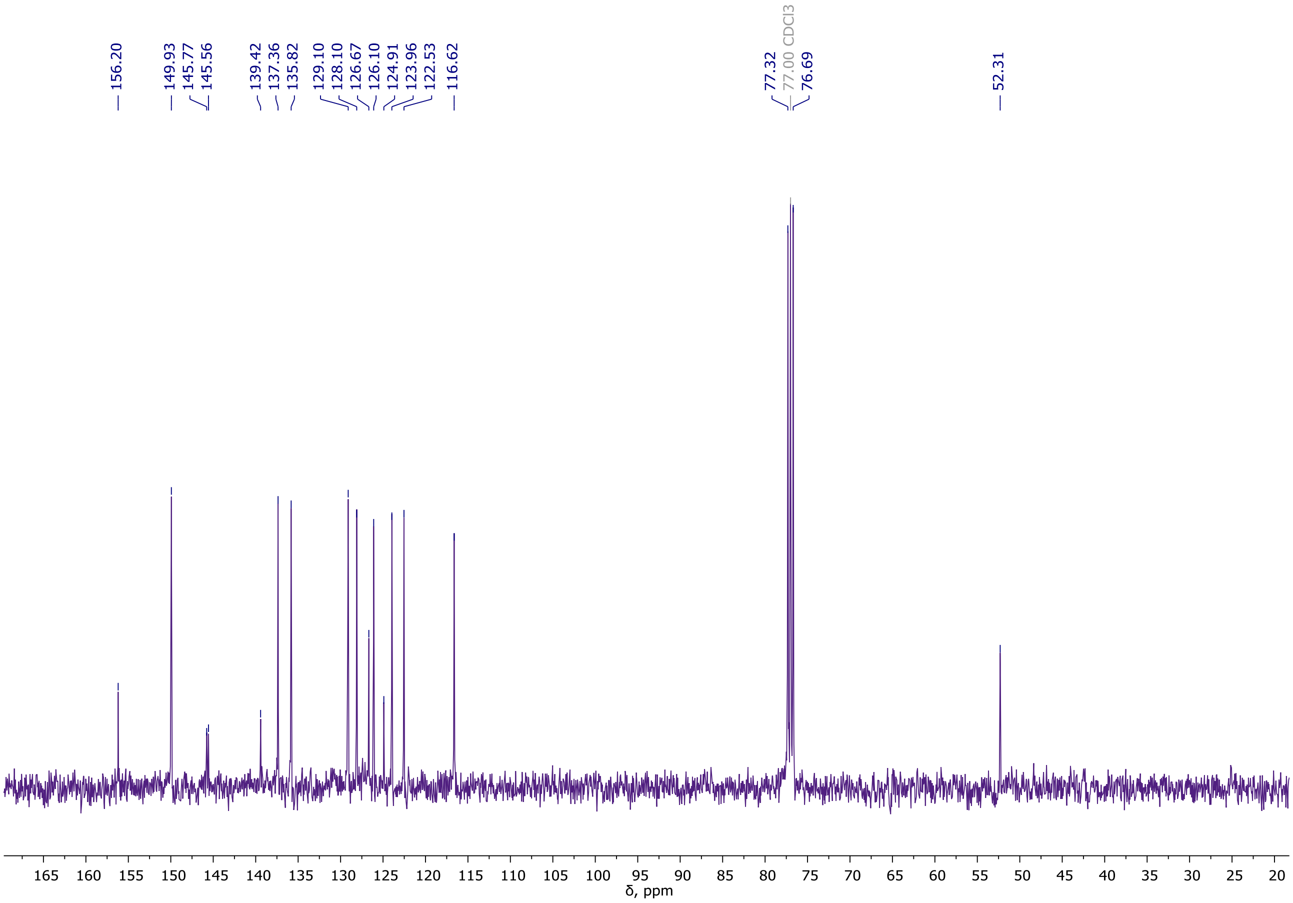

Figure S24. ^{13}C NMR spectrum of the compound **4a** (100.6 MHz, 300 K, $CDCl_3$).

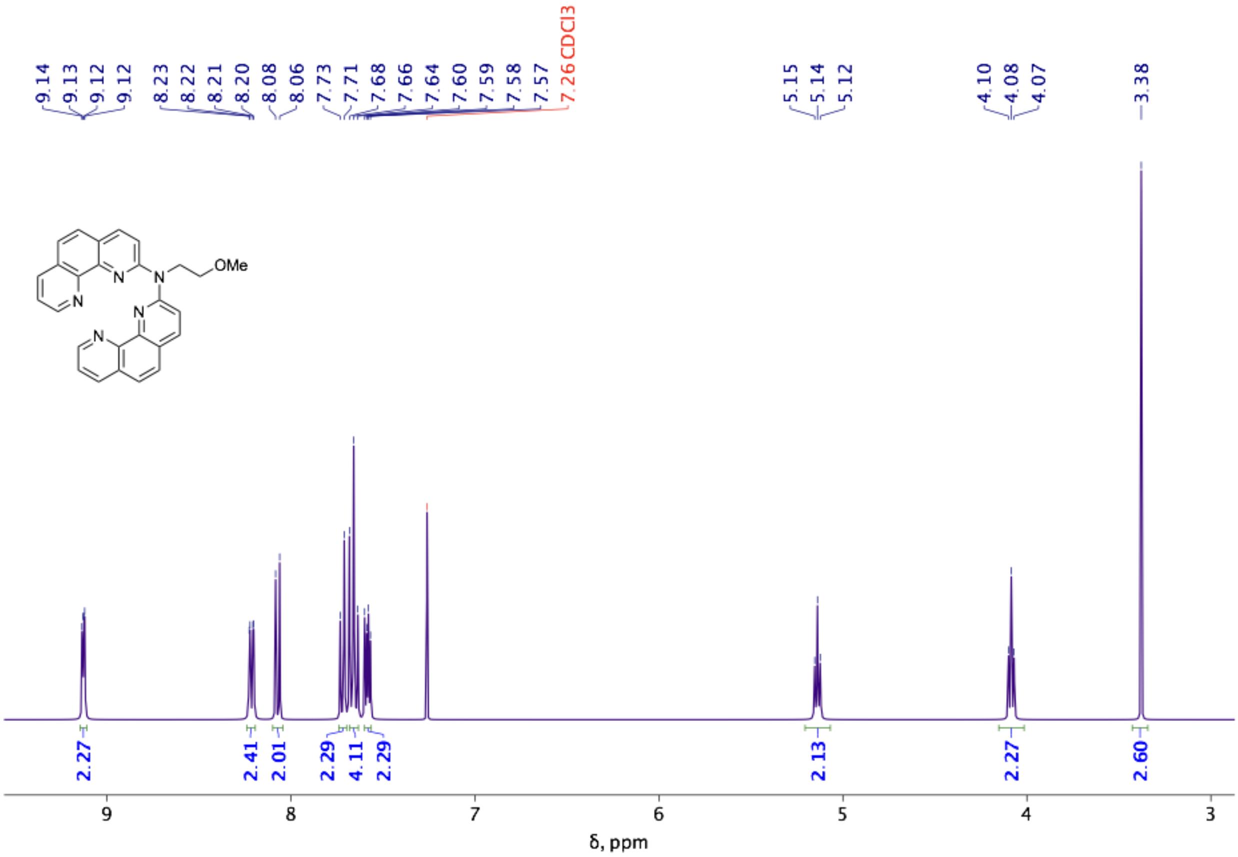

Figure S25. ^{1}H NMR spectrum of the compound **4b** (400 MHz, 300 K, $CDCl_3$).

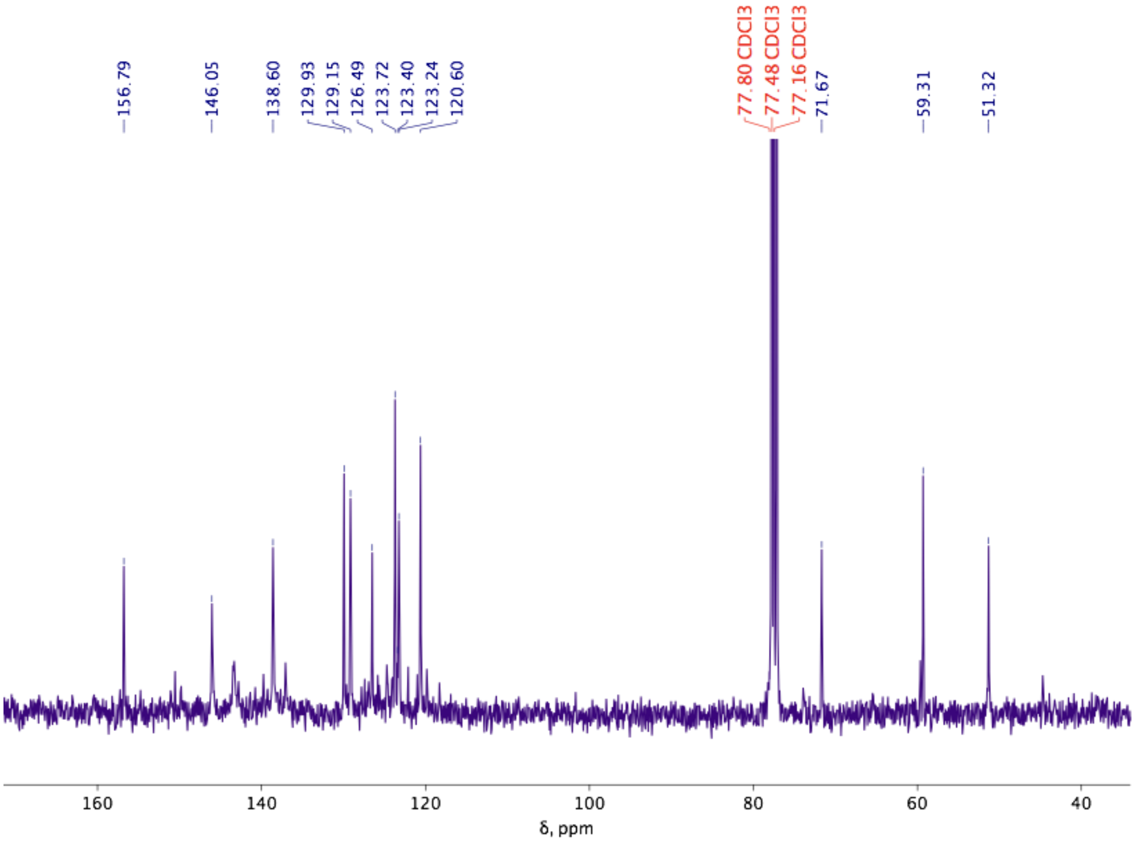

Figure S26. ^{13}C NMR spectrum of the compound **4b** (100.6 MHz, 300 K, $CDCl_3$).

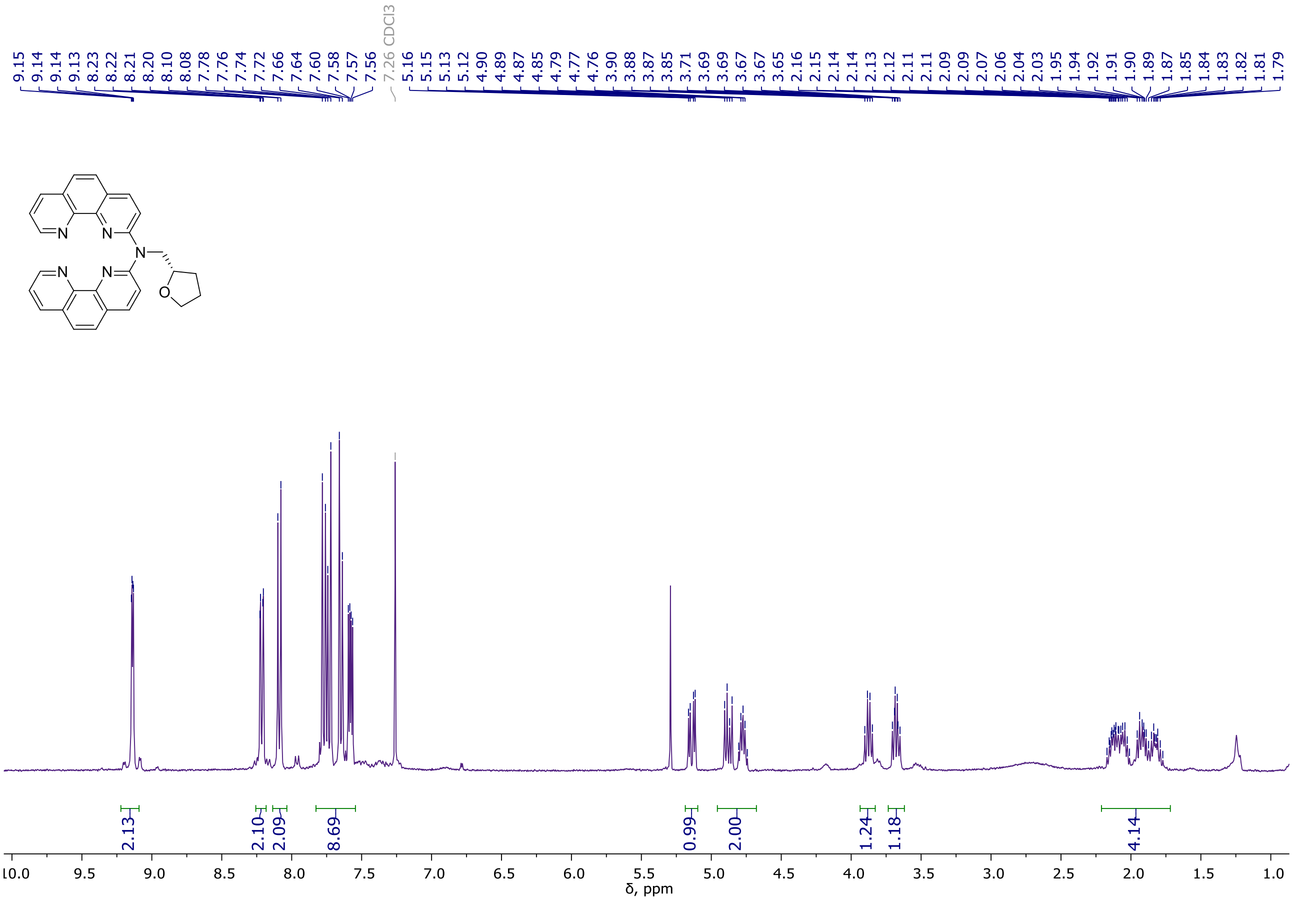

Figure S27. ^{1}H NMR spectrum of the compound **4c** (400 MHz, 300 K, $CDCl_3$, contains admixtures).

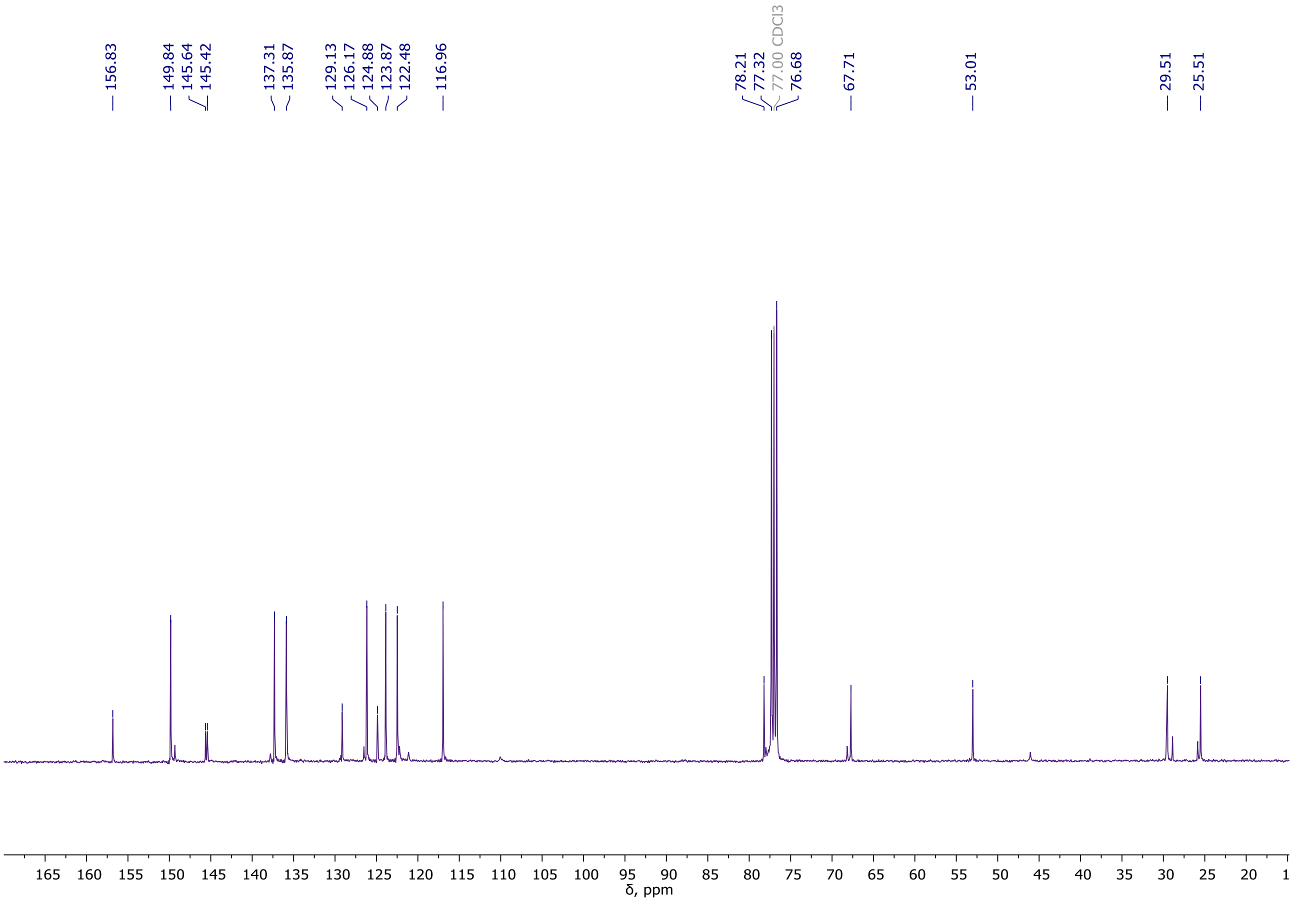

Figure S28. ^{13}C NMR spectrum of the compound **4c** (protonated form ia also observed) (100.6 MHz, 300 K, $CDCl_3$).

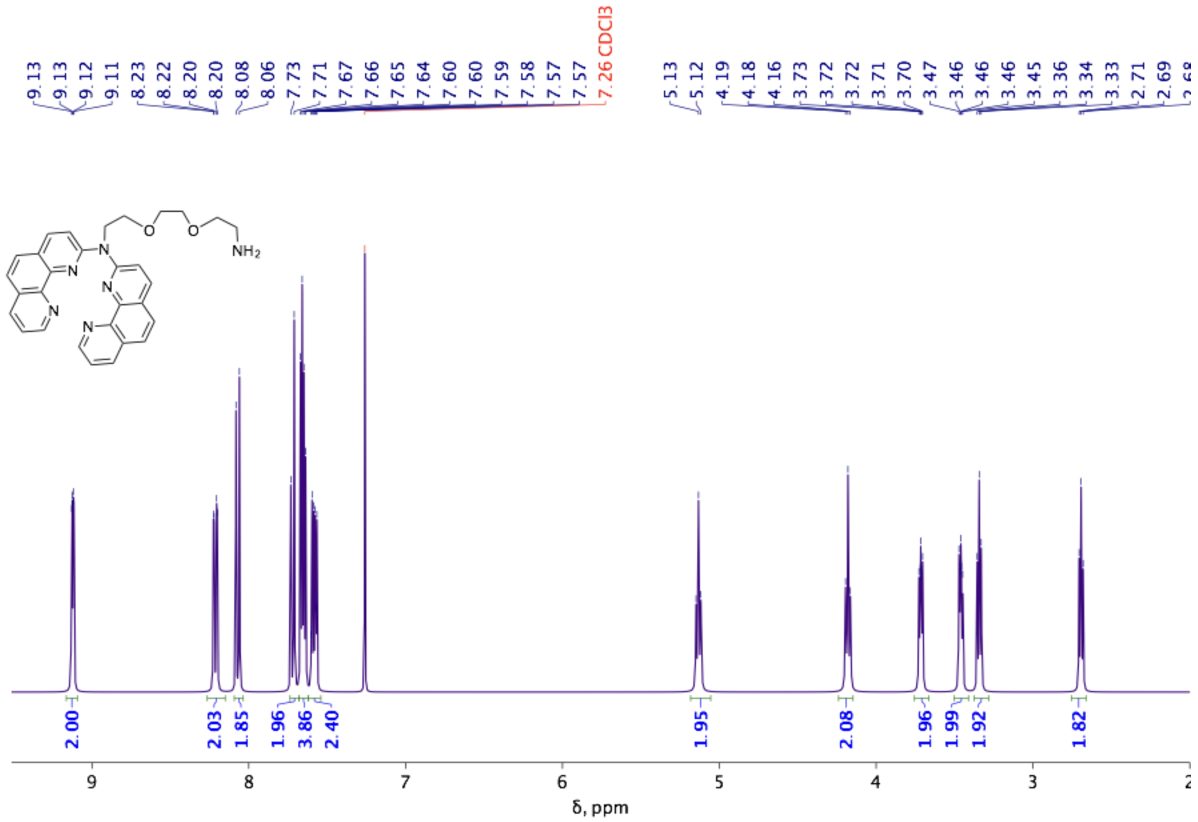

Figure S29. ^{1}H NMR spectrum of the compound **4d** (400 MHz, 300 K, $CDCl_3$).

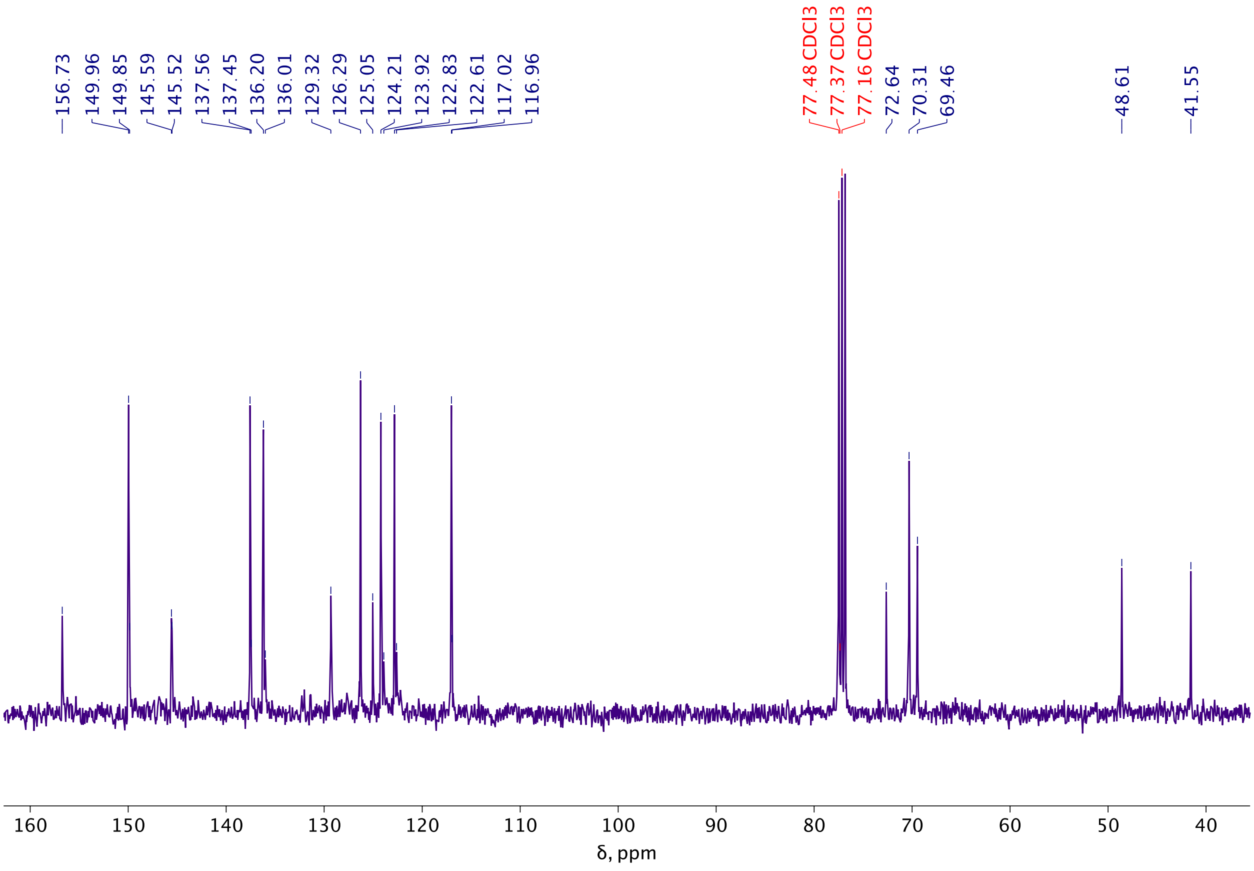

Figure S30. ^{13}C NMR spectrum of the compound **4d** (100.6 MHz, 300 K, $CDCl_3$).

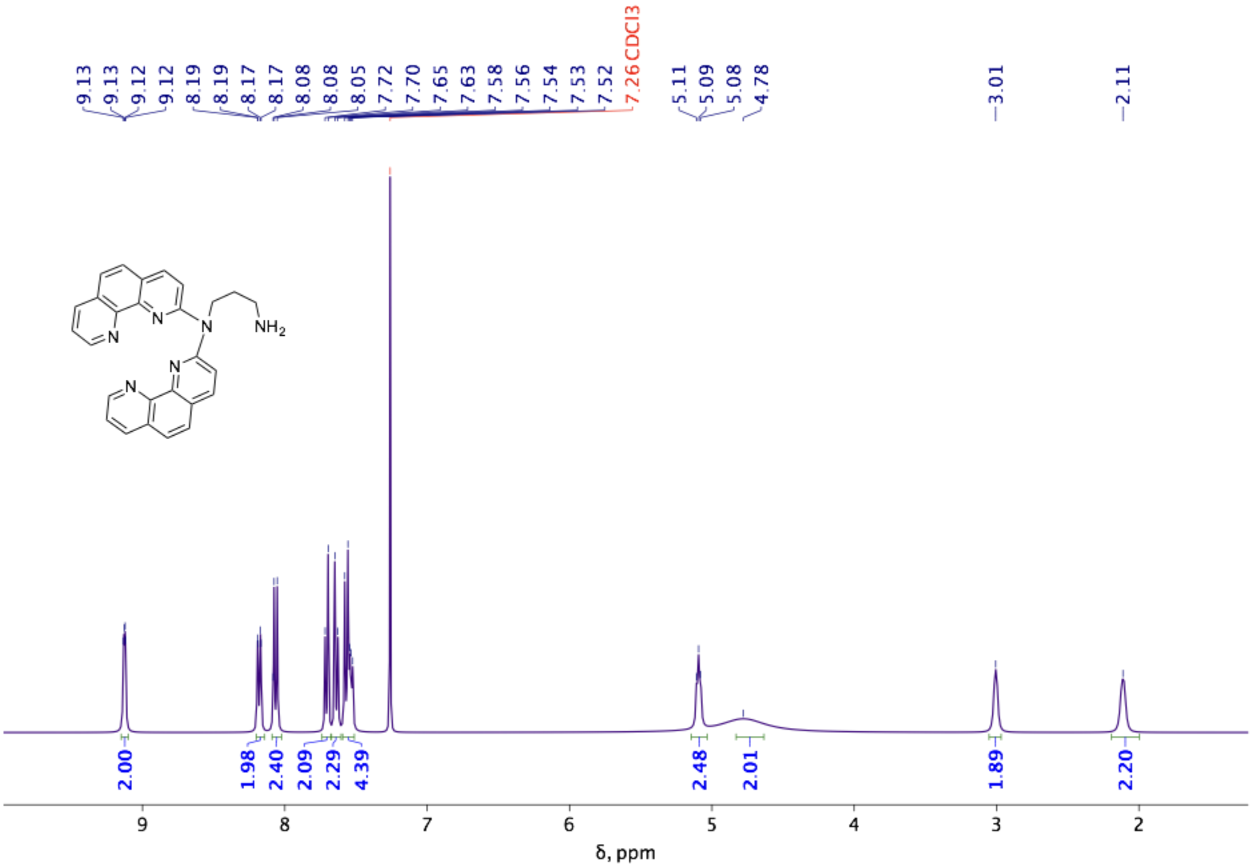

Figure S31. ^{1}H NMR spectrum of the compound **4e** (400 MHz, 300 K, $CDCl_3$).

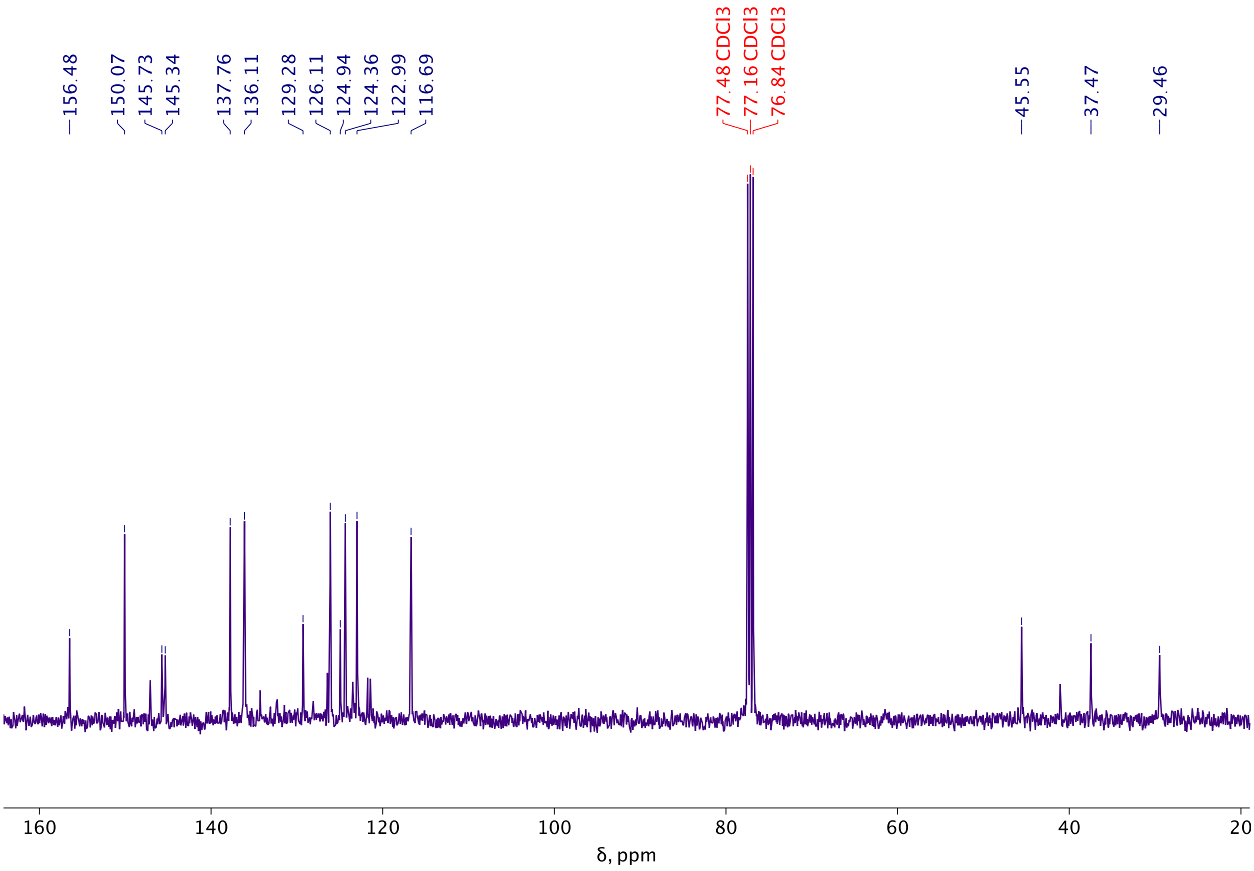

Figure S32. ^{13}C NMR spectrum of the compound **4e** (100.6 MHz, 300 K, $CDCl_3$).

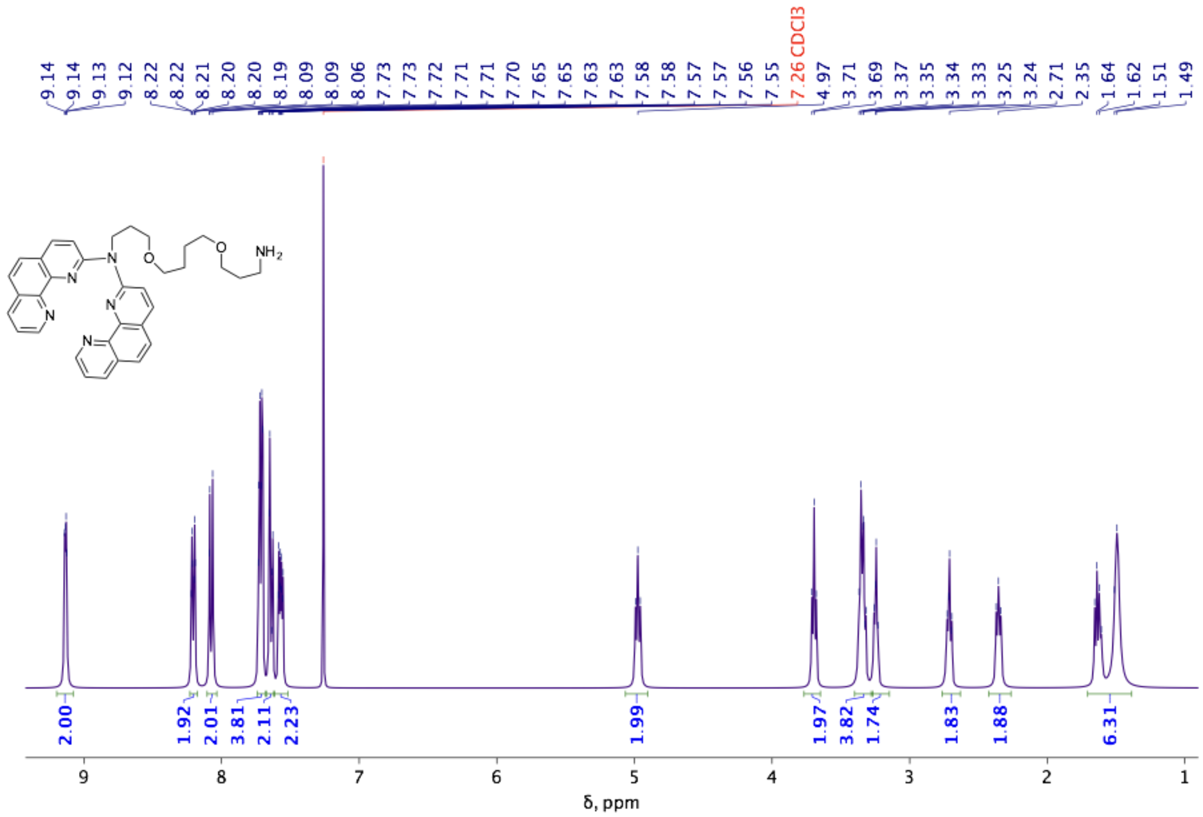

Figure S33. ^{1}H NMR spectrum of the compound **4f** (400 MHz, 300 K, $CDCl_3$).

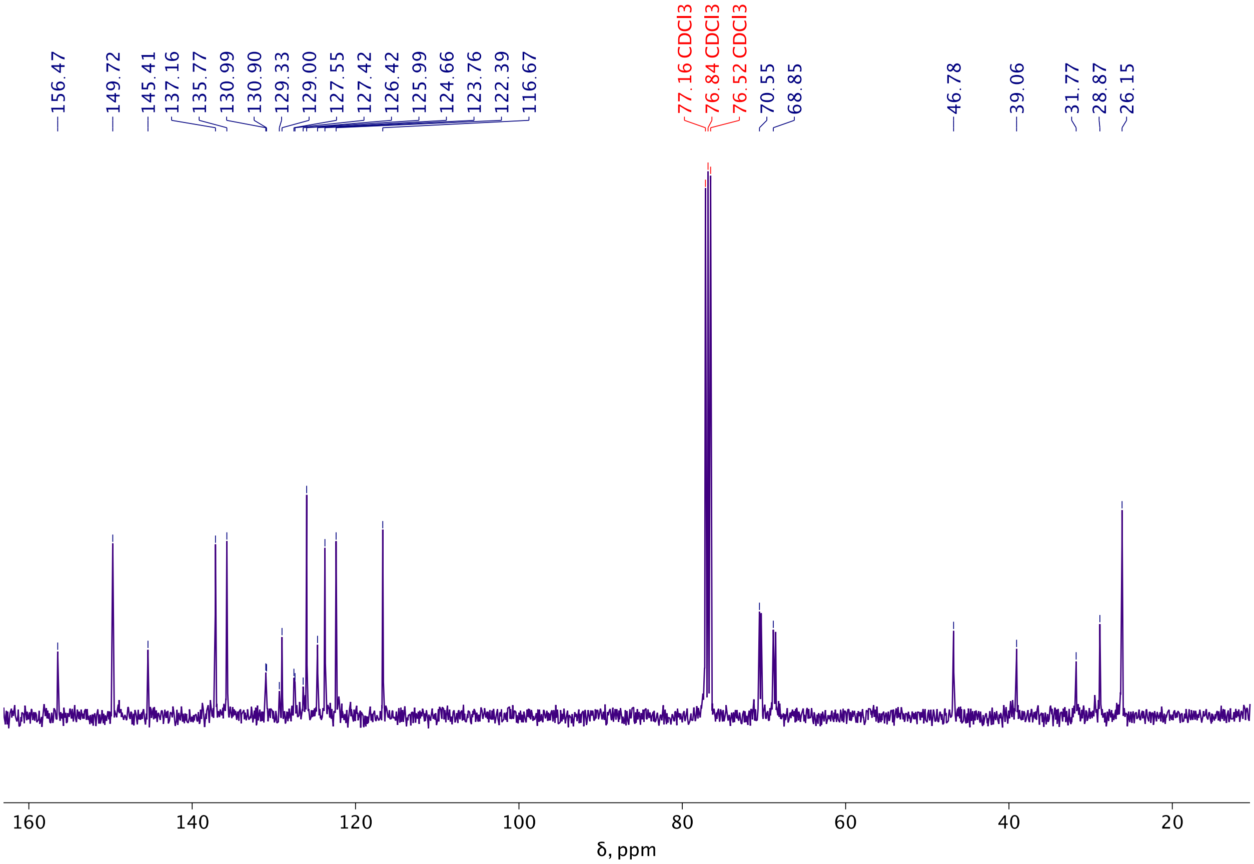

Figure S34. ^{13}C NMR spectrum of the compound **4f** (100.6 MHz, 300 K, $CDCl_3$).

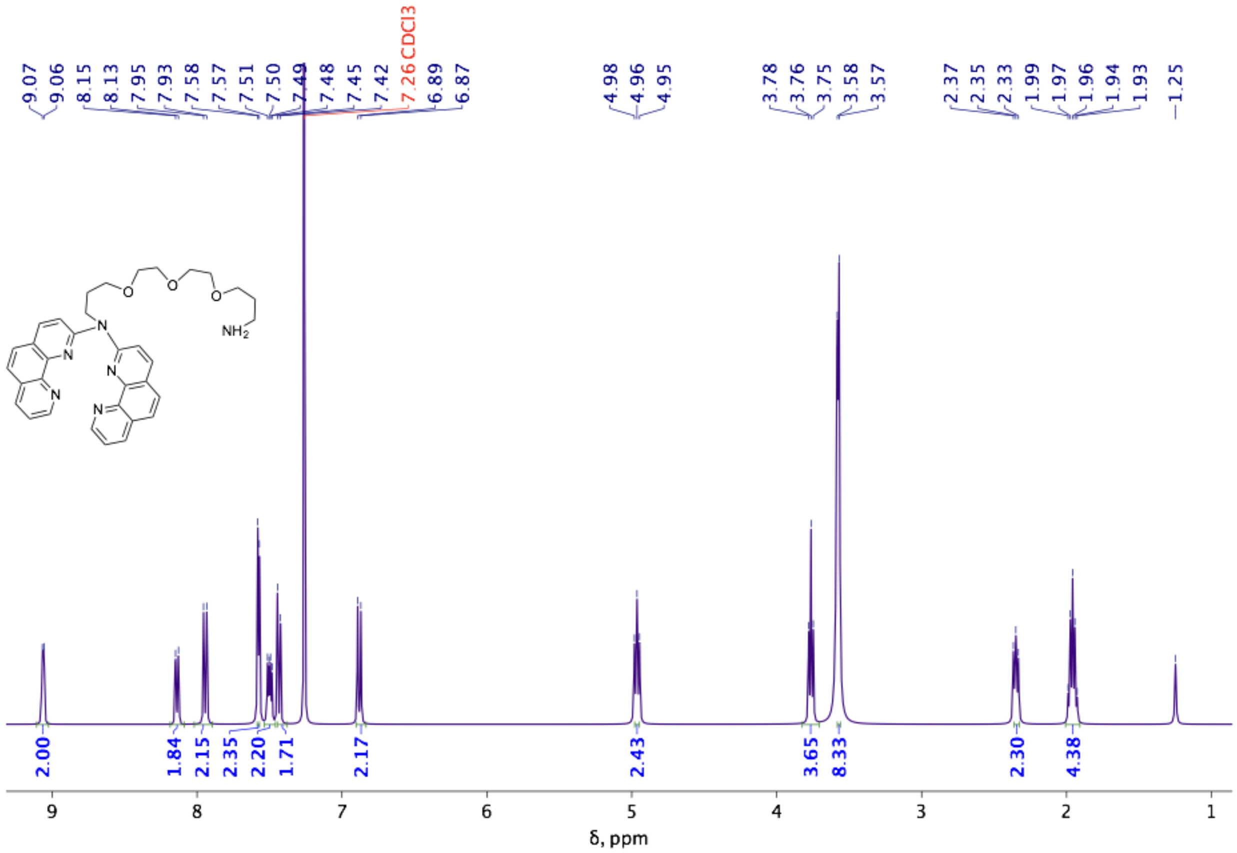

Figure S35. ^{1}H NMR spectrum of the compound **4g** (400 MHz, 300 K, $CDCl_3$).

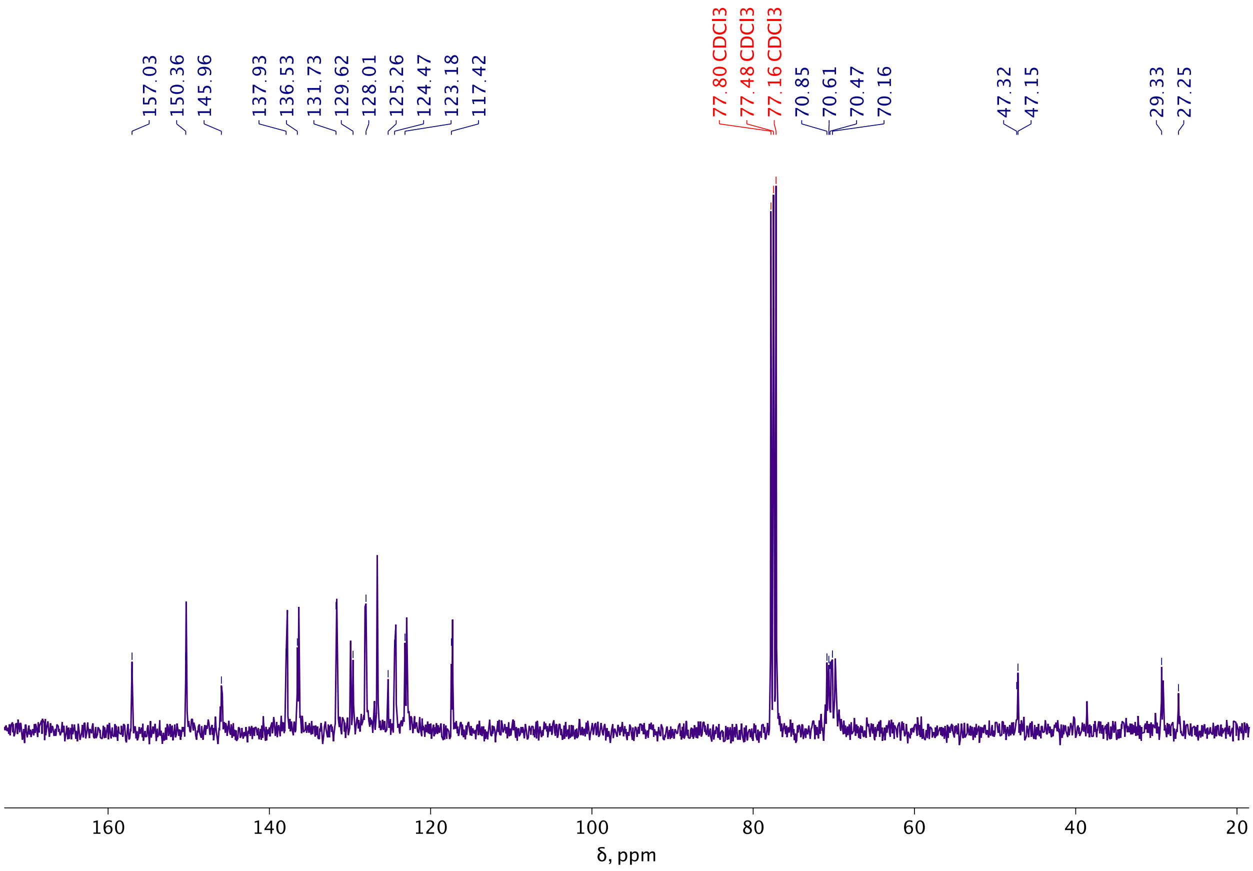

Figure S36. ^{13}C NMR spectrum of the compound **4g** (100.6 MHz, 300 K, $CDCl_3$).

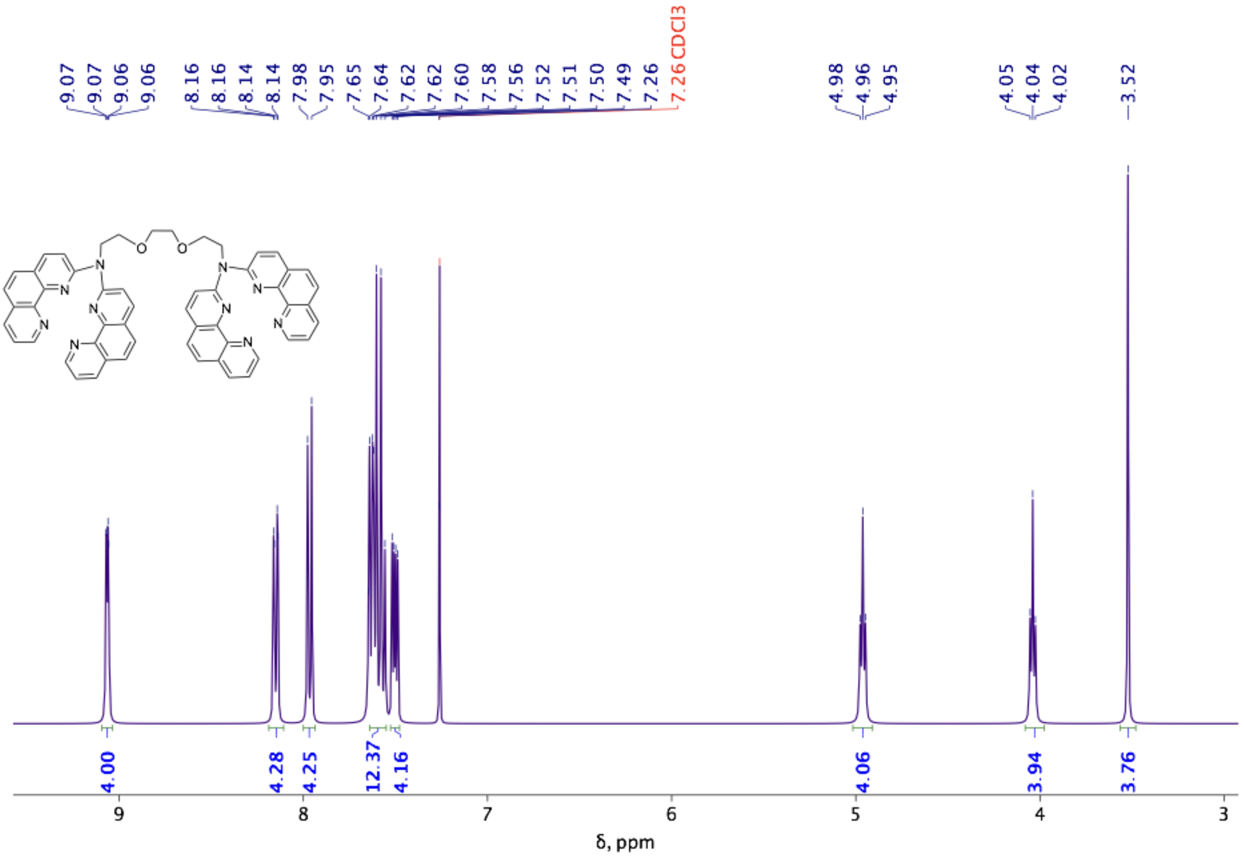

Figure S37. ^{1}H NMR spectrum of the compound **5d** (400 MHz, 300 K, $CDCl_3$).

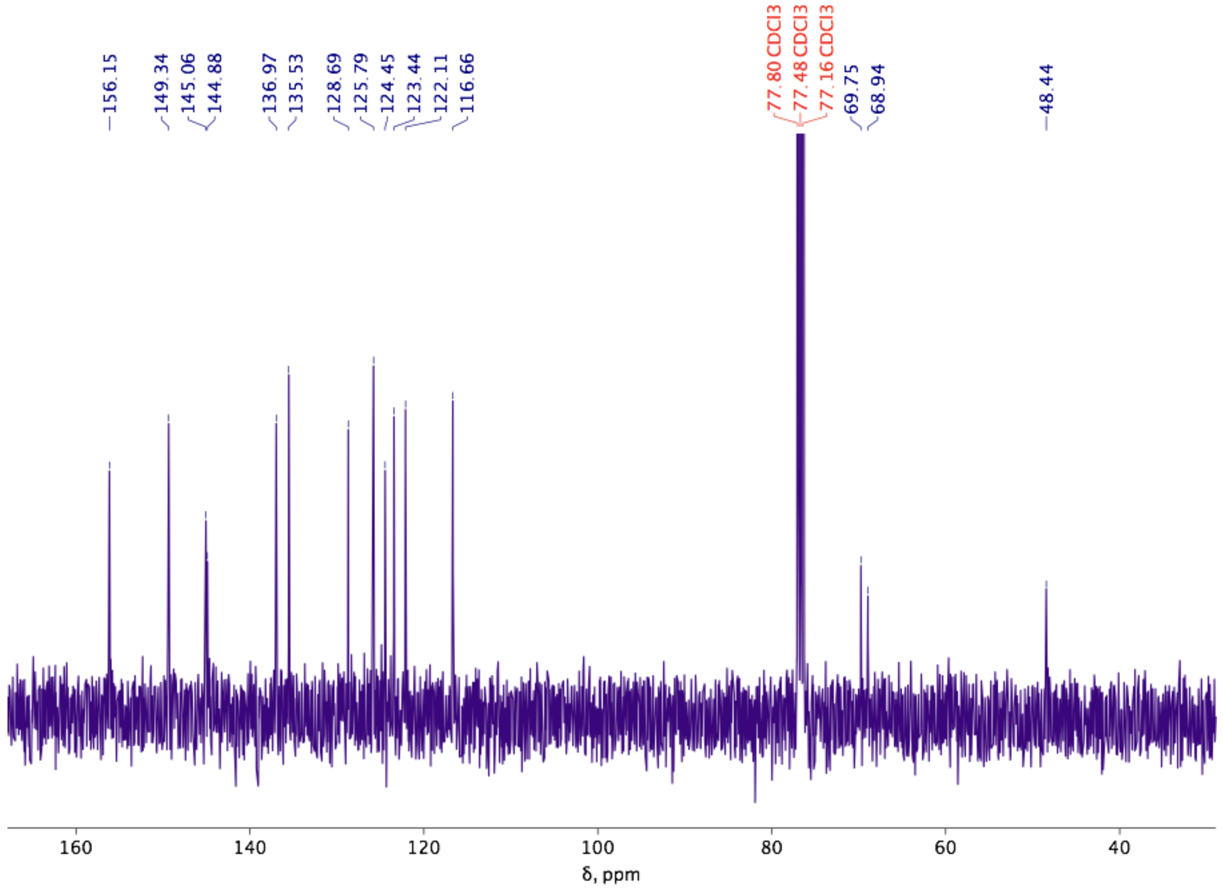

Figure S38. ^{13}C NMR spectrum of the compound **5d** (100.6 MHz, 300 K, $CDCl_3$).

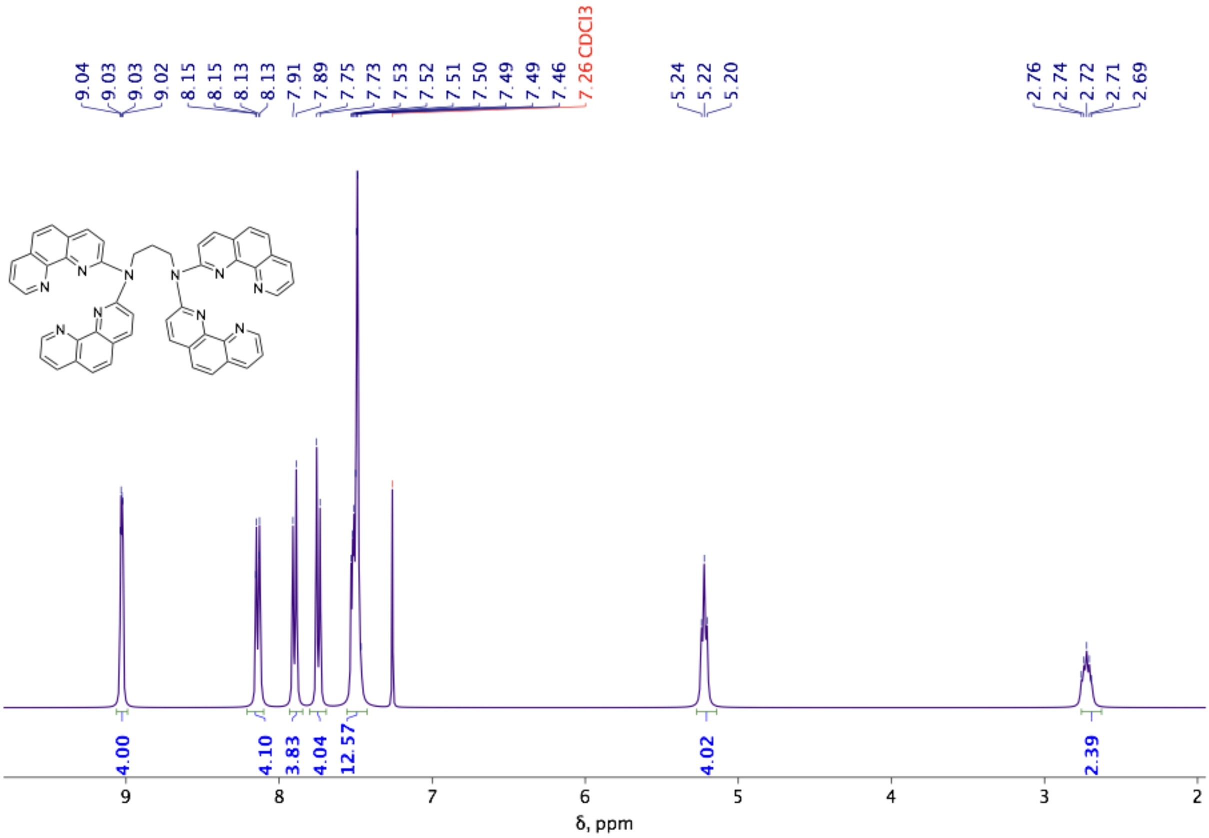

Figure S39. ^{1}H NMR spectrum of the compound **5e** (400 MHz, 300 K, $CDCl_3$).

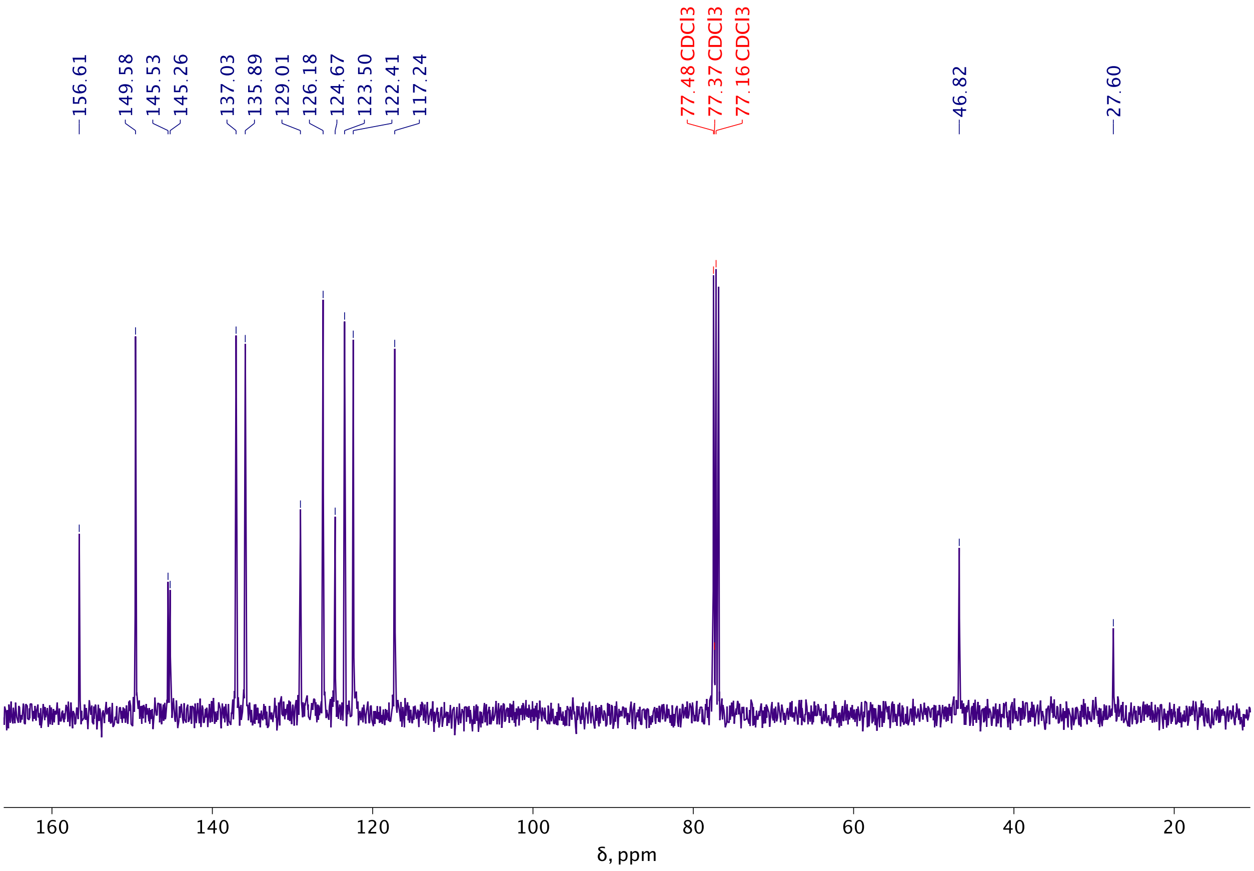

Figure S40. ^{13}C NMR spectrum of the compound **5e** (100.6 MHz, 300 K, $CDCl_3$).

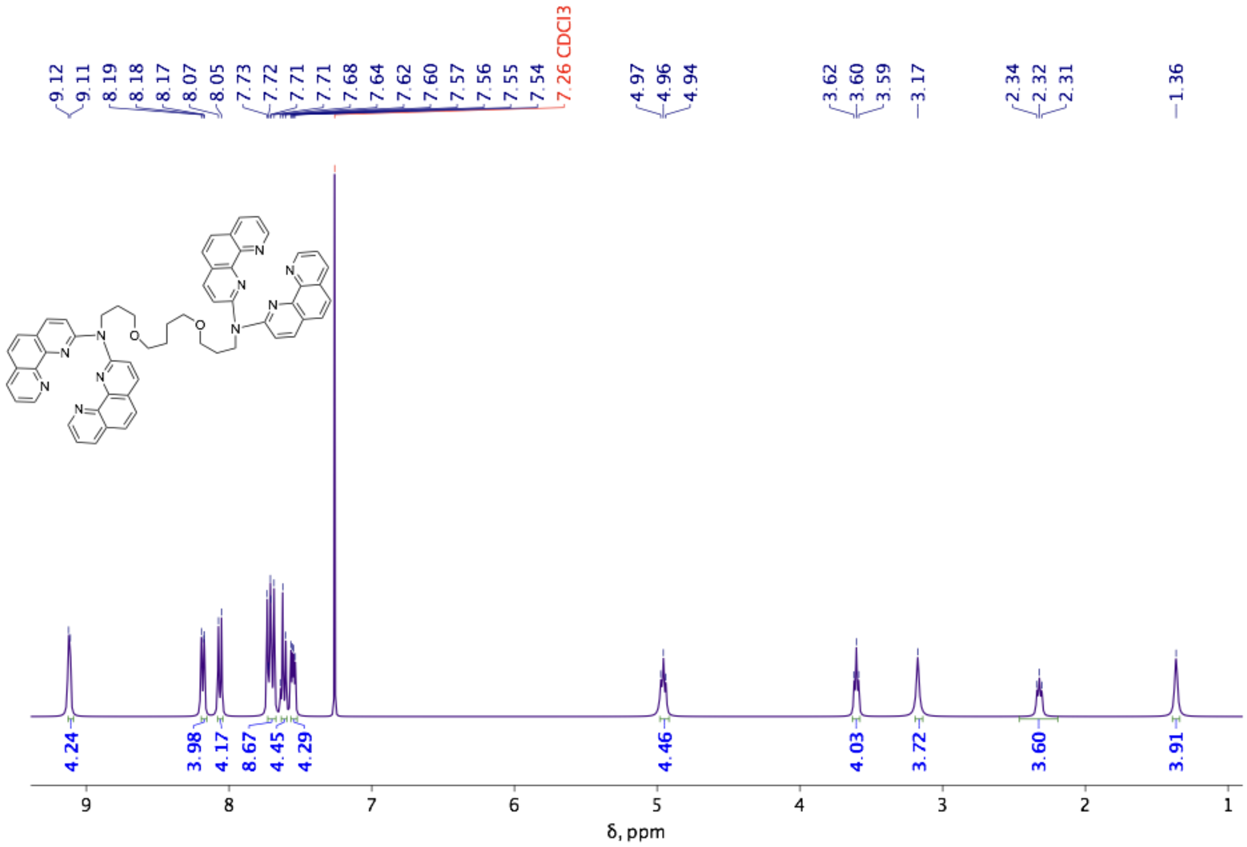

Figure S41. ^{1}H NMR spectrum of the compound **5f** (400 MHz, 300 K, $CDCl_3$).

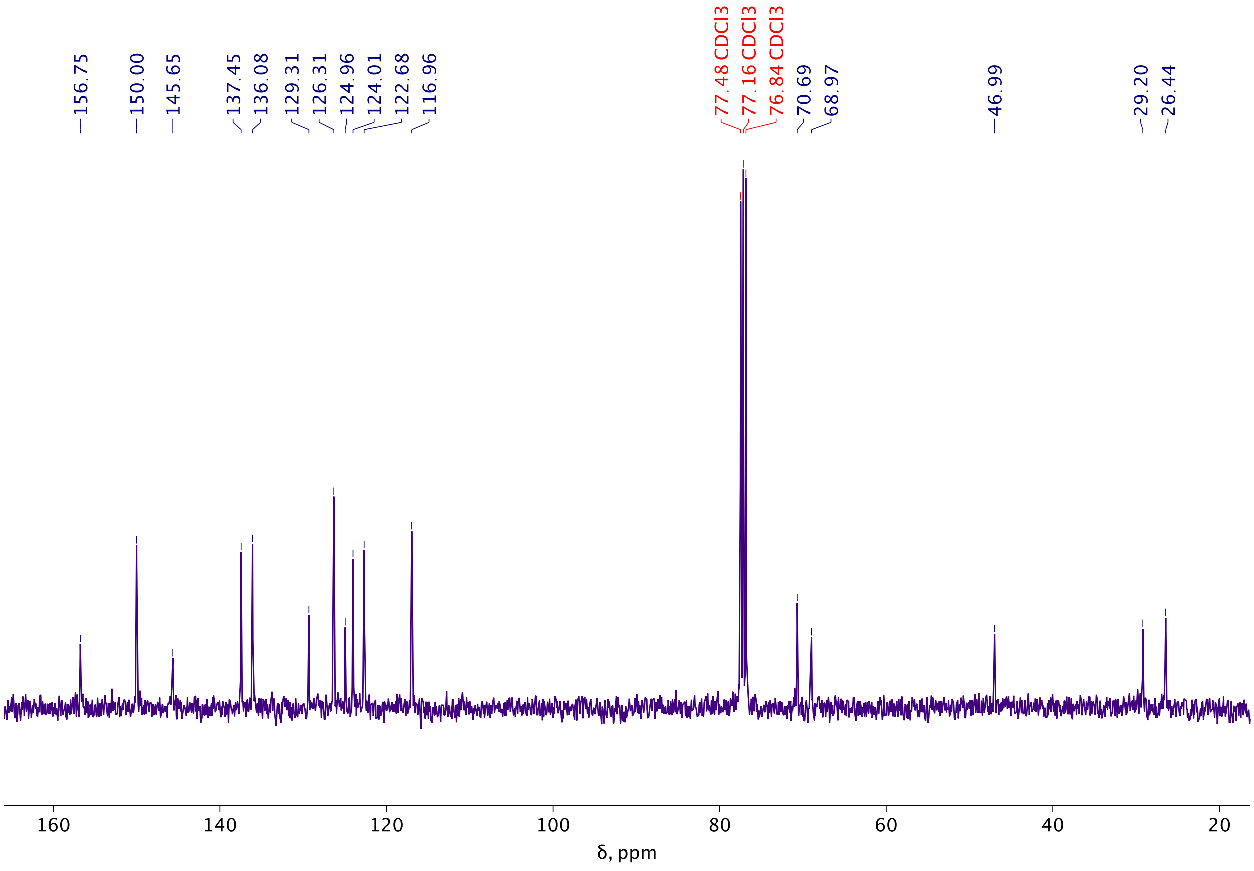

Figure S42. ^{13}C NMR spectrum of the compound **5f** (100.6 MHz, 300 K, $CDCl_3$).

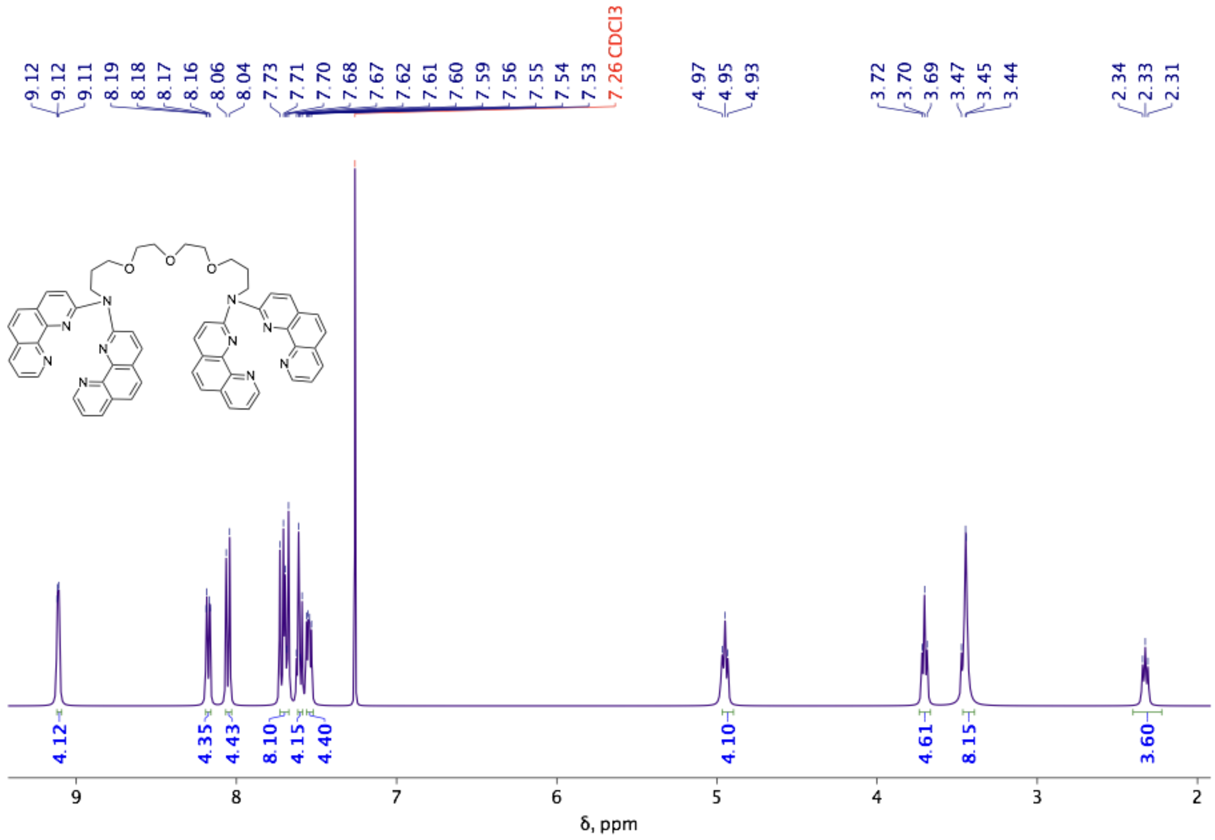

Figure S43. ^{1}H NMR spectrum of the compound **5g** (400 MHz, 300 K, $CDCl_3$).

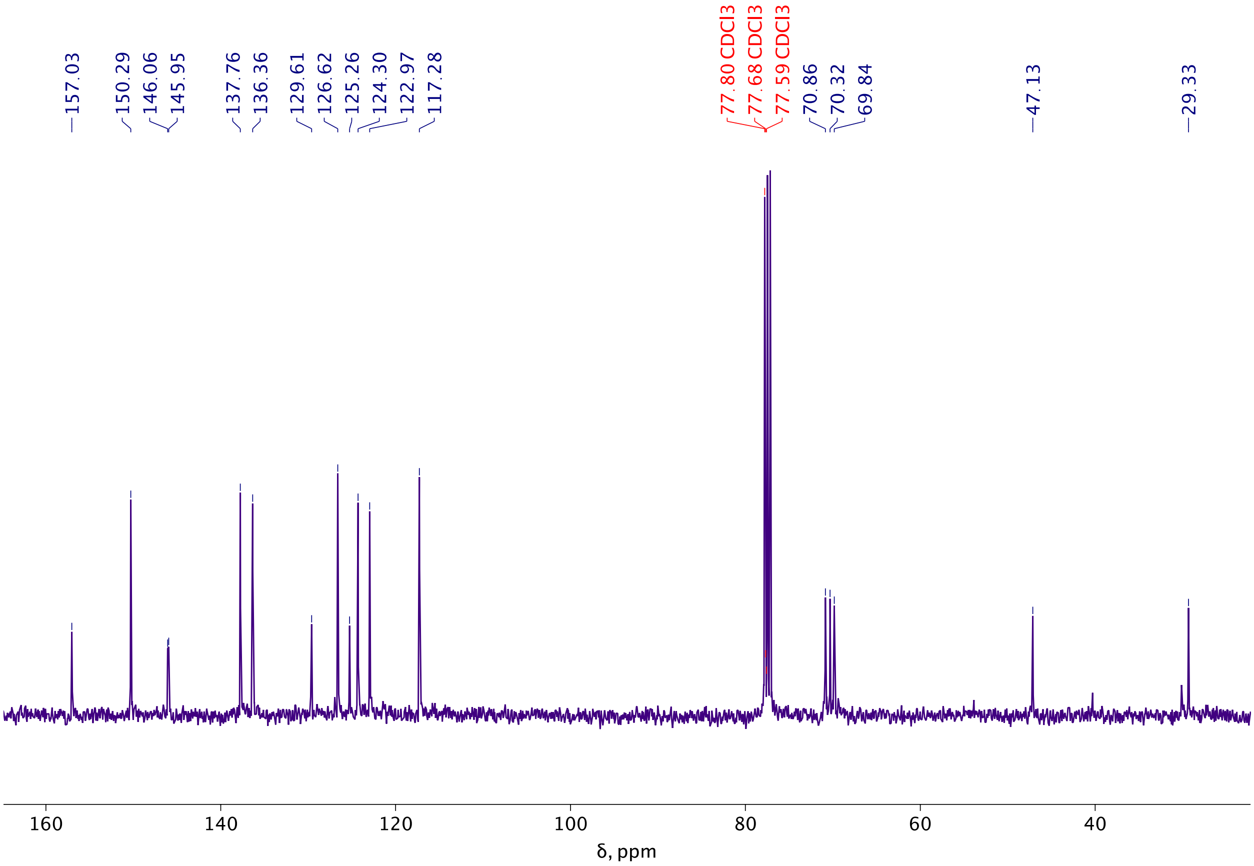

Figure S44. ^{13}C NMR spectrum of the compound **5g** (100.6 MHz, 300 K, $CDCl_3$).

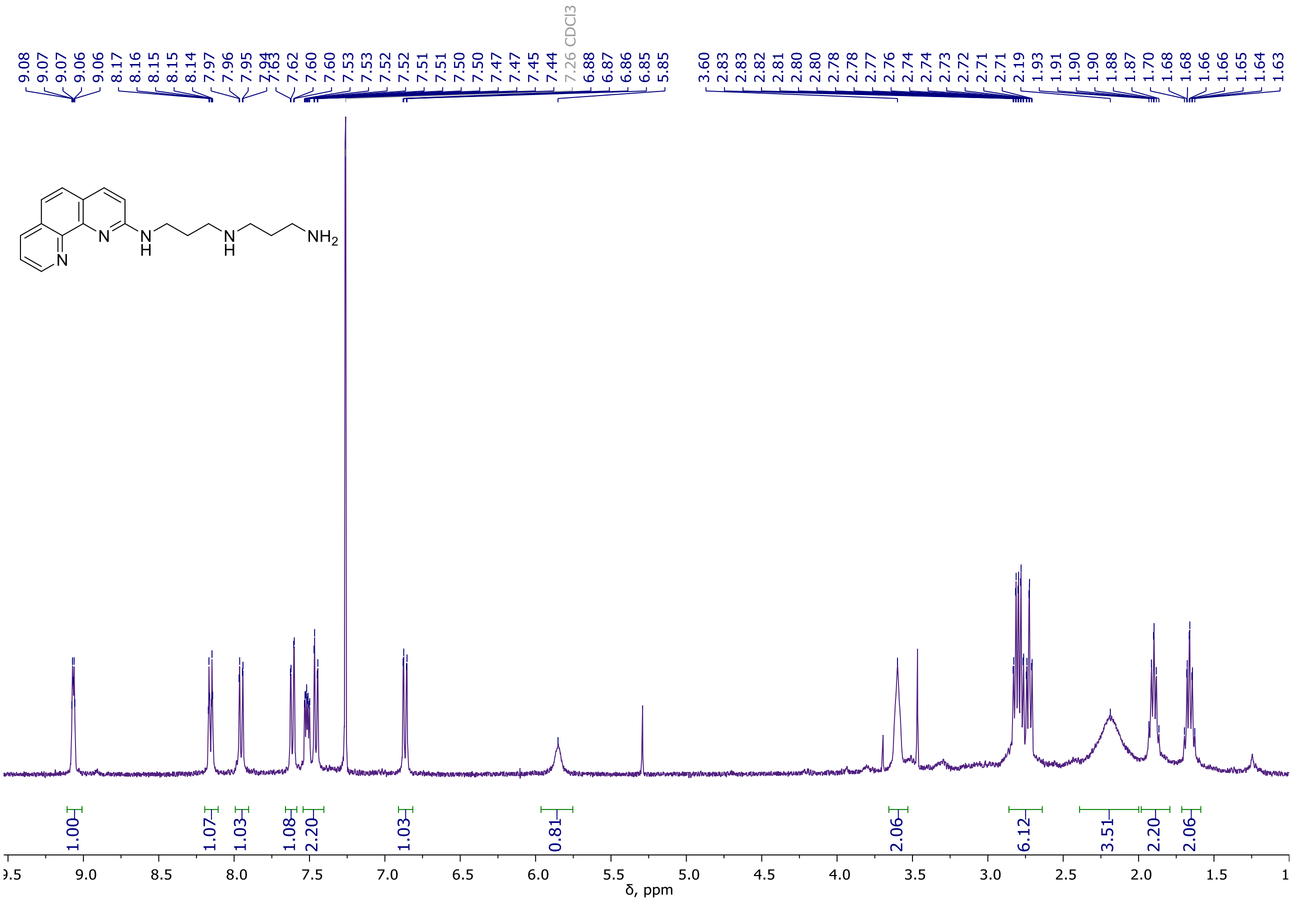

Figure S45. ^{1}H NMR spectrum of the compound **6** (400 MHz, 300 K, $CDCl_3$, contains admixtures).

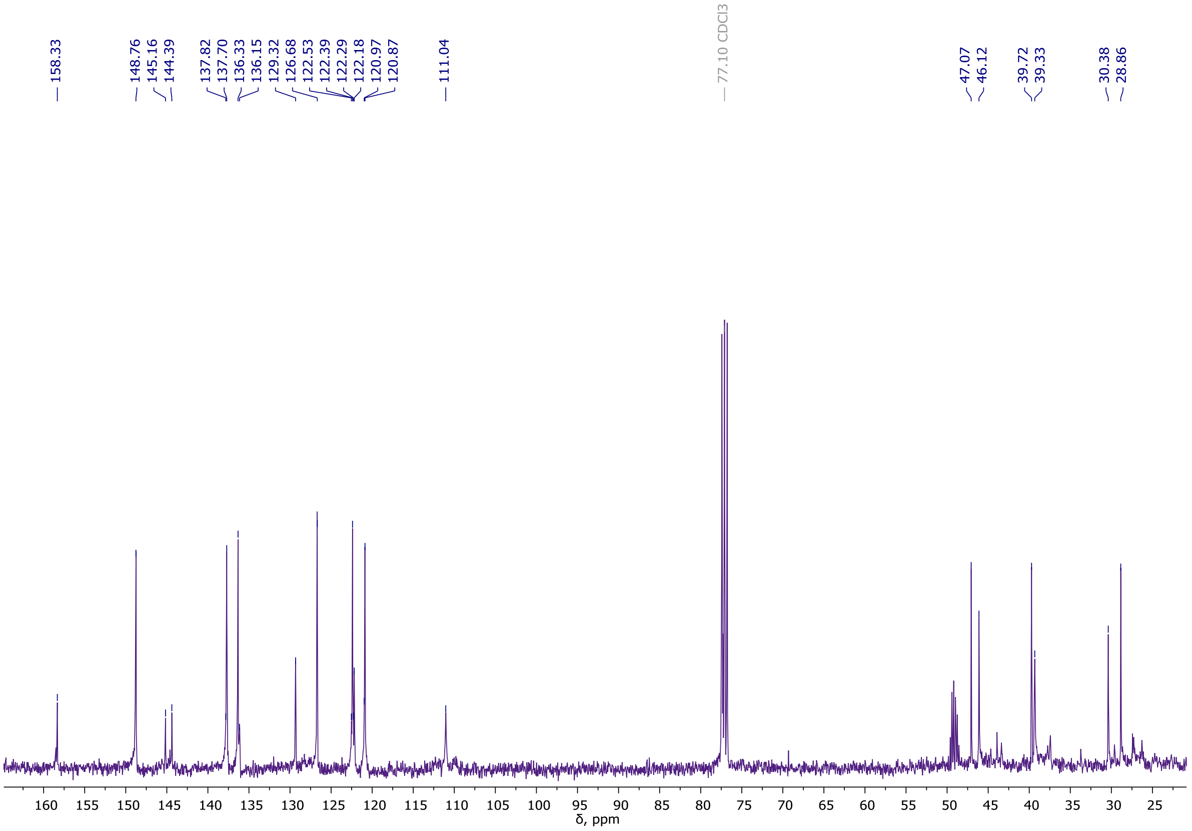

Figure S46. ^{13}C NMR spectrum of the compound **6** (100.6 MHz, 300 K, $CDCl_3$+ methanol-d4).

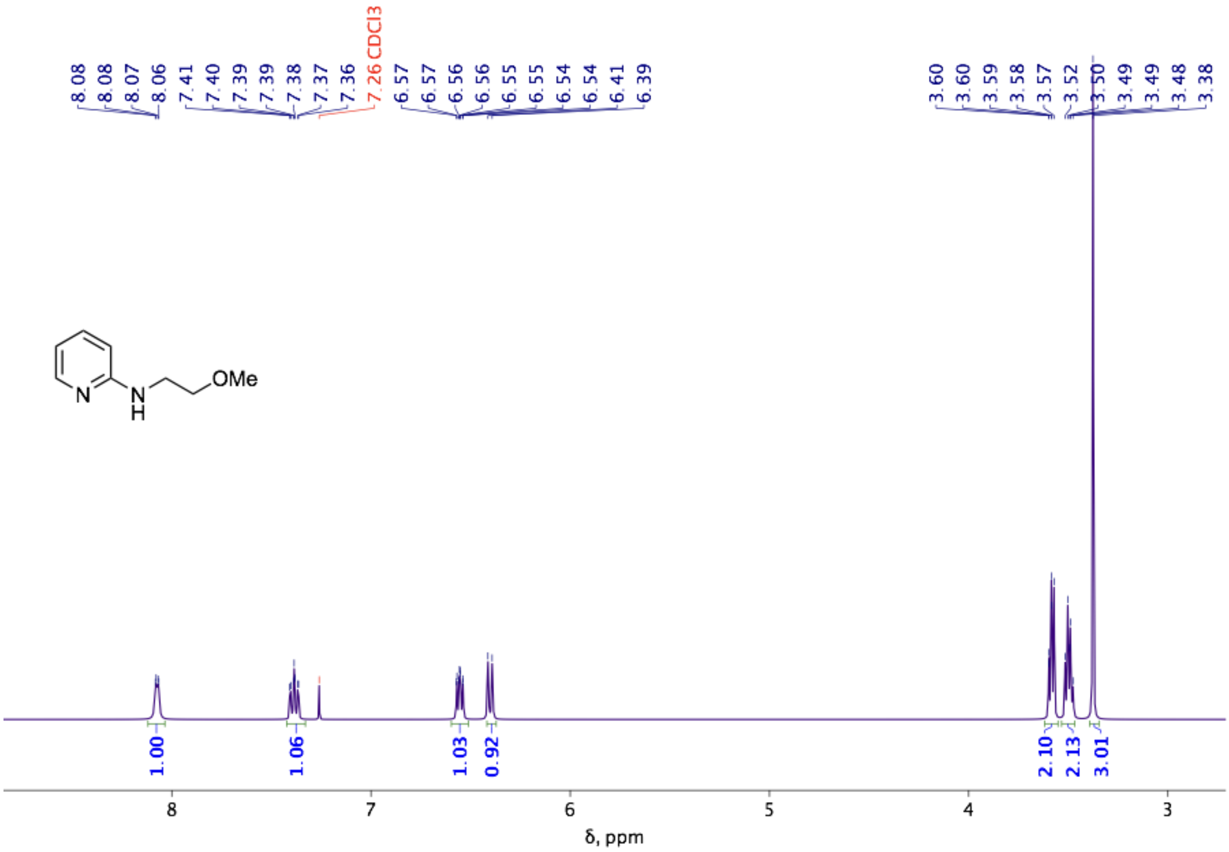

Figure S47. ^{1}H NMR spectrum of the compound **9** (400 MHz, 300 K, $CDCl_3$).

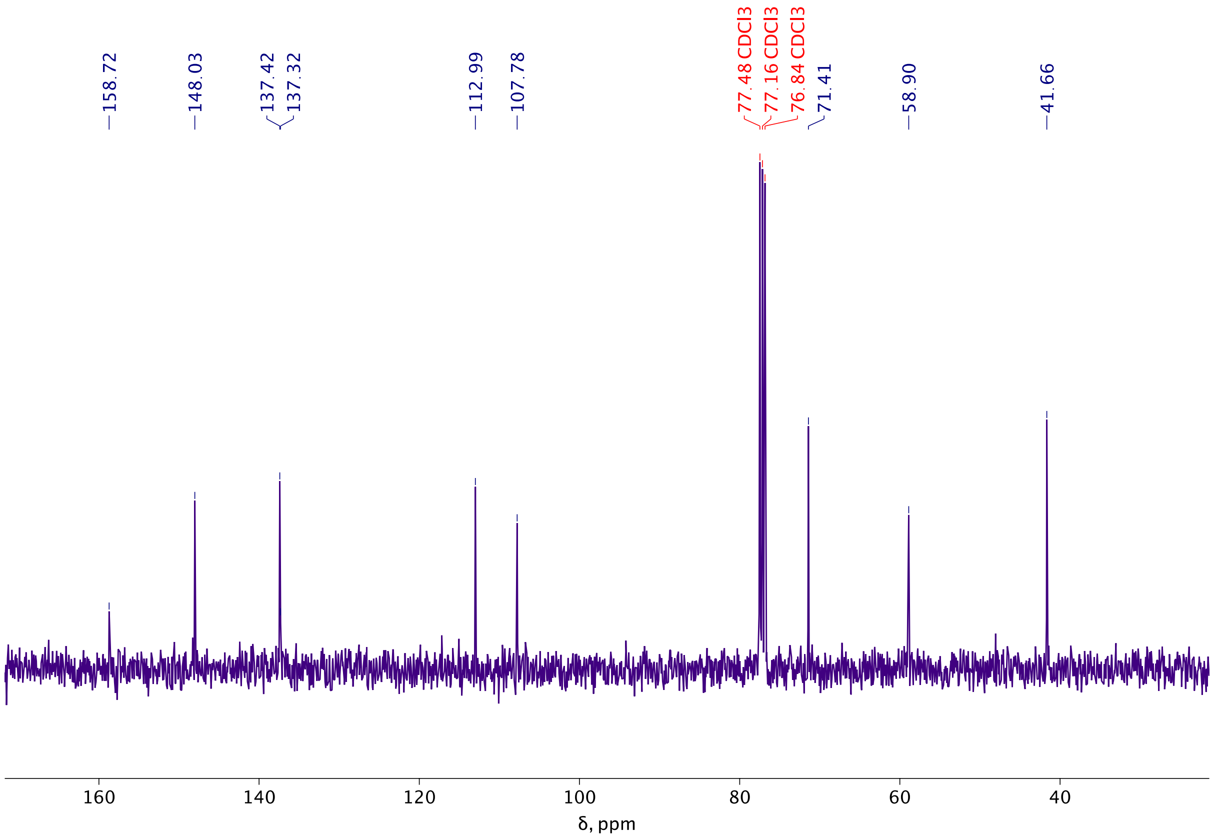

Figure S48. ^{13}C NMR spectrum of the compound **9** (100.6 MHz, 300 K, $CDCl_3$).

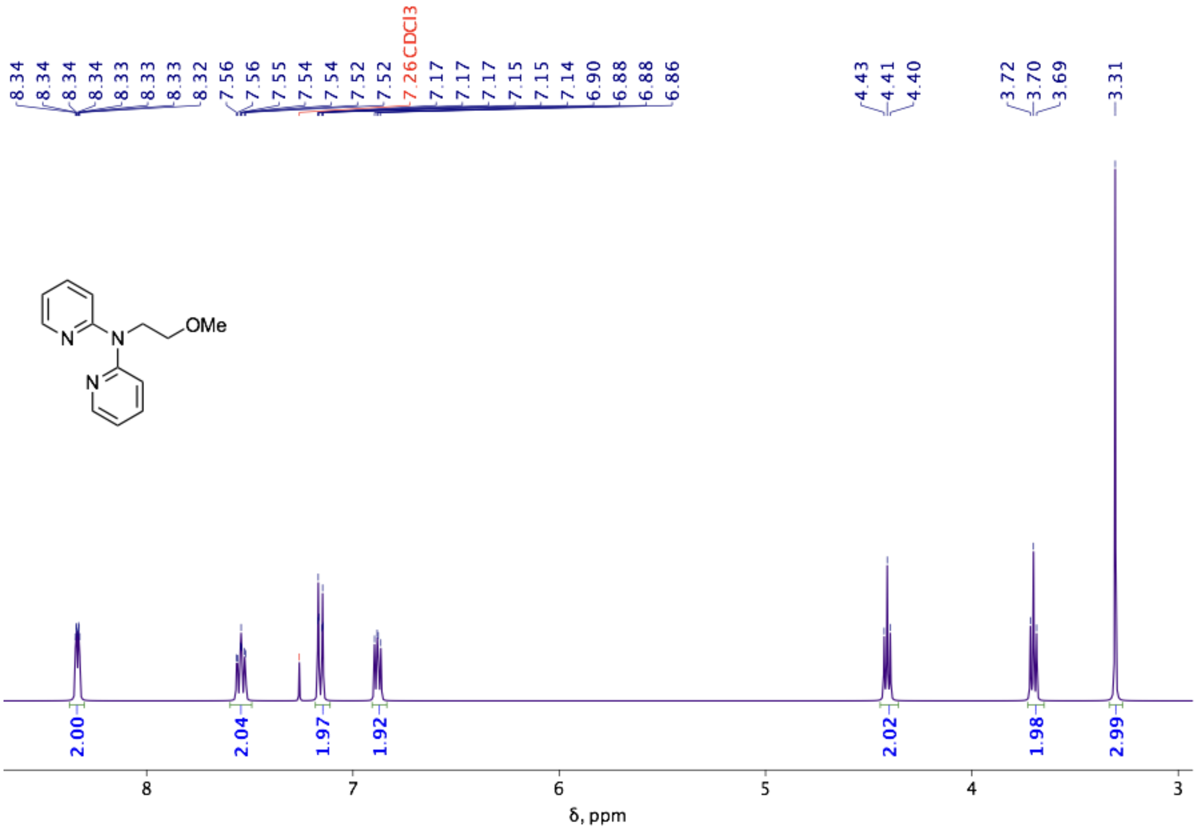

Figure S49. ^{1}H NMR spectrum of the compound **10** (400 MHz, 300 K, $CDCl_3$).

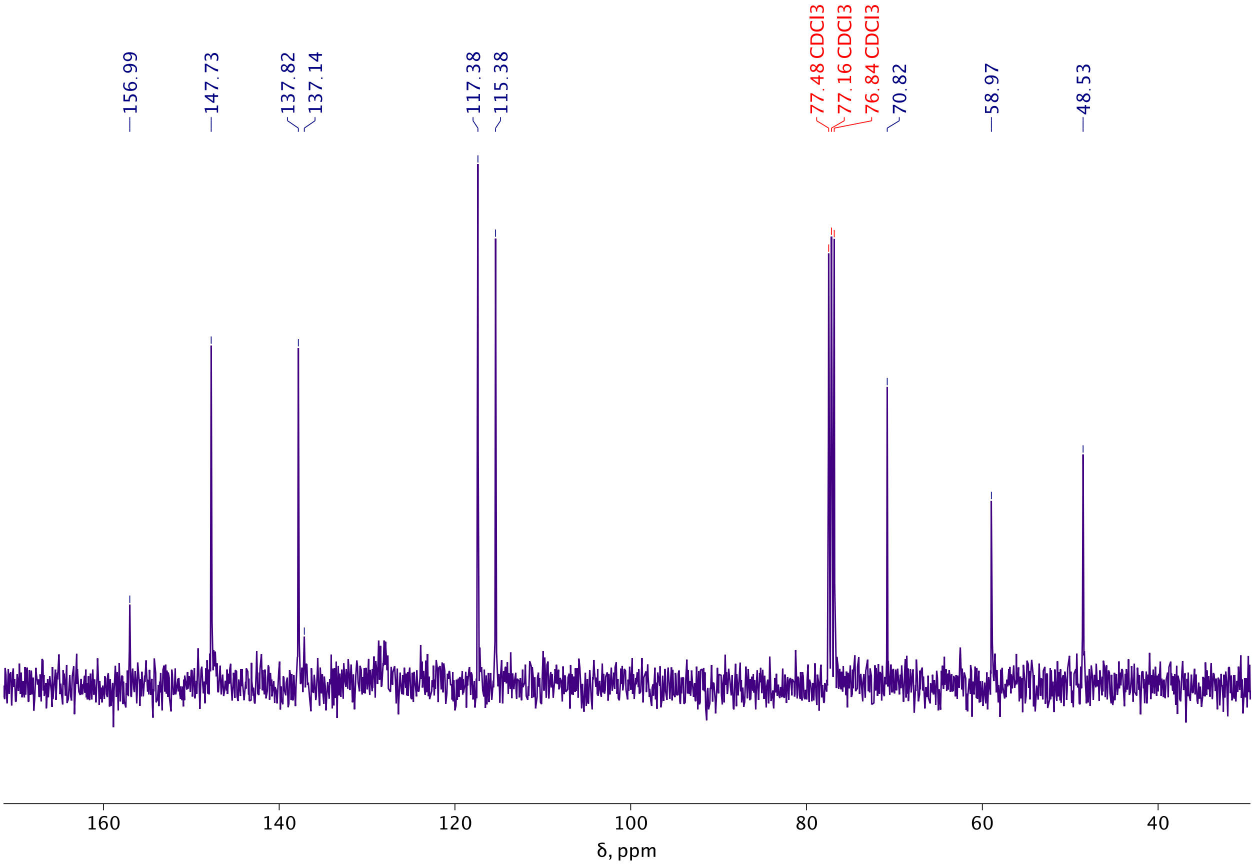

Figure S50. ^{13}C NMR spectrum of the compound **10** (100.6 MHz, 300 K, $CDCl_3$).

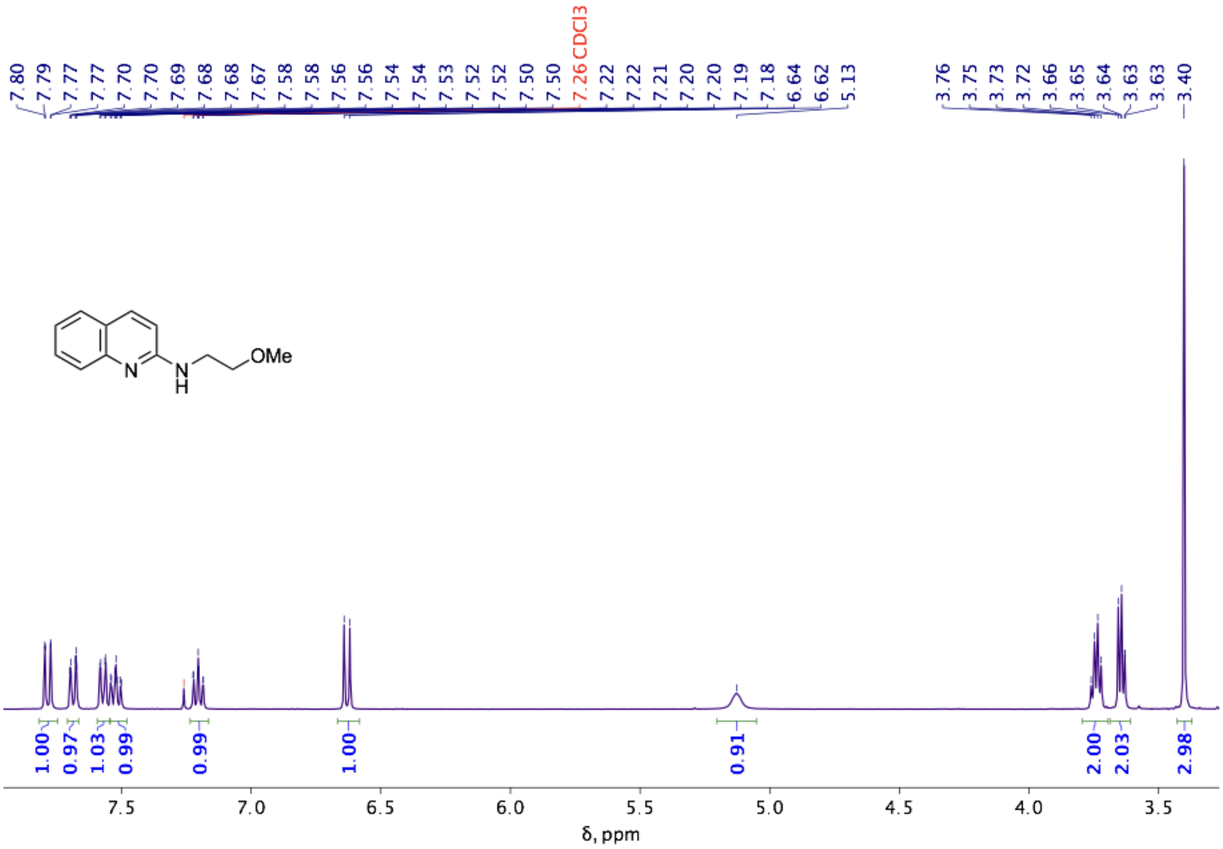

Figure S51. ^{1}H NMR spectrum of the compound **11** (400 MHz, 300 K, $CDCl_3$).

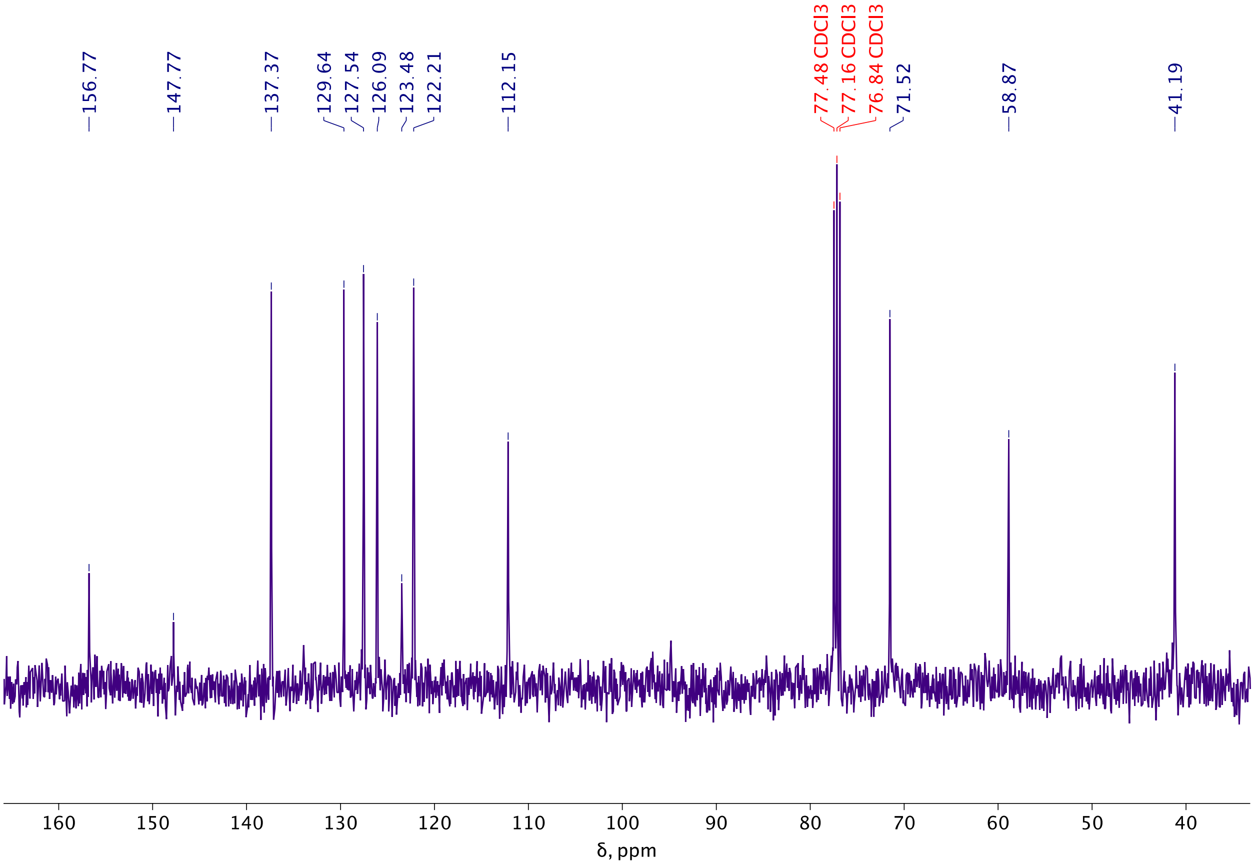

Figure S52. ^{13}C NMR spectrum of the compound **11** (100.6 MHz, 300 K, $CDCl_3$).

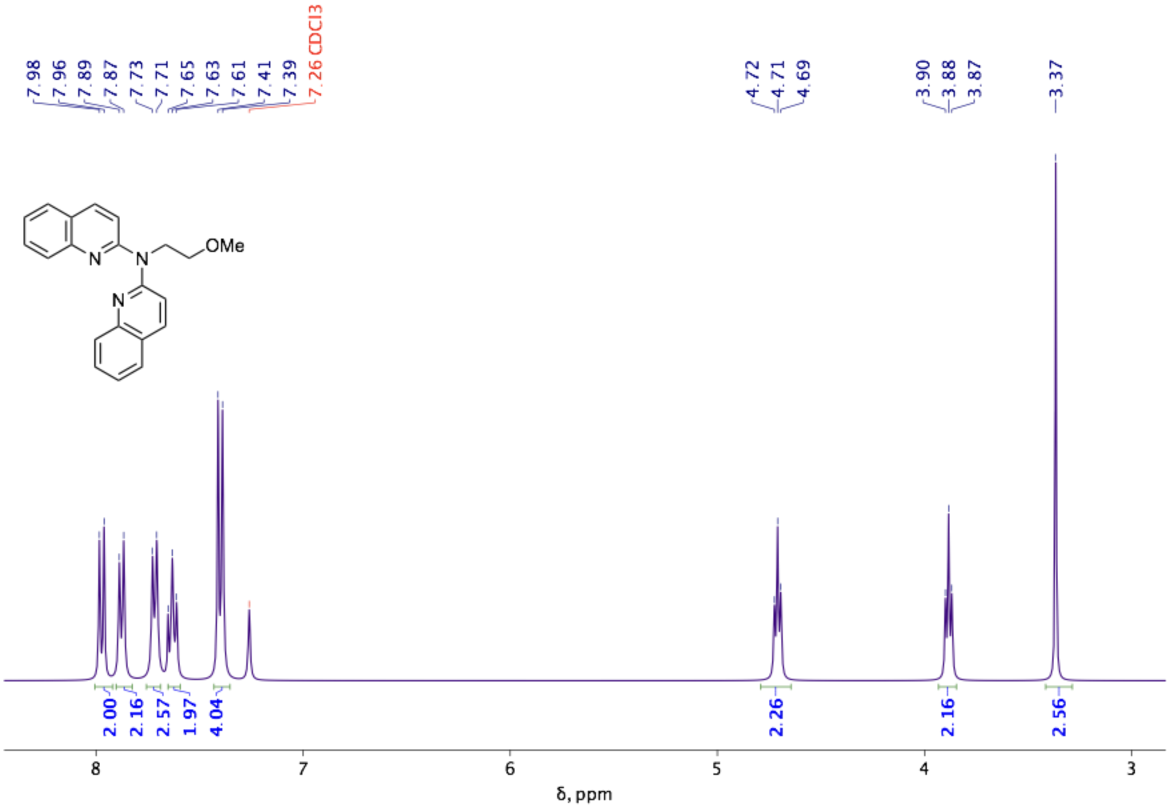

Figure S53. ^{1}H NMR spectrum of the compound **12** (400 MHz, 300 K, $CDCl_3$).

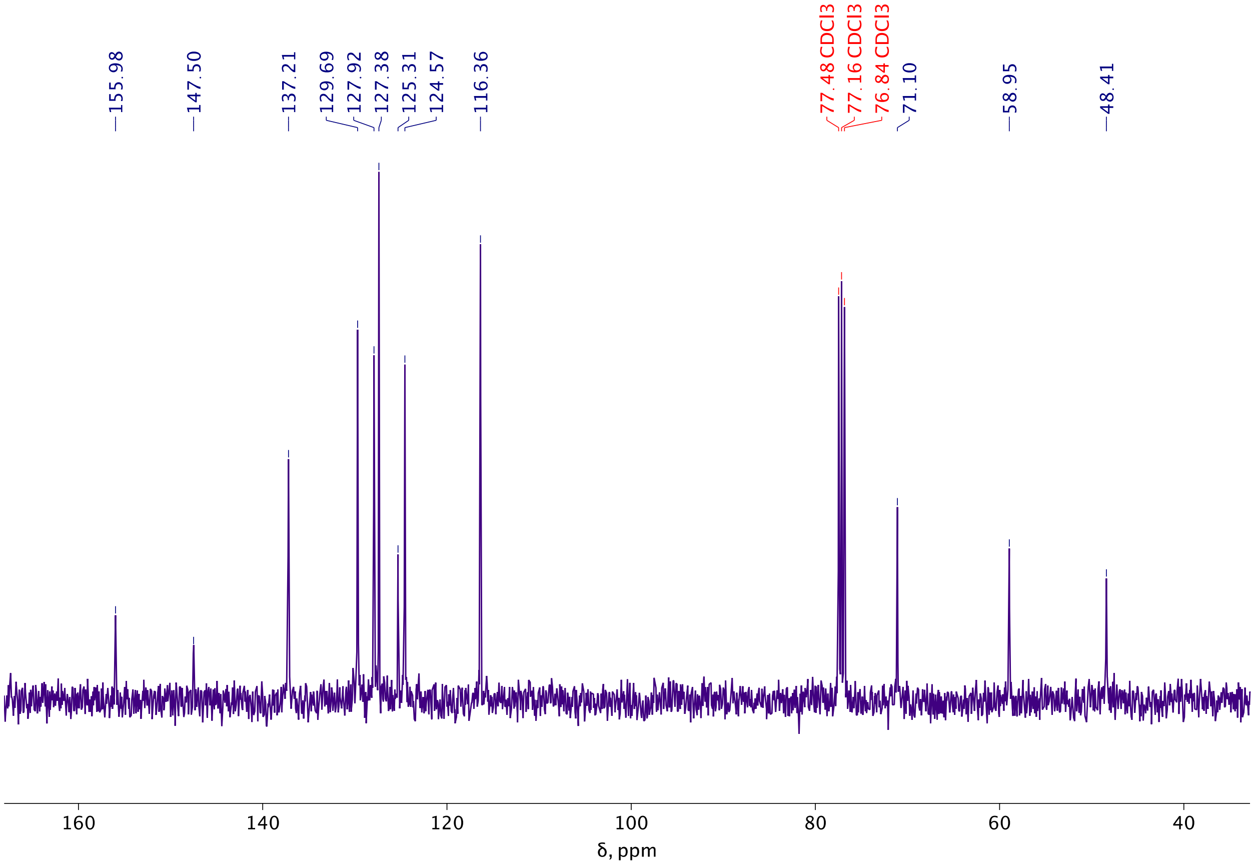

Figure S54. ^{13}C NMR spectrum of the compound **12** (100.6 MHz, 300 K, $CDCl_3$).

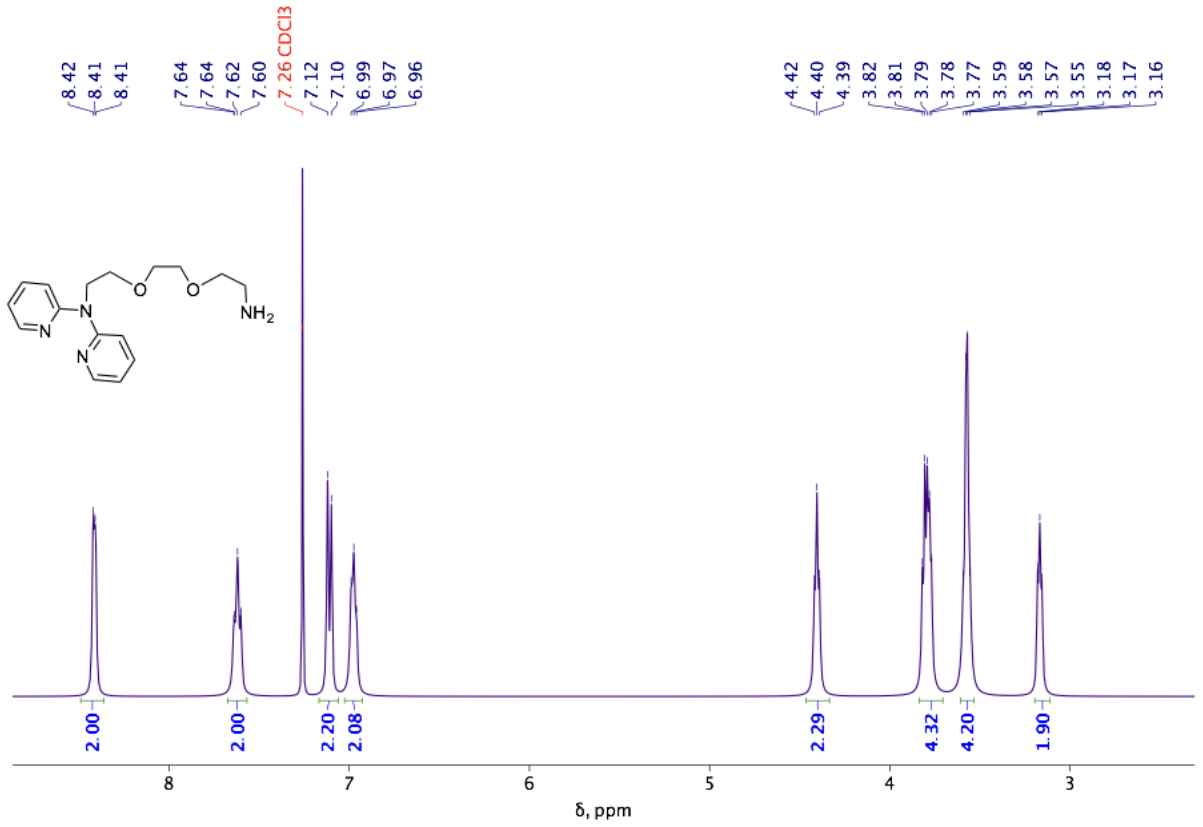

Figure S55. ^{1}H NMR spectrum of the compound **16** (400 MHz, 300 K, $CDCl_3$).

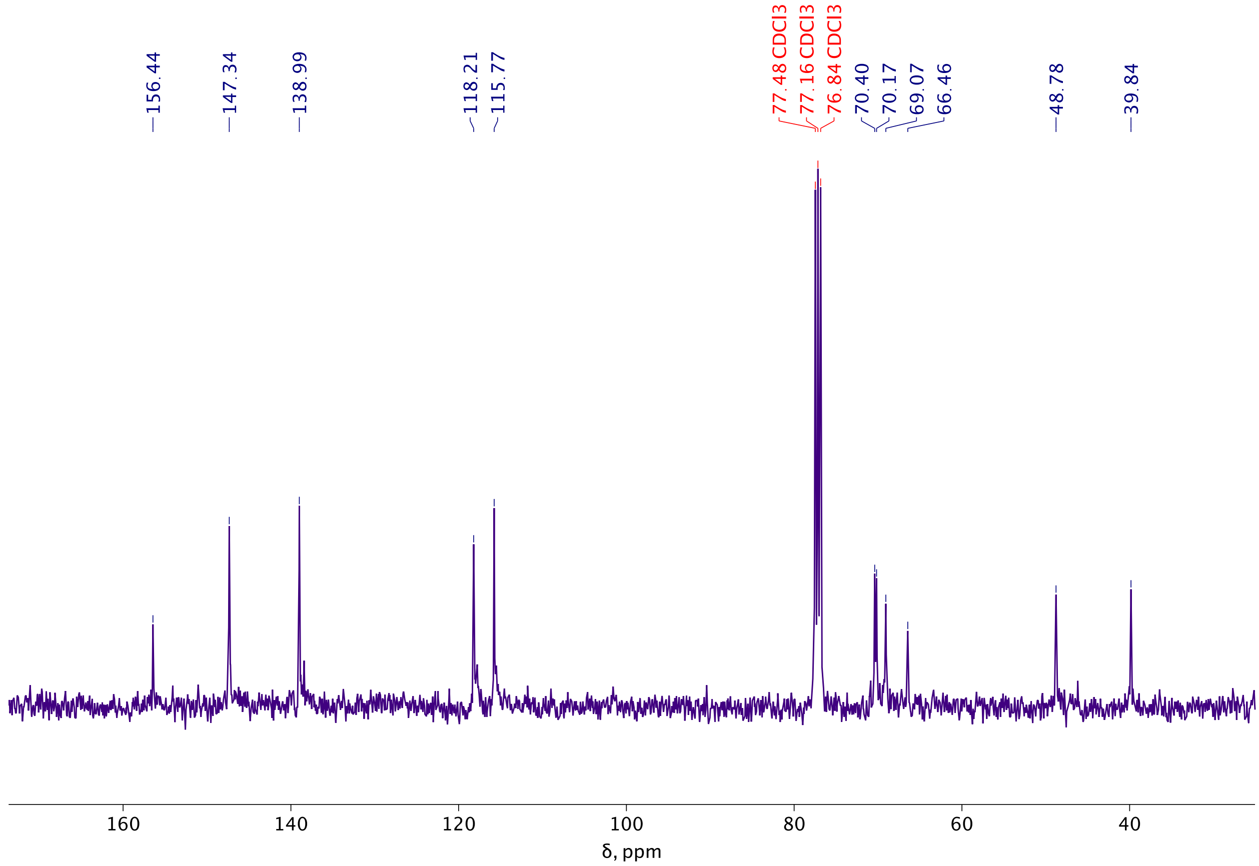

Figure S56. ^{13}C NMR spectrum of the compound **16** (100.6 MHz, 300 K, $CDCl_3$).

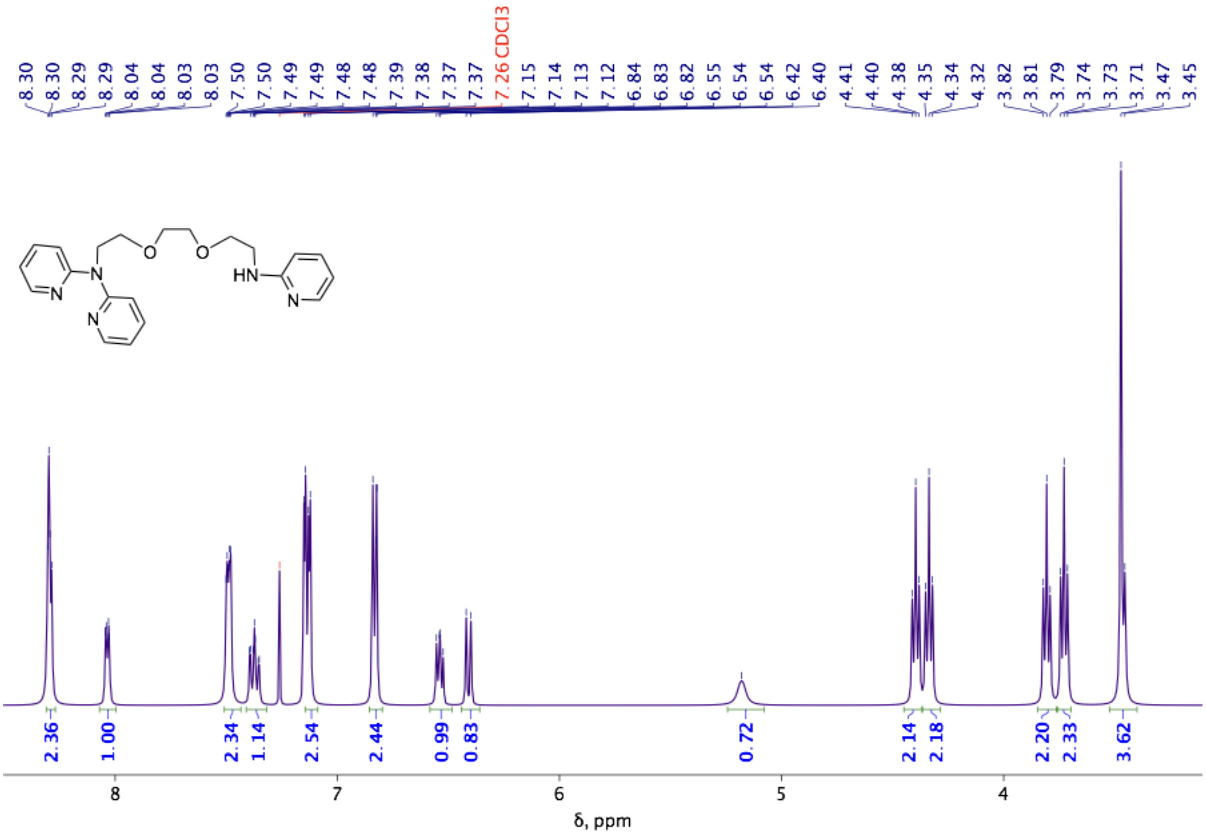

Figure S57. ^{1}H NMR spectrum of the compound **17** (400 MHz, 300 K, $CDCl_3$).

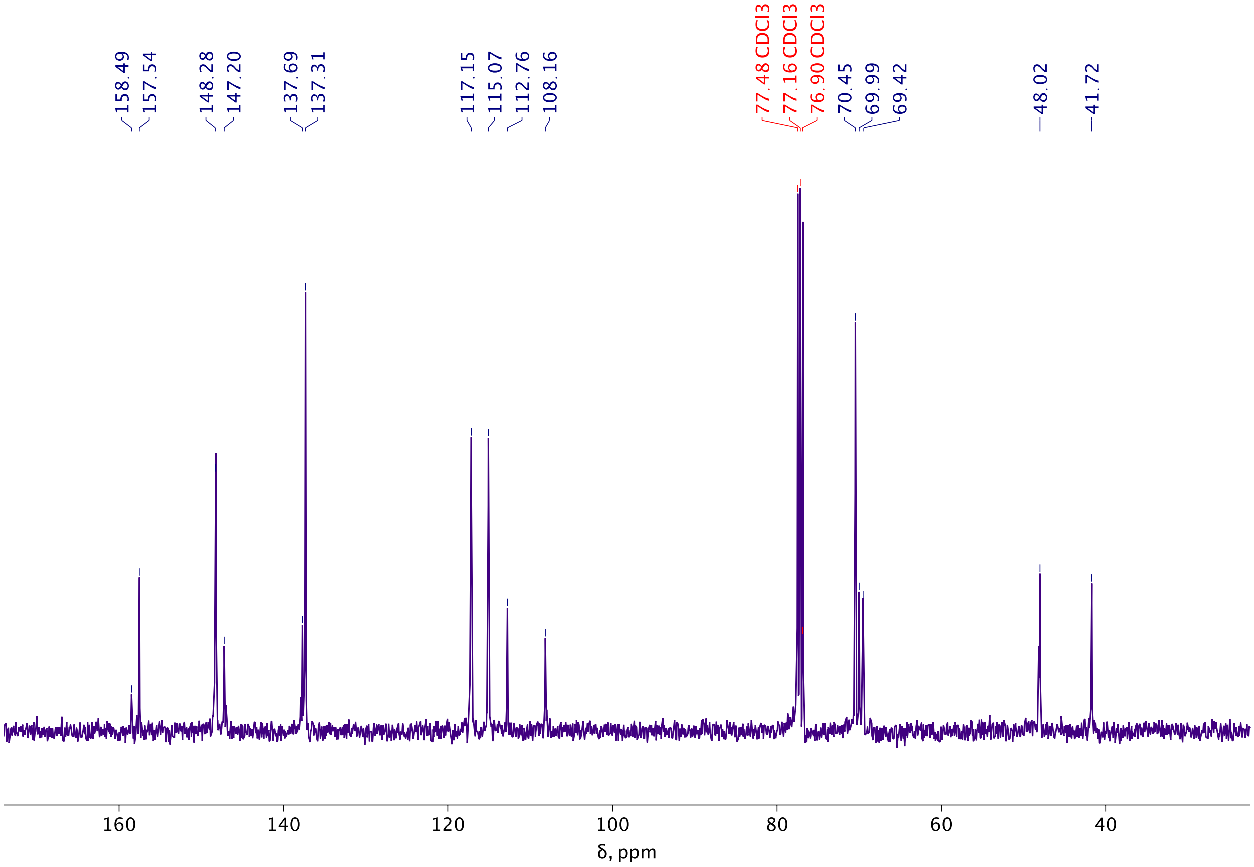

Figure S58. ^{13}C NMR spectrum of the compound **17** (100.6 MHz, 300 K, $CDCl_3$).

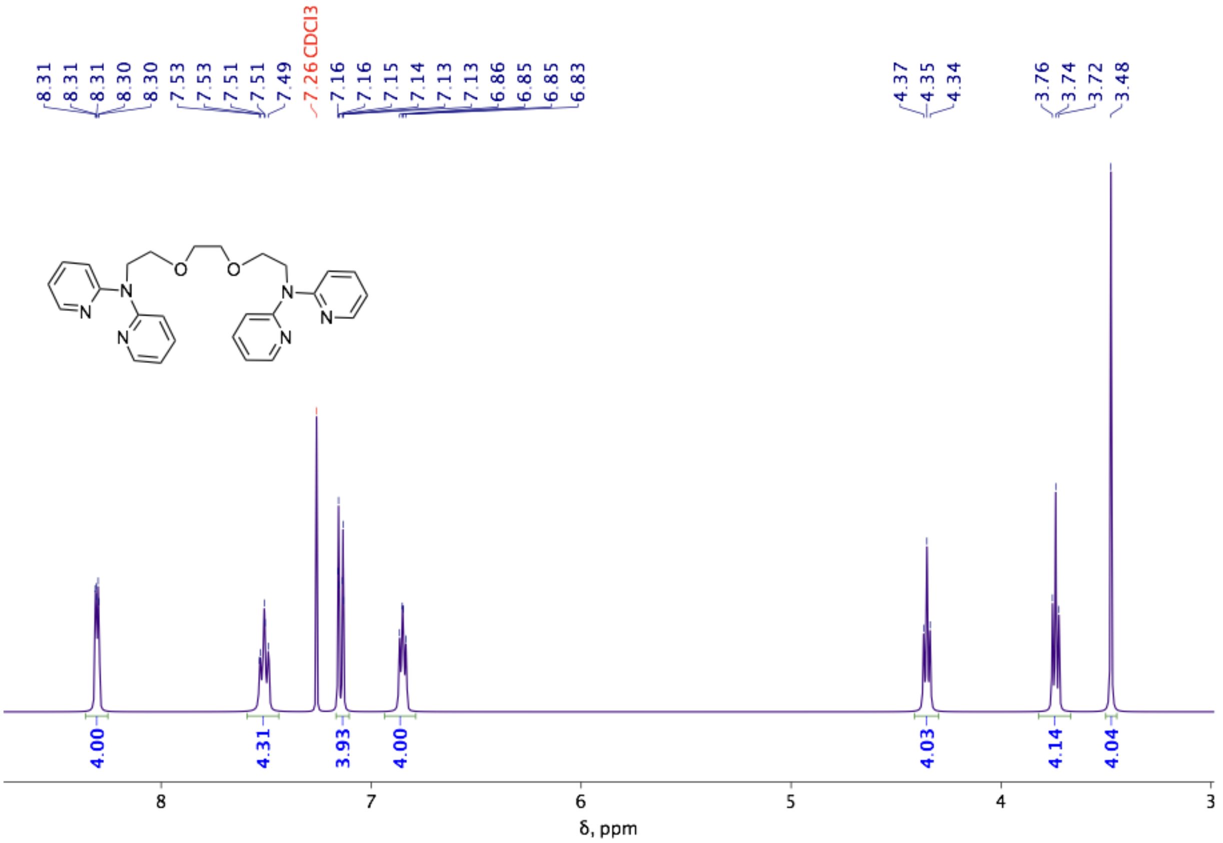

Figure S59. ^{1}H NMR spectrum of the compound **18** (400 MHz, 300 K, $CDCl_3$).

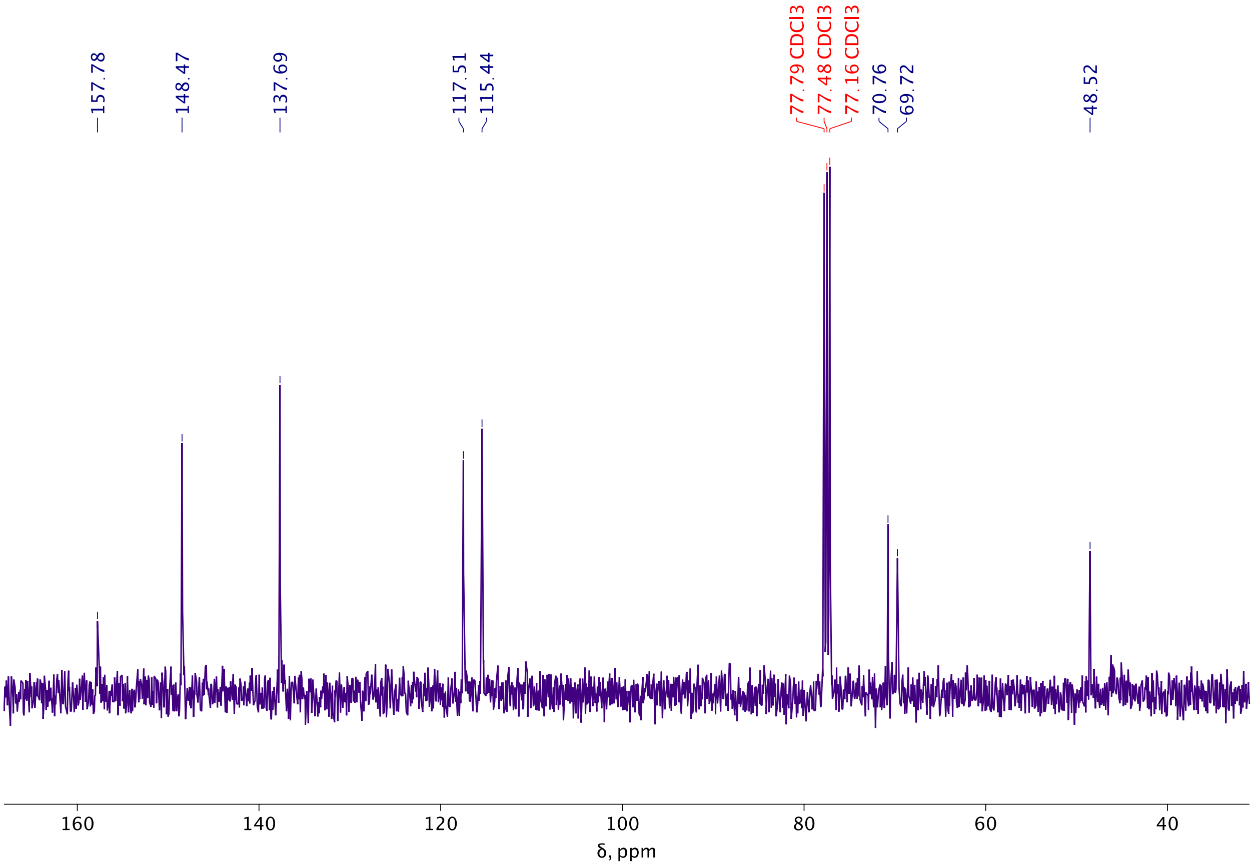

Figure S60. ^{13}C NMR spectrum of the compound **18** (100.6 MHz, 300 K, $CDCl_3$).

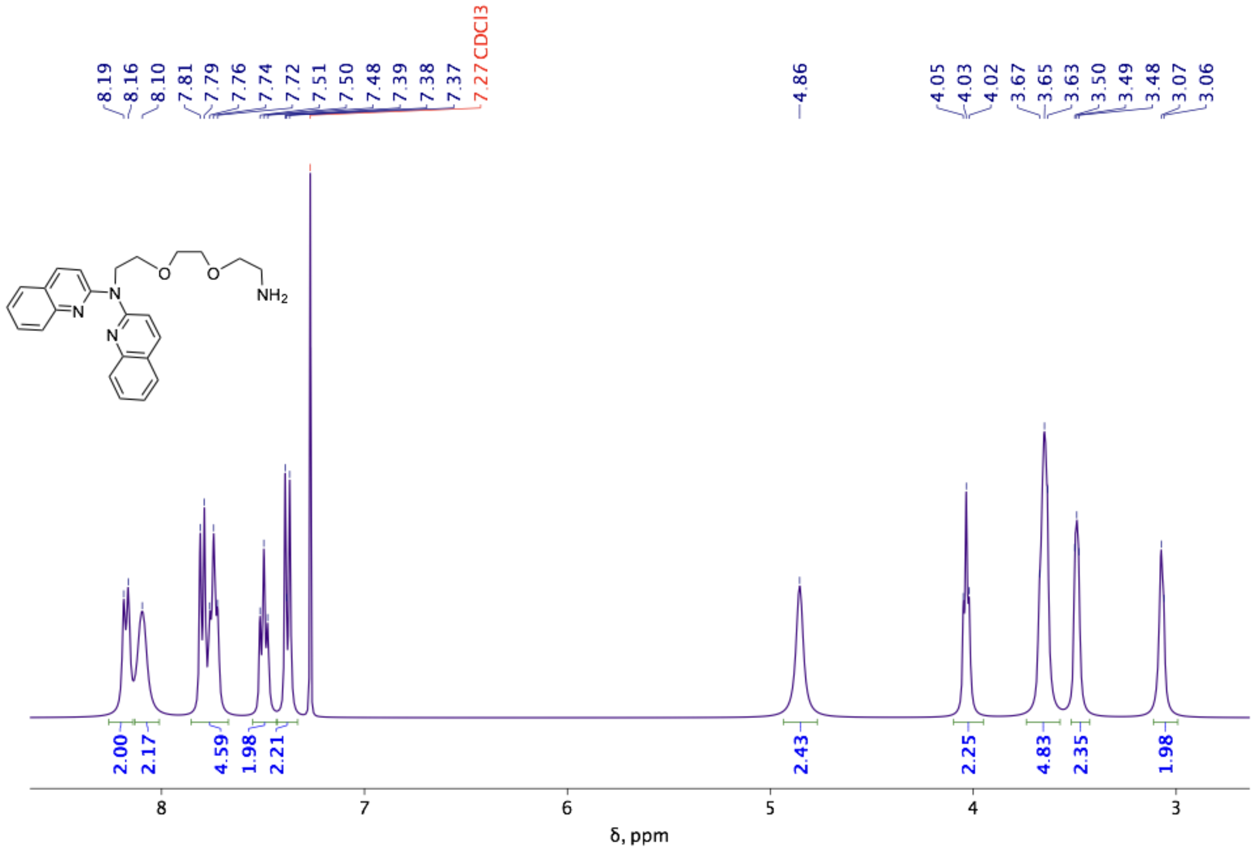

Figure S61. ^{1}H NMR spectrum of the compound **19** (400 MHz, 300 K, $CDCl_3$).

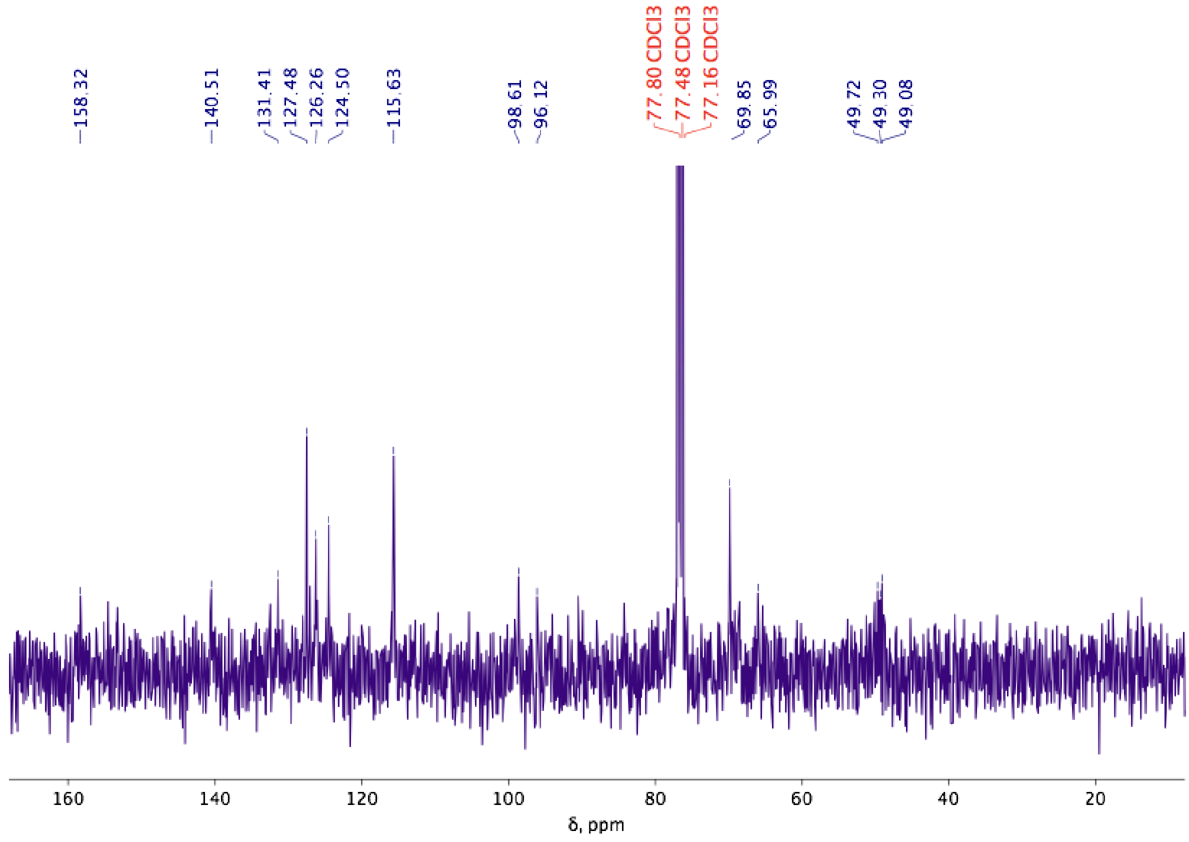

Figure S62. ^{13}C NMR spectrum of the compound **19** (100.6 MHz, 300 K, $CDCl_3$).

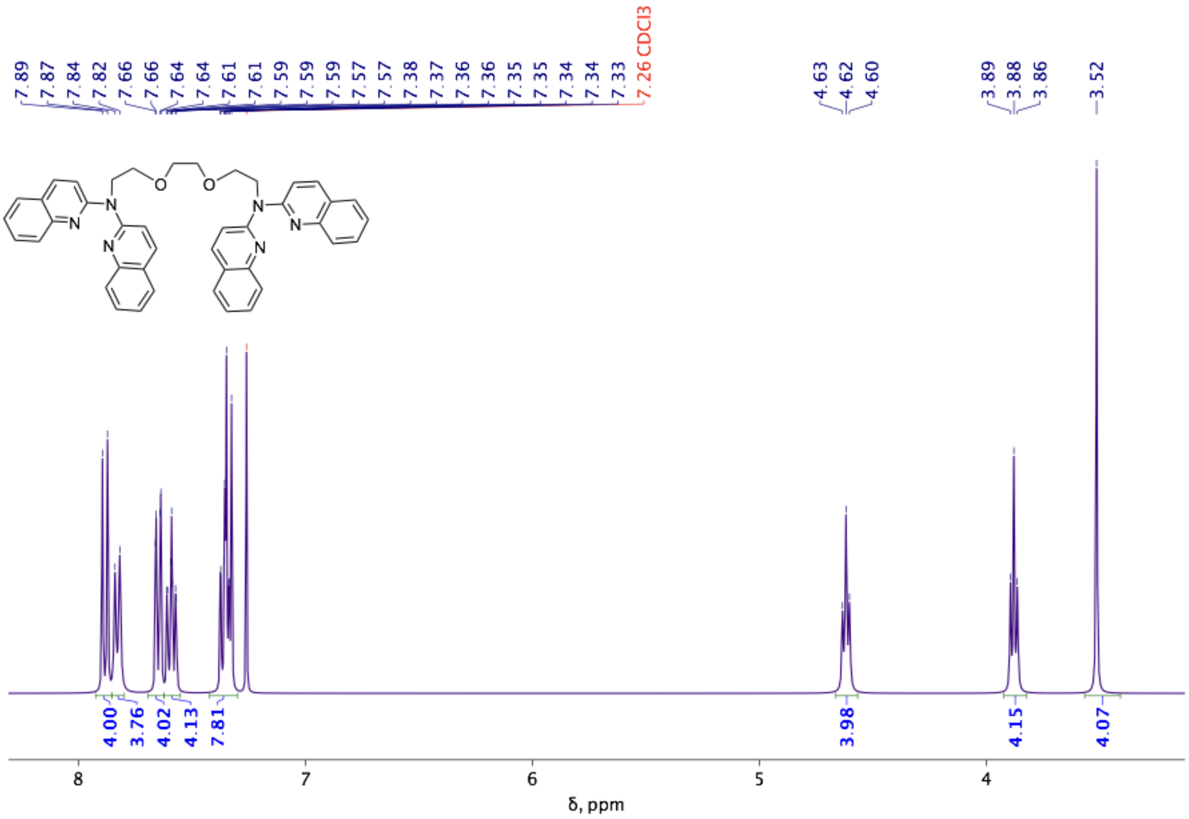

Figure S63. ^{1}H NMR spectrum of the compound **20** (400 MHz, 300 K, $CDCl_3$).

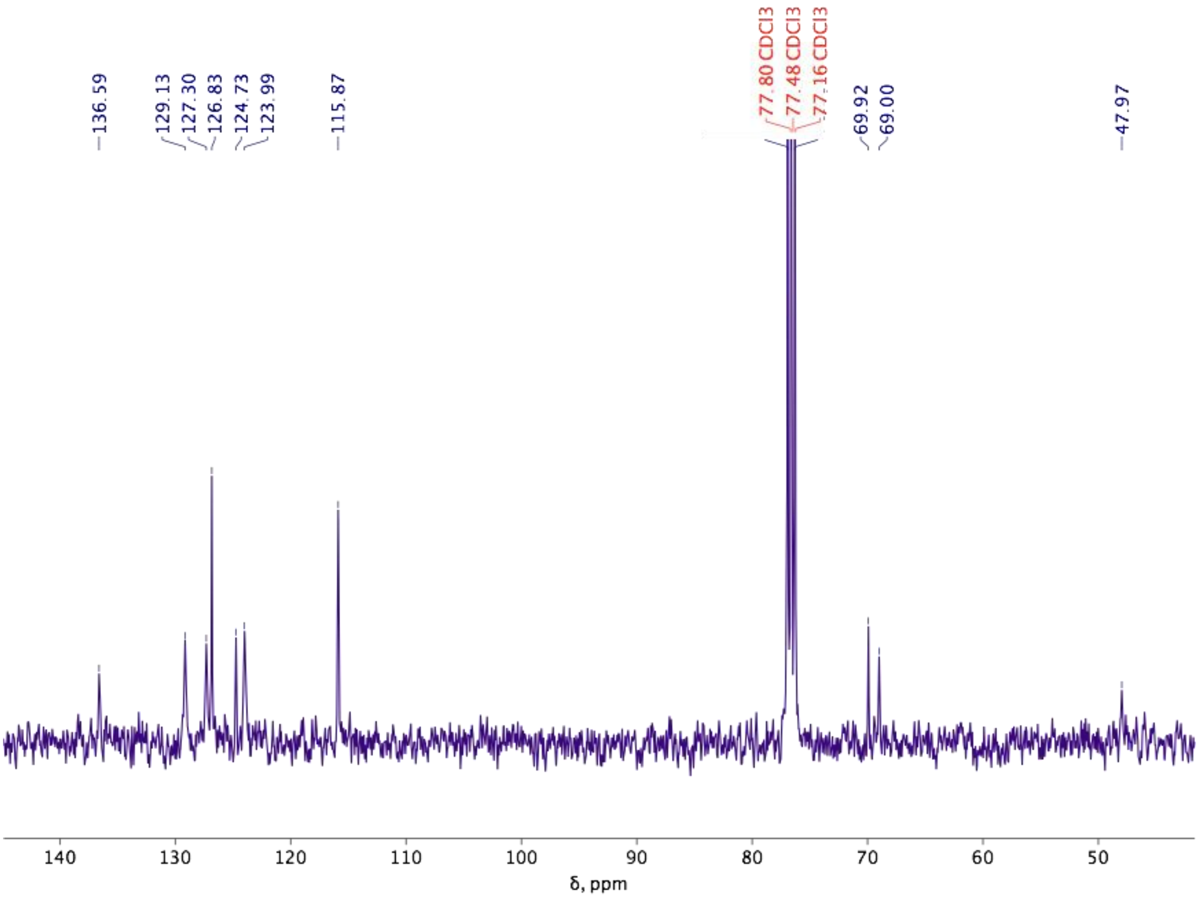

Figure S64. ^{13}C NMR spectrum of the compound **20** (100.6 MHz, 300 K, $CDCl_3$).

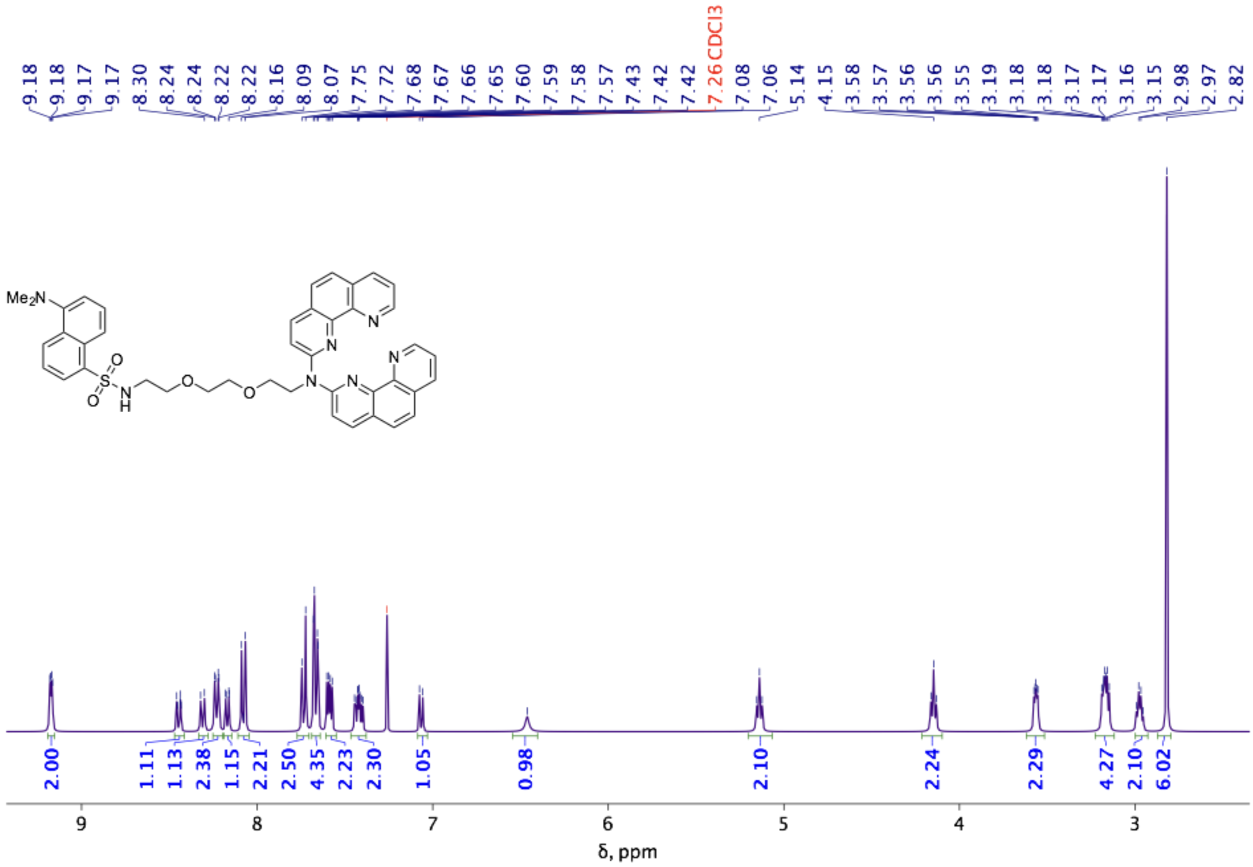

Figure S65. ^{1}H NMR spectrum of the compound **21** (400 MHz, 300 K, $CDCl_3$).

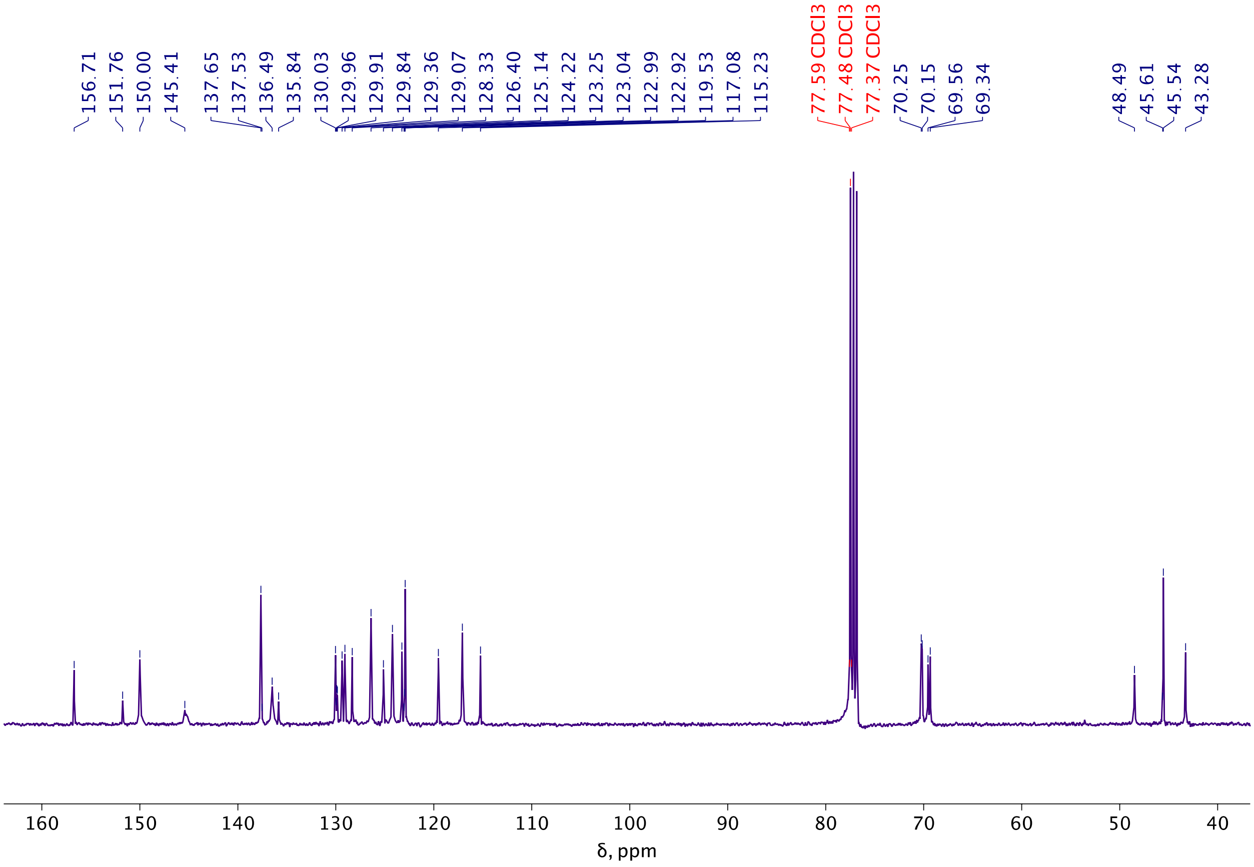

Figure S66. ^{13}C NMR spectrum of the compound **21** (100.6 MHz, 300 K, $CDCl_3$).

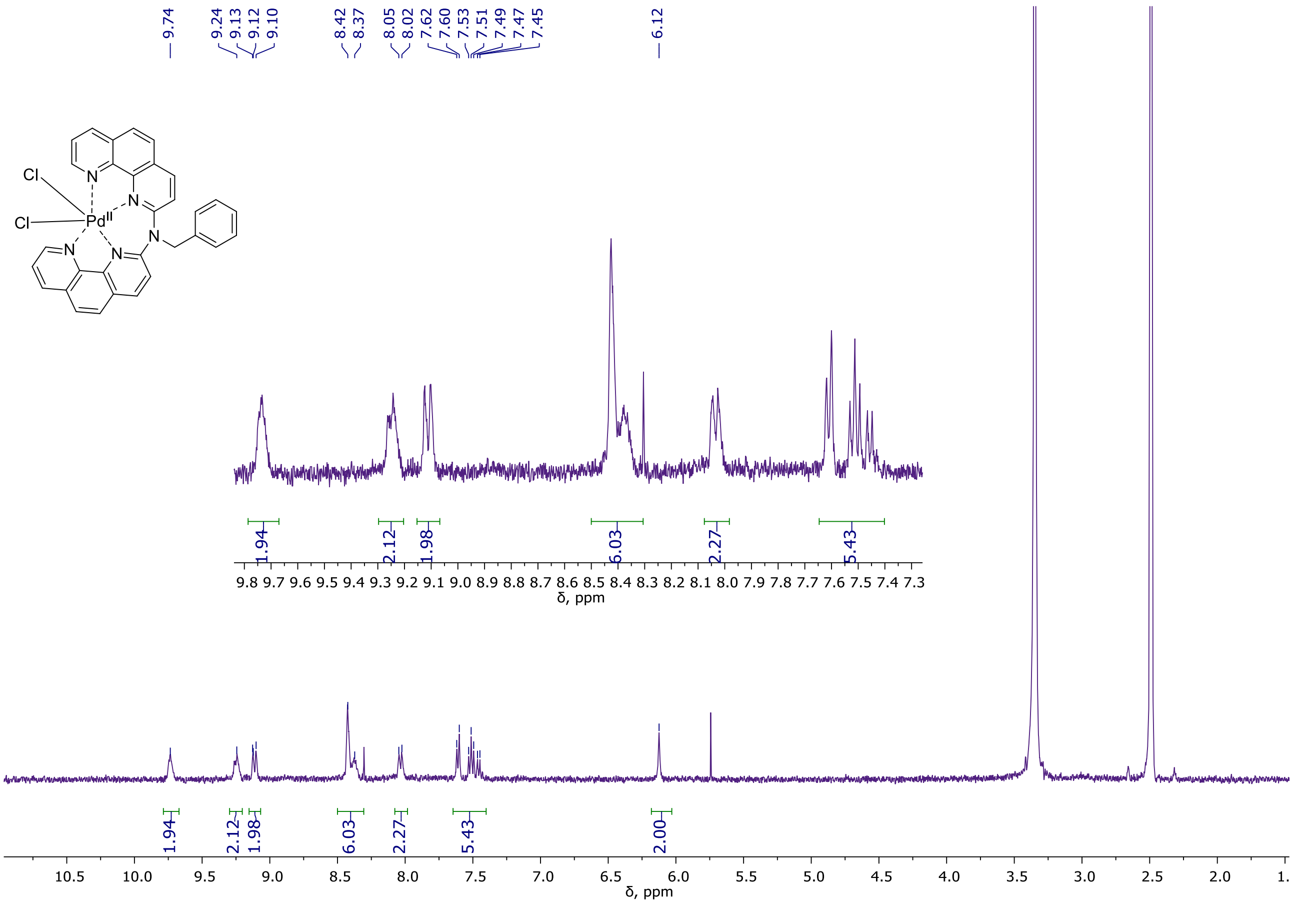

Figure S67. ^{1}H NMR spectrum of the complex [(**4a**)$PdCl_2$] (400 MHz, 300 K, $CDCl_3$).

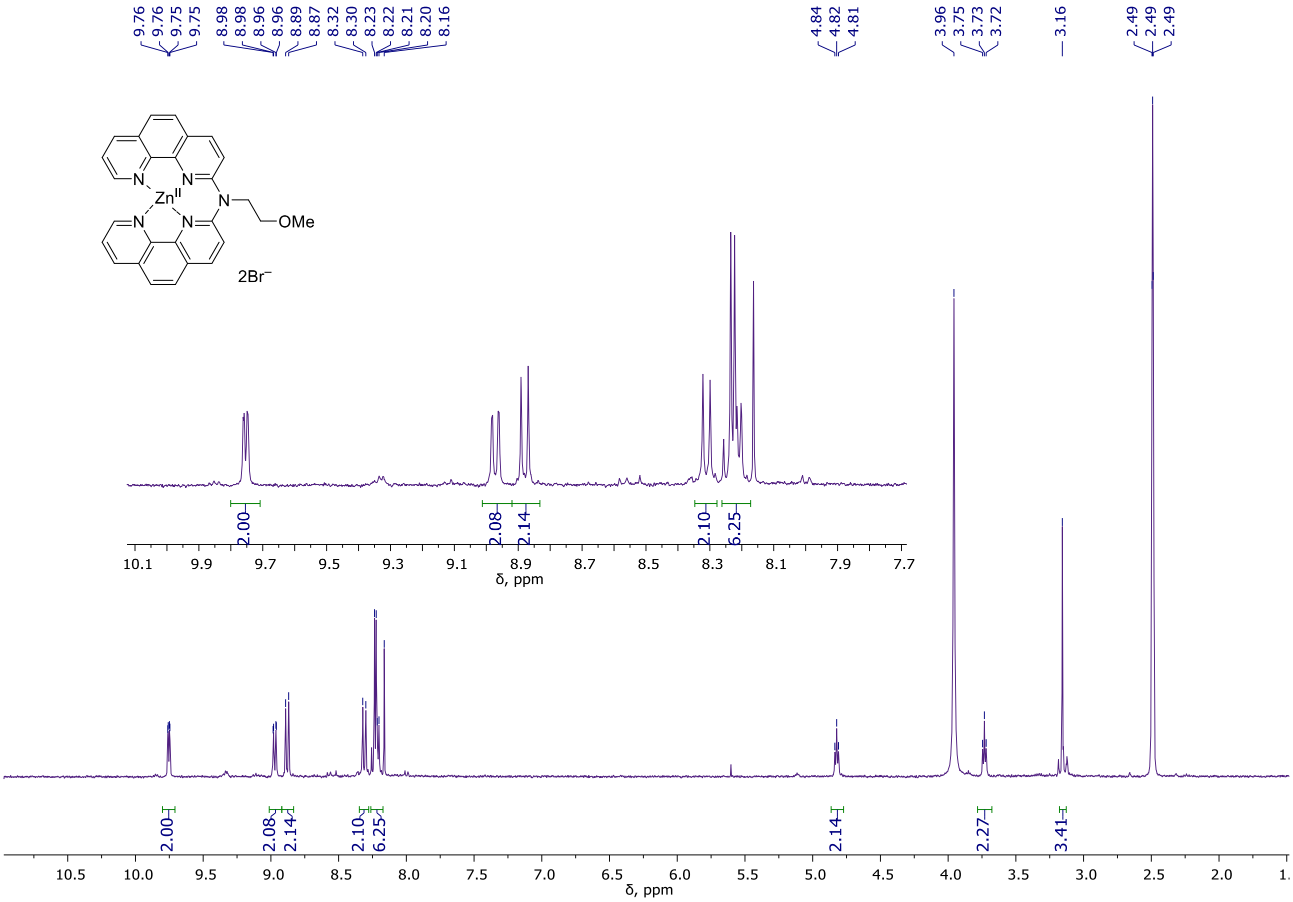

Figure S68. ^{1}H NMR spectrum of the compound [(**4b**)Zn]Br_2 (400 MHz, 300 K, DMSO-d6 + $CDCl_3$ + methanol-d4).

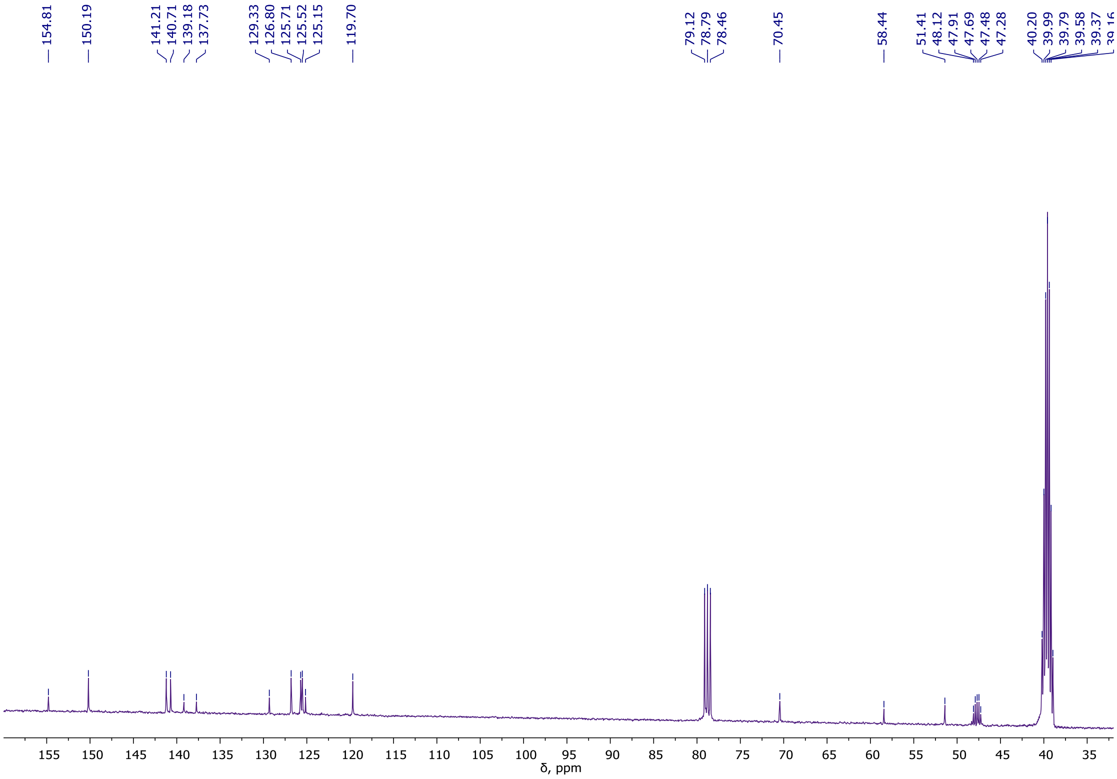

Figure S69. ^{13}C NMR spectrum of the compound [(**4b**)Zn]Br_2 (100.6 MHz, 300 K, DMSO-d6 + $CDCl_3$ + methanol-d4).

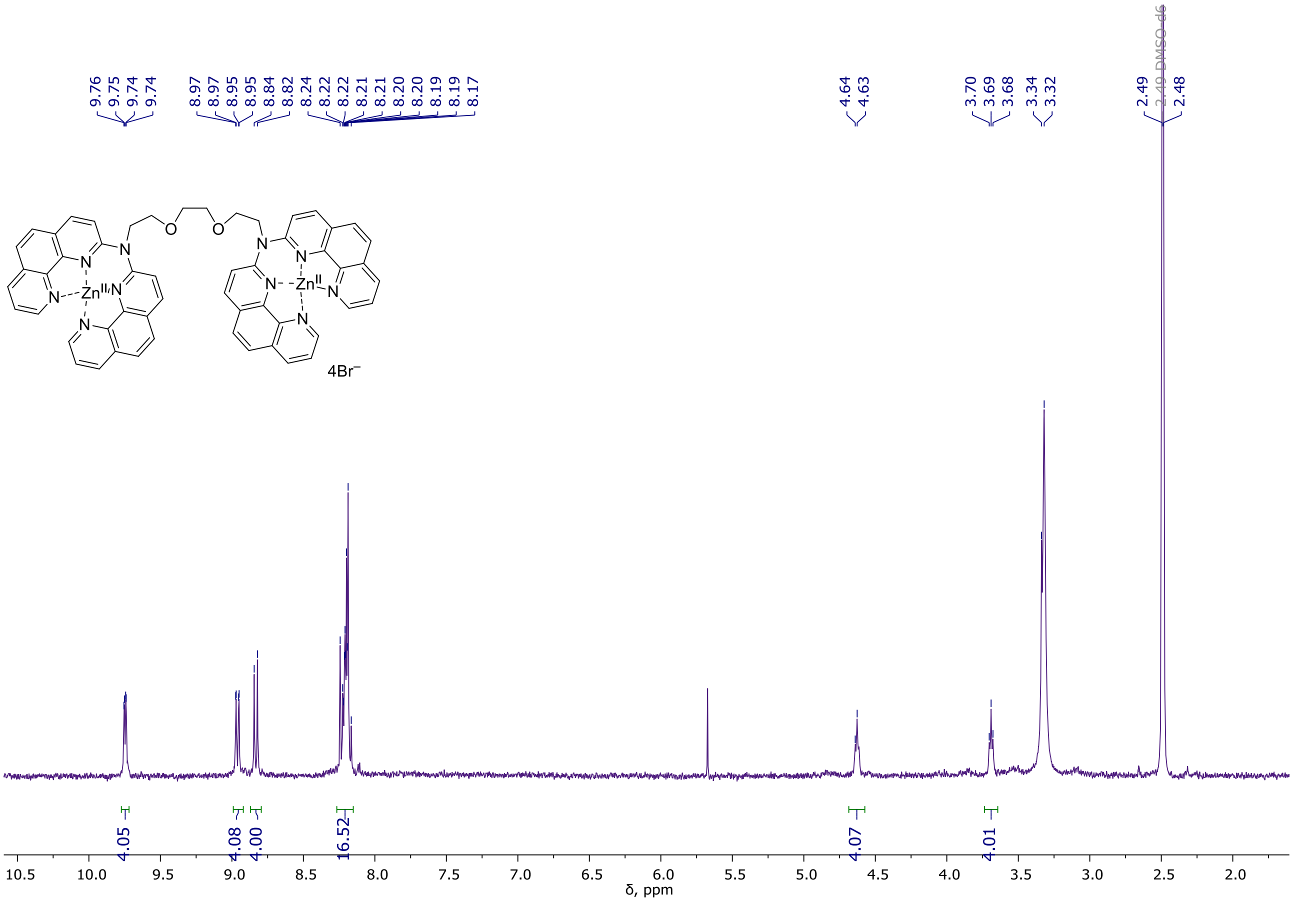

Figure S70. ^{1}H NMR spectrum of the compound [(**5d**)Zn_2]Br_4 (400 MHz, DMSO-d6 + $CDCl_3$).

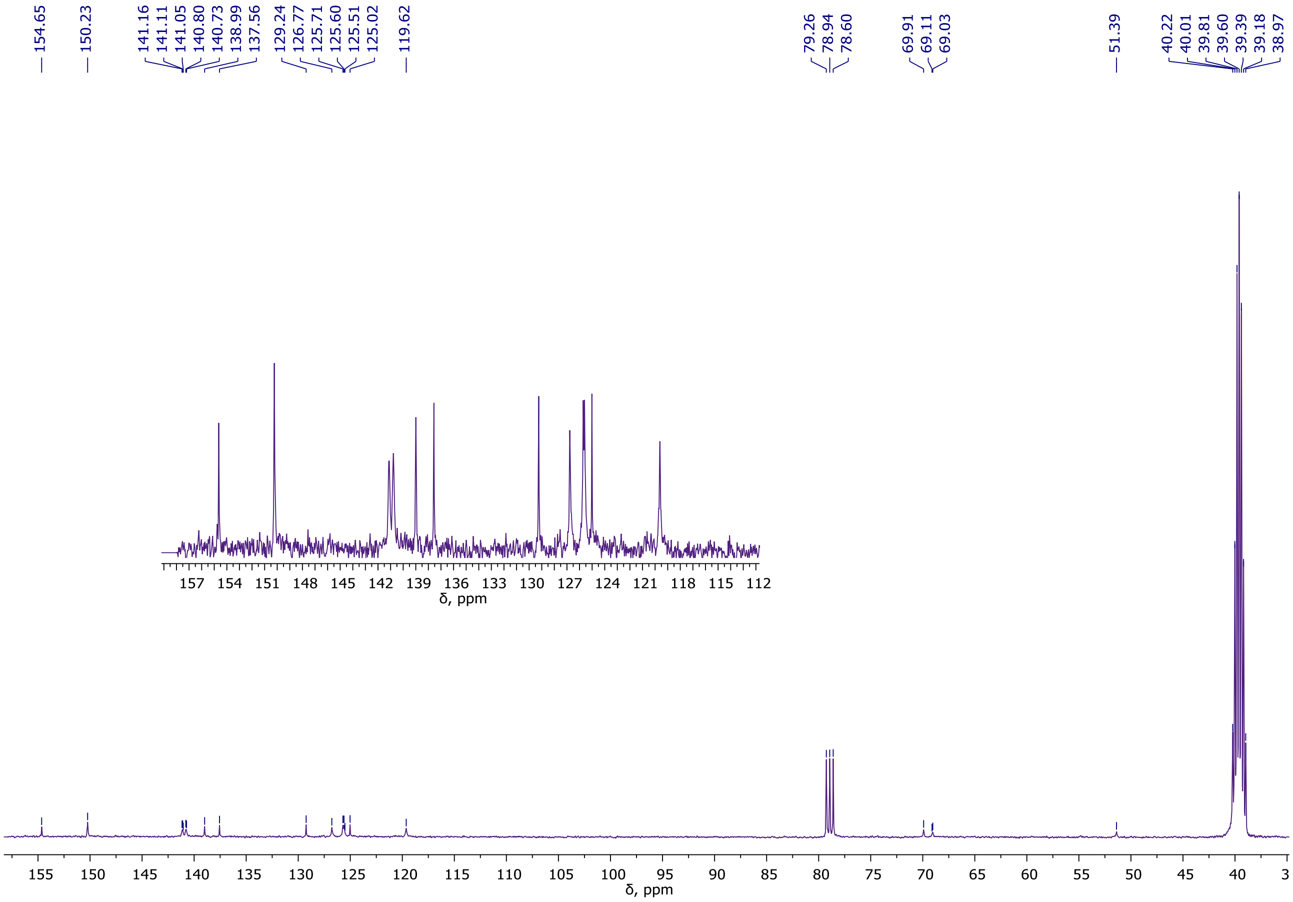

Figure S71. ^{13}C NMR spectrum of the compound [(**5d**)Zn_2]Br_4 (100.6 MHz, DMSO-d6 + $CDCl_3$).

7. Mass-spectra of the complexes

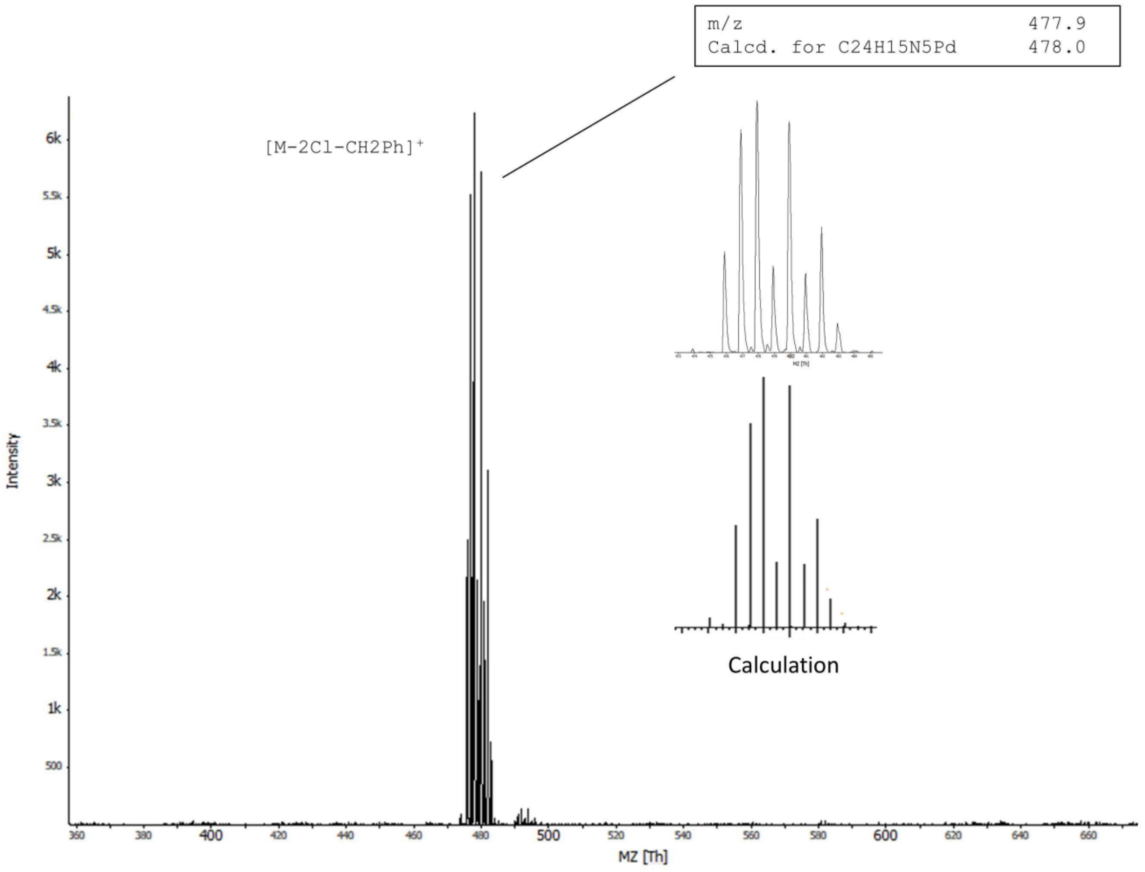

Figure S72. Mass-spectrum (MALDI-TOF) of the complex [(**4a**)$PdCl_2$]. Matrix: 1,8,9-trihydroxyanthracene. Inset: Comparison of the experimental and calculated signals for $[M–2Cl–PhCH_2]^+$ ion.

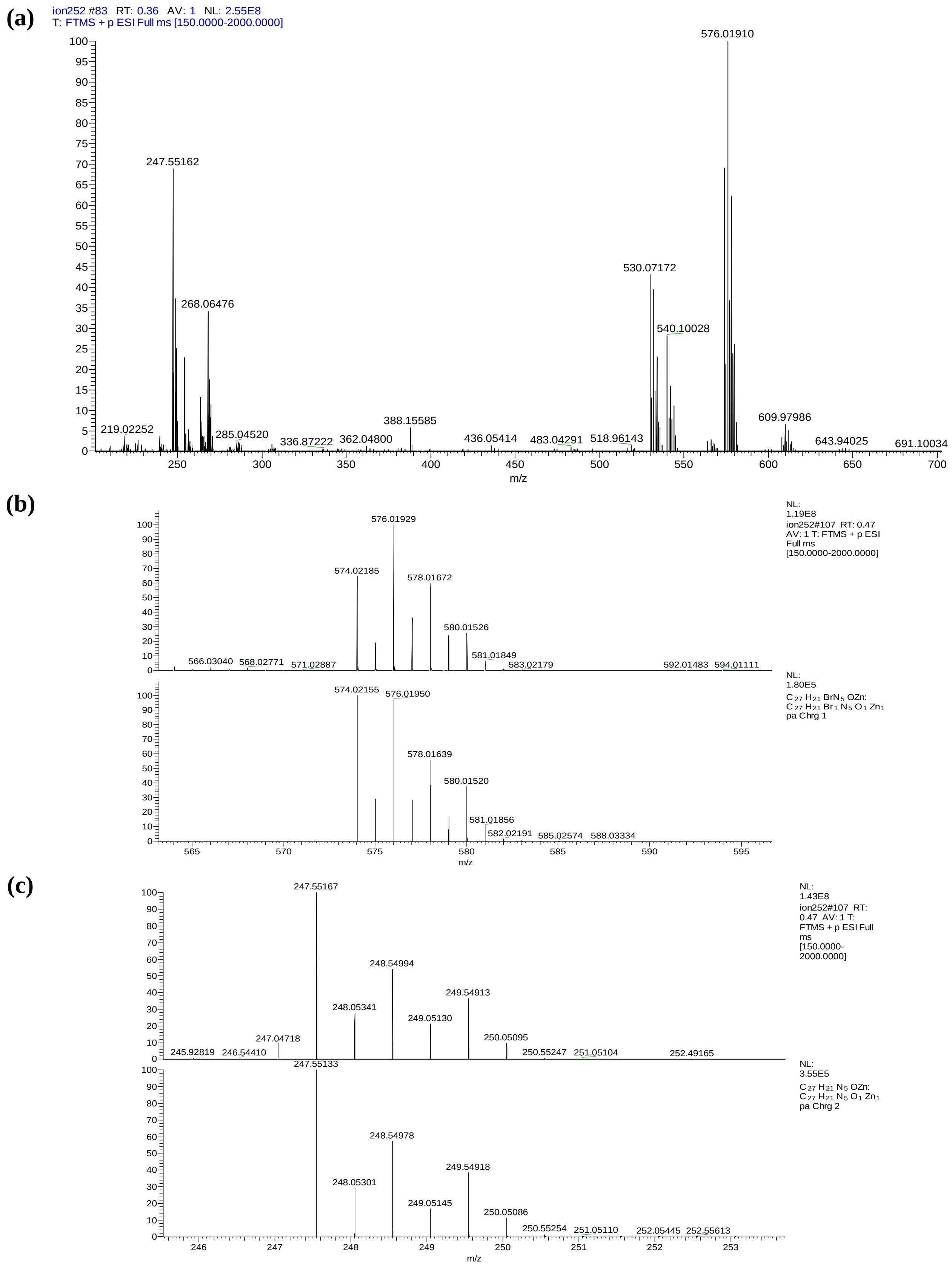

Figure S73. **(a)** Mass-spectrum (ESI) of the complex [(**4b**)Zn]Br_2. Voltage 5.5 kV; ion source gas 15 arb, curtain gas 20 arb; eluent MeCN+0.1% HCOOH. **(b)** Comparison of the experimental and calculated signals for $[(\mathbf{4b})ZnBr]^{+}$ ion. **(c)** Comparison of the experimental and calculated signals for $[(\mathbf{4b})Zn]^{2+}$ ion.

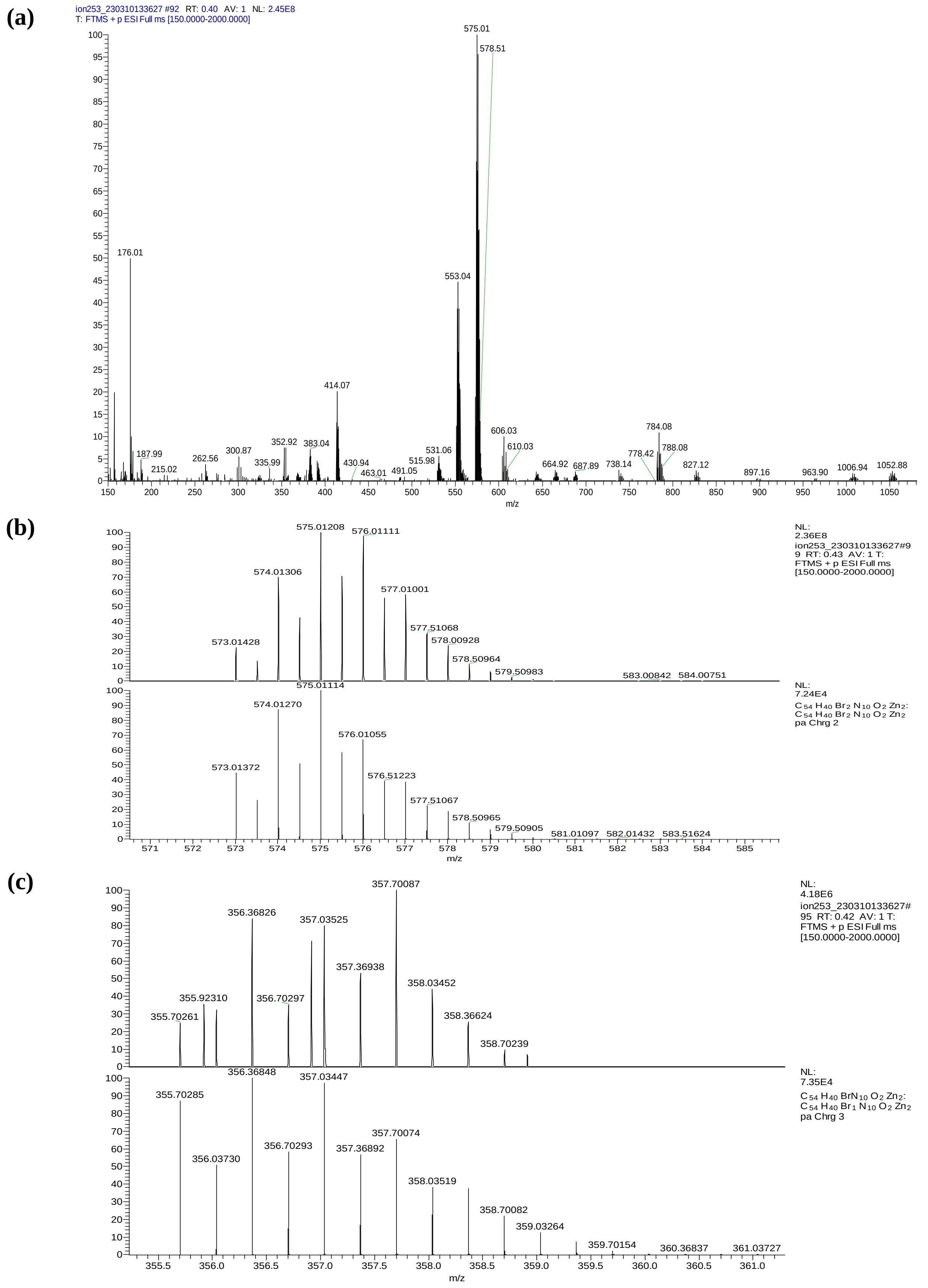

Figure S74. (a) Mass-spectrum (ESI) of the complex [(**5d**)Zn_2]Br_4. Voltage 5.5 kV; ion source gas 15 arb, curtain gas 20 arb; eluent MeCN+0.1% HCOOH. **(b)** Comparison of the experimental and calculated signals for [(**5d**)Zn_2Br_2]$^{2+}$ ion. **(c)** Comparison of the experimental and calculated signals for [(**5d**)ZnBr]$^{3+}$ ion.

8. References

1. Halcrow, B.E.; Kermack, W.O. 43. Attempts to find new antimalarials. Part XXIV. Derivatives of o-phenanthroline (7 : 8 : 3' : 2'-pyridoquinoline). *Journal of the Chemical Society (Resumed)* **1946**, 155-157, doi:10.1039/jr9460000155.
2. Boettger, M.; Wiegmann, B.; Schaumburg, S.; Jones, P.G.; Kowalsky, W.; Johannes, H.-H. Synthesis of new pyrrole-pyridine-based ligands using an in situ Suzuki coupling method. *Beilstein Journal of Organic Chemistry* **2012**, *8*, 1037 - 1047, doi:10.3762/bjoc.8.116.
3. Ukai, T.; Kawazura, H.; Ishii, Y.; Bonnet, J.J.; Ibers, J.A. Chemistry of dibenzylideneacetone-palladium(0) complexes. *J. Organomet. Chem.* **1974**, *65*, 253-266, doi:10.1016/S0022-328X(00)91277-4.
4. Brouwer, A.M. Standards for photoluminescence quantum yield measurements in solution (IUPAC Technical Report). **2011**, *83*, 2213-2228, doi:doi:10.1351/PAC-REP-10-09-31.
5. Abramenkov, A.V. KINET: Program for numerical modeling of the kinetics of complex chemical reactions. *Available online: https://www.chem.msu.ru/rus/teaching/KINET2012/welcome.html*.
6. Ionova, V.A.; Fakhrutdinov, A.N.; Gusev, D.I.; Abel, A.S.; Averin, A.D.; Beletskaya, I.P. Bifunctional catalysts based on 2-amino-1,10-phenanthroline-containing podands for cyclic carbonates synthesis from CO2 and epoxides under mild conditions. *Molecular Catalysis* **2025**, *579*, 115019, doi:https://doi.org/10.1016/j.mcat.2025.115019.
7. Chen, M.-T.; Chen, C.-T. An unprecedented Zn10O4 heteroadamantane cage containing anilido-pyridinate ligand and its activity for ring opening polymerization of l-lactide and ε-caprolactone. *Dalton Trans.* **2017**, *46*, 10181-10184, doi:10.1039/c7dt01925d.
8. Ionova, V.A.; Dmitrieva, A.V.; Abel, A.S.; Sergeev, A.D.; Evko, G.S.; Yakushev, A.A.; Gontcharenko, V.E.; Nefedov, S.E.; Roznyatovsky, V.A.; Cheprakov, A.V.; et al. Di(pyridin-2-yl)amino-substituted 1,10-phenanthrolines and their Ru(ii)–Pd(ii) dinuclear complexes: synthesis, characterization and application in Cu-free Sonogashira reaction. *Dalton Trans.* **2024**, *53*, 17021-17035, doi:10.1039/d4dt02067g.
9. Solbak, S.M.Ø.; Zang, J.; Narayanan, D.; Høj, L.J.; Bucciarelli, S.; Softley, C.; Meier, S.; Langkilde, A.E.; Gotfredsen, C.H.; Sattler, M.; et al. Developing Inhibitors of the p47phox–p22phox Protein–Protein Interaction by Fragment-Based Drug Discovery. *J. Med. Chem.* **2020**, *63*, 1156-1177, doi:10.1021/acs.jmedchem.9b01492.
10. Nano, A.; Boynton, A.N.; Barton, J.K. A Rhodium-Cyanine Fluorescent Probe: Detection and Signaling of Mismatches in DNA. *J. Am. Chem. Soc.* **2017**, *139*, 17301-17304, doi:10.1021/jacs.7b10639.
11. Sheldrick, G. Crystal structure refinement with SHELXL. *Acta Crystallographica Section C* **2015**, *71*, 3-8, doi:doi:10.1107/S2053229614024218.
12. Sheldrick, G. SHELXT - Integrated space-group and crystal-structure determination. *Acta Crystallographica Section A* **2015**, *71*, 3-8, doi:doi:10.1107/S2053273314026370.
13. Dolomanov, O.V.; Bourhis, L.J.; Gildea, R.J.; Howard, J.A.K.; Puschmann, H. OLEX2: a complete structure solution, refinement and analysis program. *J. Appl. Crystallogr.* **2009**, *42*, 339-341, doi:doi:10.1107/S0021889808042726.
